# Nitrene generation and transfer for unnatural biosynthesis in living cells

**DOI:** 10.1038/s41557-026-02224-4

**Published:** 2026-08-18

**Authors:** Isaac Donnell, Andrew Quest, Jeremy Tang, Maodun Zhang, Brandon Bloomer, Douglas S. Clark, Aindrila Mukhopadhyay, John Hartwig, Jay D. Keasling

**Affiliations:** 1 Department of Chemistry, University of California, Berkeley, Berkeley, CA USA; 2 Joint BioEnergy Institute, Lawrence Berkeley National Laboratory, Emeryville, CA USA; 3 Chemical Sciences Division, Lawrence Berkeley National Laboratory, Berkeley, CA USA; 4 College of Natural Resources, University of California, Berkeley, Berkeley, CA USA; 5 Department of Molecular and Cell Biology, University of California, Berkeley, Berkeley, CA USA; 6 Department of Chemical and Biomolecular Engineering, University of California, Berkeley, Berkeley, CA USA; 7 Molecular Biophysics and Integrated Bioimaging Division, Lawrence Berkeley National Laboratory, Berkeley, CA USA; 8 Biological Systems and Engineering Division, Lawrence Berkeley National Laboratory, Berkeley, CA USA; 9 Environmental Genomics and Systems Biology Division, Lawrence Berkeley National Laboratory, Berkeley, CA USA; 10 Department of Bioengineering, University of California, Berkeley, Berkeley, CA USA

**Keywords:** Biosynthesis, Biocatalysis

## Abstract

Synthetic biology has enabled the production of natural and unnatural products from inexpensive sustainable feedstocks. Yet, the scope of available products has been limited largely to compounds accessible from nature’s chemical reactions. Evolved enzymes can catalyse reactions of unnatural substrates or reactions not found in nature but often require the addition of synthetic reagents to purified enzymes or to resting cells containing those enzymes. Here we show that chemical reactions of metabolic intermediates produced intracellularly in living cells can include intermolecular nitrene transfers. The biosynthesis of *N*-acetoxyanilines, in combination with the generation and transfer of *N*-aryl nitrene intermediates from them catalysed by a cytochrome P450, generates amino alcohols, diamines, diarylamines and aminoalkyl arenes from simple carbon feedstocks. These products—common substructures of pharmaceuticals and agrochemicals—are challenging to synthesize by standard organic chemistry and were produced from inexpensive, renewable feedstocks. Evolution of the enzymes in this pathway showed that titres can be increased by engineering and that the products can be synthesized with high enantioselectivity.

**Figure:**
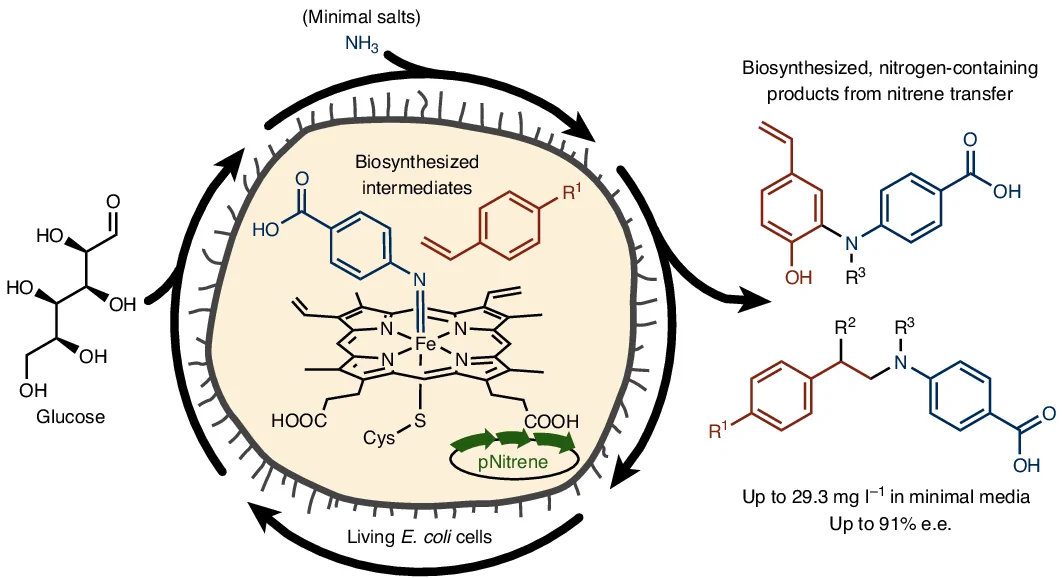

## Abstract (web summary)

The incorporation of unnatural chemical reactions into biosynthetic pathways is a key aim of synthetic biology. Now it has been shown that a nitrene precursor derived from the natural metabolite *p*-aminobenzoic acid can be generated through biosynthesis in *Escherichia coli* and transferred to several biosynthesized olefins to form new-to-nature amino alcohol, diamine and diarylamine products.

## Main

**Fig. 1:**
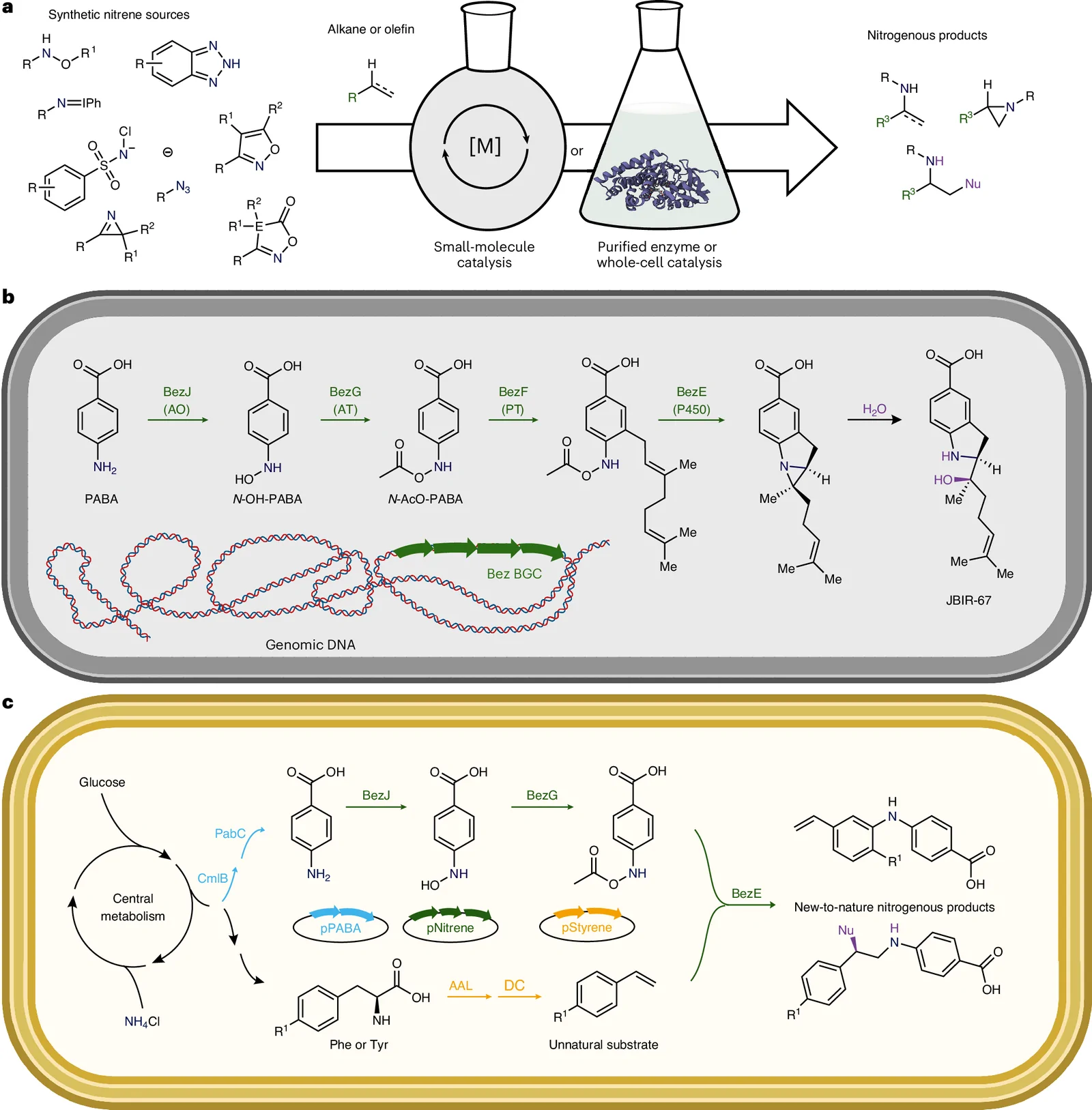
Nitrene generation and transfer for unnatural biosynthesis in cells. **a**, Prior examples of nitrene generation and transfers from a variety of nitrene precursors using small molecule catalysts, purified enzymes and enzymes in whole cells. **b**, Biosynthetic pathway of JBIR-67 in *Streptomyces* sp. RI18 as elucidated by Tsutsumi and coworkers^17^, which contains an intramolecular nitrene transfer as a step. Green reaction arrows indicate genes central to nitrene precursor biosynthesis and nitrene transfer in *Streptomyces* sp. RI18. AT, acyl transferase; PT, prenyl transferase; P450, cytochrome P450. **c**, This work: incorporation of nitrene generation and intermolecular nitrene transfer as part of an artificial biosynthetic pathway in *E. coli* for the purpose of making new-to-nature, nitrogen-containing products. Blue reaction arrows indicate PABA biosynthesis by enzymes expressed from pPABA. Yellow reaction arrows indicate styrene or 4-VP biosynthesis by enzymes expressed from pStyrene. Green reaction arrows indicate nitrene precursor biosynthesis and nitrene transfer by enzymes expressed from pNitrene. Diagrams in **b** and **c** created in BioRender; Quest, A. https://biorender.com/r4vj5yr (2026). CmlB, *S*. *ven*e*zuelae* Aminodeoxychorismate synthase; AAL, aromatic amino acid lyase; DC, decarboxylase.

Substituted arylamines constitute a broad and important class of structure found in many drug-like molecules^1–6^. Within this broad class, 1,2-amino alcohols^7^, 1,2-diamines^8^, *N*-alkylarylamines^9^ and diarylamines^10,11^ represent some of the most common structural motifs found in pharmaceutical agents. While the synthesis of nitrogen-containing molecules is conducted by a wide range of chemical transformations, including the intra- and intermolecular transfer of nitrenes to alkyl C–H bonds and olefins to generate a variety of amine products^12–14^ (Fig. 1a), the biosynthesis of nitrogen-containing natural products canonically occurs by a more narrow set of reactions, most commonly by transformations of amino acids or by the conversion of alcohols or ketones to amines^15,16^. In 2018, Tsutsumi and colleagues discovered that certain species of *Streptomyces* produced benzastatin natural products via an intramolecular nitrene transfer^17^ (Fig. 1b). We envisioned that an artificial biosynthetic pathway^18–20^ could be created in which intermolecular nitrene transfer would occur intracellularly with nitrene donors and acceptors generated biosynthetically. Such a pathway could then produce nitrogen-containing, unnatural products, and the entire sequence could be incorporated into a living cell to produce these unnatural products from simple salts and sugars, expanding the biosynthetic toolkit and enabling sustainable production of these important motifs. Microbial biosynthesis has been instrumental in the production of complex natural products on preparative scales^21–23^, and the addition of nitrene transfer into intracellular metabolism should create new capabilities for the isolation and diversification of bioactive and pharmaceutically relevant compounds.

In the last three decades, enzyme engineering has led to the intra- and intermolecular transfer of nitrenes to various acceptors, but in all examples, the nitrene precursors in these reactions were produced by chemical synthesis and added exogenously to cells or purified enzymes (Fig. 1a). Nitrene intermediates can be generated chemically from a wide variety of precursors^12,24–26^, but, of these precursors, only substituted hydroxylamines and azides are found in nature. Thus, we considered that the biosynthesis of *N*-acetoxyamines, which is part of the pathway to benzastatins, could enable the generation of nitrene intermediates within artificial biosynthetic pathways that form unnatural, nitrogen-containing products.

Here, we report the simultaneous biosynthesis of an acetoxyamine precursor to aryl nitrenes, biosynthesis of a series of vinylarenes and expression of a P450 enzyme that catalyses the nitrene-transfer reaction between the two to create new-to-nature biosynthetic pathways. These pathways form multiple nitrogen-containing products, including hydroxylamines, alkyl arylamines and diarylamines, some of which are formed by reactions not yet known to occur with P450 enzymes. Most generally, these results show that unnatural products can be produced from common cellular components by synthetic sequences that include the formation of carbon–nitrogen bonds by enzymatic reactions new to living systems.

## In vitro generation of the nitrene precursor and intermolecular transfer

**Extended Data Fig. 1:**
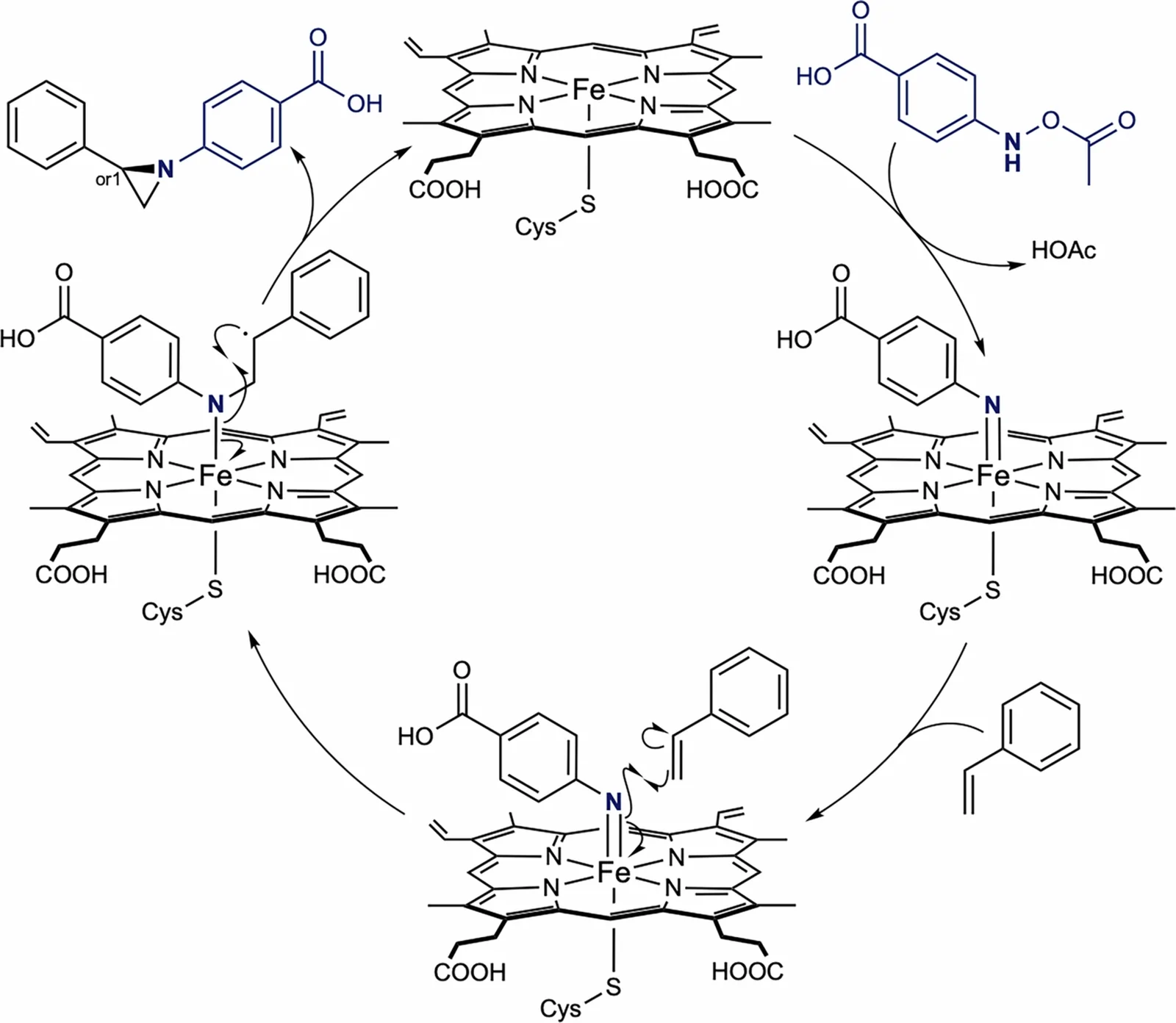
Proposed mechanism for aziridine formation. Proposed mechanism for the formation of aziridine intermediate from reactants *N*-AcO-PABA and styrene in the active site of hemoprotein BezE.

To create an artificial biosynthetic pathway involving nitrene transfer, we conducted experiments to repurpose enzymes in the benzastatin biosynthetic pathway. This pathway was recently shown to occur by prenylation of a *p*-(*N*-acetoxy)-aminobenzoic acid (*N*-AcO-PABA), intramolecular aziridination of the proximal alkene of the prenyl unit catalysed by a P450 with a nitrene generated from the *N-*acetoxyamine and ring opening of the aziridine by water or chloride^17^ (Fig. 1b and Extended Data Fig. 1). We reasoned that the full biosynthetic pathway to *N-*AcO-PABA could be used independently to produce *N*-acetoxyamines, which would undergo intermolecular nitrene transfers as part of unnatural biosynthetic sequences to form unnatural nitrogen-containing products (Fig. 1c).

**Fig. 2:**
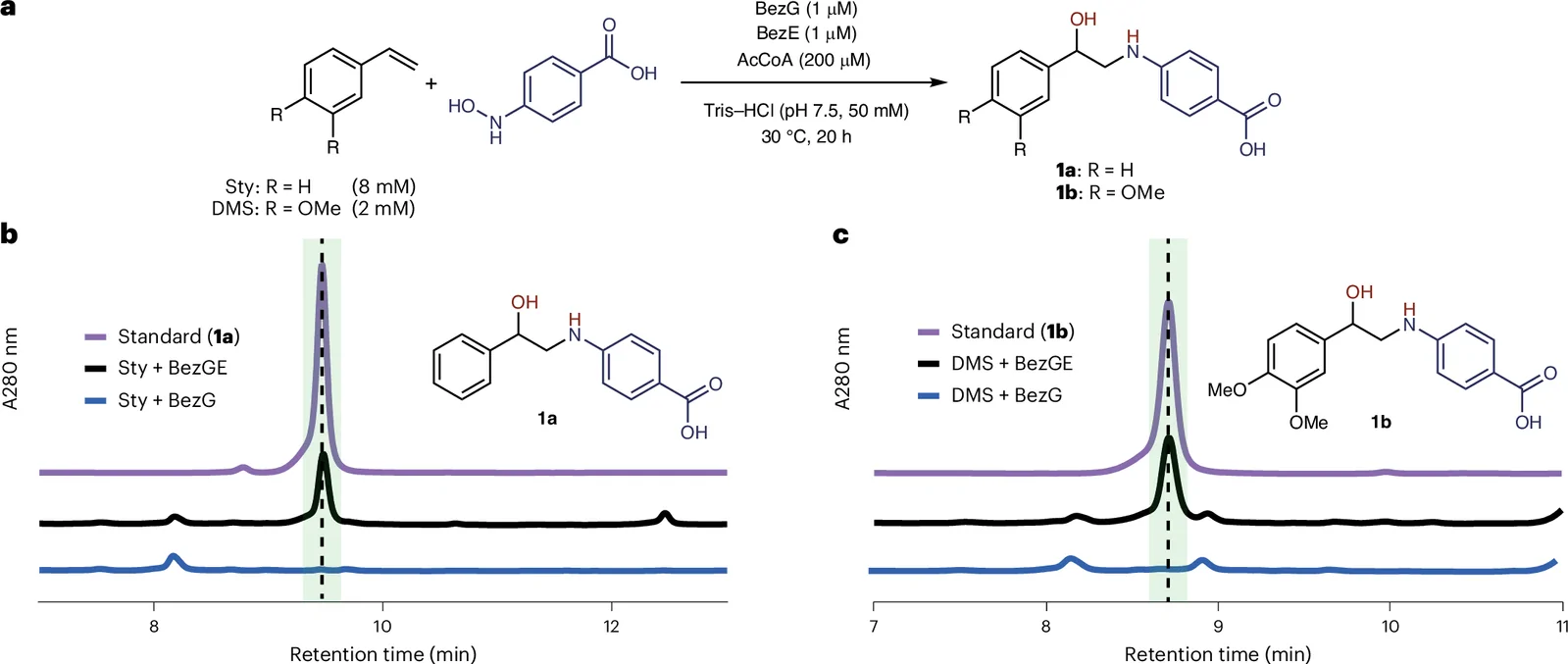
Development of the intermolecular nitrene transfer reaction. **a**, Reaction scheme for in vitro intermolecular nitrene transfer. **b**,**c**, Liquid chromatography with UV–Vis analysis of in vitro addition of PABA to styrene (**b**) or dimethoxystyrene (**c**) by nitrene transfer. The amino alcohol products (**1a** or **1b**) are produced from 4-hydroxylaminobenzoic acid, styrenyl substrate and AcCoA with the addition of purified BezG and BezE. Substrate concentrations were 200 μM *N*-OH-PABA and 8 mM styrene or 2 mM DMS unless otherwise indicated.

**Extended Data Fig. 2:**
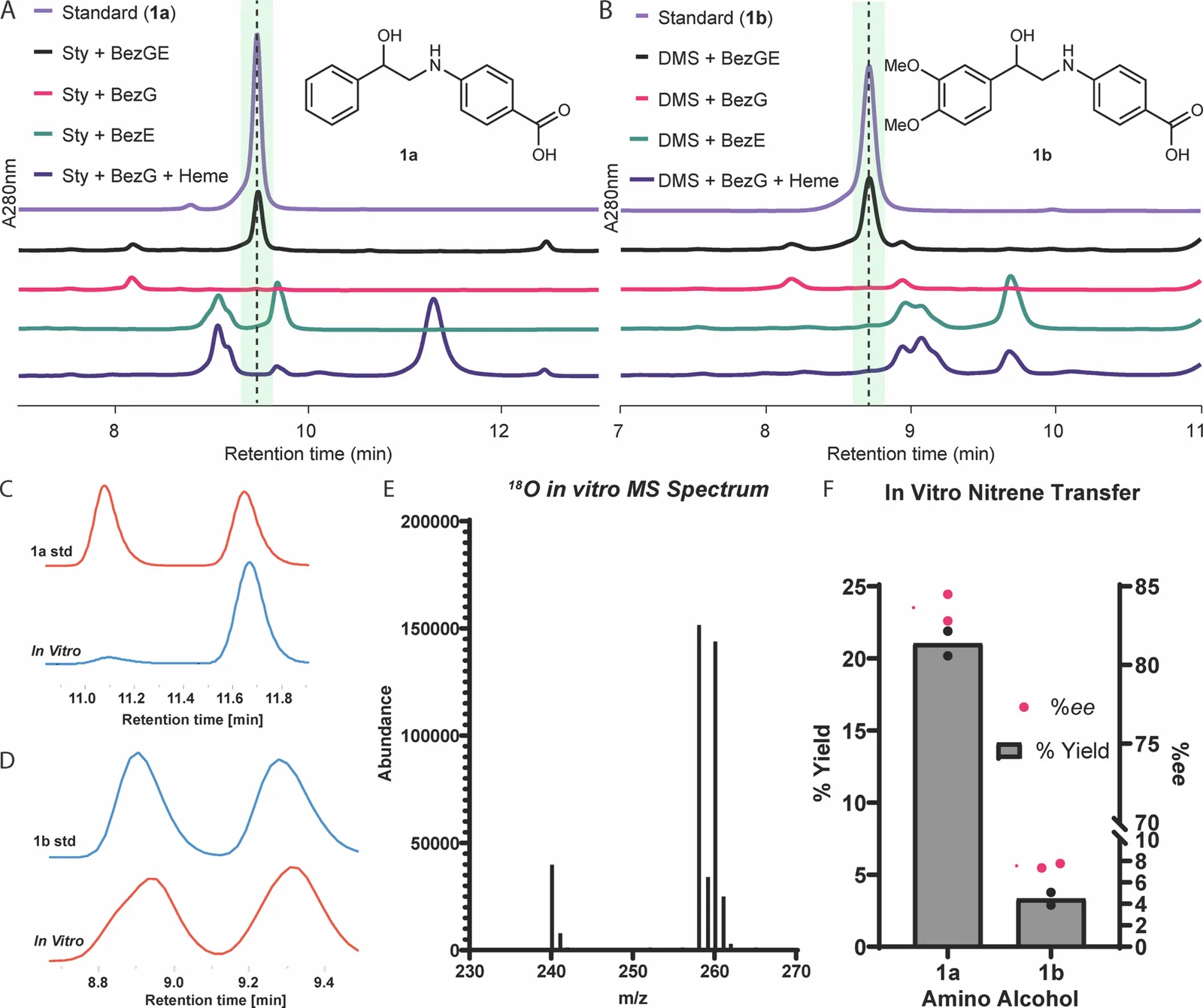
Analysis of biocatalytic reactions with styrene and dimethoxystyrene as substrates. (**a**) In vitro reactions showing dependence on both BezE and BezG for production of amino alcohol **1a**. (**b**) In vitro reactions showing dependence on both BezE and BezG for production of amino alcohol **1b**. (**c**) LC-UV/Vis chromatograms (Abs_280nm_) of a racemic standard (top) of **1a** and of **1a** produced in the in vitro nitrene transfer reaction catalyzed by BezE (bottom). (**d**) LC-MSD (SIM, 318 *m/z*) of a racemic standard of **1b** (top) and of **1b** produced in the in vitro nitrene transfer reaction catalyzed by BezE (bottom). SIM was used over UV-Vis due to co-eluants in the in vitro reaction. (**e**) Mass spectrum of compound **1a** produced in vitro in 1:1 ^16^O:^18^O water demonstrates 1:1 M:M+2 peaks in positive mode. (**f**) In vitro yields and *ee*’s of **1a** and **1b**. Source data

To determine the feasibility of these putative biosynthetic pathways, we tested the in vitro reaction of *N-*AcO-PABA with styrene catalysed by BezE_RI18_ from *Streptomyces* sp. RI18 (ref. ^17^). This enzyme was shown previously to catalyse unnatural, intramolecular transfers of a nitrene from a sulfonyl azide^27^ to a C–H bond and intermolecular transfer of a carbene from a diazoester to styrene^28^. To test for reactions involving a nitrene intermediate, we generated *N-*AcO-PABA in situ by the reaction of *p*-hydroxyamino benzoic acid (*N*-OH-PABA) with acetyl-CoA (AcCoA), as the source of the acetyl group, catalysed by BezG_niv_ from *Streptomyces niveus*. We chose to use BezG_niv_ because Tsusumi et al. were unable to purify a functional BezG_RI18_ and used BezG_niv_ as a homologue of BezG_RI18_ for their analysis. The in vitro reaction of *N-*AcO-PABA with styrene catalysed by BezE_RI18_ formed the amino alcohol **1a** from nitrene transfer and hydrolytic ring opening of the aziridine (Fig. 2) in 21% yield from 200 μM AcCoA and with 85% enantiomeric excess (e.e.; Fig. 2b and Extended Data Fig. 2a,c,f). The identity of **1a** was confirmed by comparison of the liquid chromatography (LC)–mass spectrometry (MS) trace to that of independently synthesized authentic material. Control reactions conducted in the absence of purified BezG_niv_ or BezE_RI18_, or with added free heme, showed that the reaction with styrene depends on both BezG_niv_ and BezE_RI18_. Some of the amino alcohol was detected in the absence of BezE_RI18_, but the amount was less than 3% (as determined by the extracted ion chromatogram (EIC) peak area) of that formed by the reaction catalysed by BezG_niv_ and BezE_RI18_ (Extended Data Fig. 2a).

To probe the promiscuity of the aziridination catalysed by BezE_RI18_, we conducted the in vitro reaction of DMS (2 mM) with *N-*AcO-PABA catalysed by purified BezE_RI18_. The amino alcohol **1b**, whose structure was again determined by independent synthesis, was the sole product of the reaction (Fig. 2c), albeit with an initial yield (3.3%) and e.e. (8%) much lower than those of the product from reactions with styrene (Extended Data Fig. 2b,d,f). The uncatalysed reaction produced just 1% of the amount of **1b** formed by the reaction catalysed by BezE_RI18_ (as determined by the EIC peak area). We then allowed *N-*AcO-PABA to react with styrene in 50% H_2_^18^O, catalysed by purified BezE_RI18_, to determine if such hydrolysis was inherent to the reaction, as seen with previous enzymatic nitrene transfers^29–31^, or if it occurred during LC–MS analysis. The MS indicated that the heavy oxygen from the water medium was incorporated into the product, confirming that hydrolysis occurred during the in vitro reaction and before LC–MS analysis (Extended Data Fig. 2e).

## Results

### In vivo generation of the nitrene precursor and pathway optimization

**Fig. 3:**
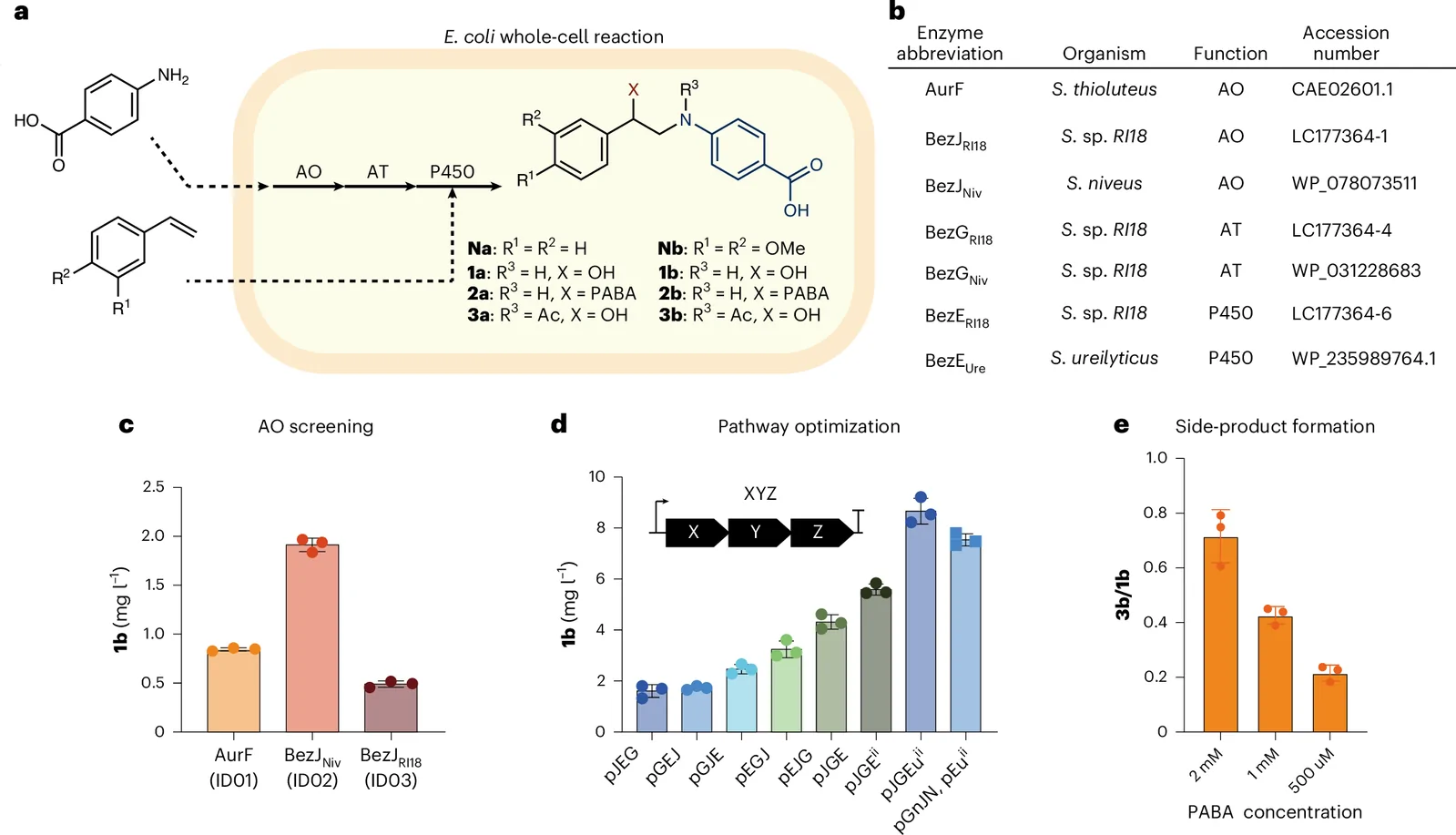
Development of the in vivo intermolecular nitrene transfer reaction. **a**, Scheme for biocatalytic nitrene transfer. **b**, Table describing the enzyme abbreviations, their organism of origin, function and accession number. **c**, Screening AO. BezG and BezE were also expressed in these experiments. **d**, Testing all permutations of the operon to identify the gene order that provides the highest product titre. JGE_Ure_, which provided the highest titre, contains the *S. ureilyticus* BezE homologue. The *X* axis labels indicate the gene order in the operon. Substrate concentrations were 250 μM PABA and 2 mM DMS, unless otherwise indicated. Condition II substrate concentrations are 500 μM PABA and 2 mM DMS. Unless the bacterial source for the gene is indicated, J is BezJ from *S. niveus*, E is BezE from *Streptomyces* sp. RI18 and G is BezG from *S. niveus*. **e**, Product ratio of diamine **3b** and amino alcohol **1b** as a function of concentration of PABA. Data are mean ± s.d. *n* = 3 biological replicates. Source data

Having observed products of apparent nitrene transfer, we sought to incorporate both the generation and reaction of this nitrene into an artificial biosynthetic pathway in vivo. To do so, we envisioned that the enzymatic oxidation of PABA to generate the corresponding hydroxylamine *N*-OH-PABA and acylation of this hydroxylamine could be conducted in vivo before transfer of the nitrene catalysed by a cytochrome P450 (Fig. 3a). We tested a series of arylamine oxidases (AOs, including BezJ from *Streptomyces* sp. Rl18 (BezJ_RI18_) and *S. niveus* (BezJ_niv_) and AurF from *Streptomyces thioluteus*^32–35^) (Fig. 3b) that would oxidize the amino group in PABA in vivo (Fig. 3c). BezJ_niv_ was chosen as an alternative in case the low in vitro activity for BezG_RI18_^17^ was also seen for BezJ_RI18_. AurF was included because it catalyses the oxidation of PABA to *p*-nitrobenzoic acid via the hydroxylamine, which we could trap by acylation^33^; as such, AurF functioned as a positive control for BezJ activity. To catalyse the acylation of the resulting hydroxylamine, we used BezG_niv_.

Each of the three arylamine oxygenases (AOs) was co-expressed in *Escherichia* *coli* BL21 Star DE3 with the acetyltransferase BezG_niv_ and the P450 BezE_RI18_; the genes encoding oxygenases and BezG_niv_ were placed under control of individual Trc promoters on one plasmid, and the gene encoding BezE_RI18_ was placed under control of the T7 promoter on a second plasmid (pGnAf, pGnJr and pGnJn for the oxygenases and pEr for BezE; Supplementary Table 1). Following expression in rich medium, the cells were concentrated and resuspended in minimal medium to an optical density (OD) of 30, and the reactants PABA and 3,4-dimethoxystyrene (DMS, chosen because DMS is less volatile than styrene and the product amino alcohol **1b** absorbs in the UV region more strongly than does the amino alcohol **1a**) were added exogenously to the cells (Fig. 3a). All three organisms (strains ID01–ID03; Supplementary Table 2), each expressing a different AO, produced detectable amounts of amino alcohol **1b**, as determined by comparing the high-performance liquid chromatography (HPLC) retention time and LC–MS extracted ion mass to those of the product generated from the in vitro experiments and by independent chemical synthesis. BezJ_niv_ was more active in vivo (2 mg l^−1^ **1b**) than BezJ_RI18_ (0.5 mg l^−1^ **1b**) and was more productive than AurF in this system (0.86 mg l^−1^ **1b**) (Fig. 3c). Thus, our next set of experiments were conducted with BezG_niv_, BezJ_niv_ and BezE_RI18_.

We sought to increase the production of the aziridine and its ring-opened products by testing all possible arrangements of the genes encoding BezE_RI18_, BezG_niv_ and BezJ_niv_ in a single operon, on the basis of the premise that the position of the enzyme in the operon could affect the amount of enzyme translated^36–38^. The construct containing *bezJGE* led to the highest production of amino alcohol **1b** after reaction optimization (5.6 mg l^−1^ and 12% e.e.; Fig. 3d).

**Extended Data Fig. 3:**
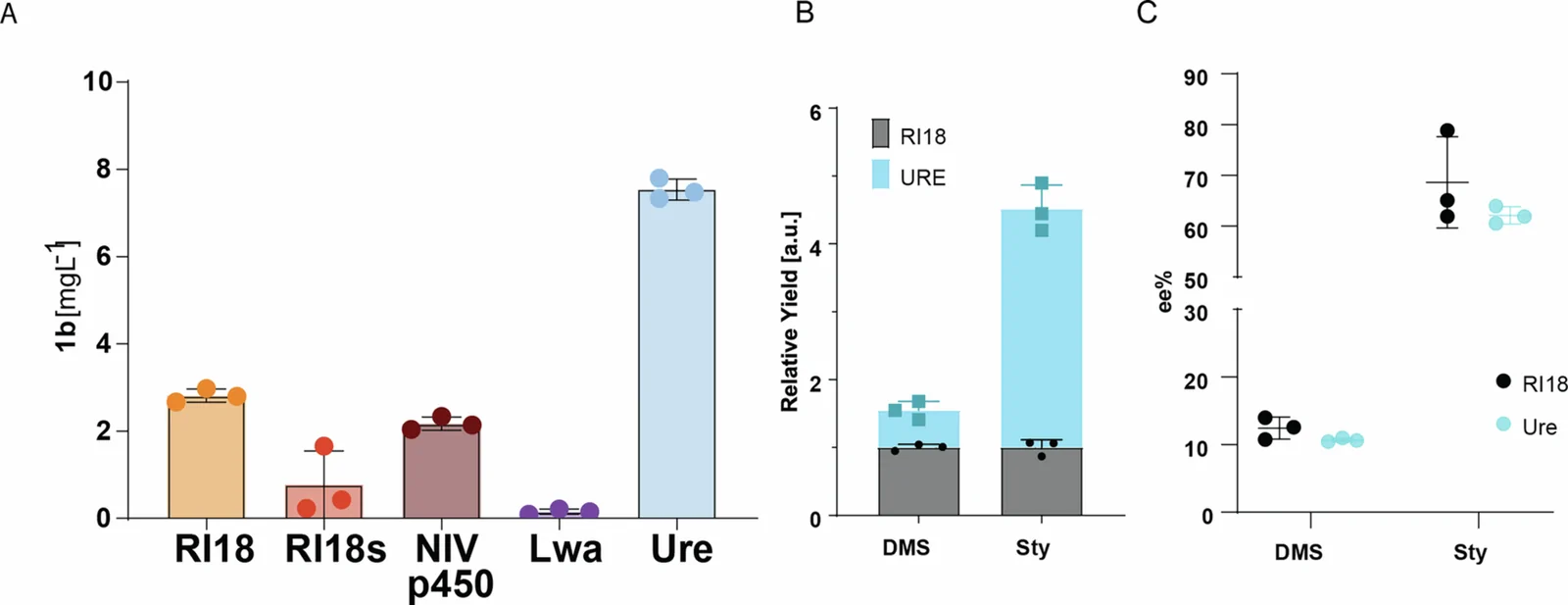
Comparison of BezE homologues for the reactions of styrene and dimethoxystyrene. (**a**) Titers of **1b** from experiments testing chosen P450 homologues and mutants. RI18s is the C365S mutant of BezE_RI18_. All cells were co-transformed with pGnJn and each P450 was in the pEr backbone. Accession numbers and sequence identities for each homologue can be found in Supplementary Table 5. (**b**) Nitrene transfer reactions to various vinyl arenes, comparing the productivity of BezE_RI18_ and BezE_Ure_ in the JGE and JGEu constructs, respectively. (**c**) Enantioselectivity of BezE_RI18_ vs BezE_Ure_ in the JGE and JGEu constructs, respectively. Cells were grown and induced in HB medium before resuspension in M9-HG reaction buffer. Substrate concentrations are 500 uM PABA, 2 mM DMS, 40 mM Sty. Data are mean ± s.d. *n*=3 biological replicates. Source data

Given our results, and those reported previously^17^, demonstrating that BezJ_niv_ and BezG_niv_ from *S. niveus* are more active for the oxidation and acylation of PABA and *N*-OH-PABA than are those from *Streptomyces* sp. RI18, we tested the activity of several homologues of the P450 BezE (identified by BLAST^39^ from *S. niveus*, *Lentzea waywayandensis* and *Streptomyces ureilyticus*; Extended Data Fig. 3a and Supplementary Table 5) for the addition of the nitrene derived from PABA to the vinylarene DMS. We also constructed the C365S mutant of BezE_RI18_, a mutation commonly made to increase nitrene transfer activity of p450s^29,40^, but its activity was lower than that of the native BezE_RI18_.

The most reactive BezE homologue we identified was that from *S. ureilyticus*, BezE_Ure_, for which the sequence similarity to BezE_RI18_ is 75%. Replacing the gene encoding BezE_RI18_ with BezE_Ure_ in the two-plasmid system (pGnJn and pEu) or the one-plasmid system (creating plasmid pJGEu, strain ID04) with addition of DMS and PABA increased production of the amino alcohol **1b** by 2.7-fold (Extended Data Fig. 3b) and 1.5-fold (Fig. 3d), respectively. The final titre of **1b** was 8.7 mg l^−1^ from pJGEu (Fig. 3d). To test if the higher activity of BezE_Ure_ would be observed for reactions of additional vinylarenes, we conducted the reaction with styrene and observed that the titre of amino alcohol **1a** from the reaction of BezE_Ure_ was also greater (five times in this case) than that from BezE_RI18_ (Extended Data Fig. 3b). Finally, the single plasmid system pJGEu (strain ID04) was more productive than the two-plasmid analogue harbouring individually transcribed genes (strain ID02), even when BezE_Ure_ was used (Fig. 3d).

The enantioselectivities (Extended Data Fig. 3c) of the amino alcohol **1a** produced from styrene (62% with BezE_Ure_ and 68% with BezE_RI18_) were much higher than those of the amino alcohol **1b** produced from DMS (11% with BezE_Ure_ and 12% with BezE_RI18_). We presume that the enantioselectivity for addition of the nitrene to dimethoxystyrene is similar to that of styrene but that ring opening of the resulting aziridine to form the amino alcohol is not stereospecific. The lower e.e. percentage of the amino alcohol from the dimethoxyaryl-substituted aziridine than from the phenyl-substituted aziridine is consistent with ring opening by partial or full generation of a carbocation, as has previously been reported^29,31^, and greater stability of the carbocation derived from the dimethoxyaryl-substituted aziridine.

**Extended Data Fig. 4:**
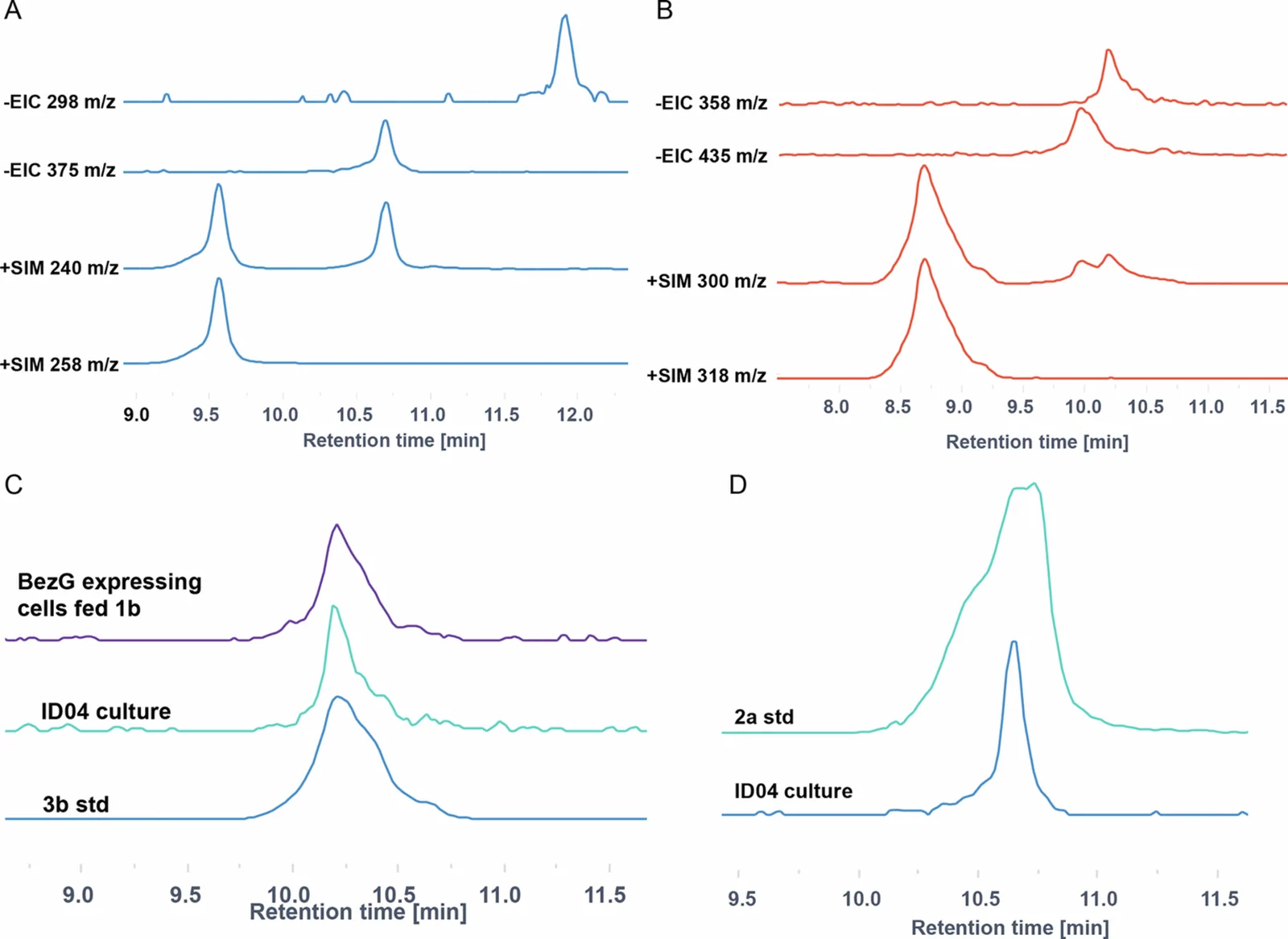
LC-MSD chromatograms of the products of nitrene transfer with styrene and dimethoxystyrene as substrates. (**a**) LC-MSD analysis of ID04 reactions with styrene (Sty). In negative mode, the acetylated amino alcohol **3a** (EIC *m/z* 298) and the PABA-opened product **2a** (EIC *m/z* 375) are detected. The amino alcohol **1a** (SIM *m/z* 258) and the diamine **2a** can also be observed as the benzylic carbocation fragment (SIM *m/z* 240) due to in-source fragmentation. (**b**) LC-MSD analysis of ID04 reactions with DMS. In negative mode, the acetylated amino alcohol **3b** (EIC *m/z* 358) and the PABA-opened product **2b** (EIC *m/z* 435) are detected at low levels. The amino alcohol **1b** (SIM m/z 318), as well as **3b** and **2b**, can also be detected as the benzylic carbocation (SIM *m/z* 300) arising from fragmentation. (**c**) Negative-mode LC-MSD chromatograms (EIC *m/z* 358) showing *N*-acetyl amino alcohol **3b** formation. Chromatograms from BL21(DE3) Star expressing pBezG and fed with **1b** show production of **3b**, indicating BezG-catalyzed acylation of the post-nitrene–transfer product. The retention time matches **3b** generated in full reactions with DMS and an authentic standard. (**d**) Negative-mode LC-MSD chromatograms (EIC *m/z* 375) showing formation of diamine **2a**. Diamine **2a** produced during ID04 reactions with styrene is shown alongside an authentic standard for comparison.

The greater production of the aziridine from DMS enabled the detection of two side products, diamine **2b** and *N*-acetyl amino alcohol **3b**. The reactions of styrene in ID04 formed the analogous diamine **2a** and *N*-acetyl amino alcohol **3a** (Fig. 3a and Extended Data Fig. 4). These compounds formed in smaller amounts from the biosynthetic process than did amino alcohols **1a** from styrene and **1b** from dimethoxystyrene; the combined peak areas of diamine **2a** and *N*-acetyl amino alcohol **3a** from styrene in the HPLC trace with ultraviolet–visible spectroscopy (UV–Vis) detection were less than 10% of that of amino alcohol **1a** (Extended Data Fig. 4a), and the peak areas of diamine **2b** and *N*-acetyl amino alcohol **3b** from dimethoxystyrene were less than 15% of that of amino alcohol **1b** (Extended Data Fig. 4b), depending on conditions. These compounds were identified by LC–MS in negative and positive ion mode, and the identities of diamine **2a** and *N*-acetyl amino alcohol **3b** were confirmed by independent synthesis (Extended Data Fig. 4c,d). *N*-acetyl amino alcohols **3a** and **3b** would form by acylation of amino alcohols **1a** and **1b** catalysed by BezG_niv_. This acylation of the amino alcohol was confirmed by feeding **1b** to cells expressing BezG_niv_ and observing the formation of **3b** (Extended Data Fig. 4c). While production of diamine **3b** is favoured at higher concentrations of PABA (Fig. 3e), the current data do not distinguish whether the diamines arise from aziridine opening by PABA or from an alternative radical diamination pathway.

### Site-directed mutagenesis of BezE_Ure_

**Extended Data Fig. 5:**
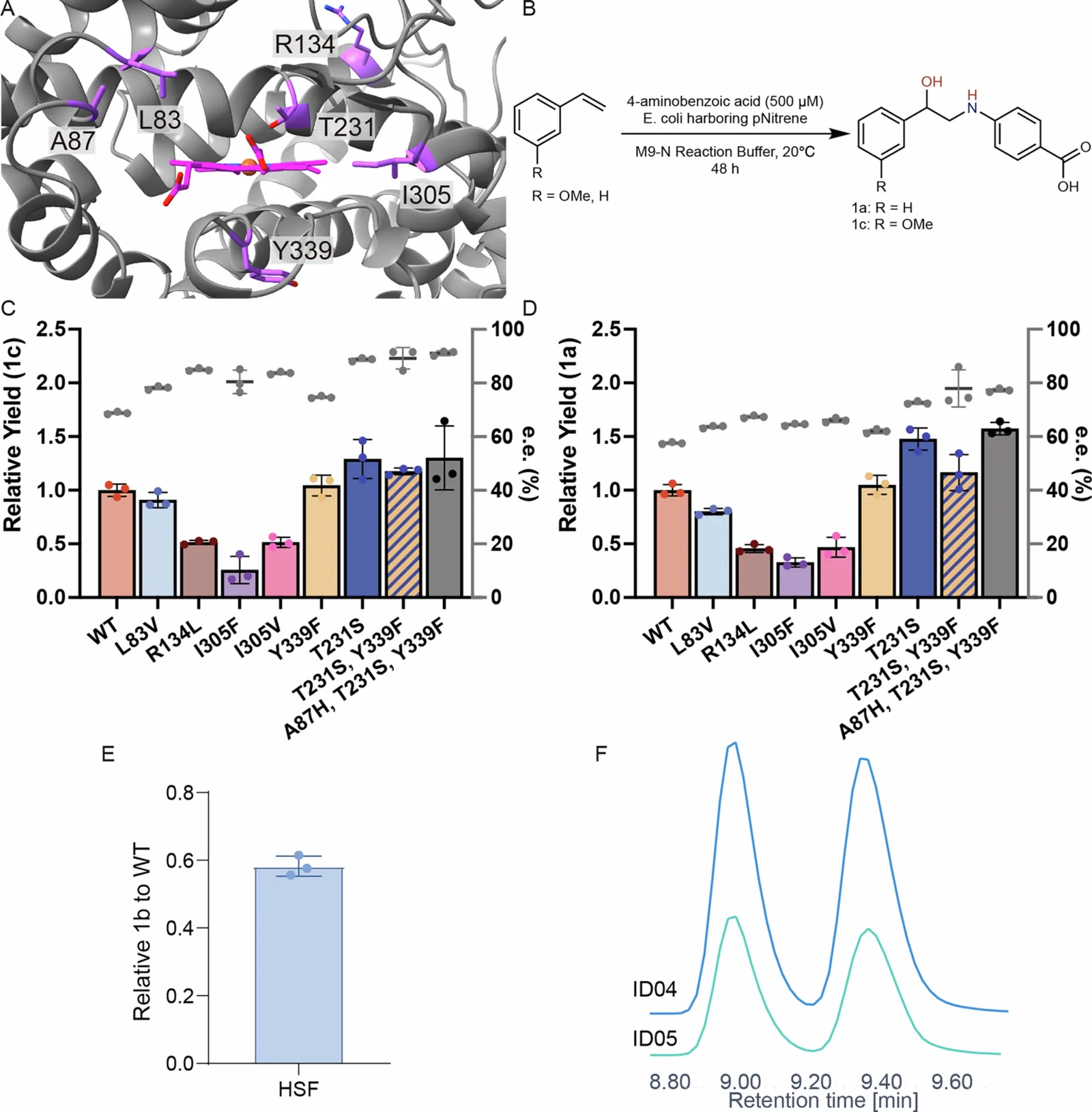
Directed evolution of BezE_Ure_. (**a**) Amino acid positions varied during site-directed mutagenesis of BezE_Ure_. Targeted residues are highlighted and labeled. (**b**) Model nitrene transfer reaction on styrene or 3-methoxystyrene to form amino alcohols **1a** or **1c**, respectively. (**c**) Two-axis plot of yield (%) and *ee* (%) of amino alcohol **1c** produced by mutants of BezE_Ure_. Colored bar graph represents yield of **1c**. The grey bars represent *ee* of **1c**. (**d**) Two-axis plot of yield (%) and *ee* (%) of amino alcohol **1a** produced by mutants of BezE_Ure_. Colored bar graph represents yield of **1a**. The grey bars represent *ee* of **1a**. (**e**) Relative production of **1b** for HSF compared to wild type (WT). (**f**) LC-MSD (SIM, 318 *m/z*) of ID04(WT) produced **1b** and of **1b** produced by ID05 (HSF) demonstrating unchanged *ee*. Data are mean ± s.d. *n*=3 biological replicates. Source data

We sought to improve the enantioselectivity of this aziridination reaction and, ultimately, to reveal the factors that influence the final e.e. of the ring-opened products. Five amino acid sites on BezE_Ure_ were chosen because of their proximity to the modelled active site (Extended Data Fig. 5a). To accentuate the effect of the active site modifications on the reactivity and stereoselectivity of nitrene transfer, 3-methoxystyrene was chosen as the model substrate (Extended Data Fig. 5b) for screening, owing to its enhanced solubility and the relatively small magnitude of the *m*-OMe Hammett parameter^41^.

**Fig. 4:**
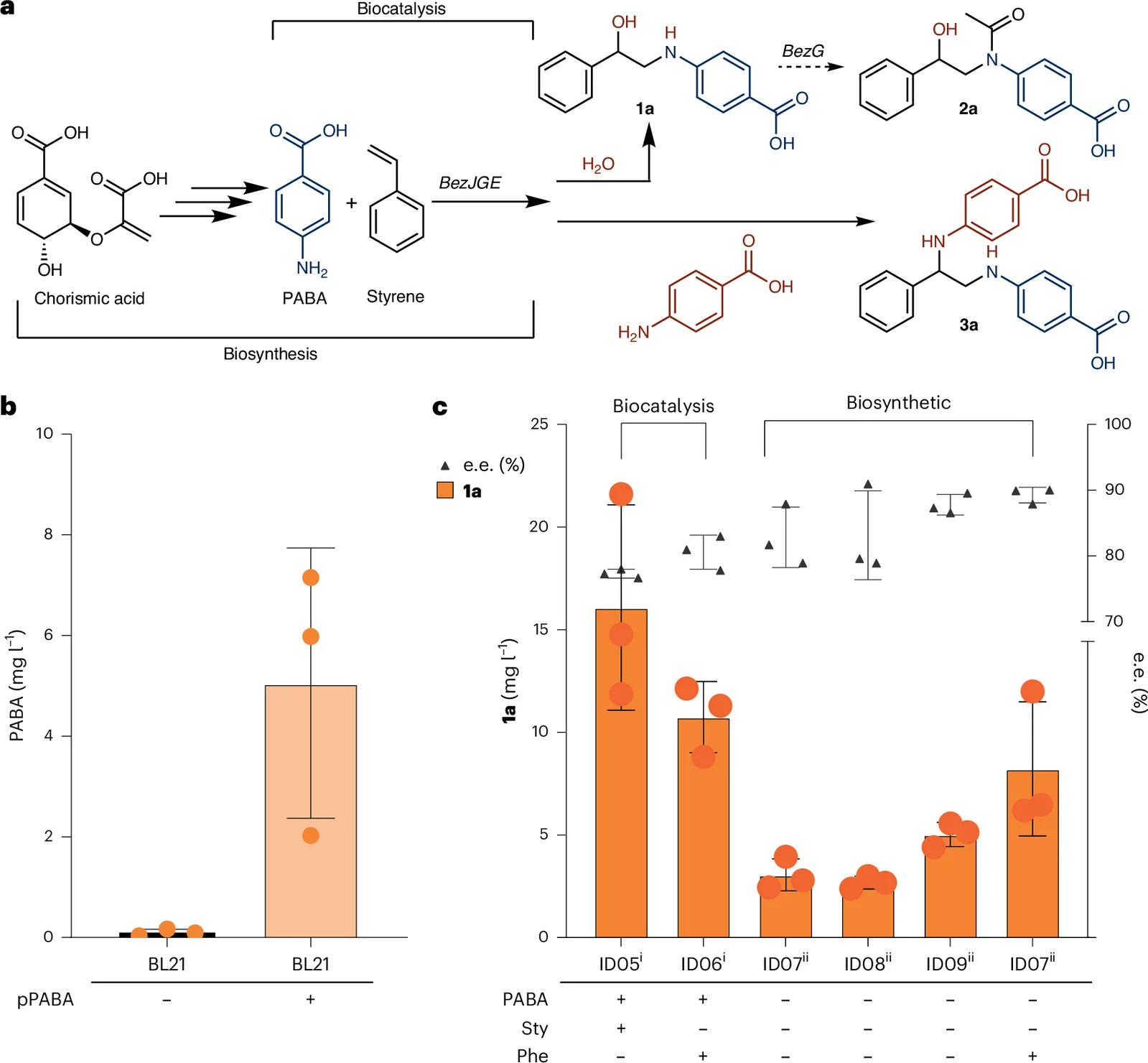
Incorporation of nitrene transfer reactions into metabolism and production of compounds in minimal media. **a**, Scheme for incorporation of the nitrene transfer reaction into metabolic pathways. **b**, Enhanced BL21 DE3 Star PABA titres in M9-HG on inclusion of the pPABA construct. **c**, Titres of **1a** produced by both biocatalysis and biosynthesis. (i) Cells were grown and induced in Hyper Broth, then resuspended in M9-HG and substrates added. (ii) Cells were grown and induced in M9-HGFe. Substrate concentrations are as follows: PABA: 500uM, Styrene: 40 mM, Phe: 8 mM. Data are mean ± s.d. *n* = 3 biological replicates. Source data

Three mutations (A87H, T231S and Y339F) increased the e.e. and yield of amino alcohol **1c** produced from biocatalytic reactions. The double mutant was less productive than was T231S but it provided the product amino alcohol **1a** with higher e.e. (Extended Data Fig. 5b,c), so this double mutant was subjected to one more round of screening. The second round of mutagenesis yielded the triple mutant A87H_T231S_Y339F (pJGEu(HSF)), strain ID05, which produced 30% more **1c** than did pJGEu and with a higher e.e. of 91%. These mutations also led to higher production of **1a** when styrene was used as the substrate. The production of **1a** was 1.6 times higher (16 mg l^−1^; Fig. 4c and Extended Data Fig. 5d) with ID05 than with ID04 containing WT BezE_Ure_ and occurred with an e.e. of 77%. However, the higher activity of this enzyme for production of **1a** and **1c** did not translate to the production of **1b**. The yield with HSF under biocatalytic conditions (with 2 mM 3-methoxystyrene and 500 μM PABA) was approximately 60%, and the e.e. was nearly identical to that of the product produced by the wild-type enzyme (Extended Data Fig. 5e,f).

### Biosynthetic production of unnatural, nitrogen-containing products

Next, we explored the in vivo production of vinylarenes that could react with the nitrene, thereby creating a fully biosynthetic pathway starting from simple sugars and minimal salts to form the corresponding aziridines and downstream ring-opened products (Fig. 4a). Production levels of styrene (via phenylanine^42,43^) and 4-VP (via tyrosine^44^) were compatible with those from overproduction of PABA because all of these intermediates share chorismate as a precursor^45^. A plasmid (pSTY; see Supplementary Table 1 for details) was built for production of styrene, and transformation of this plasmid into ID05 created strain ID06 (harbouring plasmids pJGEu(HSF) and pSTY). Biocatalytic reactions conducted with cultures of ID06 supplemented with PABA and 8 mM Phe produced 10.7 mg l^−1^ of amino alcohol **1a** (Fig. 4c).

To improve biosynthesis of the nitrene precursor, we introduced into ID06 an additional PABA production pathway (via pPABA (where p is *para*), which increased PABA production from 10 µg l^−1^ to 5 mg l^−1^ in minimal medium M9-HG when introduced into BL21 DE3 Star; Fig. 4b) creating ID07. This strain produced 3 mg l^−1^ of **1a** in glucose minimal medium (Fig. 4c). Diamine **2a** and acylated amino alcohol **3a** were not detected by LC–MS under fully biosynthetic conditions; only the amino alcohol (**1a**) was detected as a product from the reaction of the vinylarene in this cultivation. A Phe-overproducing strain of *E. coli* (NST74) transformed with pTrcHSF (pJGEu(HSF) with the Trc promoter in place of T7), pPABA and pSty (strain ID09) produced 5 mg l^−1^ **1a** in 87% e.e. (Fig. 4c), indicating that intracellular levels of Phe in ID07 limit production of **1a** (the same plasmids in BL21(ID08) produced only 2.7 mg l^−1^; Fig. 4c). A culture of ID07 supplemented with 8 mM Phe produced 8.2 mg l^−1^ of **1a** with 89% e.e. in minimal medium (Fig. 4c), confirming this hypothesis.

### Discovery of unusual enzyme-catalysed nitrene-transfer reactions

**Fig. 5:**
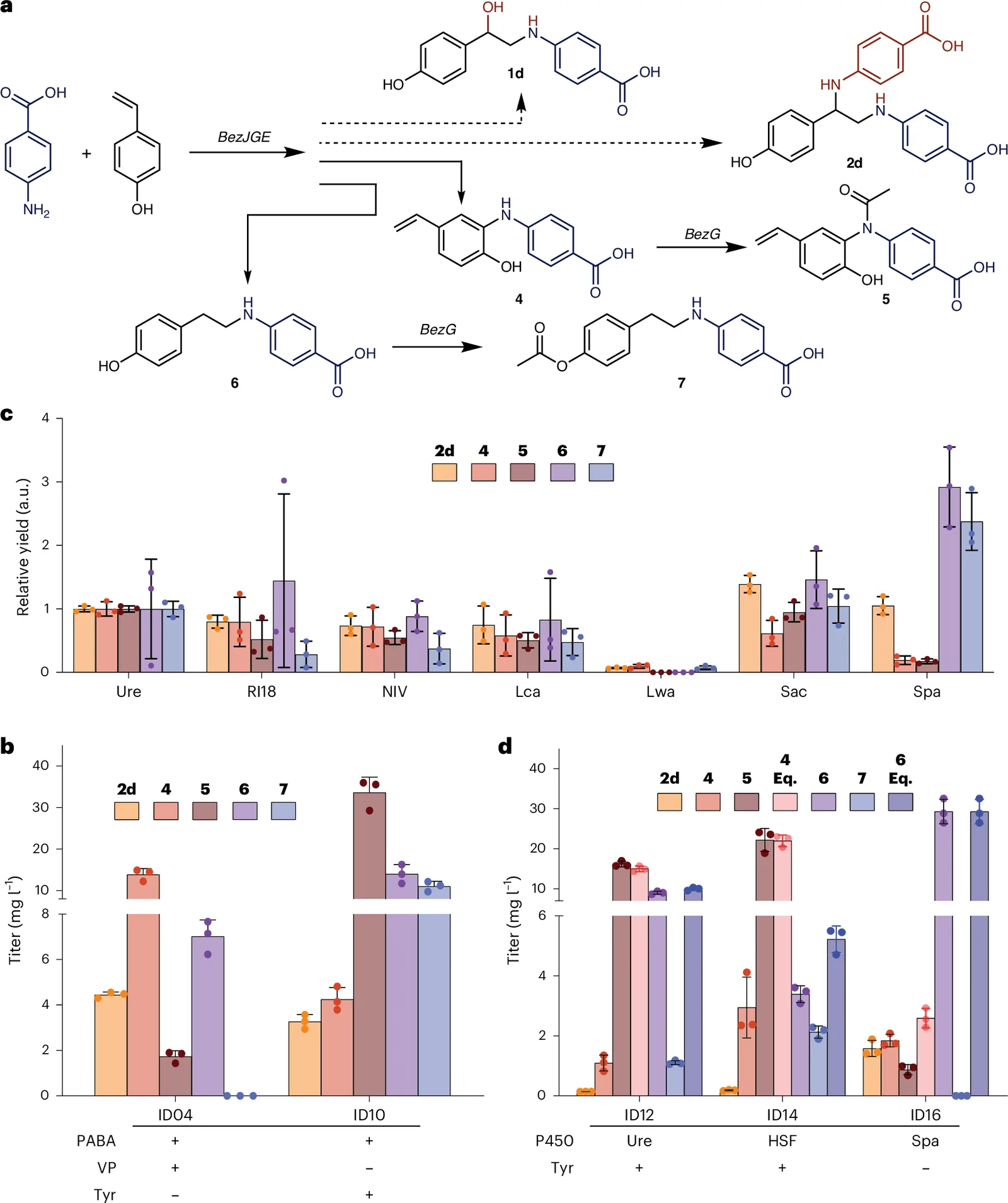
Production of vinylphenol bioproducts. **a**, Schema for the production of **2d**, **4**, **5**, **6** and **7** from PABA and 4-VP. **b**, Biocatalytic reactions of ID04 and ID10. Cells were induced in rich media, resuspended in M9 and substrates were added. Substrate concentrations are 500 μM PABA, 2 mM VP and 2 mM Tyr. **c**, Homologue screening of BezE. Supplementary Table 5 presents the accession numbers and sequence identity for all variants tested. All cells were cotransformed with pGnJn and each P450, which were expressed on the pEr backbone. **d**, Biosynthetic titres for the three P450s tested. Data are mean ± s.d. *n* = 3 biological replicates. **4** Eq. is the sum of the titres of **4** and **5** corrected for molecular weight. **6** Eq. is the sum of **5** and **6** corrected for molecular weight. Source data

**Extended Data Fig. 6:**
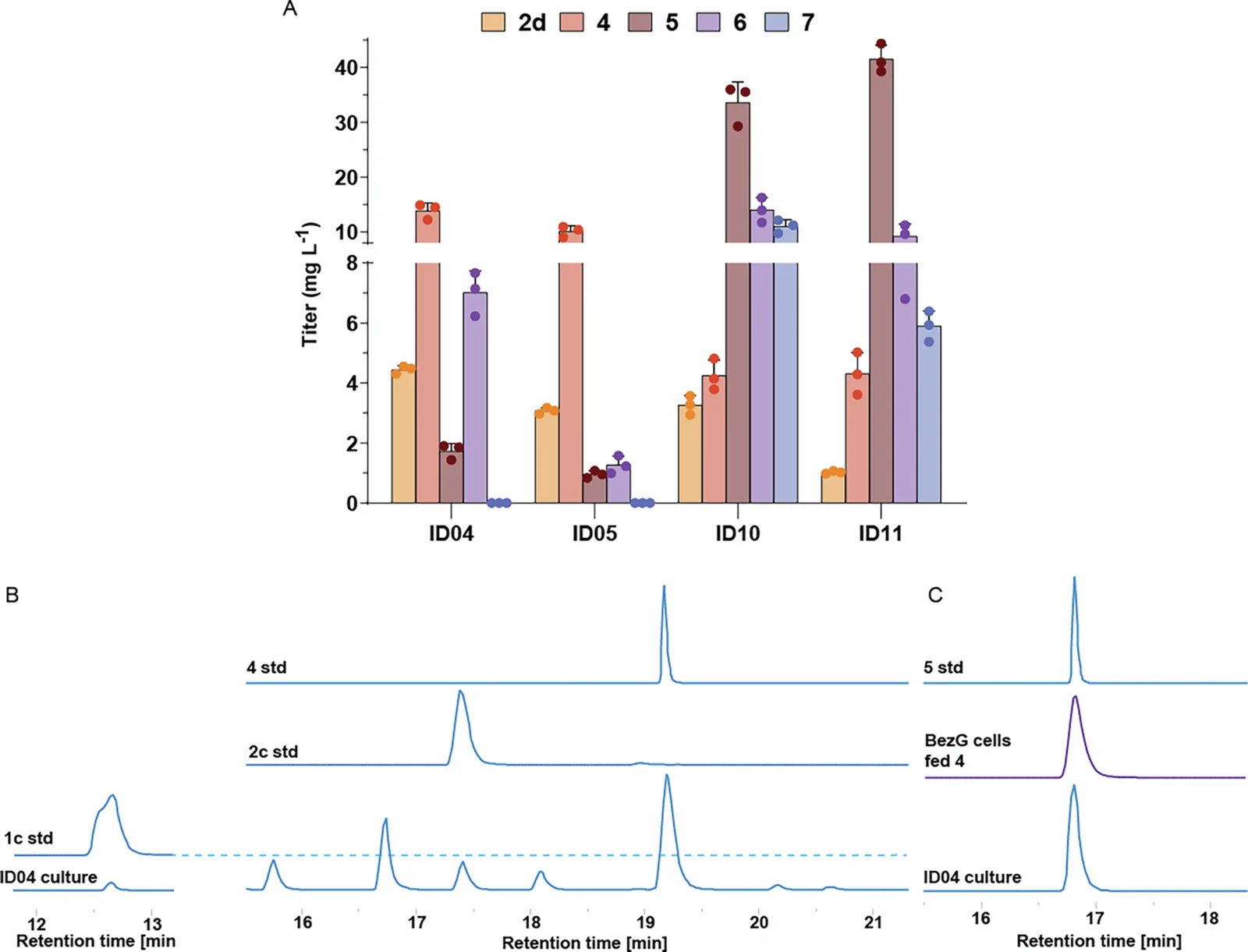
Analysis of biocatalytic reactions with vinyl phenol as substrate. (**a**) Production of **2d**, **4**, **5**, **6**, and **7** between ID04(WT) and ID05(HSF) and ID10(WT +pVP) and ID11(HSF+pVP) biocatalytic reactions. Data are mean ± s.d. *n*=3 biological replicates. (**b**) LC-MSD chromatograms (positive mode, SIM, 256 *m/z*) of the cation generated by fragmentation at the benzylic position of both amino alcohol **1c** and diamine **2d** demonstrates 4-VP reactions with ID04 negligibly produces the amino alcohol **1c**, whereas the diamine **2d** is generated, alongside diarylamine **4** (**c**) LC-MSD chromatograms (SIM, positive mode, 298 *m/z*) showing production of acylated diarylamine **5** by ID04 reactions with 4-VP. Source data

With DMS and styrene established as acceptable substrates for nitrene transfer, generating both amino alcohols and diamines, we tested whether the same classes of compounds could be produced from 4-vinylphenol (4-VP) because this vinylarene can be produced from tyrosine. However, addition of 4-VP and PABA exogenously to the strain ID05 formed several products resulting from initial reactivity of the *N*-acetoxyaniline (Fig. 5a). In contrast to reactions of the other two vinylarenes, the amino alcohol (**1d**; identity confirmed by independent synthesis) was a minor product formed in titres of just 0.5 mg l^−1^. Diamine **2d** formed in larger amounts than did the amino alcohol, with a titre of 3.1 mg l^−1^ determined by LC–MS in both positive and negative ionization mode and confirmed by independent synthesis (Extended Data Fig. 6a), but **2d** was also a minor product (Extended Data Fig. 6b). Instead, the major products, diarylamines **4** and **5** resulted from formal insertion of the nitrene into the aryl C–H bond *ortho* to the hydroxyl group. One of these two products was the free *ortho-*amino phenol (**4**), and the other product was the N-acetylated *ortho*-amino phenol (**5**), both with the vinyl group intact. The process also formed tyramine **6**. To test if the products formed from reaction of the P450, we added 4-VP and PABA exogenously to strain ID04 (wild-type BezE_Ure_). This procedure led to a higher production of most of the amines (Extended Data Fig. 6a). The cells produced 0.18 mg l^−1^ amino alcohol **1d**, 4.4 mg l^−1^ diamine **2d**, 13 mg l^−1^ diarylamine **4**, 1.7 mg l^−1^ of *N*-acetyl diarylamine **5** and 7 mg l^−1^ tyramine **6** (Fig. 5b).

To determine if the diarylamine and tyramine products also were generated when 4-VP was produced in vivo, we built a biosynthetic pathway to convert tyrosine^44^ to 4-VP on plasmid pVP and transformed this plasmid into the ID04 because this strain produced the highest titres of each biosynthetic product. The resulting strain, ID10, produced these same products (**4**, **5** and **6**) and a new compound, *O*-acetyl tyramine **7**, in addition to the amino alcohol **1d** and diamine **2d** (Fig. 5a), although the ratios of these products were different from those obtained when a culture of ID04 was supplemented with 4-VP instead of tyrosine. When the reaction medium was supplemented with PABA and tyrosine, ID10 produced 3.3 mg l^−1^ of diamine **2d** (Fig. 5b). When the medium was supplemented with only tyrosine, ID10 produced approximately the same amounts of amino alcohol **1d** (35 μg l^−1^), diamine **2d** (at an unchanged 3.3 mg l^−1^) and diarylamines **4** and **5** (4.2 mg l^−1^ and 33.6 mg l^−1^), but ID10 produced substantially more of tyramines **6** and **7** (14 mg l^−1^ and 11 mg l^−1^, respectively). This result suggests that the product distribution depends on cellular conditions (Fig. 5b), as HSF coupled with pVP (ID11) led to a similar increase in tyramine production (Extended Data Fig. 6a).

Compounds **4** and **5** formed from the reactions of 4-VP were identified by NMR spectroscopy and LC–MS after isolation from 5 L cultures of ID09 and by independent synthesis. The N-acetylated *ortho*-amino phenol **5** results from the combination of formal nitrene insertion into the C–H bond at the 2-position of 4-VP and *N-*acylation of the resulting diarylamine. This product was identified by NMR spectroscopy of material obtained from the cultures of ID09. Independent synthesis of this compound and the non-acetylated diarylamine **4** confirmed their assignments, and feeding **4** to cells expressing BezG_niv_ verified its intermediacy in the production of **5** (Extended Data Fig. 6c).

**Extended Data Fig. 7:**
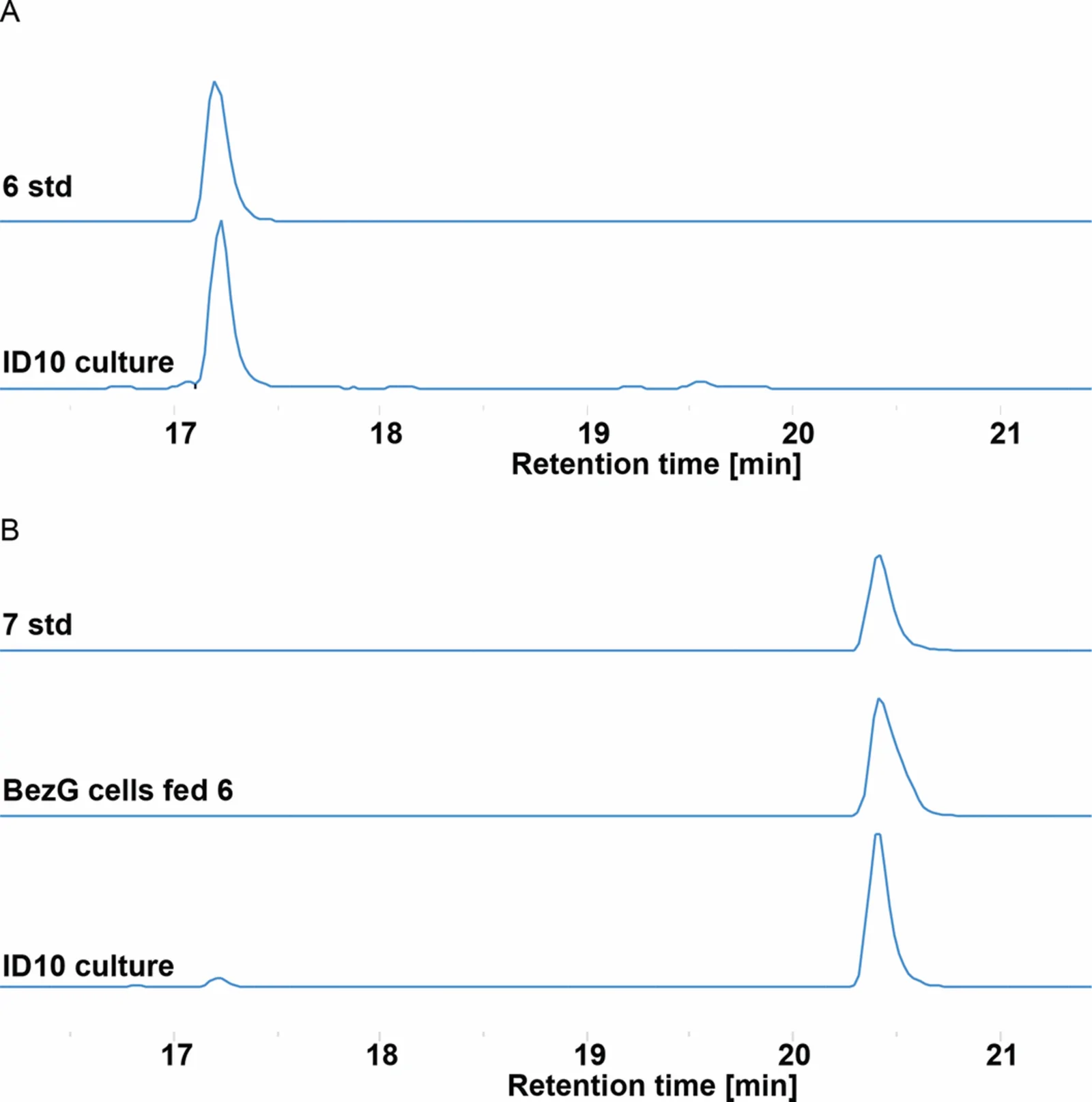
In vivo conversion of product 6 to product 7. (**a**) LC-MSD chromatograms (positive mode, EIC, 258 *m/z*) show production of tyramine **6** in ID10 cultures compared to authentic standard. (**b**) LC-MSD chromatograms (positive mode, EIC, 300 *m/z*) showing production of *O*-acyl tyramine **7** upon feeding of tyramine **6** to BL21 DE3 Star with pBezG and from ID10 cultures, thus confirming BezG’s role in generation of **7**.

*N-*aryl tyramine **6**, constituting a formal anti-Markovnikov hydroamination of the vinylarene, was also isolated from the culture and prepared independently (Extended Data Fig. 7a). Hydroamination via nitrogen radicals by enzymes has been reported^46,47^, but such reactions required photoexcitation. BezE catalyses this reaction innately. Compound **7** was determined by independent synthesis to be the *O*-acetyl tyramine derivative, resulting from an unusual regioselectivity of BezG_niv_ for acylation of the oxygen over the nitrogen of the tyramine. The selectivity of this acylation was confirmed by feeding tyramine **6** to cells expressing BezG_niv_ (Extended Data Fig. 7b). Given that tyrosine has been reported to undergo the addition of carbon radicals or oxidative coupling of nitrogen radicals at the *ortho* position^48–50^ and that we observe the product from hydroamination of this vinyarene by a pathway likely to involve the addition of a nitrogen radical to the vinyl group, we suggest that the hydroxyl group on 4-VP enables these reactions. Neither aryl C–H amination nor hydroamination has been reported to be catalysed by P450s, showing the unique capabilities of this combination of P450s and biologically produced nitrene.

**Extended Data Fig. 8:**
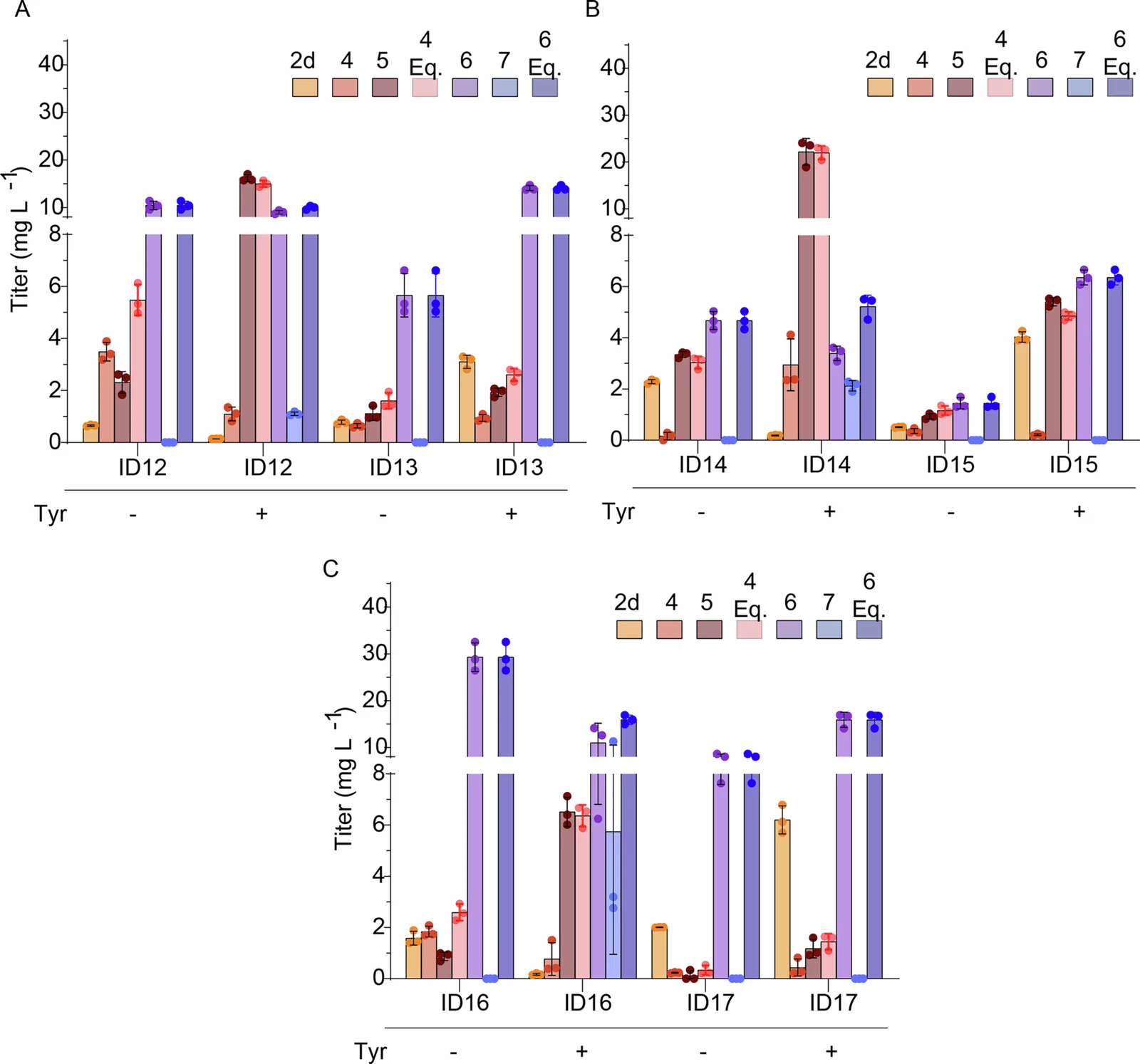
Effect of tyrosine supplementation on biosynthetic titers. (**a**) Biosynthetic titers for ID11 and ID12 with and without tyrosine supplementation. (**b**) Biosynthetic titers for ID13 and ID14 with and without tyrosine supplementation. (**c**) Biosynthetic titers for ID15 and ID16 with and without tyrosine supplementation. All cells were grown and induced in M9-HGFe and sampled after 6 days of growth. Data are mean ± s.d. *n*=3 biological replicates. Source data

To determine the distribution of these five main products under fully biosynthetic conditions, we built ID11 by transforming the PABA biosynthetic pathway on pPABA into ID10 and cultured this strain in M9 minimal medium with only glucose as a carbon source. Given the decreased volatility of vinylphenol and the effect of pVP coexpression on the biocatalytic reactions, we tested four combinations of strains and conditions: ID12 in minimal medium, ID12 in minimal medium supplemented with tyrosine, ID13 (harbouring pUVP, which has the weaker UV5 promoter in place of the stronger Trc promoter on pVP) in minimal medium and ID13 in minimal medium with tyrosine supplementation. ID12 produced 0.7 mg l^−1^ diamine **2d**, 3.5 mg l^−1^ diarylamine **4**, 1.8 mg l^−1^ acylated diarylamine **5** and 10.4 mg l^−1^ tyramine **6** (Fig. 5d and Extended Data Fig. 8a). Neither the amino alcohol **1d** nor *O*-acyl tyramine **7** was detected. Supplementation of the medium with tyrosine increased the titres of *N*-acetyl diarylamine **5** and *O*-acetyl tyramine **7** to 16 mg l^−1^ and 1.1 mg l^−1^, respectively, while decreasing the titres of diamine **2d**, diarylamine **4** and tyramine **6** to 0.14 mg l^−1^, 1.1 mg l^−1^ and 9 mg l^−1^, respectively. Growth of ID13 with and without added tyrosine produced tyramine **6** with higher selectivity than did ID12, and ID13 supplemented with tyrosine produced more of tyramine **6** (14 mg l^−1^, **7** was not detected) than did ID12 (Extended Data Fig. 8a). The additional strains ID14 (which harbours pJGEu(HSF), pPABA and pVP) and ID15 (which harbours pJGEu(HSF), pPABA and pUVP) were subjected to the same two conditions. ID14 cultures supplemented with tyrosine produced 2.9 mg l^−1^ diarylamine **4**, 22 mg l^−1^ *N*-acetyl diarylamine **5** and 5.2 mg l^−1^ tyramine **6** equivalents (consisting of 3.4 mg l^−1^ **6** and 2.2 mg l^−1^ **7**, after correcting for the molecular weight (MW) of the products; 6 Eq., Fig. 5d, and Extended Data Fig. 8b). As **5** results from acetylation of **4**, the 2.9 mg l^−1^ of diarylamine **4** and 22 mg l^−1^ of *N*-acetyl diarylamine **5** (19 mg equivalents of **4**) indicates that 22 mg l^−1^ of **4** equivalents (Fig. 5d, 4 Eq., and Extended Data Fig. 8b; after correcting for the MW of the products) are produced by the generation of *N*-acetoxyamine and formal transfer of nitrene into the C–H bond.

Given the higher selectivity for formal insertion of the nitrene unit into the aryl C–H bond by the HSF mutant, we conducted nitrene transfer to 4-VP with all prior and several additional homologues of BezE to identify variants with altered selectivity (Fig. 5c and Supplementary Table 5). The homologue from *Salinispora pacifica*, BezE_Spa_, produced almost exclusively the tyramines from 4-VP. The production of tyramine **6** was 2.9 times higher and the production of acylated tyramine **7** was 2.3 times higher than that with BezE_Ure_, and the production of diamine **4** and N*-*acetylated diamine **5** was just 0.12 and 0.17 of that with BezE_Ure_. To test the product distribution with BezE_Spa_ in the biosynthetic sequence, we placed its gene into the JGE_Spa_ operon and constructed strains ID16 (pJGE_Spa_, pPABA and pVP) and ID17 (pJGE_Spa_, pPABA and pUVP). ID16 produced 29 mg l^−1^ tyramine **6**, but no *O-*acetyl tyramine **7** was detected without tyrosine supplementation. Supplementation with tyrosine reduced production of **6** to 11 mg l^−1^ and increased production of **7** to 5.7 mg l^−1^ (which corresponds to 16 mg l^−1^ of **6** produced by acetoxyamine generation and reactivity) (Fig. 5d and Extended Data Fig. 8c).

## Discussion

Independent chemical synthesis of the reaction products formed by these various biosynthetic pathways illuminated the distinctions between artificial biosynthesis and chemical synthesis to obtain identical reaction products. The biosynthetic approach permits synthesis to begin from simple sugars, salts and other common media components, relying entirely on the enzymatic machinery present in bacteria to produce compounds containing free aromatic and aliphatic alcohols, free amines, free carboxylic acids and acyl groups installed selectively at nitrogen and oxygen. Our independent chemical synthesis of these same structures, even in racemic form, with their polar functional groups required complex and low-yielding routes tailored for each of the products because we were unable to generate them via abiological nitrene transfer reactions. These routes comprised more costly starting materials than the biosynthesis, a series of protections and deprotections and, in several cases, reactions that formed complex mixtures from low chemo- and regioselectivity and required complex and costly purification of products obtained in low yield.

In summary, our work shows that integrating a series of natural and artificial biosynthetic steps can create pathways comprising the biosynthesis of nitrene precursors from the common metabolite PABA and subsequent intermolecular transfer of a nitrene unit to a series of vinylarenes generated from amino acids. These transfers are catalysed by P450 BezE_Ure_ and homologues that constitute a class of nitrene transferases, which enable the production of diverse nitrogen-containing compounds previously inaccessible to biology. These biosyntheses were engineered in a common *E. coli* BL21 DE3 strain from simple sugars in minimal medium, and the titres and selectivities of the products were increased by metabolic and protein engineering, making future scale-up to large-volume bioreactors similar to that of other processes involving P450 enzymes. If styrene^51^ and vinylphenol^52^ were added to the medium, their toxicities would need to be ameliorated, probably through adaptive laboratory evolution. However, in vivo production of styrene or vinylphenol from glucose can avoid the toxicity of exogenous addition if the production and consumption pathways are balanced through metabolic engineering to minimize their accumulation. The unusual reactivities demonstrated by BezE and its homologues should be valuable starting points for future engineering of enzymatic amination and aziridination reactions, mechanistic studies of biological nitrene transfers and identification of additional biosynthetic gene clusters leading to nitrene transfer and their resulting natural and unnatural products. Because the acetoxyamine components of the aziridination and amination reactions stem from universal metabolic intermediates and because we have revealed robust nitrene transferases, these pathways could be incorporated into additional microbial hosts to expand the available substrates that can be modified by nitrenes generated from arylamines.

## Methods

### Chemical standards

All chemicals were obtained from standard suppliers (Millipore-Sigma, Alfa-Aesar, ThermoFisher, and so on) and used without purification. Chemically synthesized standards have a purity of 95% or higher. Methods for chemical standard synthesis and isolation of compounds from culture are provided in the Supplementary Information.

### Media preparation

Preparation of M9-High Glucose (M9-HG): 200 ml 5× M9 salts solution, 2 ml MgSO4 (1 M stock), 100 µl CaCl_2_ (1 M stock) and 15 g l^−1^ glucose were mixed and diluted to 1 L with ddH_2_O before filter sterilization. A 1,000× stock solution of trace metals (Teknova) was added to media aliquots (for 1× final concentration) directly before use to avoid precipitation. For biosynthesis reactions, there was additional iron supplementation (1 mM) using a 1 M stock of (NH_4_)_2_Fe(SO_4_)_2_ that was freshly prepared.

For preparation of M9-Autoinduction (M9-AI): 200 ml 5× M9 salts solution, 2 ml MgSO_4_ (1 M), 100 µl CaCl_2_ (1 M), 10 ml glycerol, 4 g lactose and 0.2 g glucose were mixed and diluted to 1 L with ddH_2_O before filter sterilization. A 1,000× stock solution of trace metals (Teknova) was added to media aliquots (for 1× final concentration) directly before use to avoid precipitation.

Hyper Broth was prepared following manufacturer (AthenaES) instructions.

Luria–Burtani broth and Terrific Broth were ordered as granules and were added as indicated to ddH_2_O before sterilization by autoclaving.

### In vitro nitrene transfer

BezG and BezE were expressed and purified as previously described^17^. The reaction mixture (100 µl) contained 1 µM BezG, 1 μM BezE, 200 µM *N*-OH-PABA, 200 µM AcCoA, 1 mM MgCl_2_ and 50 mM Tris–HCl buffer (pH 7.5). For reactions catalysed by free haem, the 1 µM BezE was replaced by 10 µM haem. The organic materials were diluted from a stock solution by a factor of 100× in 50% dimethyl sulfoxide (DMSO) v/v, and 1 µl of the diluted solution was added to the reaction. The final concentration of styrene was 8 mM and the final concentration of DMS was 2 mM. Reactions were incubated at 30 °C for 20 h, then quenched with 66 µl acetonitrile. Reactions were spun down in a microcentrifuge at the maximum speed for 5 min and 150 µl supernatant was removed for LC–MS analysis.

### Biocatalytic nitrene transfer reactions

Plasmids containing the genes encoding the desired enzymes were transformed into chemically competent *E. coli* NST74 (ATCC 31884) or BL21 Star DE3 (Invitrogen). For experiments with pJGEu and pSty/pVP, both plasmids were transformed simultaneously. For single enzyme expression reactions, we used the same vector as that used for purification. Selection plates were incubated at 30 °C overnight. Colonies chosen at random were inoculated into 3 ml Luria-Bertani broth medium supplemented with 0.4% glucose, added after autoclaving (LBglu), containing 25 µg ml^−1^ kanamycin, 100 µg ml^−1^ spectinomycin and/or 100 µg ml^−1^ carbenicillin depending on the plasmids used, and grown at 30 °C with shaking (220 rpm) overnight. Cells were diluted 1:25 into Hyper Broth (Athena Enzyme Systems) media containing the appropriate antibiotics and grown at 37 °C until the OD at 600 nm (OD_600_) reached 1.0, approximately 2 h of growth. The cultures were then cooled on ice for 30 min. IPTG was added to a final concentration of 0.5 mM. The cultures were grown at 20 °C with 220 rpm shaking for 24 h. Cells were spun down at 3,600*g* for 20 min, before resuspension to an OD_600_ of 30 in M9-HG. For supplementation of PABA (1,000× stock in DMSO), Phe (added as a powder) or Tyr (added as a powder), the compounds were added to the M9-HG before resuspension, resulting in the specified final concentration. The vinylarenes DMS, 3-vinylanisole and 4-hydroxy styrene (all at 2 mM final concentration) and styrene (40 mM final concentration) were added exogenously from a 100× stock solution in DMSO, resulting in the specified final concentrations. Following resuspension in M9-HG, cells were added to a 96-deep-well plate for a final reaction volume of 300 μl. The plate was sealed with a ThermoFisher Adhesive PCR Plate Foil and shaken at 220 rpm and 20 °C for 48 h. The reaction was quenched by adding 200 µl acetonitrile containing 0.2% formic acid v/v, vortexed at 1,000 rpm for 10 min, centrifuged for 20 min at 4,450*g*, supernatant pulled off and stored at −80 °C if not analysed immediately. Before analysis, the samples were thawed, briefly vortexed and centrifuged again at 4,450*g* for 20 min. 250 µl of the resulting solution was sampled and subjected to LC–MS analysis. For production of molecules for later analysis, cells were grown and induced in 15 L Terrific Broth, spun down and then resuspended in 5 L M9-HG media supplemented with 2 mM Tyr, and 500 µM PABA was split into six cultures of 500 ml and grown at 20 °C and 170 rpm for 3 days before extraction of compounds.

### Biosynthetic nitrene transfer reactions

Plasmids containing the genes encoding the desired enzymes were transformed into the appropriate chemically competent strain, NST74 (ATCC 31884) or BL21 Star DE3 (InVitrogen). Cells were first transformed with PABA, grown overnight in Luria–Bertani broth supplemented with 0.4% glucose (LBglu) with 100 µg ml^−1^ carbenicillin, and fresh competent cells were made before a second and double transformation with the appropriate nitrene and vinylarene pathway plasmids, before plating on Luria–Bertani (LB) broth agar plates with 50 µg ml^−1^ kanamycin, 100 µg ml^−1^ spectinomycin and 100 µg ml^−1^ carbenicillin. Selection plates were incubated at 30 °C overnight. Colonies chosen at random were inoculated into 3 ml LBglu containing 25 µg ml^−1^ kanamycin, 100 µg ml^−1^ spectinomycin and 100 µg ml^−1^ carbenicillin and grown at 30 °C with shaking (220 rpm) overnight. Cells were diluted 1:25 into M9-HGfe (M9-HG supplemented with 1 mM (NH_4_)_2_Fe(SO_4_)_2_ added from a 1,000× freshly prepared stock solution) with the appropriate antibiotics and grown at 37 °C until the OD_600_ reached approximately 1.0 (approximately 3 h). Reactions that produced the amino alcohol from styrene were sealed with parafilm to mitigate evaporation of styrene. Cells were induced with 500 µM IPTG, then cooled to and grown at 20 °C with 220 rpm shaking for 6 days. Aliquots of 300 µL were taken and added to 200 µl acetonitrile containing 0.2% formic acid. The resulting solution was vortexed at 1,000 rpm for 10 min and centrifuged for 20 min at 4,450*g*. The supernatant was aspirated by pipetting and stored at −80 °C before analysis. The standard volume used was 5 ml of M9-HG for non-production runs.

### Strains used and plasmid construction

The *E. coli* strains used for expression were NST74 (ATCC 31884) or BL21 Star DE3 (InVitrogen). Commercial strains were purchased as chemically competent cells. Strains from JBEI’s strain registry were made competent using standard methods^53^.

All plasmids were constructed using Golden Gate Cloning; gene blocks were ordered with bordering cutsites^54^. The DNA products were transformed into *E. coli* XL1-Blue competent cells (obtained from UC Berkeley’s QB3) and plated onto LB agar plates with the corresponding antibiotics (100 µg ml^−1^ spectinomycin, 50 µg ml^−1^ kanamycin or 100 µg ml^−1^ carbenicillin). Genes were ordered after codon optimization for use in *E. coli* from IDT, and pabC was amplified from the genome of BL21 Star DE3. Plasmids were mutagenized using a modified QuikChange method^55^.

All new plasmids and strains constructed for this study have been deposited in JBEI-ICE (https://public-registry.jbei.org).

### Library construction and screening

The JGEu or pSF plasmid was subjected to site saturation mutagenesis using a modified QuikChange method^55^. Degeneracy was introduced at the desired codons using NDT nucleotides for reduced library size. Round one sites tested were L83, R124, T231, I305 and Y339. Round 2 sites were A87 and V233. PCRs were verified by gel electrophoresis, before overnight DpnI treatment. A total of 5 μL digested PCR were transformed into 50 μl competent XL1-Blue cells. Following outgrowth in 1 ml SOC medium, 100 μl of cells, (1:10 of the outgrowth volume) were plated on an LB agar plate with kanamycin. The remaining culture was spun down, resuspended and added directly to LBglu with kanamycin. The next day, if the plate contained a minimum of 12 colonies, to ensure an approximate 10× coverage at the codon in the seed for the overnight culture, the overnight was miniprepped and transformed into *E. coli* BL21 DE3 Star. From the resulting plate, 36 colonies were chosen for 95% likely library coverage. Cells were picked into a 96-deep-well plate and grown at 30 °C with shaking (220 rpm). The following day 50 μl of overnight culture was added to 850 μl of HB in a 96-deep-well plate, and 50 μl was added to 50 μl of 50% glycerol (v/v) for storage at −80 °C. Cells were grown at 37 °C with shaking (220 rpm) for 2 h and were then induced by addition of 100 μl HB with 5 mM IPTG for a 500-μM final concentration. Cells were then grown at 20 °C with shaking at 220 rpm for 24 h. Cells were spun down at 3,600*g* for 20 min, before resuspension in 300 μl M9-HG containing 500 μM PABA and 2 mM 3-vinylanisole. The plate was sealed with a ThermoFisher Adhesive PCR Plate Foil and shaken at 220 rpm and 20 °C for 48 h. The reaction was quenched by adding 200 µl acetonitrile containing 0.2% formic acid v/v, vortexed at 1,000 rpm for 10 min, centrifuged for 20 min at 4,450*g*, supernatant pulled off and stored at −80 °C if not analysed immediately. Before analysis, the samples were thawed, briefly vortexed and centrifuged again at 4,450*g* for 20 min. A total of 250 μl of the resulting solution was sampled and subjected to LC–MS analysis. Wells with the desired activity were then sampled from the associated glycerol stock plate, and the miniprepped plasmid was sent for sequencing. The sequence verified plasmids were then transformed into *E. coli* BL21 DE3 Star and subjected to the standard biocatalytic nitrene transfer reaction protocol (growth and induction in HB, resuspension in M9-HG with added substrates) for validation of the observed activity.

### LC–MS analyses

Products were analysed by high-performance LC–MS using the Agilent 1260 infinity II system with mass selective detector XT (MSD XT). The HPLC autosampler and column compartment were maintained at 10 °C and 25 °C, respectively. Reactions with DMS and styrene were analysed with a Macherey–Nagel Sphinx RP HPLC column (5 μm, 15 cm × 4.6 mm). The mobile phase comprised 0.1% formic acid (v/v) in water (solvent A) and 0.1% (v/v) formic acid in acetonitrile (solvent B), at 25 °C. A flow rate of 1 ml min^−1^ was used. We used a gradient with an initial 98:2 ratio of A:B decreasing to 0:100 A:B over 25 min, holding constant at 0:100 for 2 min and then flushed at 98:2 for 7 min to re-equilibrate. Reactions with vinylphenol were analysed on a Macherey–Nagel NUCLEOSHELL PFP RP HPLC column (2.7 μm, 15 cm × 3 mm) with a column temperature of 40 °C. The mobile phase comprised 0.1% formic acid (v/v) in water (solvent A) and 0.1% (v/v) formic acid in acetonitrile (solvent B). A flow rate of 0.5 ml min^−1^ was used. We used a gradient with an initial 98:2 A:B decreasing to 30:70 A:B over 25 min, held at 0:100 with a flow rate of 1 ml min^−1^ for 2 min and then flushed at 98:2 for 8 min to re-equilibrate. Chiral LC was performed using a Phenomex Lux i-Amylose-3 column (5 μm, 15 cm × 4.6 mm). For analysis of e.e. of amino alcohols, the same gradient and mobile phase were used as were used for separation of achiral products. The e.e. of compound **2d** was determined with the same mobile phase with a modified gradient and with a column temperature of 40 °C. We used a flow rate of 0.5 ml min^−1^ with an initial ratio of 98:2 A:B decreasing to 0:100 A:B over 45 min, holding at 0:100 with a 1-ml min^−1^ flow rate for 2 min, then flushing at 98:2 with a flow rate of 0.5 ml min^−1^ for 8 min to re-equilibrate. Electrospray ionization MS was performed in positive-ion mode with a capillary voltage of 3,000 V, for [M + H]^+^ ions. The temperature of the drying gas and the flow rate were maintained at 350 °C and 12 l min^−1^, respectively, and the nebulizer gas was set to 35 lb in^−2^ (1 pound per square inch is equivalent to 6.894757 kPa). Chemical standards were diluted in M9-HG:ACN (60:40) to mimic sample conditions and account for any solvent effects on retention time.

### Reporting summary

Further information on research design is available in the Nature Portfolio Reporting Summary linked to this article.

## Online content

Any methods, additional references, Nature Portfolio reporting summaries, source data, extended data, supplementary information, acknowledgements, peer review information; details of author contributions and competing interests; and statements of data and code availability are available at https://doi.org/10.1038/s41557-026-02224-4.

## Supplementary information

Supplementary Information Supplementary Materials and Methods, Text, Tables 1–6, Fig. 1 and References.

Reporting Summary

## Source data

Source Data Fig. 3 Source data for Fig. 3.

Source Data Fig. 4 Source data for Fig. 4.

Source Data Fig. 5 Source data for Fig. 5.

Source Data Extended Data Fig. 2 Source data for Extended Data Fig. 2.

Source Data Extended Data Fig. 3 Source data for Extended Data Fig. 3.

Source Data Extended Data Fig. 5 Source data for Extended Data Fig. 5.

Source Data Extended Data Fig. 6 Source data for Extended Data Fig. 6.

Source Data Extended Data Fig. 8 Source data for Extended Data Fig. 8.

## Data Availability

The data supporting the findings in this study are available within this Article and its Supplementary Information. Source data are provided with this paper and are also available via figshare at https://doi.org/10.6084/m9.figshare.31817437 (ref. ^56^).
