# Supplementary material for: Nitrene generation and transfer for unnatural biosynthesis in living cells
Source: Nat Chem. 2026 Aug 18;18(11):1925–33. doi: 10.1038/s41557-026-02224-4 (PMC13719522; doi:10.1038/s41557-026-02224-4)
Supplement: Supplementary file 1 [file 41557_2026_2224_MOESM1_ESM.pdf]

nature chemistry

Supplementary information https://doi.org/10.1038/s41557-026-02224-4

Nitrene generation and transfer for unnatural biosynthesis in living cells

In the format provided by the authors and unedited

Materials and Methods ***3***

Supplemental Text ***4***

Table 1. Plasmids used in this study **4**

Table 2. Strains used in this study **5**

Table 3. Compounds produced in nitrene transfer reactions **6**

Table 4. Amino acid sequences of Proteins used **11**

Table 5. P450 percent identity matrix **14**

Table 6. Representative Plasmid maps: **15**

Table 7. Primers used for BezE$_{Ure}$ Mutagenesis **18**

Supplementary Discussion: **19**

Experimental Procedures and Characterization of Authentic Standards **19**

General Procedure for the Isolation of Biosynthetic Products from Bulk Fermentation **19**

Isolation of 2d from Biosynthesis **20**

Isolation of 7 from Biosynthesis **20**

Isolation of 5 from Biosynthesis **20**

Synthesis of 2-bromo-1-phenylethan-1-one (S1) **21**

Synthesis of *tert*-butyl 4-((2-oxo-2-phenylethyl)amino)benzoate (S2) **21**

Synthesis of *tert*-butyl 4-((2-hydroxy-2-phenylethyl)amino)benzoate (S3) **22**

Synthesis of 4-((2-hydroxy-2-phenylethyl)amino)benzoic acid (1a) **23**

Synthesis of *tert*-butyl 4-(2-phenylaziridin-1-yl)benzoate (S4) **23**

Synthesis of di-*tert*-butyl 4,4'-((1-phenylethane-1,2-diyl)bis(azanediyl))dibenzoate (S5) **24**

Synthesis of 4,4'-((1-phenylethane-1,2-diyl)bis(azanediyl))dibenzoic acid (2a) **25**

Synthesis of 2-bromo-1-(3,4-dimethoxyphenyl)ethan-1-one (S6) **26**

Synthesis of *tert*-butyl 4-((2-(3,4-dimethoxyphenyl)-2-oxoethyl)amino)benzoate (S7) **26**

***Synthesis of tert*-butyl 4-((2-(3,4-dimethoxyphenyl)-2-hydroxyethyl)amino)benzoate (S8)** ... **27**

Synthesis of 4-((2-(3,4-dimethoxyphenyl)-2-hydroxyethyl)amino)benzoic acid (1b) **28**

Synthesis of *tert*-butyl 4-(*N*-(2-(3,4-dimethoxyphenyl)-2-oxoethyl)acetamido)benzoate (S9) **29**

Synthesis *of tert*-butyl 4-(*N*-(2-(3,4-dimethoxyphenyl)-2-hydroxyethyl)acetamido)benzoate (S10) **29**

Synthesis of 4-(*N*-(2-(3,4-dimethoxyphenyl)-2-hydroxyethyl)acetamido)benzoic acid (3b) .. **30**

Synthesis of 2-bromo-1-(3-methoxyphenyl)ethan-1-one (S11) **31**

Synthesis of *tert*-butyl 4-((2-(3-methoxyphenyl)-2-oxoethyl)amino)benzoate (S12) **32**

Synthesis of 4-((2-(3-methoxyphenyl)-2-oxoethyl)amino)benzoic acid (S13)........................32
Synthesis of 4-((2-hydroxy-2-(3-methoxyphenyl)ethyl)amino)benzoic acid (1c) 33
Synthesis of *tert*-butyl 4-((2-(4-hydroxyphenyl)-2-oxoethyl)amino)benzoatebenzoate (S14) 34
Synthesis of 4-((2-(4-hydroxyphenyl)-2-oxoethyl)amino)benzoic acid (S15)........................34
Synthesis of 4-((2-hydroxy-2-(4-hydroxyphenyl)ethyl)amino)benzoic acid (1d) 35
Synthesis of 4-azidobenzoic acid (S16) 36
Synthesis of 4,4'-((1-(4-hydroxyphenyl)ethane-1,2-diyl)bis(azanediyl))dibenzoic acid (2d) ..36
Synthesis of *tert*-butyl 4-iodobenzoate (S17) 37
Synthesis of methyl 4-hydroxy-3-nitrobenzoate (S18) 38
Synthesis of 2-hydroxy-5-(methoxycarbonyl)benzenaminium acetate (S19) 39
Synthesis of methyl 3-((4-(*tert*-butoxycarbonyl)phenyl)amino)-4-hydroxybenzoate (S20)....40
Synthesis of methyl 3-((4-(*tert*-butoxycarbonyl)phenyl)(*tert*-butyldimethylsilyl)amino)-4-((*tert*-butyldimethylsilyl)oxy)benzoate (S21)...41
Synthesis of *tert*-butyl 4-((2-((*tert*-butyldimethylsilyl)oxy)-5-(hydroxymethyl)phenyl)amino) benzoate (S22)..42
Synthesis of *tert*-butyl 4-((2-((*tert*-butyldimethylsilyl)oxy)-5-formylphenyl)amino)benzoate (S23)...43
Synthesis of 4-((5-formyl-2-hydroxyphenyl)amino)benzoic acid (S24) 44
Synthesis of 4-((2-hydroxy-5-vinylphenyl)amino)benzoic acid (4) 45
Synthesis of 4-(*N*-(2-hydroxy-5-vinylphenyl)acetamido)benzoic acid (5) 46
Synthesis of 4-((4-hydroxyphenethyl)amino)benzoic acid (6)...47
Synthesis of 4-((4-acetoxyphenethyl)amino)benzoic acid (7)...47
NMR Spectra..49
References *140*
Table of Contents

Materials and Methods

Unless stated otherwise, all chemicals, salts, and solvents were obtained from commercial suppliers (e.g. Sigma-Aldrich, Ambeed, Strem Chemicals) and used without further purification. All air- and moisture-sensitive manipulations were conducted using standard Schlenk techniques under an atmosphere of nitrogen. Solvents and solutions were transferred using air-tight syringes. All flame-dried vessels were placed under vacuum and externally heated using a propane flame. All oven-dried vessels were placed into a 160 °C drying oven for at least 24 h. All reactions were performed using Teflon-coated magnetic stirring bars unless otherwise stated. Reactions were monitored by thin layer chromatography (TLC) on Kieselgel 60 F254 glass plates precoated with 0.25 mm thickness of silica gel and visualized by UV irradiation. Flash column chromatography was conducted with a Teledyne Isco CombiFlash NEXTGEN 300+ System using Teledyne RediSep Gold High Performance 20–40 µm column for normal phase and Teledyne Redisep Gold Reverse-Phase C18 20–40 µm for reverse phase separations.

Nuclear magnetic resonance (NMR) spectra were acquired on a Bruker Avance I at 400 MHz for ^{1}H, a Bruker Avance IV NEO at 500 MHz for ^{1}H and 126 MHz for ^{13}C, a Bruker Avance III at 600 MHz for ^{1}H and 151 MHz for ^{13}C, or a Bruker Avance I at 700 MHz for ^{1}H at the Pines Magnetic Resonance Center at the College of Chemistry, University of California, Berkeley. Chemical shifts were reported in ppm downfield of TMS and were referenced to residual solvent signal (^{1}H-NMR: $CDCl_3$ δ = 7.76 ppm, CD_3OD = 3.31 ppm, $(CD_3)_2SO$ = 2.50 ppm, CD_3CN = 1.94 ppm; ^{13}C-NMR: $CDCl_3$ δ = 77.16 ppm, CD_3OD = 49.00 ppm, $(CD_3)_2SO$ = 39.52 ppm, CD_3CN = 1.32 ppm). NMR Spectra are reported as follows: chemical shift (multiplicity, coupling constants where applicable, number of nuclei). Splitting is reported with the following symbols: s = singlet, d = doublet, t = triplet, q = quartet, dd = doublet of doublets, dt = doublet of triplets, td = triplet of doublets, m = multiplet. Coupling constants are reported in Hz. High resolution mass spectrometry (HRMS) was performed on a Perkin Elmer axION ESI TOF at the LBNL Catalysis Laboratory, University of California, Berkeley.

Supplemental Text

Table 1. Plasmids used in this study

Accession Code	Plasmid Name	Description	Origin
JBx_266774	pGnAf	Ampicillin resistance, ColE1 origin, P_{Trc}:*bezG*,P_{Trc}:*aurF*	This Work
JBx_266776	pGnJr	Ampicillin resistance, ColE1 origin, P_{Trc}:*bezG*,P_{Trc}:*bezJr*	This Work
JBx_266775	pGnJn	Ampicillin resistance, ColE1 origin, P_{Trc}:*bezG*,P_{Trc}:*bezJn*	This Work
JBx_266770	pEr	Spectinomycin resistance, CDF origin, P_{T7}:$bezE_{RI18}$	This Work
JBx_278371	pE Niv	Spectinomycin resistance, CDF origin, P_{T7}:$bezE_{Niv}$	This Work
JBx_278369	pE Lca	Spectinomycin resistance, CDF origin, P_{T7}:$bezE_{Lca}$	This Work
JBx_278370	pE Lwa	Spectinomycin resistance, CDF origin, P_{T7}:$bezE_{Lwa}$	This Work
JBx_278372	pE Sac	Spectinomycin resistance, CDF origin, P_{T7}:$bezE_{Sac}$	This Work
JBx_278373	pE Spa	Spectinomycin resistance, CDF origin, P_{T7}:$bezE_{Spa}$	This Work
JBx_266771	pEu	Spectinomycin resistance, CDF origin, P_{T7}:$bezE_{Ure}$	This Work
JBx_266772	pGEJ	Kanamycin resistance, ColE1 origin, P_{T7}:bezG:bezE:$bezE_{RI18}$	This Work
JBx_266773	pGJE	Kanamycin resistance, ColE1 origin, P_{T7}:bezG:bezJ:$bezE_{RI18}$	This Work
JBx_266768	pEGJ	Kanamycin resistance, ColE1 origin, P_{T7}:$bezE_{RI1}$:bezG:bezJ	This Work
JBx_266769	pEJG	Kanamycin resistance, ColE1 origin, P_{T7}:$bezE_{RI18}$:bezJ:bezG	This Work
JBx_266777	pJEG	Kanamycin resistance, ColE1 origin, P_{T7}:bezJ:$bezE_{RI1}$:bezG	This Work
JBx_266778	pJGE	Kanamycin resistance, ColE1 origin, P_{T7}:bezJ:bezG:$bezE_{RI18}$	This Work

JBx_266780	pJGEu	Kanamycin resistance, ColE1 origin, P_{T7}:bezJ:bezG:$bezE_{Ure}$	This Work
JBx_277814	pJGEu(HSF)	Kanamycin resistance, ColE1 origin, P_{T7}:bezJ:bezG:$bezE_{Ure}$(HSF)	This Work
JBx_277815	pTrc(HSF)	Kanamycin resistance, ColE1 origin, P_{Trc}:bezJ:bezG:$bezE_{Ure}$(HSF)	This Work
JBx_266785	pVP	Spectinomycin resistance, CDF origin, P_{Trc}:seTAL:baPAD	This Work
JBx_266784	pUVP	Spectinomycin resistance, CDF origin, P_{UV5}:seTAL:baPAD	This Work
JBx_266782	pPABA	Ampicillin resistance, p15a origin, P_{UV5}:pabC:cmlB(pabAB)	This Work
JBx_277816	$pJGE_{spa}$	Kanamycin resistance, ColE1 origin, P_{Trc}:bezJ:bezG:$bezE_{spa}$	This Work
JBx_266766	pBezG	Ampicillin resistance, ColE1 origin, P_{T7}:6H-bezG	Tsutsumi et al[1]
JBx_266765	pBezEu	Ampicillin resistance, ColE1 origin, P_{T7}:bezEu	This Work
JBx_266764	pBezE	Ampicillin resistance, ColE1 origin, P_{T7}:6H-$bezE_{RI18}$	Tsutsumi et al[1]
JBx_266767	pBezJ	Ampicillin resistance, ColE1 origin, P_{T7}:bezJ	This Work
JBx_266779	pMScar	Ampicillin resistance, ColE1 origin, P_{T7}:MScarlet-2I	This Work

Table 2. Strains used in this study

Strain ID	Base Strain	Plasmids/genotype	Markers
N/A	BL21 Star DE3 (Invitrogen)	F^- ompT hsdSB (r_B–, m_B–) gal dcm rne131 (DE3)	
N/A	NST74	ATCC 31884	
ID01	BL21 Star DE3	pGnAf, pEr	Carb, Spec
ID02	BL21 Star DE3	pGnJn, pEr	Carb, Spec
ID03	BL21 Star DE3	pGnJr, pEr	Carb, Spec
ID04	BL21 Star DE3	pJGEu(WT)	Kan

ID05	BL21 Star DE3	p JGEu (HSF)	Kan
ID06	BL21 Star DE3	p JGEu (HSF), pSty	Kan, Spec
ID07	BL21 Star DE3	p JGEu (HSF), pSty, pPABA	Kan, Spec, Carb
ID08	BL21 Star DE3	pTrc(HSF), pSty, pPABA	Kan, Spec, Carb
ID09	NST74	pTrc(HSF), pSty, pPABA	Kan, Spec, Carb
ID10	BL21 Star DE3	p JGEu (WT), pVP	Kan, Spec
ID11	BL21 Star DE3	p JGEu (HSF), pVP	Kan, Spec
ID12	BL21 Star DE3	p JGEu (WT), pVP, pPABA	Kan, Spec, Carb
ID13	BL21 Star DE3	p JGEu (WT), pUVP, pPABA	Kan, Spec, Carb
ID14	BL21 Star DE3	p JGEu (HSF), pVP, pPABA	Kan, Spec, Carb
ID15	BL21 Star DE3	p JGEu (HSF), pUVP, pPABA	Kan, Spec, Carb
ID16	BL21 Star DE3	pJGEspa, pVP, pPABA	Kan, Spec, Carb
ID17	BL21 Star DE3	pJGEspa, pUVP, pPABA	Kan, Spec, Carb

Table 3. Compounds produced in nitrene transfer reactions

**Compound ID**	**Structure**	**MW (g mol^{-1})**	**Reaction Type**	**Culture Additives**	**Strain ID**	**Max Titer (mgL^{-1})**
1a	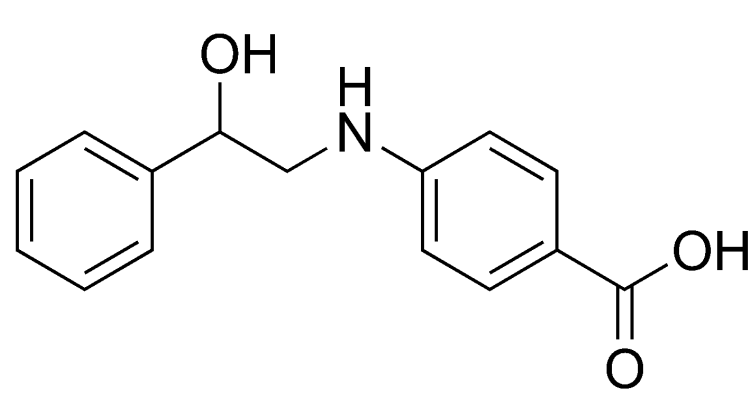	257	Biocatalytic	Glucose (15 g/L) PABA (0.5 mM) Sty (40 mM)	ID05	16.1
			Biocatalytic	Glucose (15 g/L) PABA	ID06	10.7

				(0.5 mM)<br>Phe<br>(8 mM)		
			Biosynthetic	Glucose<br>(15 g/L)	ID09	5.0
			Biosynthetic	Glucose<br>(15 g/L)<br>Phe<br>(8 mM)	ID07	8.2
2a	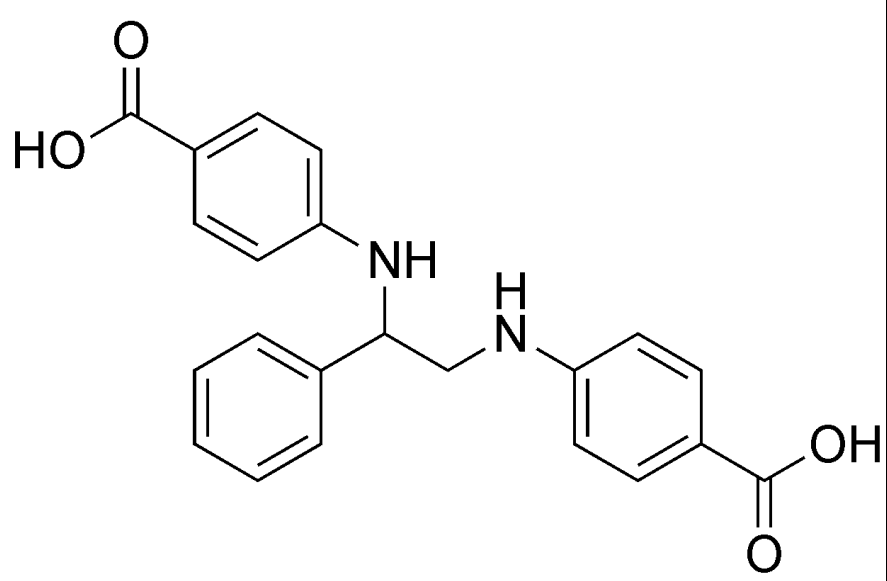	376	N/A			
3a	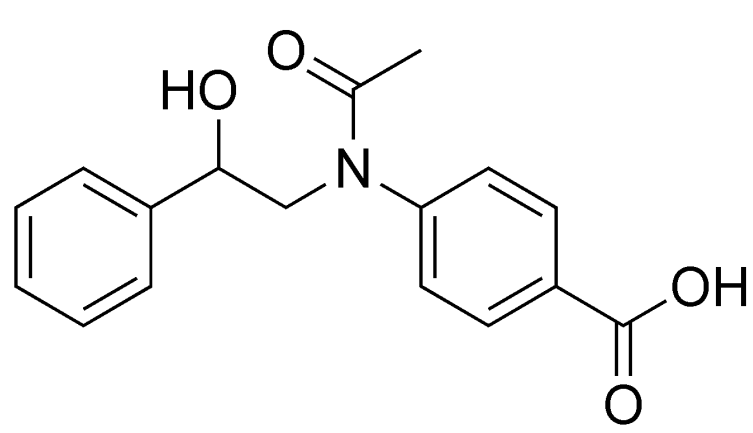	299	N/A			
1b	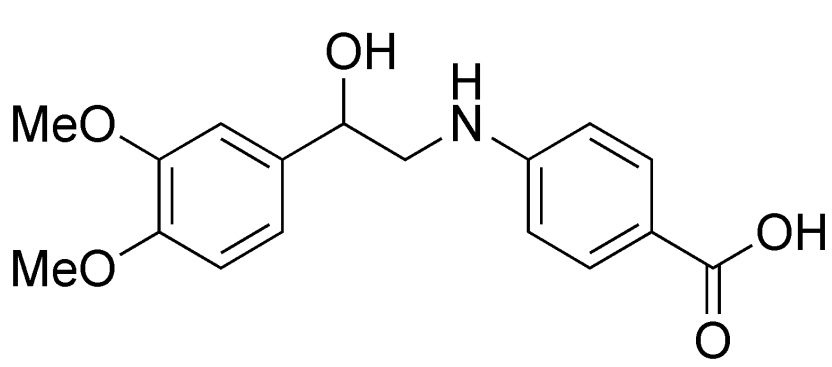	317	Biocatalytic	Glucose<br>(15 g/L)<br>PABA<br>(0.5 mM)<br>DMS<br>(2 mM)	ID04	8.7

2b	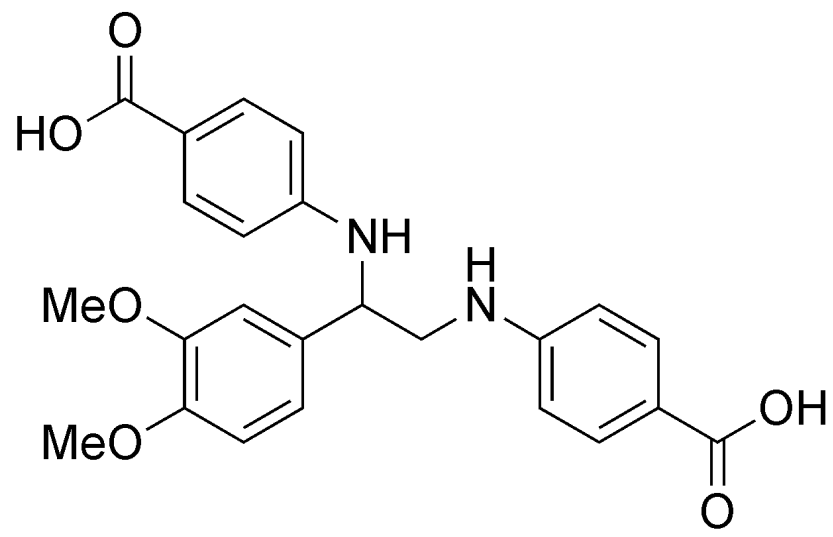	436	N/A			
3b	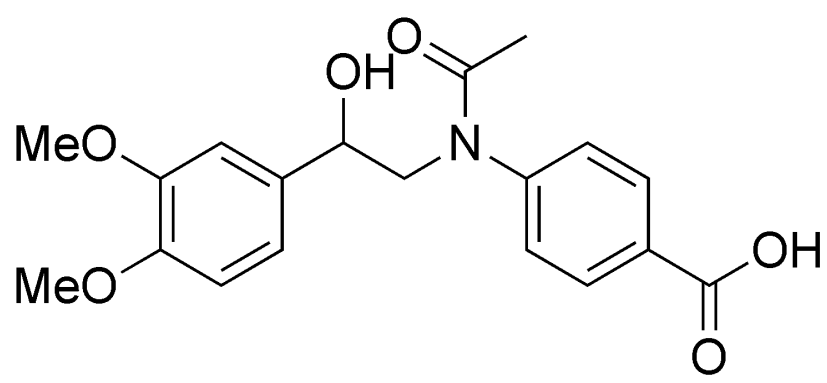	359	N/A			
1c	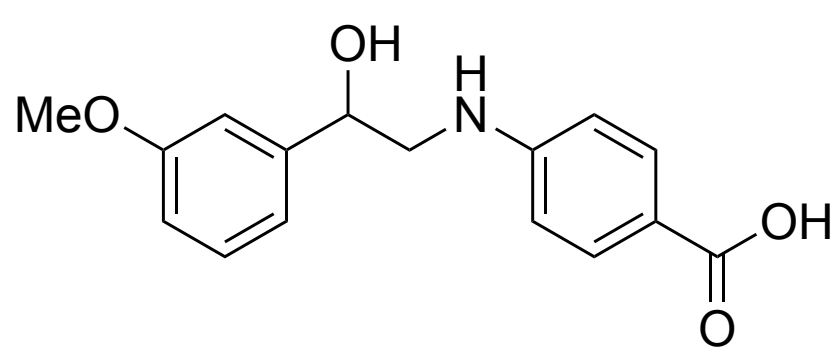	287	Biocatalytic	Glucose (15 g/L) PABA (0.5 mM) 3-VA (2 mM)	ID05	1.6
1d	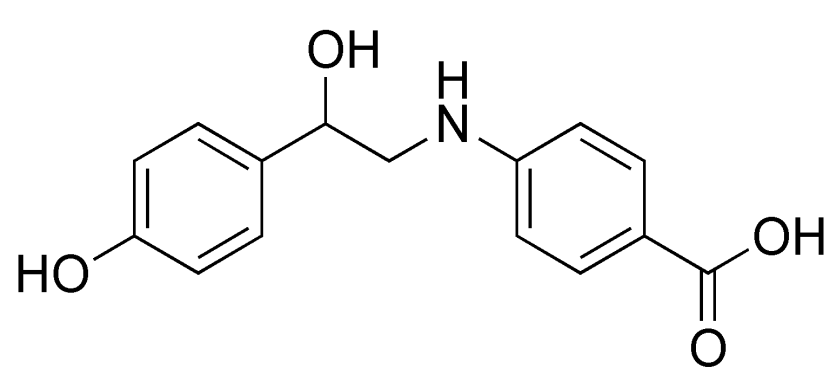	273	N/A			
2d	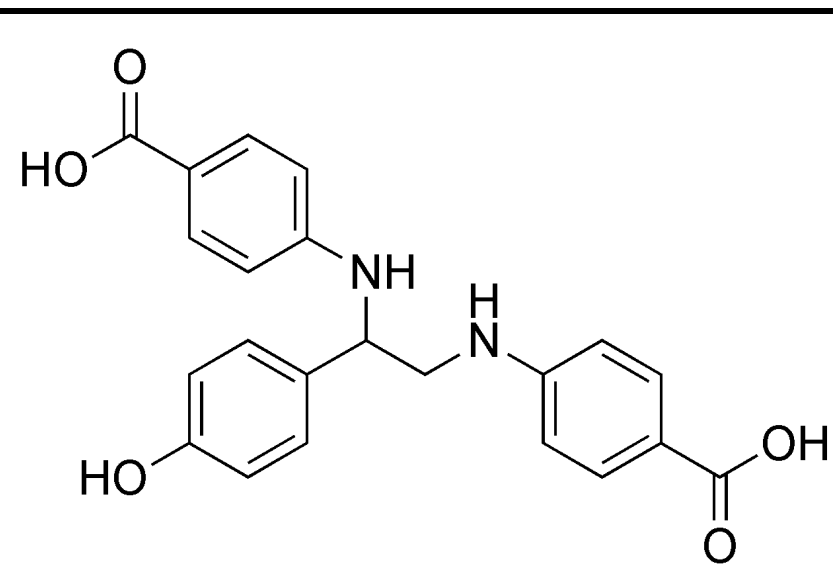	392	Biocatalytic	Glucose (15 g/L) PABA (0.5 mM) 4-VP (2 mM)	ID04	4.4
			Biocatalytic	Glucose (15 g/L) PABA	ID10	3.2

				(0.5 mM)<br>Tyr<br>(2 mM)		
			Biosynthetic	Glucose<br>(15 g/L)	ID16	2.0
			Biosynthetic	Glucose<br>(15 g/L)<br>Tyr<br>(2 mM)	ID16	6.2
4	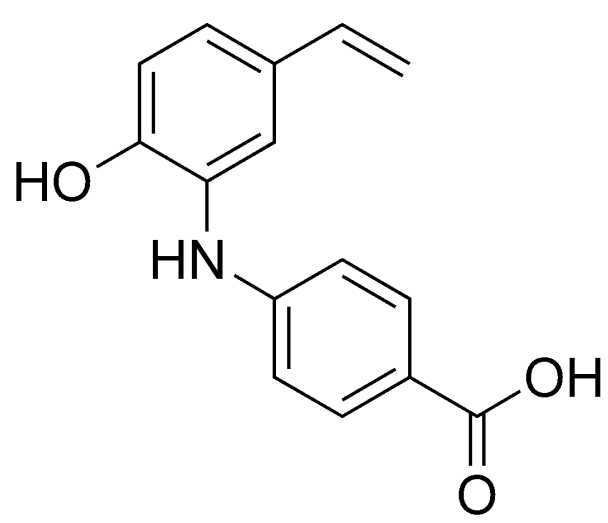	255	Biocatalytic	Glucose<br>(15 g/L)<br>PABA<br>(0.5 mM)<br>4-VP<br>(2 mM)	ID04	13.8
			Biocatalytic	PABA<br>(0.5 mM)<br>Tyr<br>(2 mM)	ID11	4.3
			Biosynthetic	Glucose<br>(15 g/L)	ID12	3.5
			Biosynthetic	Glucose<br>(15 g/L)<br>Tyr<br>(2 mM)	ID14	2.9
5		297	Biocatalytic	Glucose<br>(15 g/L)	ID04	1.4

	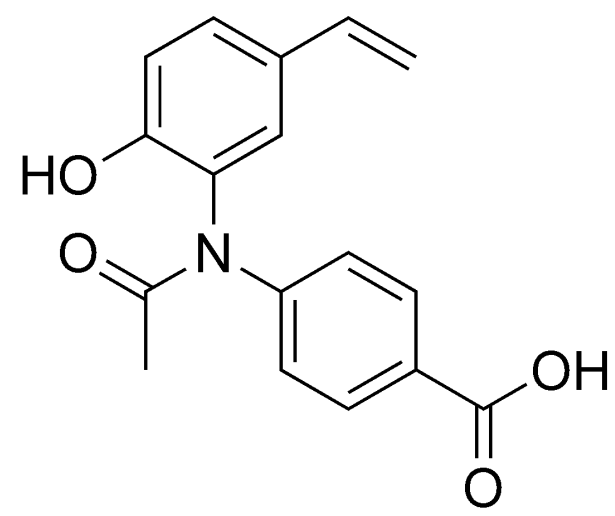			PABA (0.5 mM) 4-VP (2 mM)		
			Biocatalytic	Glucose (15 g/L) PABA (0.5 mM) Tyr (2 mM)	ID11	41.5
			Biosynthetic	Glucose (15 g/L)	ID14	3.3
			Biosynthesis	Glucose (15 g/L) Tyr (2 mM)	ID14	22.2
6	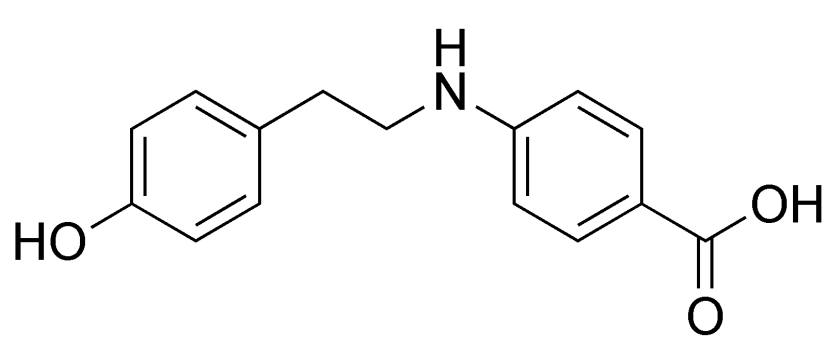	257	Biocatalytic	Glucose (15 g/L) PABA 4-VP	ID04	7.0
			Biocatalytic	Glucose (15 g/L) PABA Tyr	ID10	14.0
			Biosynthetic	Glucose (15 g/L)	ID16	29.3

			Biosynthetic	Glucose (15 g/L)Tyr	ID13	14.1
7	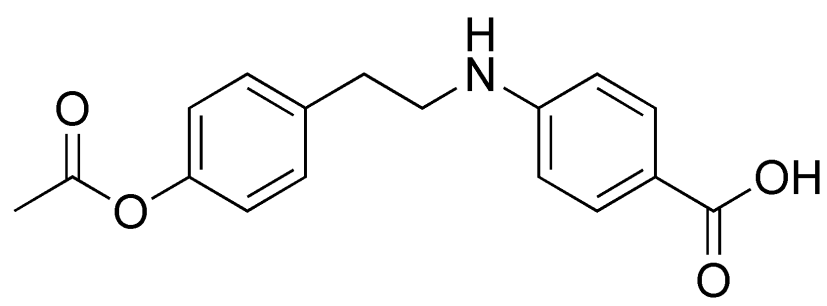	299	Biocatalytic	Glucose (15 g/L) PABA (0.5 mM) 4-VP (2 mM)	ID04	0.0
			Biocatalytic	Glucose (15 g/L) PABA (0.5 mM) Tyr (2 mM)	ID10	11.0
			Biosynthetic	Glucose (15 g/L)	ID17	0.0
			Biosynthetic	Glucose (15 g/L) Tyr (2 mM)	ID17	5.7

Table 4. Amino acid sequences of Proteins used

Protein Name	Sequence
BezG$_{Niveus}$	MTDELRATDSMWQGSGVDLDAYLARVGYQGDVAPDLTTLRGLHTA<br>HVDAIAFDNLDAVLGRTVVPIDLDSVQEKIVRQGRGGWCLEQVVLM<br>AAVLDRIGFTFTAFAGRTRVRTGSKFGPALHVALCVEADGARWIHDV<br>SFGAFGPHEPIRLAENARLDGDWSFDLLREPTGEHVLRFLRPEGPVEL<br>YGFTTDVRYPSDFELLNHFCLTHPRSPFNHRMVLQRTQPEVRHLLAG<br>STLTEIRPEGPPTVRELDVDELLSAPREIFGITLDPGDVQALGKILDGP

BezE$_{RI18}$	MGDSTSASTSTSTAWSEPRFRRRPSVSSPPAQLPFEQPNVLRLAPGLRT<br>LRSRGAVHRVRTVEGDEAWLVTGHAWVRRLLDDDRLGRSNPDPEA<br>AAKDSGSALLASLLGGFATDHARLRALLEPHFSPERMLALRPYVEKL<br>TRQLLDGLAARKPPVDLRQELALSLPIQVMCEWLGVPQEDKDRFGG<br>WTQDAANVGDPARSKQGLGQLFGYCRQLVADKRRNPGDDAISRICA<br>TEGIDDNEVLGLTALLLFGGYETTVARIGTCVSLLLADPEQWQALLRD<br>PDLVPGAVEETLRMSMPNPHTGGMPRYALTDFEIDGVTVRTGDFVLL<br>NIIAANHDETAFADPDRVDVTRDTTGSLAFGHGAHHCVGAMLARMQ<br>LQVVLSQLVARFPTLRLAVGAEELRLRDSTLIGGLVHLPVTW
BezE$_{Ureilyticus}$	MSVNAPAQLPFEQTHLLQVAPLLRTLQSQGTVHRVRTPEGDEAWLV<br>TGHAQVQQLLDDDRLSRSNPEPGAARDSGSALLDSLLGSFANDHARL<br>RSLLEPHFSPKLMLALRSHVETLTEQLLDEMAGQTPPVDLRLALALPL<br>PILVMCEWLGVPHQDKDQFGAWTQDAANVQDRVRSKQGMAELFGY<br>CQQLIADKRQNPGDDVISRIGATEGINDQEVVGLTALLLFGGYETTVA<br>RIGTGALLLLANPEQRQALLDDPSLIPSGVEEILRMSMPNPHNGGMPR<br>YALTDLEIDGTTIRAGDLVLLNIIAANHDQTVFTDPERVDVTRQTAGS<br>LTFGHGTHYCVGAPLARMQLQVAIARLVARFPTMRLAVDVKELKLR<br>HDTLLGGLVELPVTWGS<br>HSF mutation loci are in green
BezE$_{Spa}$	MMNTVAQLPFTQTHVLDVAPALRLLQSRGKVHRVRTPEGVPAWLVT<br>GHAEVQQLLDDDRLSRSDPGGRDGGTALLNKLLGPLADDHPRLRSLL<br>EPQFTPERLEPLRAVVEKLTEQHLDELATRTPPVDLRPTLAMSLPILVL<br>CEWLGVPAEDKGRFSVWTQDAAGVQDPERSQRGLAELFGYCRQLV<br>AAKRQDPGDDVISRLIATAGIGDTEVVALTALLLFGGYETTVARIGTG<br>VLLLLTNPDQWAAVRADPALVPATVDELLRRSMPNPHNGGMPRFAV<br>TGFEIDGAAIRAGDLVLLNIIAANHDETAFPDPDRLDITRPTAGSLAFG<br>YGRHSCVGAPLARMVLRVALSRLITRFPDLRLAVGVDELKLRHETLV<br>GGLVELPVTWGPRGS
BezJ$_{Niveus}$	MTAAREVPEYERDLLVQLTLRWGKRVAVKKDELDLDGHFDAALPDF<br>PEHLVPVLALPGAGRLDRDARERILSASWIAYNAKTAAIEDEIILPACR<br>LMLQDRIPVRRDDVAVDALHQTIIDEHYHILMCHNAVGVTRRRRDLA<br>DVRFAPGVWSVVRGRDAVRAGLSGFDRDIVDIAFSLAAETTISGFLST<br>LATAQEIQPMNRITTDLHRRDESGHAVVFRELCGSLYRNLDGVQQRM<br>FREALVTGLTAFRSADLDPWVAVAAQGGFEIDTGQLSEWAASRPAPA<br>RDTGPLQLLLDDLGISHDLVEMVRR
AtPAL	MDQIEAMLCGGGEKTKVAVTTKTLADPLNWGLAADQMKGSHLDEV<br>KKMVEEYRRPVVNLGGETLTIGQVAAISTVGGSVKVELAETSRAGVK<br>ASSDWVMESMNKGTDSYGVTTGFGATSHRRTKNGTALQTELIRFLN<br>AGIFGNTKETCHTLPQSATRAAMLVRVNTLLQGYSGIRFEILEAITSLL<br>NHNISPSLPLRGTITASGDLVPLSYIAGLLTGRPNSKATGPDGESLTAK

	EAFEKAGISTGFFDLQPKEGLALVNGTAVGSGMASMVLFEANVQAV LAEVLSAIFAEVMSGKPEFTDHLTHRLKHHPGQIEAAAIMEHILDGSS YMKLAQKVHEMDPLQKPKQDRYALRTSPQWLGPQIEVIRQATKSIER EINSVNDNPLIDVSRNKAIHGGNFQGTPIGVSMDNTRLAIAAIGKLMF AQFSELVNDFYNNGLPSNLTASSNPSLDYGFKGAEIAMASYCSELQYL ANPVTSHVQSAEQHNQDVNSLGLISSRKTSEAVDILKLMSTTFLVGIC QAVDLRHLEENLRQTVKNTVSQVAKKVLTTGINGELHPSRFCEKDLL KVVDREQVFTYVDDPCSATYPLMQRLRQVIVDHALSNGETEKNAVT SIFQKIGAFEEELKAVLPKEVEAARAAYGNGTAPIPNRIKECRSYPLYR FVREELGTKLLTGEKVVSPGEEFDKVFTAMCEGKLIDPLMDCLKEWN GAPIPIC
SeTAL	MTQVVERQADRLSSREYLARVVRSAGWDAGLTSCTDEEIVRMGASA RTIEEYLKSDKPIYGLTQGFGPLVLFDADSELEQGGSLISHLGTGQGAP LAPEVSRLILWLRIQNMRKGYSAVSPVFWQKLADLWNKGFTPAIPRH GTVSASGDLQPLAHAALAFTGVGEAWTRDADGRWSTVPAVDALAA LGAEPFDWPVREALAFVNGTGASLAVAVLNHRSALRLVRACAVLSA RLATLLGANPEHYDVGHGVARGQVGQLTAAEWIRQGLPRGMVRDG SRPLQEPYSLRCAPQVLGAVLDQLDGAGDVLAREVDGCQDNPITYEG ELLHGGNFHAMPVGFASDQIGLAMHMAAYLAERQLGLLVSPVTNGD LPPMLTPRAGRGAGLAGVQISATSFVSRIRQLVFPASLTTLPTNGWNQ DHVPMALNGANSVFEALELGWLTVGSLAVGVAQLAAMTGHAAEGV WAELAGICPPLDADRPLGAEVRAARDLLSAHADQLLVDEADGKDFG
FDC	MRKLNPALEFRDFIQVLKDEDDLIEITEEIDPNLEVGAIMRKAYESHLP APLFKNLKGASKDLFSILGCPAGLRSKEKGDHGRIAHHLGLDPKTTIK EIIDYLLECKEKEPLPPITVPVSSAPCKTHILSEEKIHLQSLPTPYLHVSD GGKYLQTYGMWILQTPDKKWTNWSIARGMVVDDKHITGLVIKPQHI RQIADSWAAIGKANEIPFALCFGVPPAAILVSSMPIPEGVSESDYVGAI LGESVPVVKCETNDLMVPATSEMVFEGTLSLTDTHLEGPFGEMHGYV FKSQGHPCPLYTVKAMSYRDNAILPVSNPGLCTDETHTLIGSLVATEA KELAIESGLPILDAFMPYEAQALWLILKVDLKGLQALKTTPEEFCKKV GDIYFRTKVGFIVHEIILVADDIDIFNFKEVIWAYVTRHTPVADQMAFD DVTSFPLAPFVSQSSRSKTMKGGKCVTNCIFRQQYERSFDYITCNFEK GYPKGLVDKVNENWKRYGYK
BaPAD	MGKSLKKEQIEMTNQPREDVKDFVGSHMIYTYENGWEYEIYIKNDKT IDYRIHSGMVAGRWVRDQEVDLVKLTEGVYKVSWTEPTGTDVSLNF MPNEKRMHGIIFFPKWVHEHPEITVCYQNDYIDVMEESREKYETYPK FVVPEFADITFLENAGIDNQETISKAPYEGMTDDIRAGKLK
$PabC_{E. Coli}$	MFLINGHKQESLAVSDRATQFGDGCFTTARVIDGKVSLLSAHIQRLQD ACQRLMISCDFWPQLEQEMKTLAAEQQNGVLKVVISRGSGGRGYST LNSGPATRILSVTAYPAHYDRLRNEGITLALSPVRLGRNPHLAGIKHL

	NRLEQVLIRSHLEQTNADEALVLDSEGWVTECCAANLFWRKGNVVY<br>TPRLDQAGVNGIMRQFCIRLLAQSSYQLVEVQASLEESLQADEMVIC<br>NALMPVMPVCACGDVSFSSATLYEYLAPLCERPN
CmlB$_{Strep.\ Venezualae}$<br>(pabAB)	MRTLLIDNYDSFTHNLFQYIGEATGQPPVVVPNDADWSRLPLEDFDAI<br>VVSPGPGSPDRERDFGISRRAITDSGLPVLGVCLGHQGIAQLFGGTVG<br>LAPEPMHGRVSEVRHTGEDVFRGLPSPFTAVRYHSLAATDLPDELEPL<br>AWSDDGVVMGLRHREKPLWGVQFHPESIGSDFGREIMANFRDLALA<br>HHRARRDAADSPYELHVRRVDVLPDAEEVRRGCLPGEGATFWLDSS<br>SVLEGASRFSFLGDDRGPLAEYLTYRVADGVVSVRGSDGTTTRTRRP<br>FFSYLEEQLERRRVPVAPDLPFEFNLGYVGYLGYELKAETTGDPAHRS<br>PHPDAAFLFADRAIALDHQEGCCYLLALDRRGHDDGARAWLRETAE<br>TLTGLAVRVPAEPTPAMVFGVPEAAAGFGPLARARHDKDAYLKRIDE<br>CLKEIRNGESYEICLTNMVTAPTEATALPLYSALRAISPVPYGALLEFP<br>ELSVLSASPERFLTIGADGGVESKPIKGTRPRGGTAEEDERLRADLAG<br>REKDRAENLMIVDLVRNDLNSVCAIGSVHVPRLFEVETYAPVHQLVS<br>TIRGRLRPGTSTAACVRAAFPGGSMTGAPKKRTMEIIDRLEEGPRGVY<br>SGALGWFALSGAADLSIVIRTIVLADGRAEFGVGGAIVSLSDQEEEFTE<br>TVVKARAMVTALDGSAVAGAR

Table 5. P450 percent identity matrix

Accession Code	Abrev.	RI18	NIV	LWa	SAc	LCa	Ure	SPa
LC177364.1	RI18		87.692	84.103	82.308	77.436	74.872	68.462
WP_031228687.1	NIV	87.692		82.051	85.128	77.179	77.436	68.462
WP_093588991.1	LWa	84.103	82.051		83.29	83.033	72.751	70.619
WP_029184464.1	SAc	82.308	85.128	83.29		77.121	71.979	67.866
WP_191297479.1	LCa	77.436	77.179	83.033	77.121		70.437	68.38
WP_235989764.1	Ure	74.872	77.436	72.751	71.979	70.437		71.465
WP_018812029.1	SPa	68.462	68.462	70.619	67.866	68.38	71.465	

Table 6. Representative Plasmid maps:

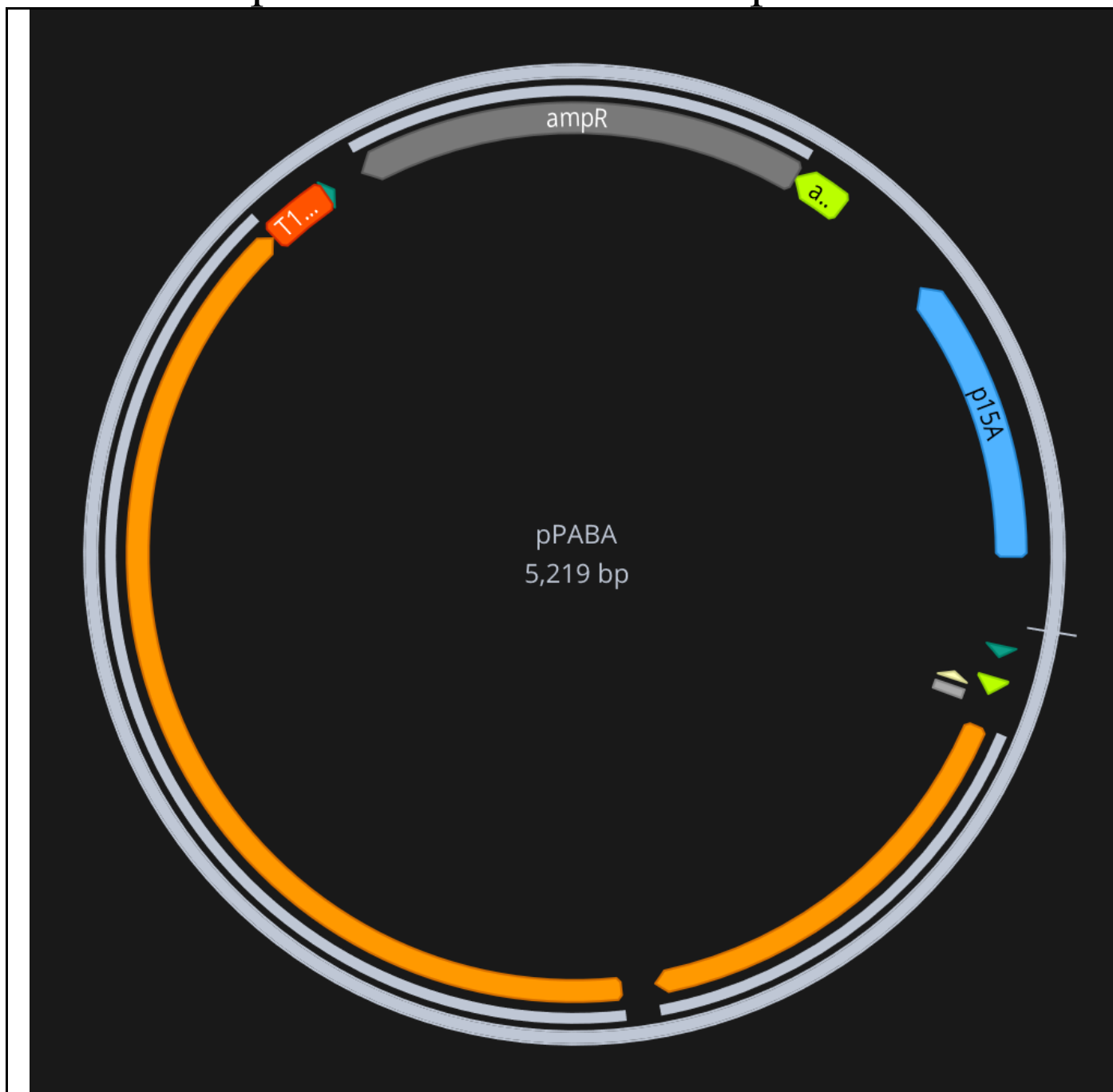

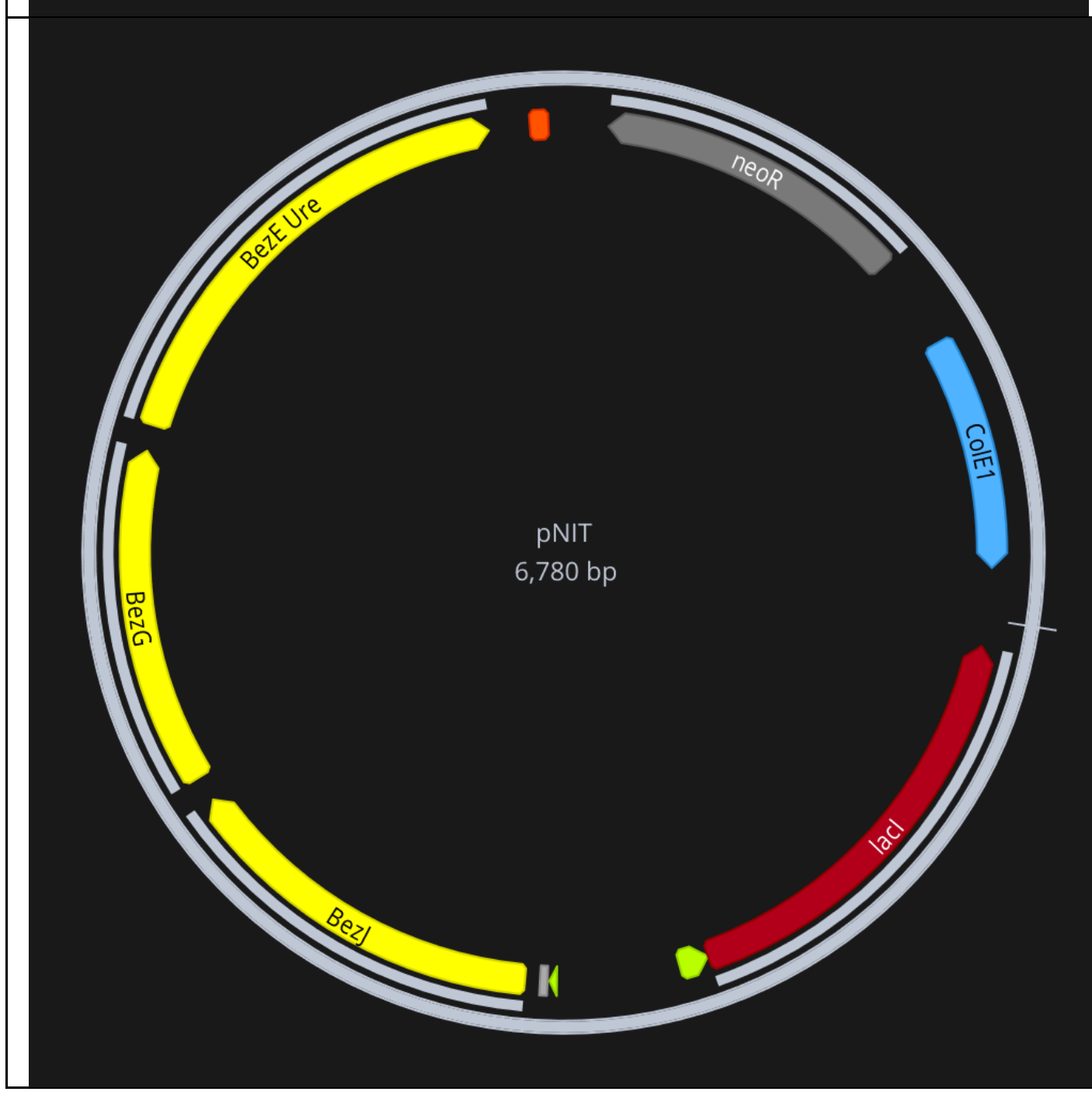

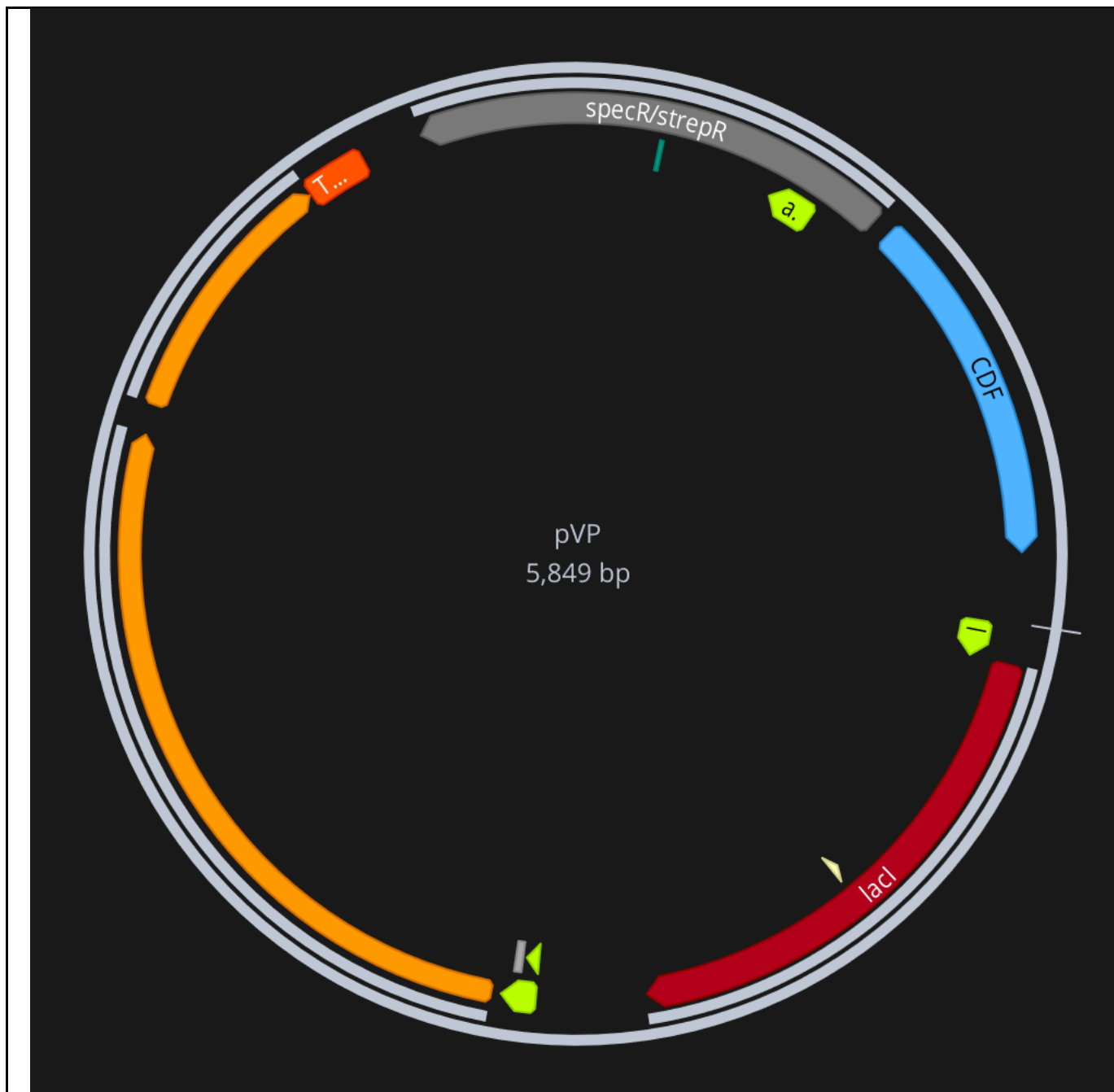
specR/strepR
a.
CDF
pVP
5,849 bp
T...
lacI

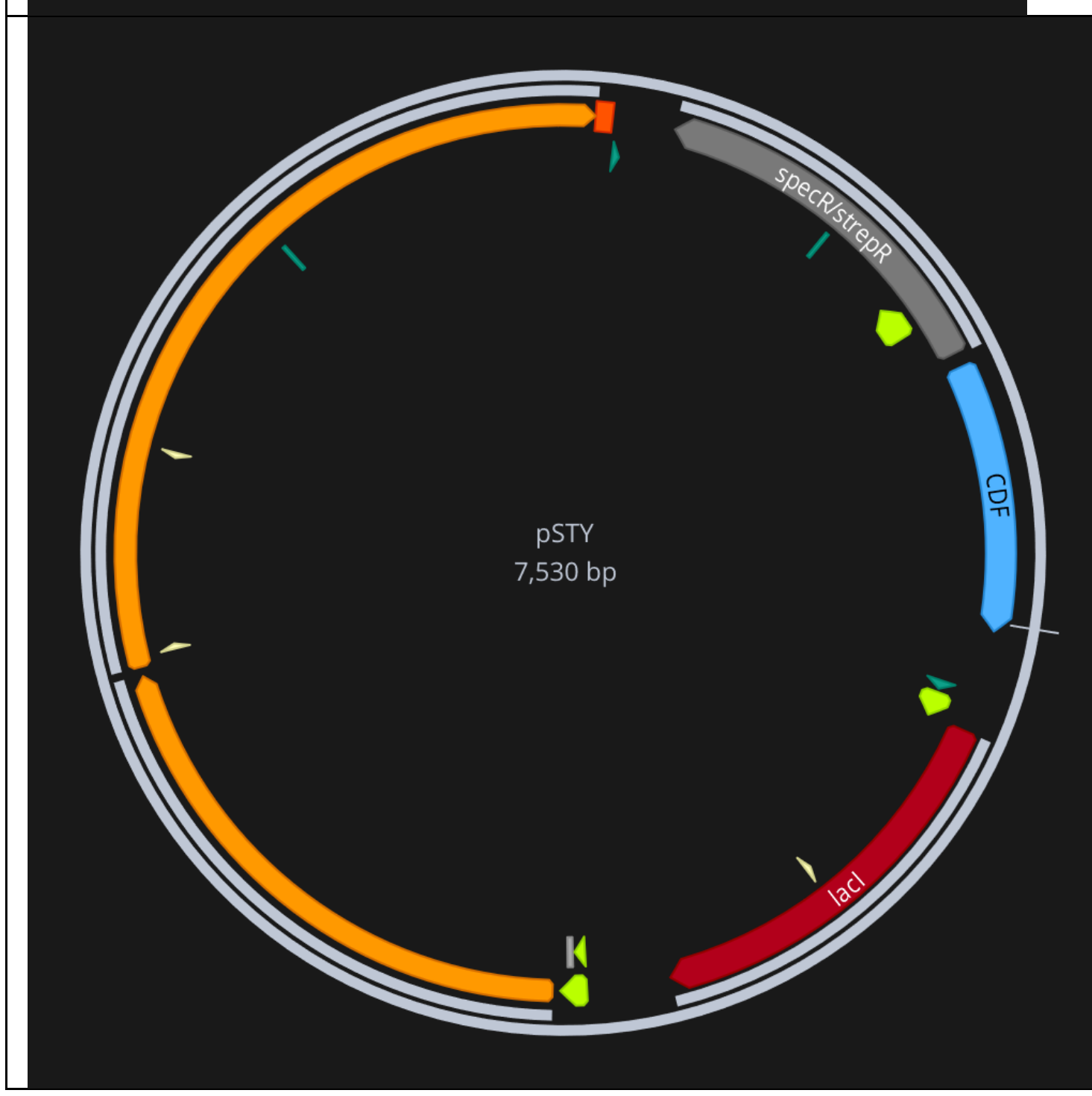
specR/strepR
CDF
pSTY
7,530 bp
lacI

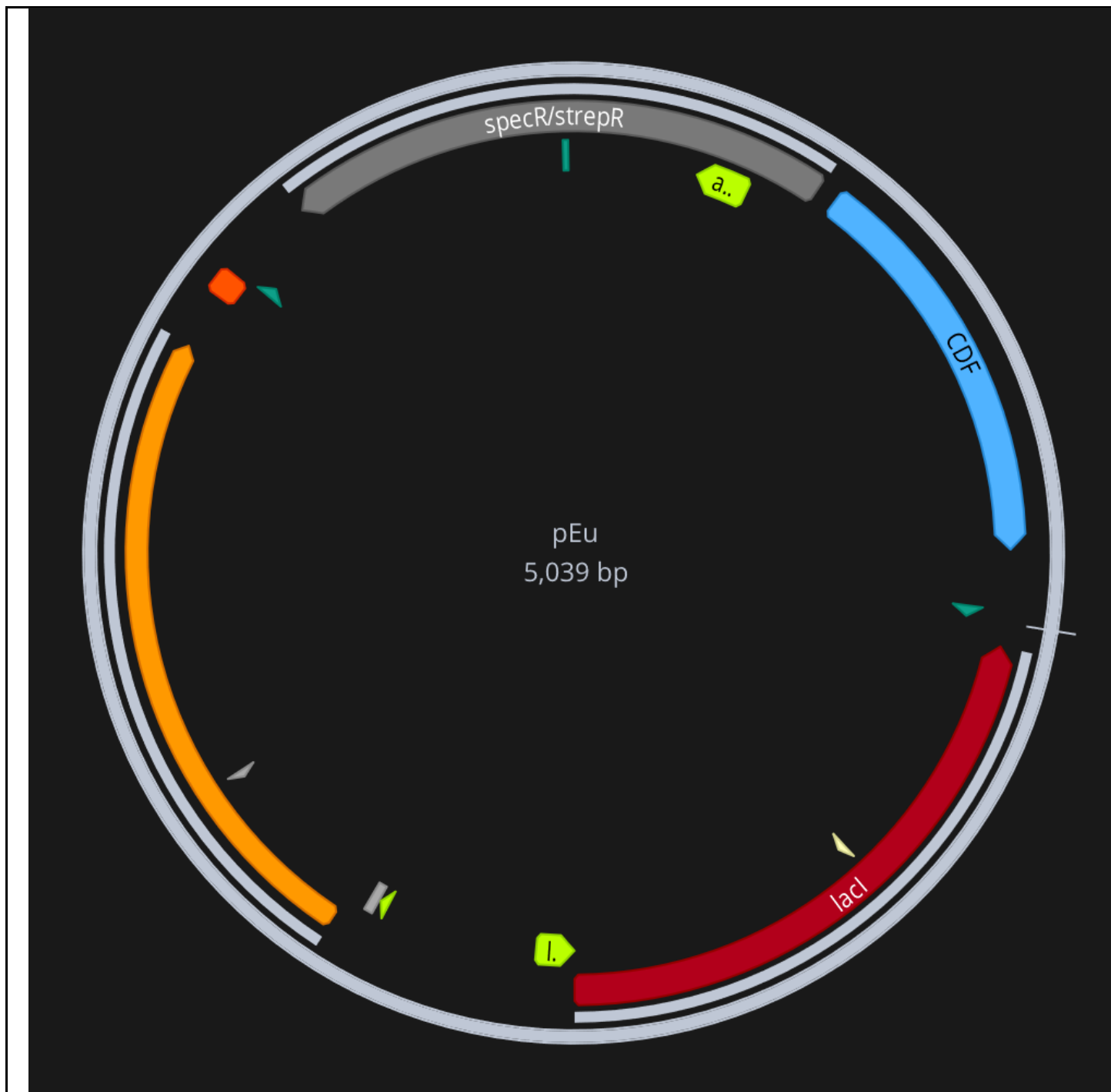
specR/strepR
a..
CDF
pEu
5,039 bp
lacI
l.

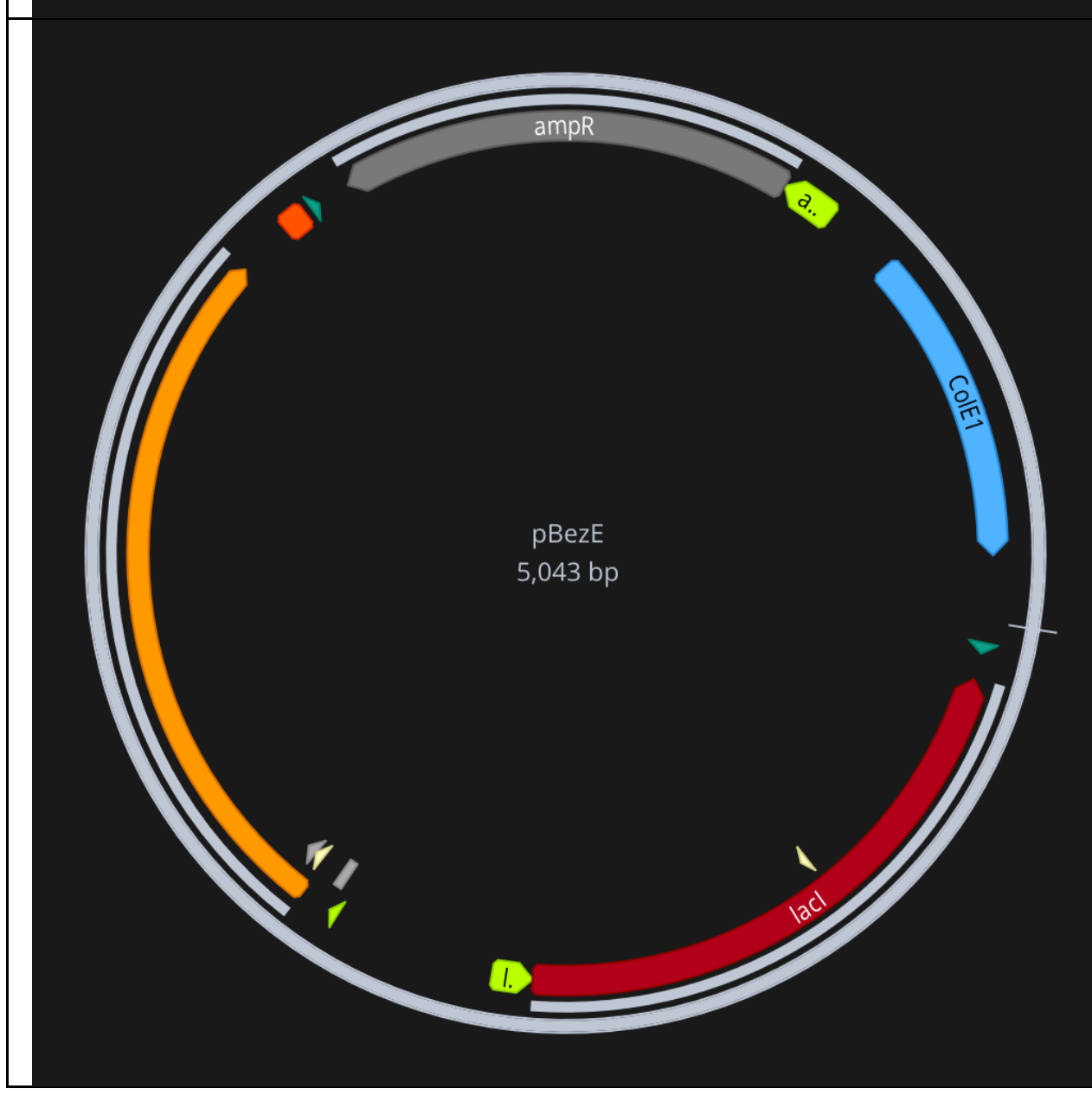
ampR
a..
ColE1
pBezE
5,043 bp
lacI
l.

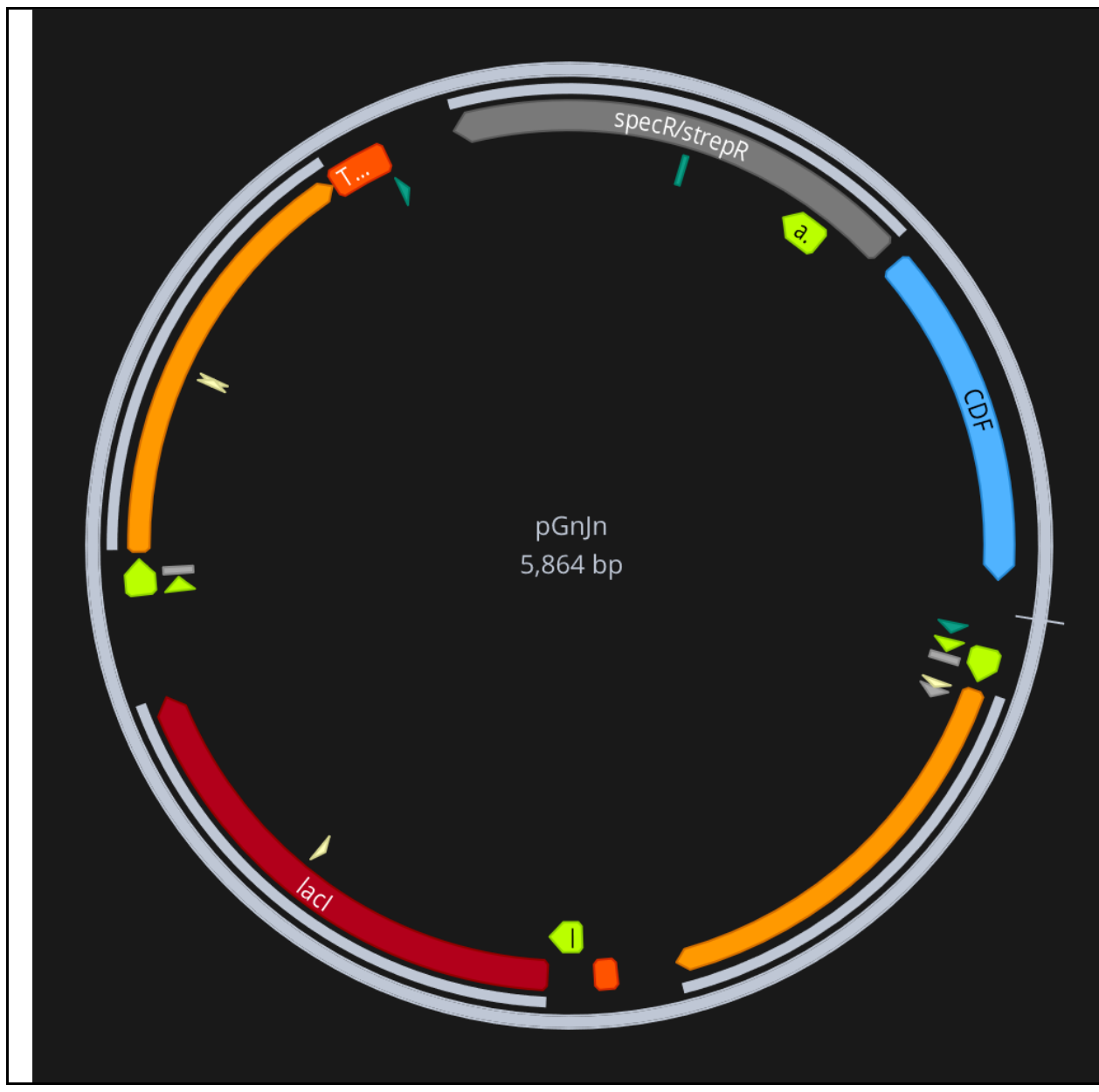

Table 7. Primers used for $BezE_{Ure}$ Mutagenesis

Name	Sequence
ID_BezE.R134X.F	ACTCCGCCTGTTGACCTGNDTTTGGCGCTGGCGTTAC
ID_BezE.R134.R	CAGGTCAACAGGCGGAGTCTGAC
ID_BezEu.A87.R	AAAACTGCCTAACAGGCTGTCCAGC
ID_BezEu.A87X.F	GACAGCCTGTTAGGCAGTTTTNDTAACGATCATGCGCGTCTGCGTTCTC
ID_BezEu.C340S.F	CGGTACACATTATAGCGTTGGTGCACCGTTGGCCCG
ID_BezEu.C340S.R	CAACGCTATAATGTGTACCGTGGCCGAACGTGAGAGAAC
ID_BezEu.I305.R	ATTTAACAGCACCAGGTCACCCGCAC
ID_BezEu.I305X.F	GTGACCTGGTGCTGTTAAATNDTATCGCTGCGAACCATGATCAAACGG
ID_BezEu.L83.R	GCTGTCCAGCAATGCGCTACCG
ID_BezEu.L83X.F1	GCATTGCTGGACAGCCTGNDTGGCAGTTTTGCTAACGATCATGCGCG
ID_BezEu.T231.R	ACCGCCGAACAATAACAACGCGGTCAGGCCCACG
ID_BezEu.T231X.F	GTTGTTATTGTTCGGCGGTTATGAANDTACCGTCGCGCGCATCGGCAC
ID_BezEu.V233.R	GGTGGTTTCATAACCGCCGAACAATAAC
ID_BezEu.V233X.F	CGGCGGTTATGAAACCACCNDTGCGCGCATCGGCACGGGTG
ID_BezEu.Y339X.F	CGTTCGGCCACGGTACACATNDTTGTGTTGGTGCACCGTTGGCCCG
ID_BezEu.Y339.R	GTGTACCGTGGCCGAACGTGAGAGAACC

Supplementary Discussion:

This work highlights the challenges of introducing unnatural reactivity into biological systems. The promiscuity of BezG and the aziridine capture by PABA both limit the selectivity needed for commercially viable processes. These issues are further compounded when using vinyl phenol as a substrate, which leads to four identifiable products—and likely additional species that escaped isolation and characterization. Nonetheless, our media optimization, protein engineering, and homolog screening demonstrate that these unique compounds arise from P450-mediated pathways, expanding the scope of achievable C–N bond-forming reactions. Elucidating the precise mechanisms underlying their formation will require deeper analysis beyond the scope of this study. Independent feeding experiments (Fig. S1) also suggest that tyramine may originate from diamine 2d. However, given the extremely low conversion of 2d to 6 and the increased specificity of BezE$_{spa}$, this is unlikely to represent a major biosynthetic route. We also cannot rule out the formation of additional, unexpected compounds that could not be isolated or characterized under our purification conditions.

Experimental Procedures and Characterization of Authentic Standards

General Procedure for the Isolation of Biosynthetic Products from Bulk Fermentation

1.0-3.0 L of biosynthetic culture quenched with acetonitrile (3:2 ratio culture : acetonitrile) was concentrated under reduced pressure to give approximately 100 mL of a brown, thick slurry. The remaining water was removed by blowing a stream of nitrogen on it overnight, furnishing a brown, viscous paste which was subsequently treated and sonicated with 500 mL of methanol with 0.1% formic acid, causing the methanol layer to turn slightly brown. The methanol was decanted and stored. The remaining slurry was extracted with two additional 500-mL portions of methanol containing 0.1% formic acid, with the assistance of sonication. The methanol extracts were combined and concentrated to give another viscous, brown paste, which was purified by reverse-phase chromatography (0-40% acetonitrile/water with 0.1% formic acid). Fractions were analyzed by LC-MS, and potential product-containing fractions were pooled and concentrated under reduced pressure.

Isolation of **2d** from Biosynthesis

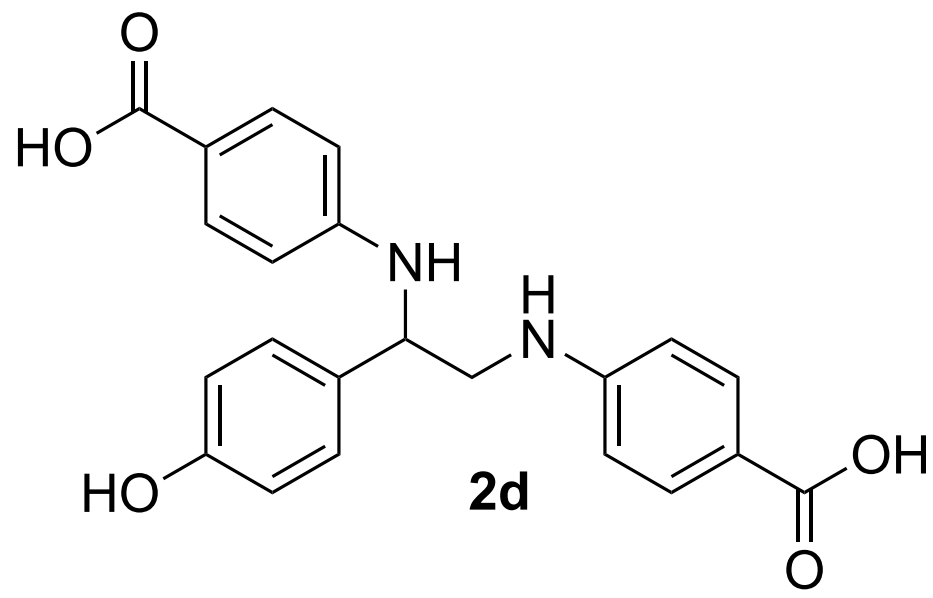

2d

From 2.0 L of bulk fermented culture quenched with 1.0 L of acetonitrile, **2d** (0.87 mg, 0.002 mmol) was isolated as a light-brown, flaky solid. The isolated solid was analyzed by ^{1}H-NMR and ^{13}C-NMR to elucidate structure.

Isolation of **7** from Biosynthesis

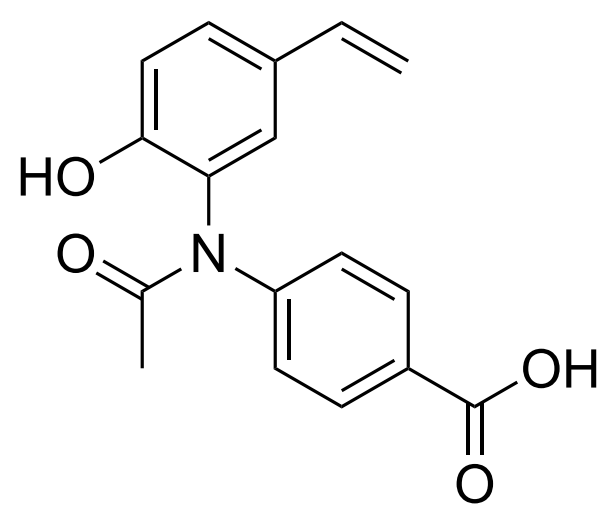

5

From 3.0 L of bulk fermented culture quenched with 1.5 L of acetonitrile, **5** (1.2 mg, 0.004 mmol) was isolated as an impure dark-brown oil. Isolated compounds were analyzed by ^{1}H-NMR and ^{13}C-NMR to elucidate structure.

Isolation of **5** from Biosynthesis

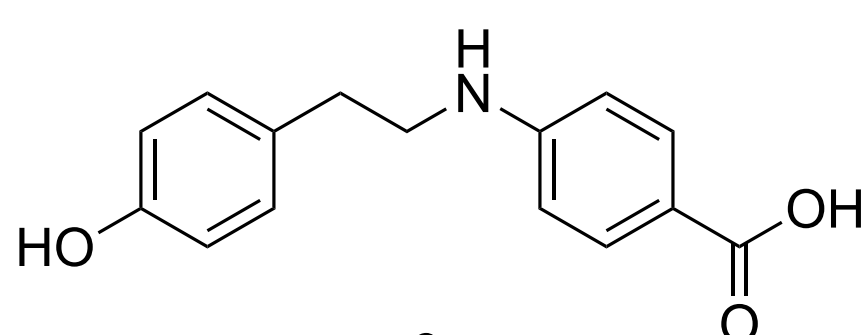

6

From 3.0 L of bulk fermented culture quenched with 1.5 L of acetonitrile, **6** (3.4 mg, 0.013 mmol) was isolated as an impure, golden-brown, flaky solid. Isolated compounds were analyzed by ^{1}H-NMR and ^{13}C-NMR to elucidate structure.

Synthesis of 2-bromo-1-phenylethan-1-one (**S1**)

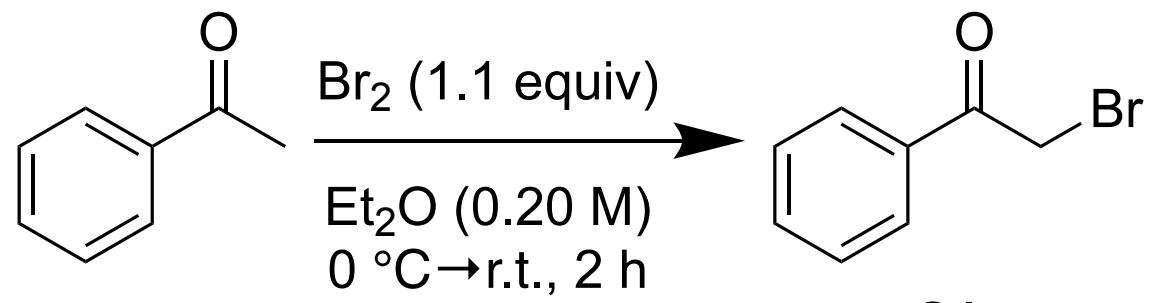

Into a 500-mL beaker with magnetic stir bar was added acetophenone (4.81 g, 40.0 mmol) and 200 mL of diethyl ether (0.20 M). This mixture was allowed to stir until fully dissolved before the reaction mixture was placed in an ice bath and allowed to cool to 0 °C over 15 min. Once cooled, bromine (6.32 g, 40.0 mmol, 1.0 equiv) was added dropwise over 10 min, producing a red-orange solution, which slowly emitted an acidic vapor. Once all the bromine was added, the reaction mixture was removed from the ice bath and allowed to warm to room temperature over the course of 1 h. During this time, the reaction mixture slowly turned from orange to yellow, releasing a colorless vapor (presumably HBr gas). Once this color change had occurred, the reaction was quenched with addition of distilled water. This mixture was allowed to stir for 20 min before being transferred into a separatory funnel. The aqueous layer was removed, and the ether layer was washed sequentially with distilled water, saturated bicarbonate solution, and brine, then dried over $MgSO_4$. The compound was concentrated under reduced pressure to give a light yellow solid. The solid was recrystallized from hot ether/hexanes to give **S1** (6.45 g, 32.4 mmol, 81% yield) as a white crystalline solid.

^{1}H NMR (600 MHz, $CDCl_3$) δ 8.01 – 7.96 (m, 2H), 7.61 (ddt, *J* = 8.7, 7.0, 1.3 Hz, 1H), 7.52 – 7.47 (m, 2H), 4.45 (s, 2H).

^{13}C NMR (151 MHz, $CDCl_3$) δ 191.38, 134.09, 134.06, 129.03, 128.97, 31.05.

HRMS (ESI): *m/z* for $C_8H_7BrNaO^+$ $[M+Na]^+$ calcd.: 220.9578, found: 220.9585.

Synthesis of *tert*-butyl 4-((2-oxo-2-phenylethyl)amino)benzoate (**S2**)

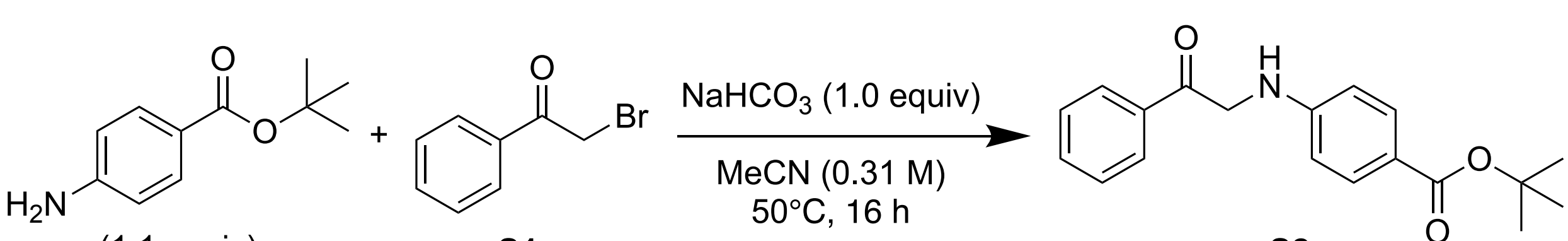

Into a 250-mL round bottom flask containing a stir bar was added **S1** (5.00 g, 25.1 mmol), *tert*-butyl 4-aminobenzoate (5.34 g, 27.6 mmol, 1.1 equiv), and sodium bicarbonate (2.11 g, 25.1

mmol, 1.0 equiv). To this mixture was added 80 mL of acetonitrile (0.31 M), and the reaction mixture was heated to 50 °C in an oil bath and allowed to stir overnight under an atmosphere of air. After stirring overnight, the reaction appeared light-yellow in color with a white solid. The reaction mixture was allowed to cool to room temperature, during which time a yellow solid began to crystallize from solution. The solid was collected by filtration through a medium glass frit, washed with water to remove excess sodium bicarbonate, and purified by recrystallization from hot acetonitrile/water to give **S2** (5.00 g, 16.1 mmol, 64% yield) as a yellow crystalline solid.

^{1}H NMR (600 MHz, DMSO) δ 8.11 – 8.04 (m, 2H), 7.71 – 7.66 (m, 1H), 7.66 – 7.62 (m, 2H), 7.56 (t, *J* = 7.8 Hz, 2H), 6.74 – 6.68 (m, 2H), 6.65 (t, *J* = 5.5 Hz, 1H), 4.77 (d, *J* = 5.5 Hz, 2H), 1.50 (s, 9H).

^{13}C NMR (151 MHz, DMSO) δ 195.98, 165.26, 152.00, 134.99, 133.61, 130.65, 128.79, 127.90, 118.35, 111.44, 79.01, 49.38, 27.98.

HRMS (ESI): *m/z* for $C_{19}H_{22}NO_3^+$ $[M+H]^+$ calcd.: 312.1594, found: 312.1580.

Synthesis of *tert*-butyl 4-((2-hydroxy-2-phenylethyl)amino)benzoate (**S3**)

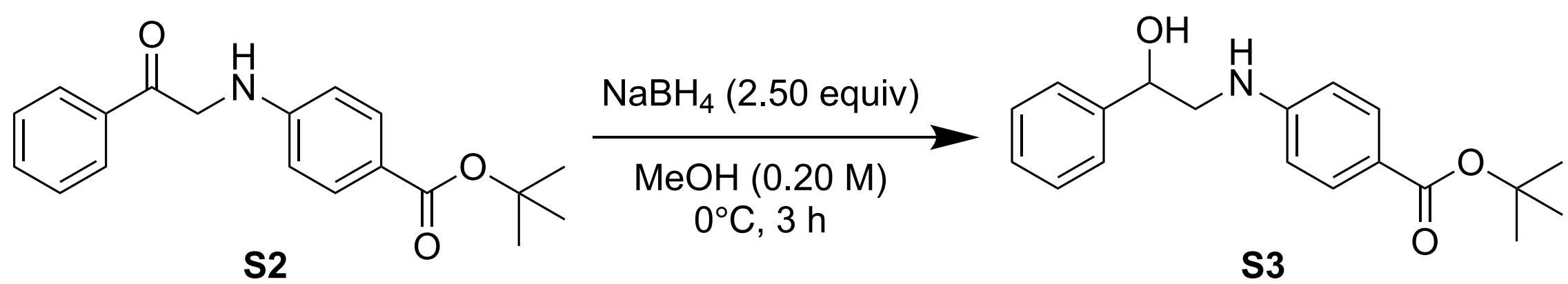

S2 **S3**

To a 20-mL scintillation vial containing a stir bar was added **S2** (621 mg, 1.99 mmol) and methanol (10 mL, 0.20 M). The mixture was allowed to stir until fully dissolved, at which point the solution was cooled to 0 °C in an ice bath for 20 min. Once cooled, sodium borohydride (151 mg, 2.0 equiv, 3.99 mmol) was added in three portions over 1 h. Once all the sodium borohydride has been added, the reaction mixture was allowed to warm to room temperature and stir for 2 h under an atmosphere of air. The reaction was quenched with addition of 2 mL of saturated NH_4Cl, followed by concentration under reduced pressure, furnishing an orange-brown residue. The residue was dissolved in ethyl acetate and transferred to a separatory funnel. The organic layer was washed twice with distilled water and once with brine. The organic layer was collected and dried over MgSO4 before the solvent was removed under reduced pressure. The resultant yellow residue was purified by column chromatography (0-20% ethyl acetate/hexanes) to give **S3** (554 mg, 1.77 mmol, 89% yield) as a colorless foam.

^{1}H NMR (600 MHz, MeOD) δ 7.74 – 7.68 (m, 2H), 7.43 – 7.37 (m, 2H), 7.34 (dd, *J* = 8.4, 6.9 Hz, 2H), 7.29 – 7.23 (m, 1H), 6.62 – 6.55 (m, 2H), 4.86 – 4.83 (m, 1H), 3.40 – 3.32 (m, 2H), 1.55 (s, 9H).

^{13}C NMR (151 MHz, MeOD) δ 168.32, 153.92, 144.20, 132.25, 129.37, 128.60, 127.16, 120.09, 112.32, 81.00, 73.15, 51.82, 28.60.

HRMS (ESI): *m/z* for $C_{19}H_{24}NO_3^+$ $[M+H]^+$ calcd.: 314.1751, found: 314.1742.

Synthesis of 4-((2-hydroxy-2-phenylethyl)amino)benzoic acid (**1a**)

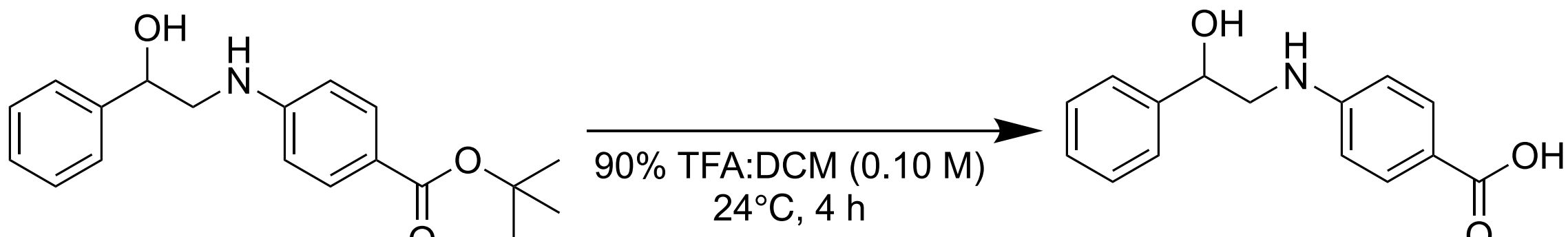

S3 **1a**

Into a 20-mL scintillation vial containing a stir bar was added **S3** (313 mg, 1.00 mmol) and 10 mL of TFA (0.10 M). This solution was allowed to stir at room temperature for 16 h, acquiring a dark red color. The reaction was quenched by removal of the solvent under reduced pressure, leaving a red-brown solid residue. The residue was purified by column chromatography (50-100% ethyl acetate/hexanes) to give **1a** (260 mg, 0.95 mmol, 95% yield) as an off-white solid.

^{1}H NMR (600 MHz, MeOD) δ 7.82 – 7.77 (m, 2H), 7.42 – 7.38 (m, 2H), 7.36 – 7.31 (m, 2H), 7.28 – 7.23 (m, 1H), 6.63 – 6.59 (m, 2H), 4.85 (dd, *J* = 7.6, 5.1 Hz, 2H), 3.41 – 3.33 (m, 2H).

^{13}C NMR (151 MHz, MeOD) δ 170.71, 154.18, 144.12, 132.76, 129.37, 128.61, 127.16, 118.51, 112.37, 73.15, 51.75.

HRMS (ESI): *m/z* for $C_{15}H_{16}NO_3^+$ $[M+H]^+$ calcd.: 258.1125, found: 258.1129.

Synthesis of *tert*-butyl 4-(2-phenylaziridin-1-yl)benzoate (**S4**)

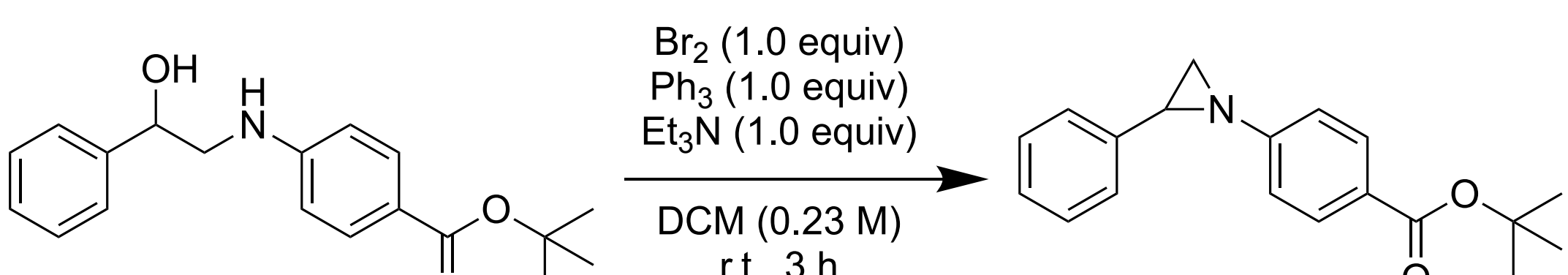

S3 **S4**

The procedure followed was a modified version of that in reference 56. Into a flame-dried, 50-mL round bottom flask containing a stir bar under an atmosphere of nitrogen was added triphenylphosphine (837 mg, 3.19 mmol, 1.0 equiv). The triphenylphosphine was dissolved in 14

mL of dichloromethane (0.23 M). Bromine (164 μL, 3.19 mmol, 1.0 equiv) was added to this solution over the course of 5 min, initially turning the mixture red before fading to yellow. This reaction mixture was allowed to stir at room temperature for 30 min. **S3** (1.00 g, 3.19 mmol) in 2 mL of dichloromethane was added to the reaction mixture over the course of 5 min. Once the solution of **S3** was fully added, triethylamine (445 μL, 3.19 mmol, 1.0 equiv) was added to the reaction mixture via syringe. The reaction mixture was allowed to stir for 3 h under an atmosphere of nitrogen. The reaction mixture was quenched by addition of saturated NH_4Cl and was transferred to a separatory funnel. The aqueous phase was removed, and the organic layer was washed sequentially with water and brine. The organic layer was collected, dried over $MgSO_4$, and concentrated under reduced pressure to give a light brown residue which was purified by column chromatography (0-30% ethyl acetate/hexanes) to give **S4** (443 mg, 1.50 mmol, 47% yield) as a colorless oil. **S4** was estimated to be approximately 90% pure based on ^{1}H-NMR. These impurities did not affect subsequent steps.

^{1}H NMR (500 MHz, $CDCl_3$) δ 7.92 – 7.77 (m, 2H), 7.43 – 7.32 (m, 5H), 6.63 – 6.48 (m, 2H), 5.11 (t, *J* = 7.2 Hz, 1H), 3.89 (dd, *J* = 14.5, 7.8 Hz, 1H), 3.77 (dd, *J* = 14.4, 6.6 Hz, 1H), 1.57 (s, 9H).

^{13}C NMR (126 MHz, $CDCl_3$) δ 166.04, 150.24, 139.26, 131.60, 129.14 (d, *J* = 4.1 Hz), 127.80, 121.66, 112.09, 80.20, 52.73, 51.03, 28.44.

HRMS (ESI): *m/z* for $C_{19}H_{21}NNaO_2^+$ $[M+Na]^+$ calcd.: 318.146418, found: 318.1477.

Synthesis of di-*tert*-butyl 4,4'-((1-phenylethane-1,2-diyl)bis(azanediyl))dibenzoate (**S5**)

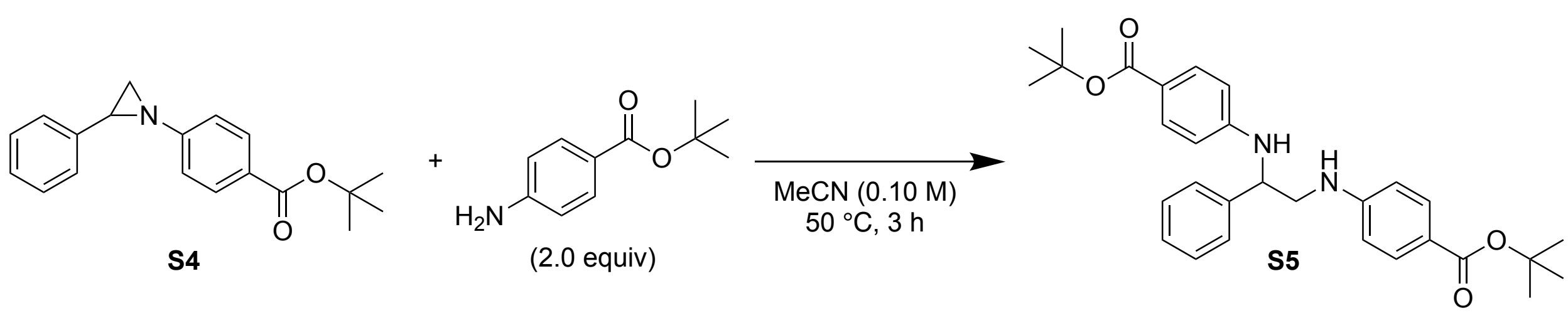

Into a 20-mL scintillation vial containing a stir bar was added **S4** (0.443 g, 1.50 mmol) and tert-butyl 4-aminobenzoate (580 mg, 3.00 mmol, 2.0 equiv). This mixture was dissolved in 15 mL of acetonitrile (0.10 M) and was allowed to stir at 50 °C for 3 h, after which time the reaction was quenched by evaporation of solvent under reduced pressure. The light brown oil was triturated with hexanes and recrystallized from hot dichloromethane/hexanes to give **S5** (261 mg, 0.524 mmol, 36% yield) as a white, powdery solid.

^{1}H NMR (500 MHz, $CDCl_3$) δ 7.84 (d, *J* = 8.7 Hz, 2H), 7.80 – 7.69 (m, 2H), 7.41 – 7.33 (m, 4H), 7.30 (td, *J* = 5.9, 2.5 Hz, 1H), 6.63 (d, *J* = 8.5 Hz, 2H), 6.52 (d, *J* = 8.7 Hz, 2H), 4.75 – 4.68 (m, 1H), 3.60 (dd, *J* = 13.0, 5.0 Hz, 1H), 3.52 (dd, *J* = 13.0, 7.7 Hz, 1H), 1.57 (dd, *J* = 2.7, 1.1 Hz, 9H), 1.56 – 1.51 (m, 9H).

^{13}C NMR (151 MHz, $CDCl_3$) δ 166.04, 166.02, 150.77, 150.14, 139.99, 131.59, 131.36, 129.27, 128.22, 126.54, 121.97, 121.64, 112.85, 112.35, 80.29, 80.13, 57.03, 50.21, 28.46, 28.43.

HRMS (ESI): *m/z* for $C_{30}H_{37}N_2O_4^+$ $[M+H]^+$ calcd.: 489.2748, found: 489.2727.

Synthesis of 4,4'-((1-phenylethane-1,2-diyl)bis(azanediyl))dibenzoic acid (**2a**)

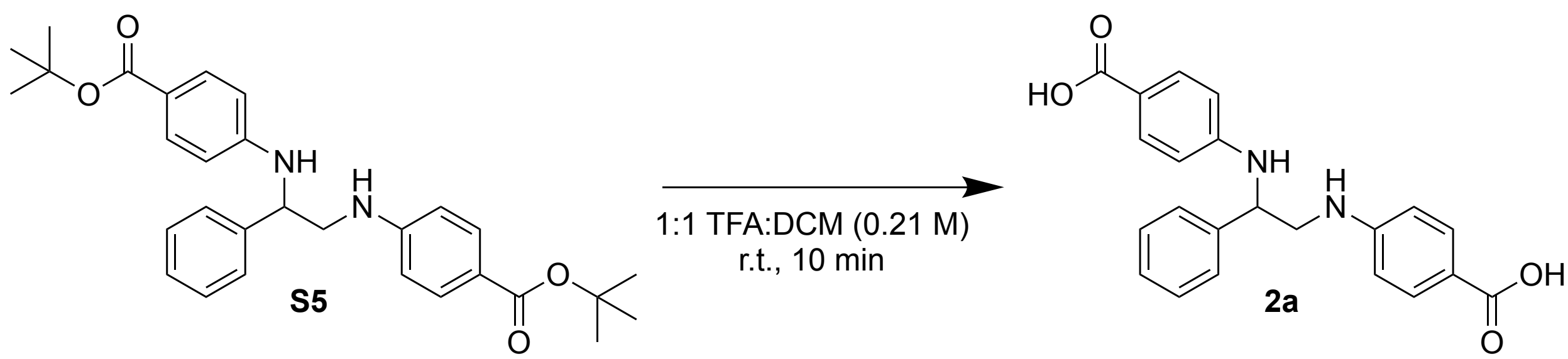

In a 20-mL scintillation vial containing a stir bar, **S5** (100 mg, 0.205 mmol) was dissolved in 10 mL of 50% trifluoroacetic acid in dichloromethane (0.21 M). The solution became dark red and fumed. This solution was allowed to stir at room temperature for 10 min, at which time the reaction was quenched by evaporation of solvent under reduced pressure. The dark-red residue was dissolved in diH_2O, forming a colorless solution. Glacial acetic acid was then added dropwise with stirring, causing precipitation. When the addition of more glacial acetic acid yielded no more solid, the solid was collected by filtration, washed with minimal cold water, and dried under vacuum to give **2a** (64.8 mg, 0.172 mmol, 84% yield) as a cream-colored solid.

^{1}H NMR (500 MHz, DMSO) δ 7.68 (d, *J* = 8.4 Hz, 2H), 7.59 (d, *J* = 8.4 Hz, 2H), 7.47 – 7.42 (m, 2H), 7.33 (t, *J* = 7.6 Hz, 2H), 7.23 (t, *J* = 7.4 Hz, 1H), 7.07 (d, *J* = 7.3 Hz, 1H), 6.69 – 6.58 (m, 3H), 6.54 (d, *J* = 8.5 Hz, 2H), 4.68 (td, *J* = 7.9, 5.1 Hz, 1H), 3.40 (ddd, *J* = 21.5, 11.0, 6.2 Hz, 2H).

^{13}C NMR (126 MHz, DMSO) δ 167.73, 167.65, 151.88, 151.59, 141.91, 131.15, 130.87, 128.48, 127.17, 126.71, 118.18, 118.00, 111.79, 111.06, 55.66, 49.22.

HRMS (ESI): *m/z* for $C_{22}H_{20}N_2NaO_4^+$ $[M+Na]^+$ calcd.: 399.1315, found: 399.1318.

Synthesis of 2-bromo-1-(3,4-dimethoxyphenyl)ethan-1-one (**S6**)

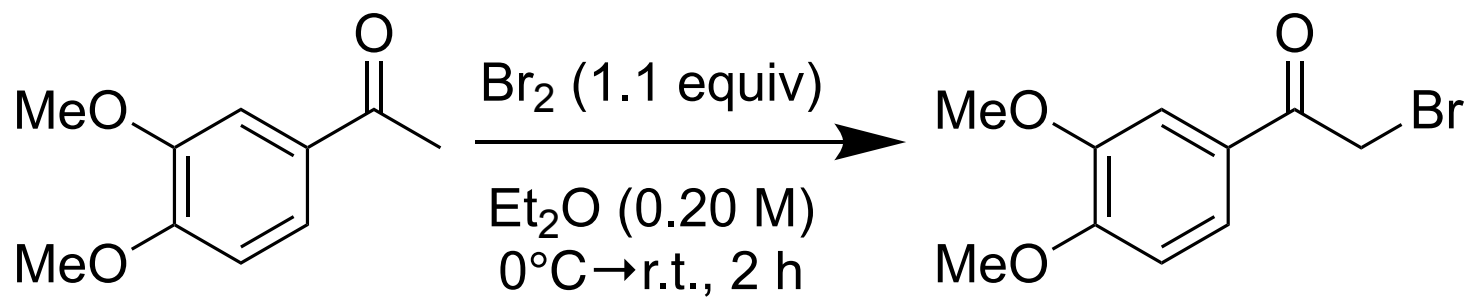

S6

Into a 500-mL beaker containing a magnetic stir bar was added 3,4-dimethoxyacetophenone (7.21 g, 40.0 mmol) and 200 mL of diethyl ether (0.20 M). This mixture was allowed to stir until fully dissolved before the reaction mixture was placed in an ice bath and allowed to cool to 0°C over 15 min. Once cooled, bromine (6.32 g, 40.0 mmol, 1.0 equiv) was added dropwise over 10 min. The solution turned red-orange and slowly emitted a vapor. Once all the bromine was added, the reaction mixture was removed from the ice bath and allowed to warm to room temperature over the course of 1 h. During this time, an orange precipitate, which slowly redissolved into the reaction solution, formed. Once the solid had redissolved, the reaction was quenched with addition of distilled water. This mixture was allowed to stir for 20 min before being transferred into a separatory funnel. The aqueous layer was removed. The ether layer was washed sequentially with distilled water, saturated bicarbonate solution, and brine, and was then dried over $MgSO_4$. The solution was concentrated under reduced pressure to give a light purple oil. This oil was triturated with hexanes and recrystallized from hot ether/hexanes to give **S6** (7.45 g, 28.8 mmol, 72% yield) as a fine, white, crystalline solid which slowly turned light purple over the following days. There was no change in the NMR spectra of the compound after this change in color, but the compound was stored at -20°C away from light to mitigate degradation.

^{1}H NMR (600 MHz, $CDCl_3$) δ 7.60 (dd, J = 8.4, 2.1 Hz, 1H), 7.53 (d, J = 2.1 Hz, 1H), 6.90 (d, J = 8.4 Hz, 1H), 4.40 (s, 2H), 3.95 (s, 3H), 3.93 (s, 3H).

^{13}C NMR (151 MHz, $CDCl_3$) δ 190.19, 154.17, 149.46, 127.20, 123.99, 111.00, 110.24, 56.27, 56.17, 30.48.

HRMS (ESI): m/z for $C_{10}H_{12}BrO_3^+$ $[M+H]^+$ calcd.: 258.9964, found: 258.9955.

Synthesis of *tert*-butyl 4-((2-(3,4-dimethoxyphenyl)-2-oxoethyl)amino)benzoate (**S7**)

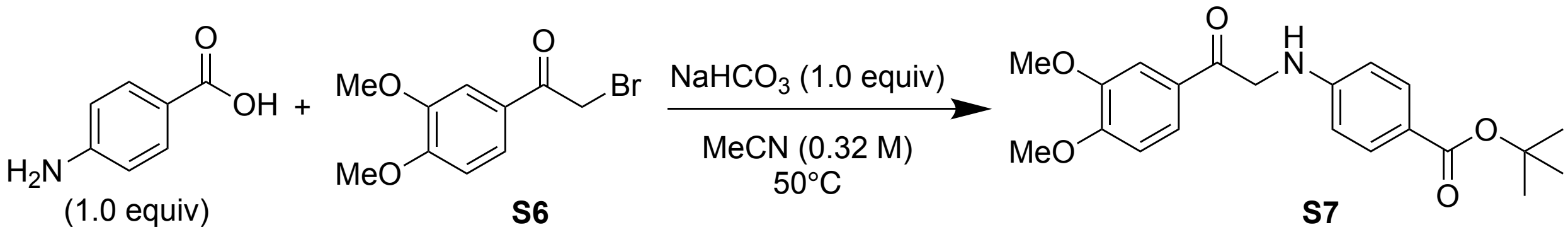

(1.0 equiv) **S6** **S7**

Into a 100-mL round bottom flask containing a magnetic stir bar was added **S6** (5.00 g, 19.3 mmol), tert-butyl 4-aminobenzoate (4.10 g, 21.2 mmol, 1.1 equiv), and sodium bicarbonate (1.62 g, 19.3 mmol, 1.0 equiv). To this mixture was added 60 mL of acetonitrile (0.32 M), and the reaction mixture was heated at 50 °C in an oil bath and stirred overnight under an atmosphere of air. After stirring overnight, the reaction appeared brown in color with a white solid. The reaction mixture was allowed to cool to room temperature, during which time a white solid began to crystallize from solution. The solid was collected by filtration through a medium glass frit, washed with water to remove excess sodium bicarbonate, and purified by recrystallization from hot acetonitrile/water to give **S7** (5.31 g, 14.3 mmol, 74% yield) as a white, crystalline solid.

^{1}H NMR (600 MHz, DMSO) δ 7.77 (dd, *J* = 8.4, 2.0 Hz, 1H), 7.67 – 7.61 (m, 2H), 7.53 (d, *J* = 2.0 Hz, 1H), 7.10 (d, *J* = 8.5 Hz, 1H), 6.72 – 6.66 (m, 2H), 6.61 (t, *J* = 5.5 Hz, 1H), 4.71 (d, *J* = 5.5 Hz, 2H), 3.86 (s, 3H), 3.84 (s, 3H), 1.50 (s, 9H).

^{13}C NMR (151 MHz, DMSO) δ 194.32, 165.27, 153.44, 152.05, 148.67, 130.64, 127.82, 122.56, 118.26, 111.44, 111.01, 110.29, 79.02, 55.80, 55.62, 48.95, 27.99.

HRMS (ESI): *m/z* for $C_{21}H_{26}NO_5^+$ $[M+H]^+$ calcd.: 372.1805, found: 372.1802.

Synthesis of tert-butyl 4-((2-(3,4-dimethoxyphenyl)-2-hydroxyethyl)amino)benzoate (**S8**)

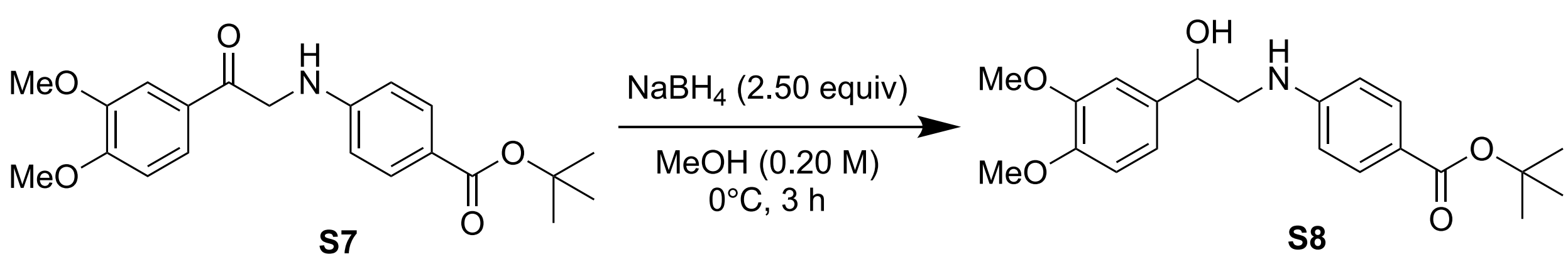

Into a 20-mL scintillation vial containing a stir bar was added **S7** (900 mg, 2.42 mmol) and methanol (12 mL, 0.20 M). The mixture was allowed to stir until fully dissolved, at which point the solution was cooled to 0 °C in an ice bath for 20 min. Once cooled, sodium borohydride (229 mg, 6.06 mmol, 2.5 equiv), was added in three portions over 1 h. Once all the sodium borohydride had been added, the reaction mixture was allowed to warm to room temperature and was stirred for 2 h under an atmosphere of air. The reaction was quenched with addition of saturated NH_4Cl, followed by concentration under reduced pressure. This residue was dissolved in ethyl acetate and transferred to a separatory funnel. The organic layer was washed twice with distilled water and once with brine, sequentially. The organic layer was collected and dried over $MgSO_4$, before the solvent was removed under reduced pressure. The resultant yellow residue was purified by column

chromatography (0-50% ethyl acetate/hexanes) to give **S8** (819 mg, 2.19 mmol, 90% yield) as an off-white foam.

^{1}H NMR (600 MHz, DMSO) δ 7.65 – 7.58 (m, 2H), 6.99 (d, *J* = 1.3 Hz, 1H), 6.90 (d, *J* = 1.1 Hz, 2H), 6.66 – 6.59 (m, 2H), 6.38 (t, *J* = 5.8 Hz, 1H), 5.42 (d, *J* = 4.4 Hz, 1H), 4.67 (dt, *J* = 7.6, 4.7 Hz, 1H), 3.74 (d, *J* = 11.7 Hz, 6H), 3.29 – 3.14 (m, 2H), 1.49 (s, 9H).

^{13}C NMR (151 MHz, DMSO) δ 165.30, 152.46, 148.54, 147.91, 136.44, 130.76, 118.07, 117.59, 111.55, 110.93, 109.94, 78.90, 70.50, 55.58, 55.43, 50.70, 28.01.

HRMS (ESI): *m/z* for $C_{21}H_{28}NO_5^+$ $[M+H]^+$ calcd.: 374.1962, found: 374.1965.

Synthesis of 4-((2-(3,4-dimethoxyphenyl)-2-hydroxyethyl)amino)benzoic acid (**1b**)

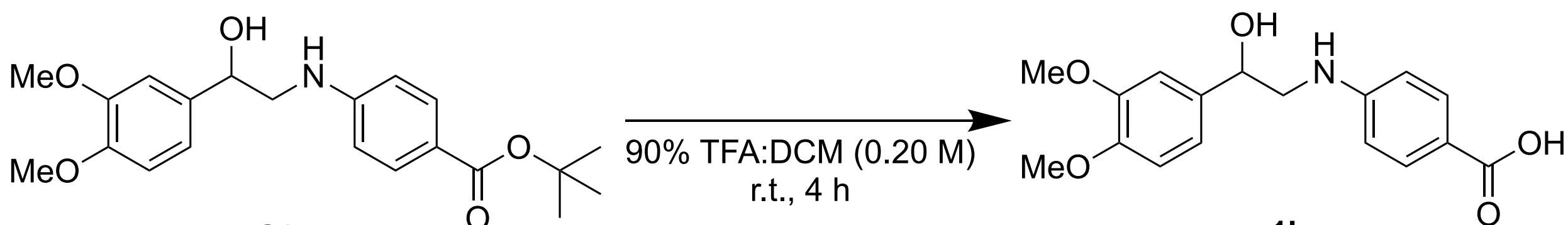

Into a 20-mL scintillation vial containing a stir bar was added **S8** (0.819 g, 2.19 mmol) and 11 mL of 90% trifluoroacetic acid in dichloromethane (0.20 M), causing the solution to fume slightly and become light yellow brown in color. The reaction was capped under air and was stirred at room temperature for 4 h. Once complete, the reaction was quenched by evaporation of solvent under reduced pressure, leaving a dark-red residue, which was purified by reverse-phase chromatography (0-30% Acetonitrile/Water with 0.1% formic acid) to give **1b** (604 mg, 1.91 mmol, 87% yield) as a light green, crystalline solid.

^{1}H NMR (500 MHz, DMSO) δ 11.79 (s, 1H), 7.72 – 7.58 (m, 2H), 6.98 (s, 1H), 6.89 (s, 2H), 6.67 – 6.55 (m, 2H), 6.36 (t, *J* = 5.9 Hz, 1H), 5.55 – 5.39 (m, 1H), 4.67 (t, *J* = 6.4 Hz, 1H), 3.73 (d, *J* = 8.9 Hz, 6H), 3.27 – 3.13 (m, 3H).

^{13}C NMR (126 MHz, DMSO) δ 167.72, 152.68, 148.65, 148.04, 136.50, 131.27, 118.25, 116.92, 111.64, 111.15, 110.02, 70.69, 55.70, 55.56, 50.78.

HRMS (ESI): *m/z* for $C_{17}H_{20}NO_5^+$ $[M+H]^+$ calcd.: 318.1336, found: 318.1344.

Synthesis of *tert*-butyl 4-(*N*-(2-(3,4-dimethoxyphenyl)-2-oxoethyl)acetamido)benzoate (**S9**)

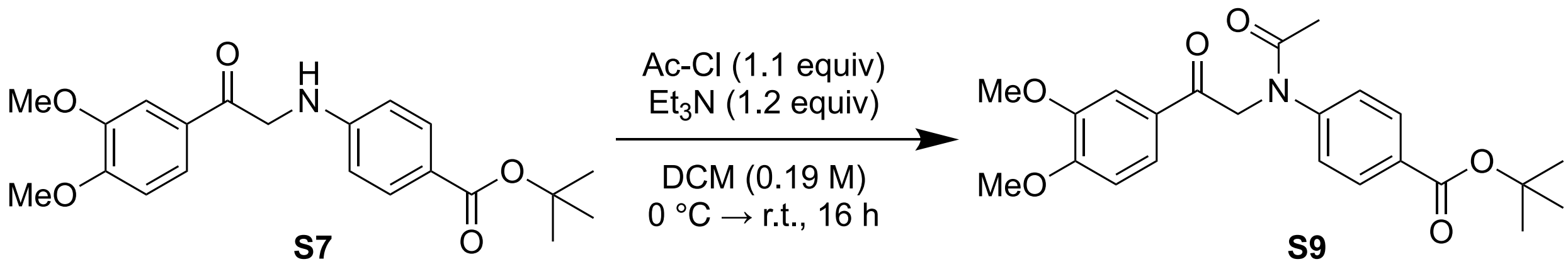

Into a 20-mL scintillation vial containing a stir bar, **S7** (500 mg, 1.35 mmol) was dissolved in 7 mL of dichloromethane (0.19 M), giving a colorless solution. Once dissolved, the solution was cooled in an ice bath for 15 min. To the cooled solution was added acetyl chloride (105 μL, 1.48 mmol, 1.1 equiv), followed by slow addition of triethylamine (225 μL, 1.62 mmol, 1.2 equiv) over 5 min. The reaction turned light yellow and formed a colorless solid. The reaction mixture was allowed to warm to room temperature while stirring for 16 h. Once complete, the reaction was quenched with addition of saturated sodium bicarbonate, after which time the reaction mixture was transferred to a separatory funnel. The layers were separated, and the aqueous layer was extracted with dichloromethane two more times. The organic layers were combined and sequentially washed with diH_2O, saturated NH_4Cl, and brine. The organic layer was dried over $MgSO_4$ and concentrated to give **S9** (552 mg, 1.33 mmol, 99% yield) as a colorless solid that was used without further purification.

^{1}H NMR (600 MHz, DMSO) δ 7.20 (d, J = 8.1 Hz, 2H), 6.86 (dt, J = 8.4, 1.8 Hz, 1H), 6.79 – 6.63 (m, 3H), 6.21 (dd, J = 8.5, 1.5 Hz, 1H), 4.35 (s, 2H), 3.08 (d, J = 1.5 Hz, 3H), 3.05 (d, J = 1.6 Hz, 3H), 1.17 (s, 3H), 0.78 (d, J = 1.5 Hz, 9H).

^{13}C NMR (151 MHz, DMSO) δ 191.59, 170.63, 164.12, 153.32, 148.40, 146.36, 130.63, 129.58, 127.17, 126.80, 121.83, 109.64, 109.39, 80.50, 54.85, 54.31, 54.25, 26.16, 20.10.

HRMS (ESI): *m/z* for $C_{23}H_{28}NO_6^+$ $[M+H]^+$ calcd.: 414.1911, found: 414.1895.

Synthesis *of tert*-butyl 4-(*N*-(2-(3,4-dimethoxyphenyl)-2-hydroxyethyl)acetamido)benzoate (**S10**)

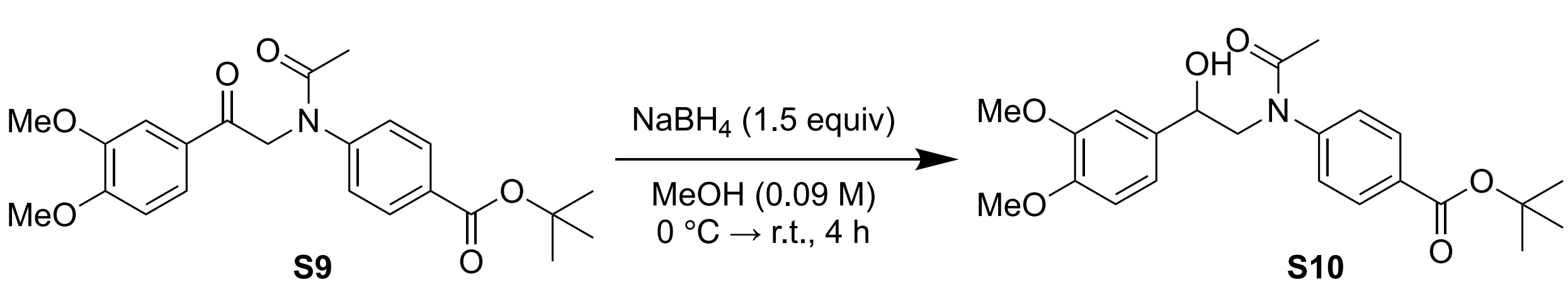

Into a 20-mL scintillation vial containing a stir bar was added **S9** (300 mg, 0.726 mmol) and methanol (8 mL, 0.09 M) to form a colorless solution. Once dissolved, the reaction mixture was cooled in an ice bath for 15 min, before sodium borohydride (42 mg, 1.1 mmol, 1.5 equiv) was added in three portions over the course of an hour. After the final addition of $NaBH_4$, the reaction was allowed to warm to room temperature, over 4 h. After completion, the reaction was quenched with the slow addition of saturated NH_4Cl. The reaction was then transferred to a separatory funnel, diluted with diH_2O, and the aqueous layer was extracted four times with ethyl acetate. The organic layers were combined and washed sequentially with water and brine. The organic layer was collected, dried over $MgSO_4$, and concentrated under reduced pressure to give a gummy, colorless residue. This residue was purified by column chromatography (0-50% ethyl acetate/hexanes) to give **S10** (138 mg, 0.332 mmol, 46% yield) as a colorless solid.

^{1}H NMR (500 MHz, DMSO) δ 7.89 (d, *J* = 8.2 Hz, 2H), 7.37 (d, *J* = 8.2 Hz, 2H), 6.90 – 6.81 (m, 2H), 6.77 (dd, *J* = 8.2, 2.0 Hz, 1H), 5.37 (s, 1H), 4.66 (s, 1H), 3.76 (d, *J* = 5.7 Hz, 2H), 3.70 (d, *J* = 11.3 Hz, 6H), 1.80 (s, 3H), 1.55 (s, 9H).

^{13}C NMR (126 MHz, DMSO) δ 169.24, 164.40, 148.58, 148.04, 147.63, 135.78, 129.91, 129.70, 128.04, 118.31, 111.59, 109.82, 80.88, 70.02, 56.73, 55.59, 55.40, 27.77, 22.78.

HRMS (ESI): *m/z* for $C_{23}H_{30}NO_6^+$ $[M+H]^+$ calcd.: 416.2068, found: 416.2050.

Synthesis of 4-(*N*-(2-(3,4-dimethoxyphenyl)-2-hydroxyethyl)acetamido)benzoic acid (**3b**)

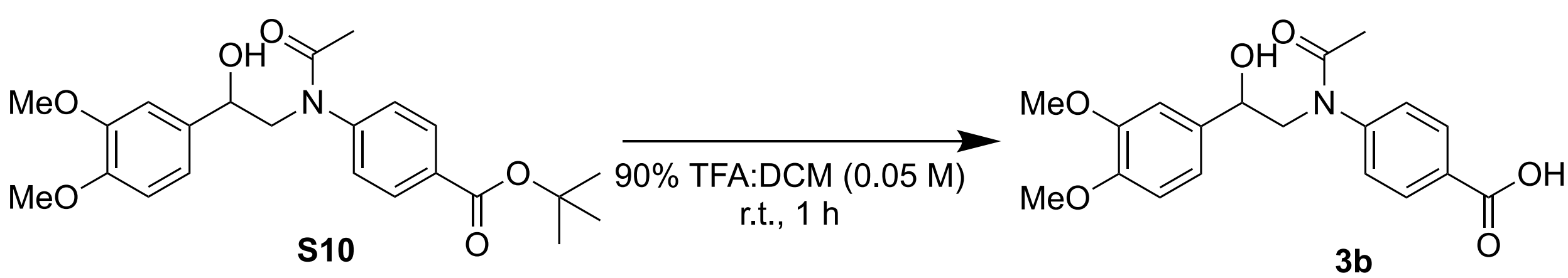

Into a 20-mL scintillation vial containing a stir bar, **S10** (100 mg, 0.241 mmol) was dissolved in 5 mL of 90% trifluoroacetic acid in dichloromethane (0.05 M), causing the solution turn a dull brown color. The reaction vessel was capped under an atmosphere of air, and the reaction was allowed to stir at room temperature for 1 h, after which time the reaction was quenched by evaporation of solvent under reduced pressure to give a dark brown residue. The residue was purified by column chromatography (30-70% ethyl acetate/hexanes) to give **4** (57.1 mg, 0.161 mmol, 67% yield) as a brown solid.

^{1}H NMR (500 MHz, CD_3CN) δ 7.78 – 7.71 (m, 2H), 6.97 – 6.87 (m, 3H), 6.68 – 6.60 (m, 2H), 5.77 (dd, *J* = 7.9, 4.8 Hz, 1H), 3.78 (s, 3H), 3.77 (s, 3H), 3.55 (dd, *J* = 14.2, 7.9 Hz, 1H), 3.46 (dd, *J* = 14.2, 4.8 Hz, 1H), 2.01 (s, 3H).
^{13}C NMR (126 MHz, CD_3CN) δ 171.87, 169.29, 153.43, 150.02, 149.99, 132.57, 132.04, 120.08, 118.50, 112.50, 112.42, 111.21, 75.10, 56.44, 56.42, 48.43, 21.40.
HRMS (ESI): *m/z* for $C_{19}H_{22}NO_6^+$ $[M+H]^+$ calcd.: 360.1442, found: 360.1435.

Synthesis of 2-bromo-1-(3-methoxyphenyl)ethan-1-one (**S11**)

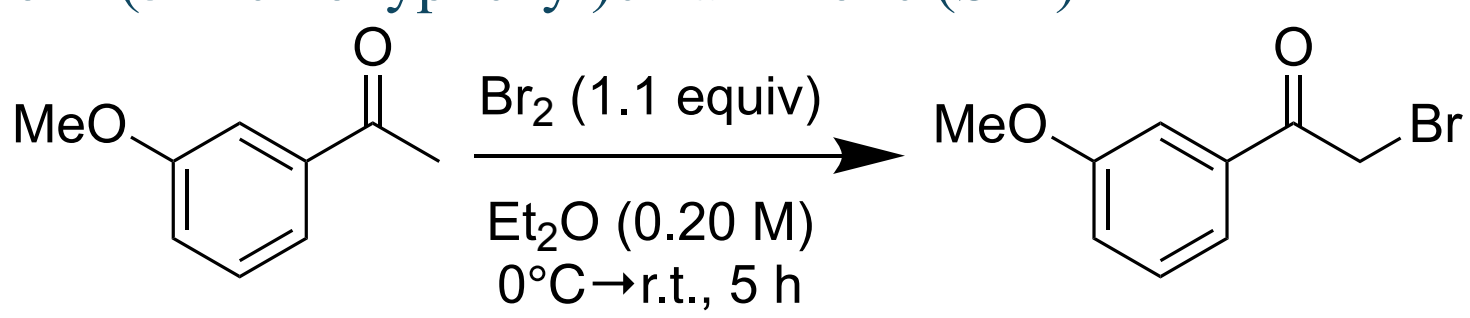

S11

Into a 500-mL beaker containing a magnetic stir bar was added 3-methoxyacetophenone (6.00 g, 40.0 mmol) and 200 mL of diethyl ether (0.20 M). This mixture was allowed to stir until fully dissolved before the reaction mixture was placed in an ice bath and allowed to cool to 0 °C over 15 min. Once cooled, bromine (6.70 g, 41.9 mmol, 1.05 equiv) was added dropwise over 10 min. The solution turned red-orange in color and slowly emitted a vapor. Once all the bromine was added, the reaction mixture was removed from the ice bath and allowed to warm to room temperature over the course of 1 h. During this time, an orange precipitate, which slowly redissolved into the reaction solution, formed. The reaction mixture was allowed to continue stirring for a total of 5 hours at room temperature before distilled water was added, forming a biphasic mixture. This mixture was allowed to stir for 20 min before being transferred into a separatory funnel. The aqueous layer was removed. The ether layer was washed sequentially with distilled water, saturated bicarbonate solution, and brine, and was then dried over $MgSO_4$. The solution was concentrated under reduced pressure to give a yellow oil. This oil was triturated with hexanes and recrystallized from hot ether/hexanes to give **S6** (5.23 g, 22.8 mmol, 57% yield) as fine, colorless plates.

^{1}H NMR (500 MHz, $CDCl_3$) δ 7.55 (ddd, *J* = 7.6, 1.8, 1.1 Hz, 1H), 7.50 (dd, *J* = 2.7, 1.7 Hz, 1H), 7.39 (t, *J* = 7.9 Hz, 1H), 7.15 (ddd, *J* = 8.4, 2.7, 1.1 Hz, 1H), 4.44 (s, 2H), 3.86 (s, 3H).
^{13}C NMR (126 MHz, $CDCl_3$) δ 191.35, 160.21, 135.50, 130.05, 121.72, 120.74, 113.31, 55.73, 31.22.

HRMS (ESI): *m/z* for $C_9H_{10}BrO_2^+$ $[M+H]^+$ calcd.: 228.9859, found: 228.9859

Synthesis of *tert*-butyl 4-((2-(3-methoxyphenyl)-2-oxoethyl)amino)benzoate (**S12**)

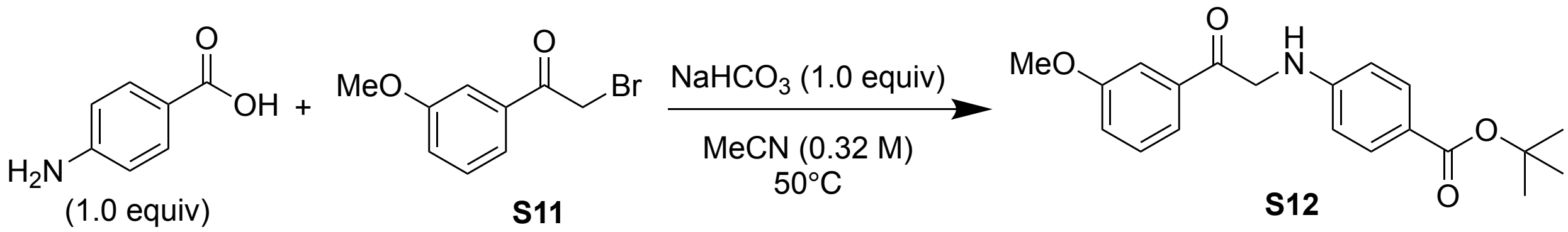

Into a 100-mL round bottom flask containing a magnetic stir bar was added **S12** (4.00 g, 17.5 mmol), tert-butyl 4-aminobenzoate (3.37 g, 17.5 mmol, 1.0 equiv), and sodium bicarbonate (1.47 g, 17.5 mmol, 1.0 equiv). To this mixture was added 55 mL of acetonitrile (0.32 M), and the reaction mixture was heated at 50 °C in an oil bath and stirred overnight under an atmosphere of air. After stirring overnight, the reaction appeared yellow-brown in color with a white solid. The reaction mixture was hot-filtered through a fine glass frit to remove the sodium bicarbonate, and the solvent was removed under reduced pressure to give a brown residue. This residue was recrystallized from boiling acetone/water to give **S13** (4.35 g, 12.7 mmol, 73% yield) as yellow, needle-like crystals.

^{1}H NMR (500 MHz, DMSO) δ 7.66 (dt, *J* = 7.8, 1.2 Hz, 1H), 7.65 – 7.61 (m, 2H), 7.54 (dd, *J* = 2.7, 1.5 Hz, 1H), 7.48 (t, *J* = 7.9 Hz, 1H), 7.26 (ddd, *J* = 8.2, 2.7, 1.0 Hz, 1H), 6.71 – 6.67 (m, 2H), 6.65 (t, *J* = 5.6 Hz, 1H, NH), 4.76 (d, *J* = 5.5 Hz, 2H), 3.84 (s, 3H), 1.49 (s, 9H).
^{13}C NMR (126 MHz, DMSO) δ 195.92, 165.29, 159.50, 152.05, 136.38, 130.67, 130.04, 120.35, 119.59, 118.33, 112.59, 111.47, 79.07, 55.44, 49.50, 28.02.
HRMS (ESI): *m/z* for $C_{20}H_{24}NO_4^+$ $[M+H]^+$ calcd.: 342.1700, found: 342.1698

Synthesis of 4-((2-(3-methoxyphenyl)-2-oxoethyl)amino)benzoic acid (**S13**)

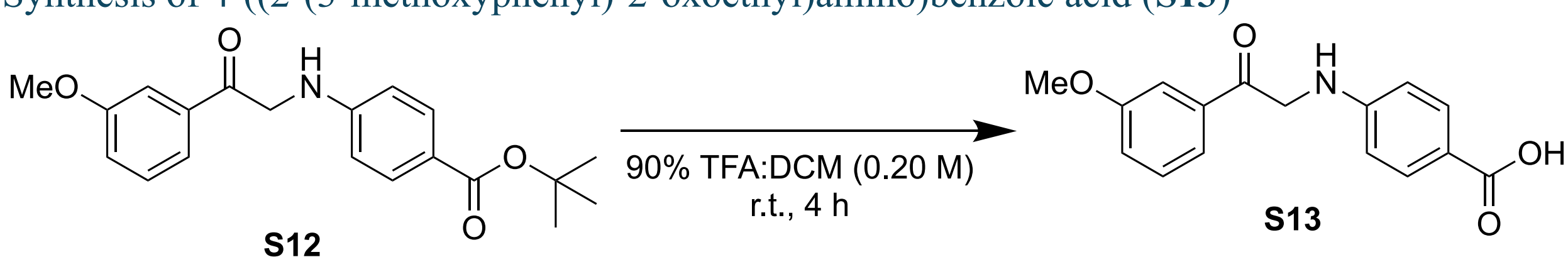

Into a 250-mL round bottom flask was added **S12** (4.00 g, 11.7 mmol), and the solid was dissolved in 100 mL of 90% trifluoroacetic acid in dichloromethane (0.12 M). Upon addition of the trifluoroacetic acid solution, the solution became noticeably warm, evolved a small amount of gas,

and became light-yellow in color. The reaction was lightly capped under an atmosphere of air and allowed to stir at room temperature for 4 h. After this time, the solvent was removed under reduced pressure to give a light-brown residue which was purified by recrystallization from boiling acetone/water to give **S13** (2.76 g, 9.67 mmol, 83% yield) as a colorless, crystalline solid.

^{1}H NMR (500 MHz, DMSO) δ 12.06 (s, 1H, COOH), 7.71 – 7.63 (m, 3H), 7.55 (dd, *J* = 2.7, 1.5 Hz, 1H), 7.48 (t, *J* = 7.9 Hz, 1H), 7.25 (ddd, *J* = 8.2, 2.7, 1.0 Hz, 1H), 6.73 – 6.68 (m, 2H), 6.66 (t, *J* = 5.5 Hz, 1H, NH), 4.77 (d, *J* = 5.5 Hz, 2H), 3.84 (s, 3H).
^{13}C NMR (126 MHz, CD_3CN) δ 195.93, 167.51, 159.50, 152.09, 136.36, 131.00, 130.03, 120.36, 119.62, 117.57, 112.59, 111.53, 55.44, 49.52.
HRMS (ESI): *m/z* for $C_{16}H_{16}NO_4^+$ $[M+H]^+$ calcd.: 286.1074, found: 286.1073

Synthesis of 4-((2-hydroxy-2-(3-methoxyphenyl)ethyl)amino)benzoic acid (**1c**)

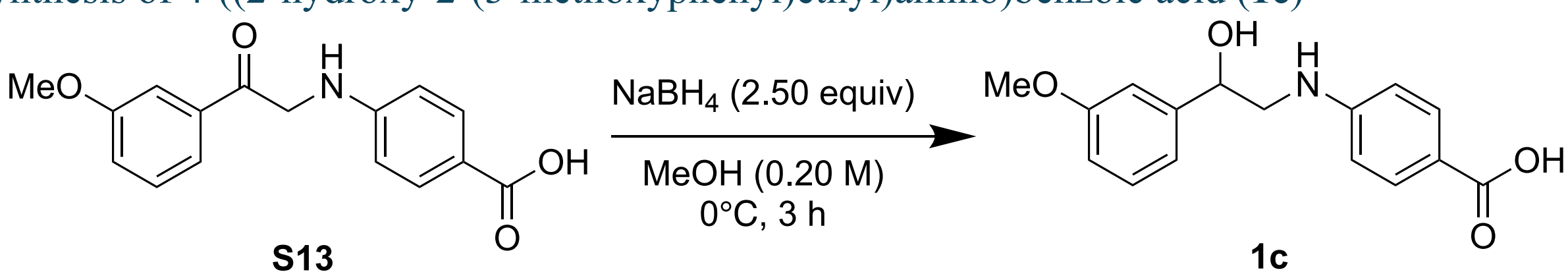

Into a 250-mL round bottom flask with a stir bar was added **S13** (2.50 g, 8.76 mmol). The solid was suspended in 44 mL of methanol (0.20 M) and was stirred until dispersed in the solution. This suspension was cooled in an ice bath for 15 min before sodium borohydride (497 mg, 13.1 mmol, 1.50 equiv) was added in three portions over the course of 30 min. After addition of $NaBH_4$, the reaction was allowed to warm to room temperature for 3 h with stirring under air. Once complete, the reaction was quenched with slow addition of saturated NH_4Cl. The solvent was removed under reduced vacuum, leaving a colorless, heterogenous residue. This residue was recrystallized from boiling water/acetone to give **1c** (1.69 g, 5.88 mmol, 67% yield) as a colorless solid.

^{1}H NMR (500 MHz, DMSO) δ 11.98 (s, 1H, COOH), 7.70 – 7.61 (m, 2H), 7.25 (t, *J* = 8.0 Hz, 1H), 6.97 (dt, *J* = 4.0, 1.8 Hz, 2H), 6.82 (ddd, *J* = 8.2, 2.6, 1.1 Hz, 1H), 6.66 – 6.60 (m, 2H), 6.44 (t, *J* = 5.9 Hz, 1H, NH), 5.52 (s, 1H, OH), 4.75 – 4.65 (m, 1H), 3.75 (s, 3H), 3.30 – 3.15 (m, 2H).
^{13}C NMR (126 MHz, DMSO) δ 167.53, 159.17, 152.50, 145.63, 131.12, 129.11, 118.27, 116.88, 112.51, 111.60, 111.03, 70.65, 54.97, 50.64.
HRMS (ESI): *m/z* for $C_{16}H_{18}NO_4^+$ $[M+H]^+$ calcd.: 288.1230, found: 288.1228

Synthesis of *tert*-butyl 4-((2-(4-hydroxyphenyl)-2-oxoethyl)amino)benzoatebenzoate (**S14**)

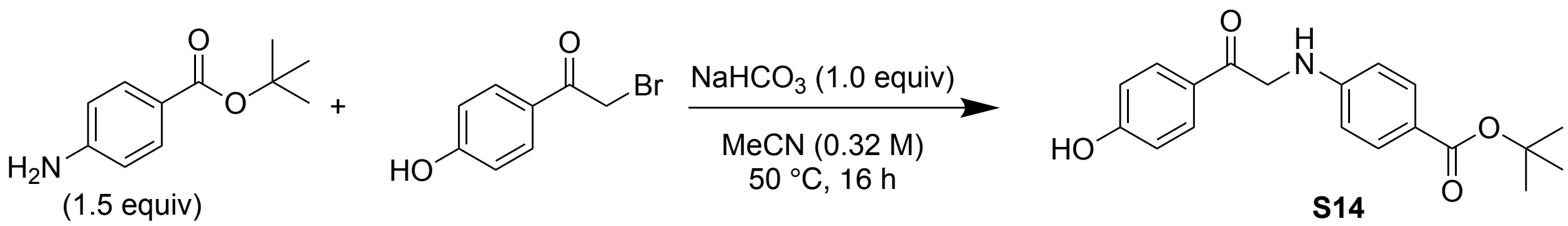

Into a 100-mL round bottom flask with stir bar was added 2-bromo-1-(4-hydroxyphenyl)ethan-1-one (3.42 g, 15.9 mmol), tert-butyl 4-aminobenzoic acid (4.60 g, 23.8 mmol, 1.5 equiv), and sodium bicarbonate (1.33 g, 15.9 mmol, 1.0 equiv). To this mixture was added 50 mL of acetonitrile (0.32 M), and the mixture was heated at 50 °C in an oil bath and stirred overnight under an atmosphere of air. After stirring overnight, the reaction appeared pale red-brown , and a white precipitate formed. The reaction mixture was allowed to cool to room temperature, and the excess salt was filtered through a medium glass frit. The filtrate was collected and concentrated under reduced pressure to give a light-brown solid. This solid was recrystallized from hot acetonitrile/diH_2O to give **S14** (3.69 g, 11.7 mmol, 73% yield) as a yellow powder.

^{1}H NMR (600 MHz, DMSO) δ 10.43 (s, 1H), 8.08 – 7.86 (m, 2H), 7.73 – 7.55 (m, 2H), 6.97 – 6.81 (m, 2H), 6.74 – 6.63 (m, 2H), 6.57 (t, *J* = 5.5 Hz, 1H), 4.65 (d, *J* = 5.4 Hz, 2H), 1.50 (s, 9H).

^{13}C NMR (151 MHz, DMSO) δ 193.79, 165.26, 162.44, 152.03, 130.63, 130.46, 126.50, 118.22, 115.32, 111.41, 78.99, 48.78, 27.99.

HRMS (ESI): *m/z* for $C_{19}H_{22}NO_4^+$ $[M+H]^+$ calcd.: 328.1543, found: 328.1554.

Synthesis of 4-((2-(4-hydroxyphenyl)-2-oxoethyl)amino)benzoic acid (**S15**)

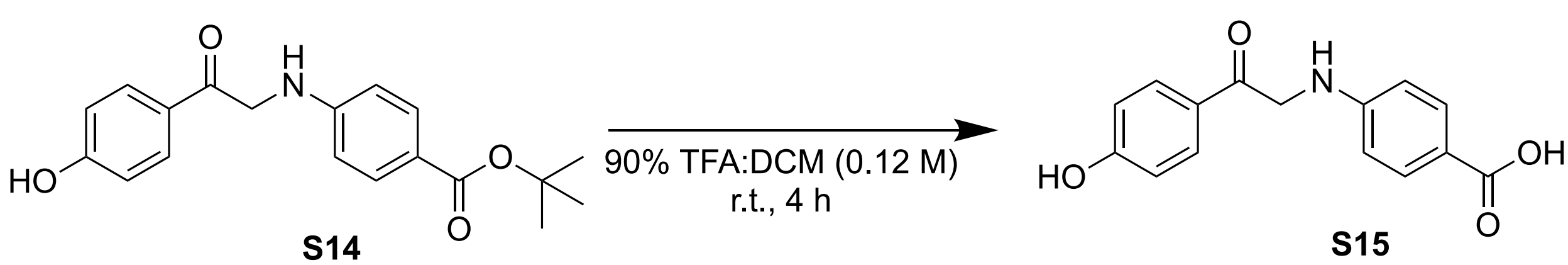

Into a 250-mL round bottom flask was added **S14** (5.00 g, 11.7 mmol), and the solid was dissolved in 100 mL of 90% trifluoroacetic acid in dichloromethane (0.12 M). Upon addition of the trifluoroacetic acid solution, a small amount of gas evolution was observed and the solution turned light brown. The reaction was lightly capped under an atmosphere of air and allowed to stir at

room temperature for 4 h. After this time, the solvent was removed under reduced pressure to give a red-brown residue which was purified by reverse-phase chromatography (0-40% acetonitrile/water with 0.1% formic acid) to give **S15** (2.03 g, 7.49 mmol, 64% yield) as a colorless solid.

^{1}H NMR (600 MHz, DMSO) δ 10.44 (s, 1H), 8.05 – 7.88 (m, 2H), 7.75 – 7.61 (m, 2H), 6.95 – 6.82 (m, 2H), 6.75 – 6.63 (m, 2H), 4.65 (s, 2H).

^{13}C NMR (151 MHz, CD_3CN) δ 195.21, 166.73, 156.15, 152.44, 152.12, 133.64, 131.99, 130.60, 122.85, 121.19, 112.72, 85.03, 80.46, 50.66, 28.50, 27.81.

HRMS (ESI): *m/z* for $C_{15}H_{14}NO_4^+$ $[M+H]^+$ calcd.: 272.0917, found: 272.0911.

Synthesis of 4-((2-hydroxy-2-(4-hydroxyphenyl)ethyl)amino)benzoic acid (**1d**)

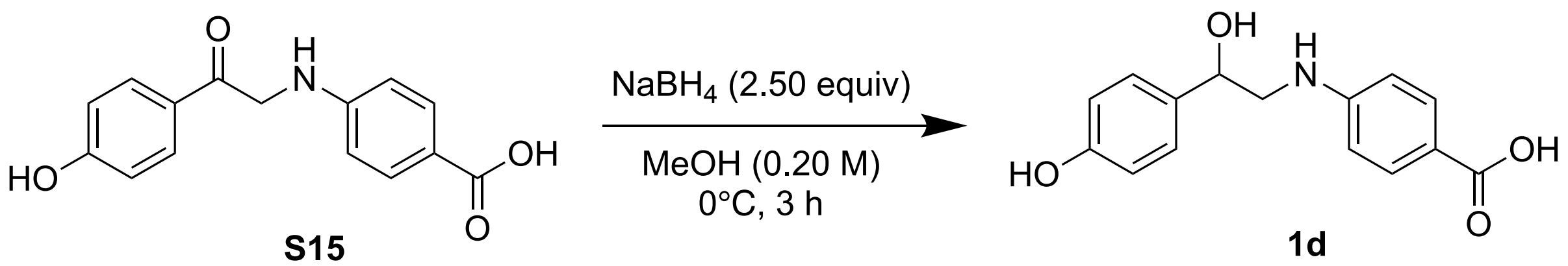

Into a 250-mL round bottom flask with a stir bar was added **S15** (2.00 g, 7.28 mmol). The solid was dissolved in 37 mL of methanol (0.20 M) and was stirred until a colorless solution had formed. Once homogeneous, the solution was cooled in an ice bath for 15 min before sodium borohydride (690 mg, 18.2 mmol, 2.50 equiv) was added in three portions over the course of 1 h. After addition of $NaBH_4$, the reaction was allowed to warm to room temperature for 3 h with stirring under air. Once complete, the reaction was quenched with slow addition of saturated NH_4Cl. The solvent was removed under reduced vacuum, leaving a colorless, heterogenous residue. This residue was directly purified by reverse-phase chromatography (0-30% acetonitrile/water with 0.1% formic acid) to give **1d** (458 mg, 1.67 mmol, 23% yield) as a colorless solid.

^{1}H NMR (500 MHz, CD_3CN) δ 8.15 (td, J = 7.1, 3.6 Hz, 2H), 7.73 (tq, J = 9.6, 2.9 Hz, 2H), 7.59 (dt, J = 9.2, 2.6 Hz, 2H), 6.60 (td, J = 7.1, 3.4 Hz, 2H), 4.94 (dd, J = 7.3, 4.9 Hz, 1H), 3.47 – 3.29 (m, 2H).

^{13}C NMR (126 MHz, DMSO) δ 168.01, 156.53, 152.07, 134.12, 130.99, 127.13, 118.44, 114.79, 110.86, 70.42, 50.91.

HRMS (ESI): *m/z* for $C_{15}H_{16}NO_4^+$$[M+H]^+$ calcd.: 274.1074, found: 274.1074.

Synthesis of 4-azidobenzoic acid (**S16**)

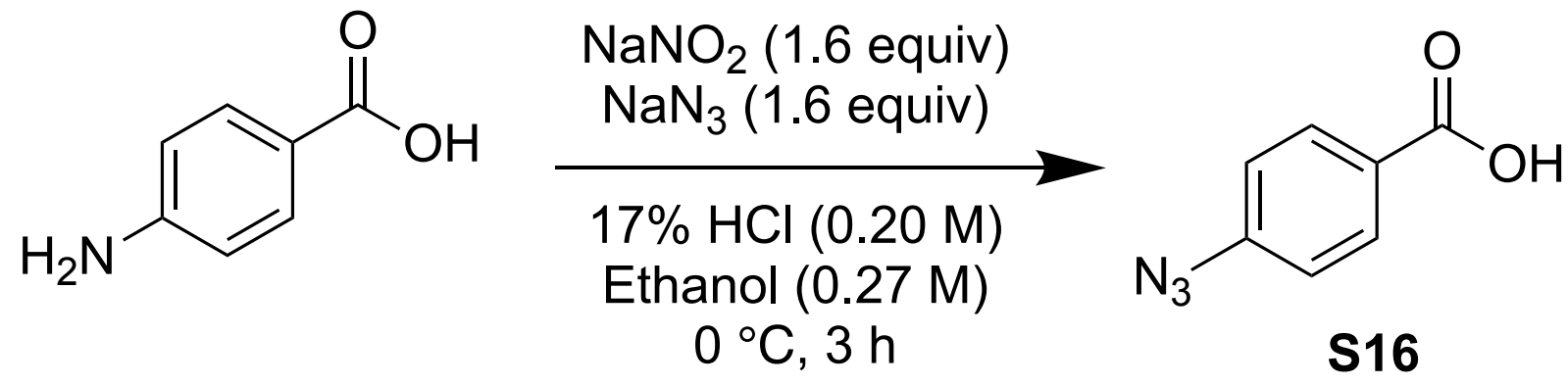

The procedure followed was a modified version of that in reference 57. Into a 250-mL beaker containing a stir bar was added 4-aminobenzoic acid (2.743 g, 20.00 mmol) and 100 mL of warm 17% HCl. This mixture was allowed to stir for 10 min, forming a white suspension. To this mixture was added ethanol (75 mL) until the 4-aminobenzoic acid was fully dissolved. The solution was then cooled in an ice bath for 30 min. Sodium nitrite (2.208 g, 32.00 mmol, 1.6 equiv) was dissolved in ~10 mL of diH_2O and added to the reaction mixture slowly over 15 min, causing the release of red NO_2 gas, and the solution to become light red. This mixture was allowed to stir at 0 °C for 30 min. Sodium azide (2.090 g, 32.00 mmol, 1.6 equiv) was dissolved in minimal diH_2O (~10 mL) and very slowly added to the reaction mixture dropwise over 20 min. With each addition, copious gas evolved and a white precipitate began to form. After the final addition of NaN_3, the reaction was allowed to stir for 20 min before the precipitate was removed by filtration through a medium glass frit. The solid was washed with copious diH_2O and allowed to dry. The solid was then recrystallized from hot diH_2O/Ethanol to give **S16** (2.54 g, 15.6 mmol, 78% yield) as an off-white crystalline solid.

^{1}H NMR (500 MHz, MeOD) δ 8.02 (d, *J* = 8.7 Hz, 1H), 7.13 (d, *J* = 8.7 Hz, 1H).
^{13}C NMR (126 MHz, MeOD) δ 168.97, 146.22, 132.64, 128.46, 119.90.
HRMS (ESI): *m/z* for $C_7H_6N_3O_2^+$ $[M+H]^+$ calcd.: 164.0455, found: 164.0453.

Synthesis of 4,4'-((1-(4-hydroxyphenyl)ethane-1,2-diyl)bis(azanediyl))dibenzoic acid (**2d**)

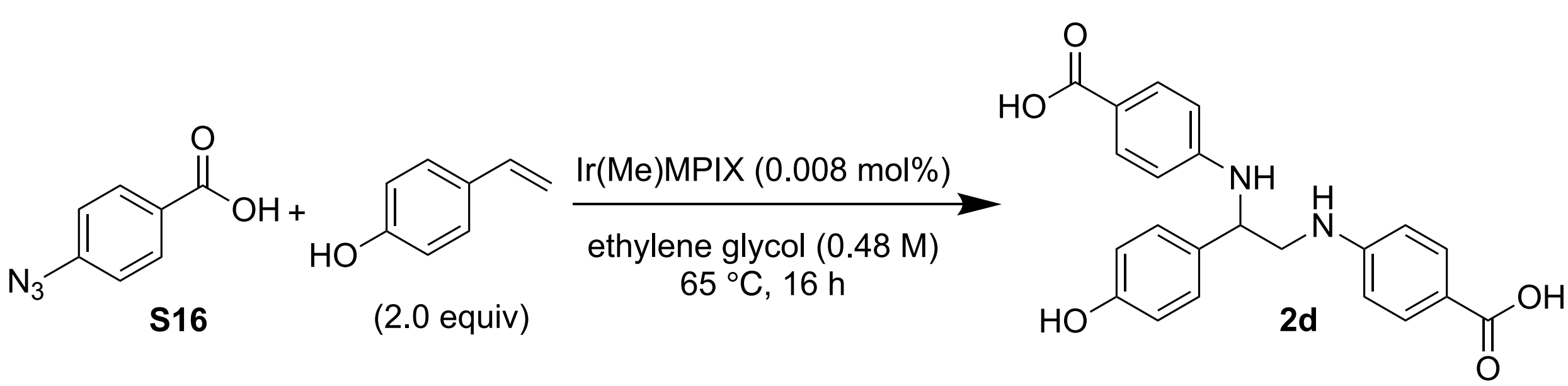

Ir(Me)MPIX was prepared as described by reference 58. Into a 20-mL scintillation vial containing a stir bar was added **S16** (500 mg, 3.06 mmol) and 4-vinylphenol as a 10% w/w solution in ethylene glycol (7.36 g, 6.13 mmol, 2.0 equiv; 0.48 M in ethylene glycol). The solution was degassed by sparging with N_2 for 30 min before 50 μL of a 15 mM solution of Ir(Me)MPIX (0.25 μmol, 0.008 mol%) in DMSO was added to the reaction mixture. The reaction was capped under an atmosphere of nitrogen and allowed to stir at 65 °C for 16 h. The reaction was then allowed to cool to room temperature before being poured into 40 mL of diH_2O, causing precipitation of a yellow solid. The heterogeneous mixture was extracted with ethyl acetate four times, dissolving the solid. The ethyl acetate washes were combined and transferred to a separatory funnel and washed with water and brine. The organic layer was dried over $MgSO_4$ and concentrated to give an off-white solid. This residue was dissolved in 0.1 M NaOH, and the solution was filtered through a cotton plug. The solution was acidified with glacial acetic acid, causing precipitation. When the solution reached pH 3, the solid was removed by filtration, washed with minimal water, and dried under vacuum to give **2d** (227 mg, 0.890 mmol, 25% yield) as a light-brown solid. The material was kept dry at -20 °C due to observed decomposition at room temperature.

^{1}H NMR (500 MHz, DMSO) δ 12.00 (s, 2H), 9.26 (s, 2H), 7.67 (d, *J* = 8.9 Hz, 2H), 7.58 (d, *J* = 8.9 Hz, 2H), 7.21 (d, *J* = 8.5 Hz, 2H), 6.93 (d, *J* = 7.2 Hz, 1H), 6.70 (d, *J* = 8.5 Hz, 2H), 6.65 – 6.60 (m, 2H), 6.56 – 6.47 (m, 3H), 4.62 – 4.49 (m, 1H), *4.31 (2H).

^{13}C NMR (126 MHz, DMSO) δ 167.50, 167.43, 156.50, 152.09, 151.87, 131.76, 131.18, 130.88, 127.69, 117.28, 117.23, 115.22, 111.82, 111.05, 55.08, 49.32.

HRMS (ESI): *m/z* for $C_{22}H_{20}N_2NaO_5^+$ $[M+Na]^+$ calcd.: 407.1601, found: 407.1600.

* Determined by DEPT-135 and HSQC.

Synthesis of *tert*-butyl 4-iodobenzoate (**S17**)

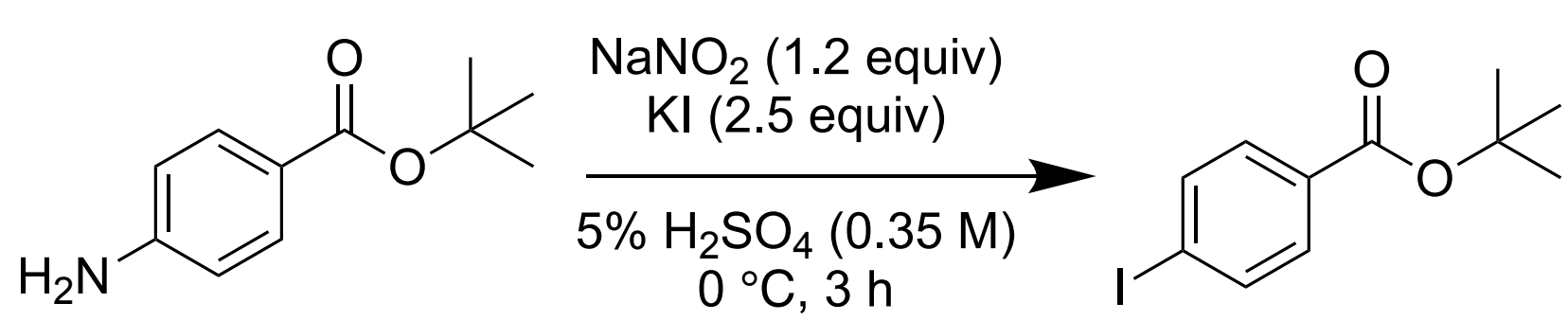

S17

Into a 1000-mL beaker with a stir bar under an atmosphere of air was measured 285 mL of diH_2O and 15 mL of 98% H_2SO_4 (5% v/v H_2SO_4). This solution was allowed to cool in an ice bath for 15 min, before *tert*-butyl 4-aminobenzoate (20.00 g, 103.5 mmol) was added. The solid was allowed

to stir until it formed a fine suspension. Large pieces of solid were broken up with a metal spatula. Sodium nitrite (8.57 g, 124.2 mmol, 1.2 equiv) was dissolved in minimal water and added slowly over 20 min to the reaction mixture. Upon addition, the reaction produced small amounts of red nitrogen dioxide gas while the solution turned a slight red-brown color. The reaction was allowed to stir at 0 °C for 1 h until all the solid was fully dissolved. Potassium iodide (34.36 g, 207.0 mmol, 2.0 equiv) was dissolved in minimal diH_2O and slowly added to the reaction mixture, causing formation of a dark red oil. Once all the KI was added, the reaction was allowed to stir at 0 °C for 1 h. The reaction was then layered with 100 mL of hexanes and allowed to stir vigorously for 10 min, before the organic layer was decanted and stored. The reaction was then washed two more times with 100-mL portions of hexanes, and all the organic fractions were combined. The organic layer was then transferred to a separatory funnel and sequentially washed with 1 N HCl, 1 M NaOH, diH_2O, and brine. The organic layer was then dried over $MgSO_4$, passed through a short silica plug with more hexanes, and concentrated under reduced pressure to give **S17** (26.76 g, 87.92 mmol, 85% yield) as a pale red oil. No further purification was necessary.

^{1}H NMR (500 MHz, $CDCl_3$) δ 7.79 – 7.72 (m, 2H), 7.68 (d, J = 8.5 Hz, 2H), 1.58 (s, 9H).

^{13}C NMR (126 MHz, $CDCl_3$) δ 165.41, 137.70, 131.70, 131.15, 100.25, 81.65, 28.37.

HRMS (ESI): m/z for $C_{11}H_{13}INaO_2^+$ $[M+Na]^+$ calcd.: 326.9852, found: 326.9855.

Synthesis of methyl 4-hydroxy-3-nitrobenzoate (**S18**)

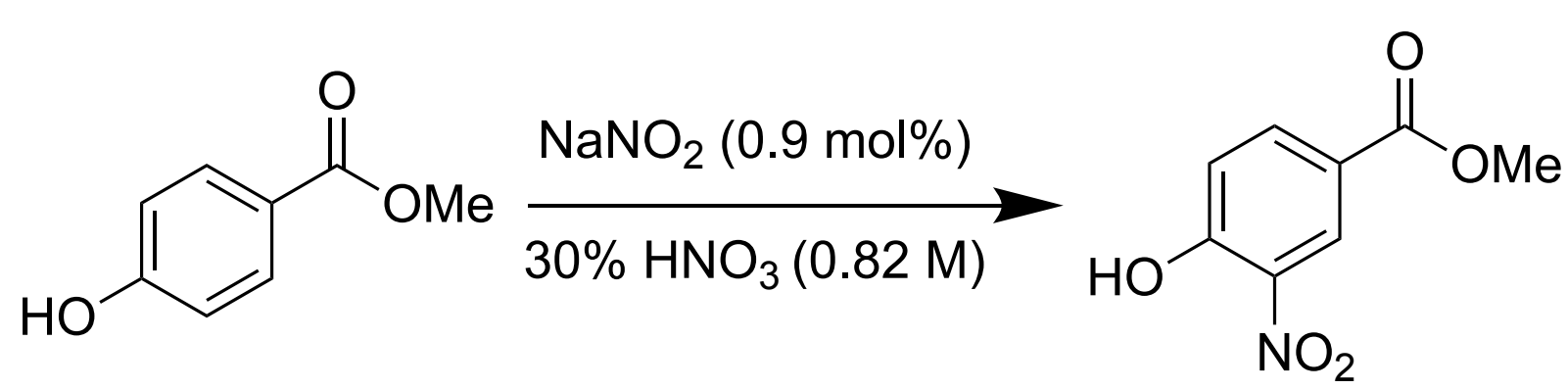

S18

Into a 500-mL beaker with a stir bar under an atmosphere of air was measured 100 mL of diH_2O and 100 mL of 68% nitric acid (final concentration 34% nitric acid). This solution was allowed to cool in an ice bath for 30 min with stirring, before finely divided methyl 4-hydroxybenzoate (25.00 g, 164.3 mmol, 0.82 M) was added and allowed to form a fine suspension. Large pieces of reagent were broken up with a metal spatula. Once fully dispersed, sodium nitrite (100 mg, 1.5 mmol, 0.9 mol%) was added to the reaction, causing formation of brown nitrogen dioxide gas. The reaction temperature was monitored with a thermometer and was allowed to stir at 0 °C for 1 h, before warming to room temperature over the course of 4 h. The solution slowly turned light-yellow and

darkened with time. After three total hours of reaction time, most of the solid had dissolved before a light-yellow precipitate began appearing. The reaction was then monitored by TLC for consumption of starting material. Once the reaction progress seemed to plateau, the reaction mixture was cooled in an ice bath for 20 min, causing further precipitation. The solid was then filtered through a medium porosity glass frit, washed with cold diH_2O, and dried. The resulting solid was then dried under vacuum overnight to give **S18** (28.24 g, 143.2 mmol, 87% yield) as a light-orange powder. No further purification was necessary.

^{1}H NMR (500 MHz, DMSO) δ 11.93 (s, 1H), 8.42 – 8.30 (m, 1H), 8.04 (dt, *J* = 8.7, 2.0 Hz, 1H), 7.21 (dd, *J* = 8.7, 1.2 Hz, 1H), 3.83 (s, 3H).

^{13}C NMR (126 MHz, DMSO) δ 164.65, 155.92, 136.83, 135.30, 126.83, 120.45, 119.46, 52.38.

HRMS (ESI): *m/z* for $C_8H_7NNaO_5^+$ $[M+Na]^+$ calcd.: 220.0216, found: 220.0221.

Synthesis of 2-hydroxy-5-(methoxycarbonyl)benzenaminium acetate (**S19**)

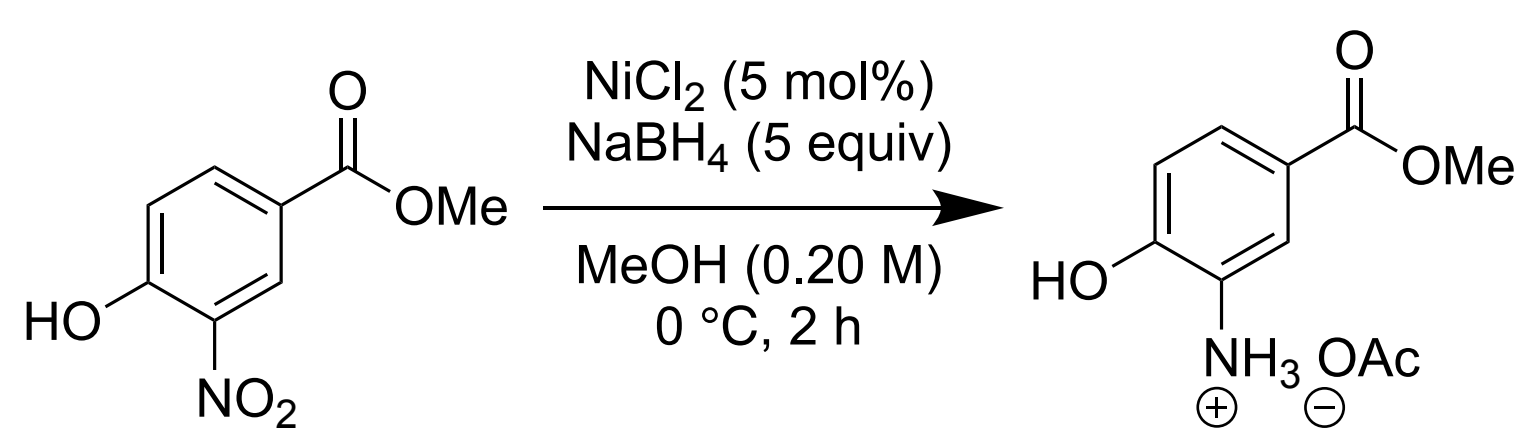

S18 **S19**

Into a 500 mL round bottom flask with a stir bar under an atmosphere of air was added nickel(II) chloride hexahydrate (1.21 g, 5.07 mmol, 10 mol%) and methanol (250 mL, 0.20 M), which slowly turned the solution green. To this vessel was added a portion of sodium borohydride (1.00 g, 0.52 equiv) very slowly, which caused vigorous bubbling and formation of a black precipitate. This mixture was allowed to stir for 10 min. The suspension was then cooled in an ice bath for 20 min with stirring. To the reaction mixture was added **S18** (10.00 g, 50.72 mmol) slowly over 10 min, which caused the mixture to become yellow orange as the phenol was deprotonated, forming a yellow precipitate. Over the course of 3 h, the remaining sodium borohydride (8.59 g; 9.59 g total, 253.6 mmol total, 5.0 equiv total) was added very slowly, in 0.5 g portions. Upon each addition, vigorous gas evolution and heat was observed, causing the solution to reflux gently. After the final addition of sodium borohydride, the reaction was stirred for 1 h at 0 °C. The reaction was quenched with the very slow addition of 20 mL of water and 10 mL of glacial acetic acid until the pH was ~3. The mixture was concentrated under reduced pressure to give a black-yellow residue. To the

residue was added water and ethyl acetate, and the mixture was transferred to a separatory funnel. The organic layer was stored, and the aqueous layer was extracted three more times with ethyl acetate. The organic layers were combined and washed with brine and dried over $MgSO_4$. The solvent was then evaporated under reduced pressure to give a brown residue, which was suspended in dichloromethane. Glacial acetic acid was then slowly added until no further precipitate was formed. The solution was allowed to cool at -20 °C in a freezer overnight. The precipitate was then filtered through a fine glass frit, washed thoroughly with dichloromethane, and dried to give **S19** (5.562 g, 24.48 mmol, 48% yield) as a light-brown acetate salt.

^{1}H NMR (500 MHz, DMSO) δ 9.94 (s, 1H), 7.23 (d, *J* = 2.1 Hz, 1H), 7.09 (dd, *J* = 8.1, 2.1 Hz, 1H), 6.70 (d, *J* = 8.2 Hz, 1H), 3.74 (s, 3H), 1.91 (s, 3H).

^{13}C NMR (126 MHz, DMSO) δ 172.12, 166.70, 148.70, 136.69, 120.73, 118.87, 114.77, 113.70, 51.47, 21.11.

HRMS (ESI): *m/z* for $C_8H_{10}NO_3^+$ $[M\text{-}OAc+H]^+$ calcd.: 168.0655, found: 168.0659.

Synthesis of methyl 3-((4-(*tert*-butoxycarbonyl)phenyl)amino)-4-hydroxybenzoate (**S20**)

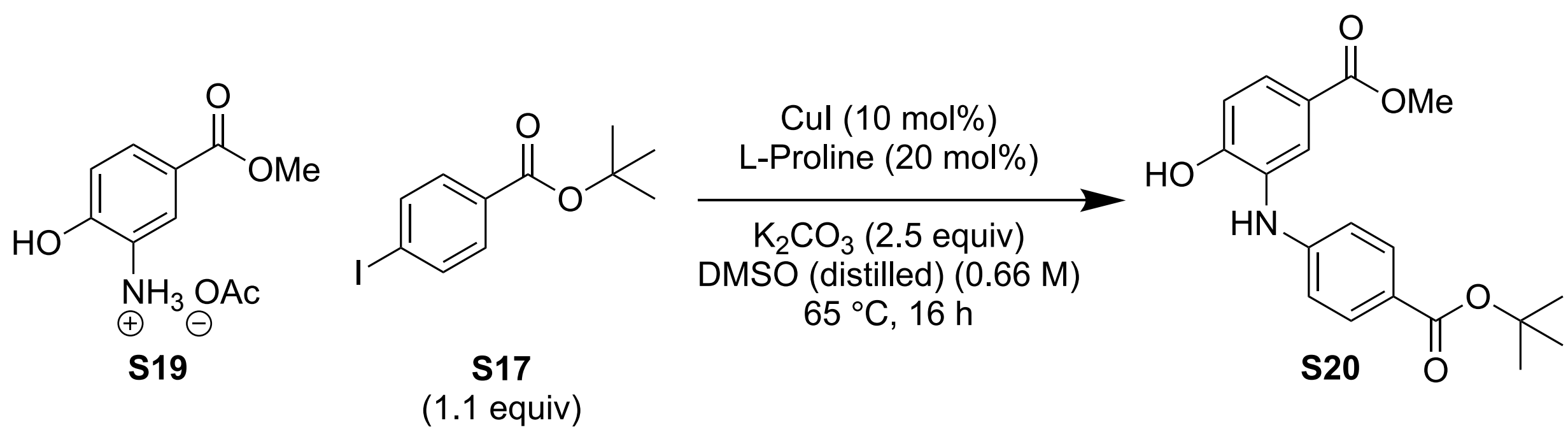

The procedure followed was a modified version of that in reference 59. DMSO was dried over CaH_2 for 24 h, vacuum distilled, and stored under an atmosphere of nitrogen prior to use. Into an oven-dried 20-mL scintillation vial containing a stir bar was added **S19** (1.00 g, 5.98 mmol), **S17** (2.00 g, 6.58 mmol, 1.1 equiv), K_2CO_3 (2.48 g, 17.9 mmol, 3.0 equiv), CuI (114 mg, 0.598 mmol, 10 mol%), and L-proline (138 mg, 1.20 mmol, 20 mol%). The vessel was placed under vacuum and backfilled with nitrogen three times. DMSO (9.1 mL, 0.66 M) was then added, and the vial was capped under an atmosphere of nitrogen. The reaction mixture was then allowed to stir at 65 °C for 16 h, during which time the reaction mixture became very dark red and viscous. The reaction was quenched by pouring the hot mixture into diH_2O, causing precipitation of a grey solid. The vial was further washed with diH_2O. Ethyl acetate was then added to the aqueous suspension,

dissolving the solid. This biphasic mixture was then transferred to a separatory funnel, and the organic layer stored. The aqueous layer was then extracted once more with ethyl acetate. The organic layers were combined, washed two times with diH_2O and then once with brine. The organic layer was then dried over $MgSO_4$ and concentrated under reduced pressure to yield a grey-brown residue. The residue was purified by column chromatography (15-30% ethyl acetate/hexanes) to give **S20** (957 mg, 2.79 mmol, 47% yield) as a light-red foam.

^{1}H NMR (500 MHz, $CDCl_3$) δ 7.95 (d, *J* = 2.0 Hz, 1H), 7.87 (d, *J* = 8.5 Hz, 2H), 7.82 – 7.76 (m, 1H), 7.02 (d, *J* = 8.4 Hz, 1H), 6.81 (d, *J* = 8.5 Hz, 2H), 5.80 (s, 1H), 3.86 (s, 3H), 1.57 (s, 9H).

^{13}C NMR (126 MHz, $CDCl_3$) δ 166.91, 166.16, 154.63, 148.59, 131.62, 128.28, 128.19, 125.51, 123.92, 123.23, 115.62, 114.88, 80.86, 52.30, 28.51.

HRMS (ESI): *m/z* for $C_{19}H_{21}KNO_5^+$ $[M+K]^+$ calcd.: 382.1052, found: 382.1064.

Synthesis of methyl 3-((4-(*tert*-butoxycarbonyl)phenyl)(*tert*-butyldimethylsilyl)amino)-4-((*tert*-butyldimethylsilyl)oxy)benzoate (**S21**)

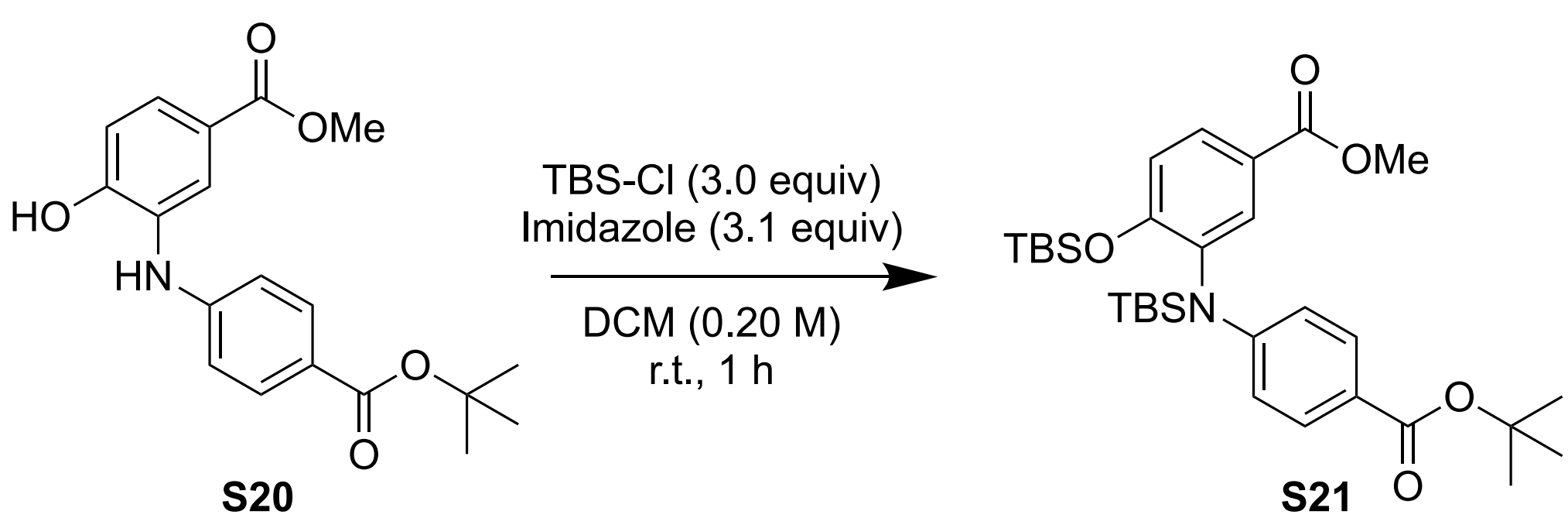

Into an oven-dried, 250-mL round bottom flask containing a stir bar was added **S20** (2.87 g, 8.36 mmol), imidazole (1.76 g, 25.9 mmol, 3.1 equiv), and TBS-Cl (3.78 g, 25.1 mmol, 3.0 equiv). These solids were then dissolved in dichloromethane (42 mL, 0.20 M), creating a yellow solution, and the reaction mixture was allowed to stir at room temperature under an atmosphere of air for 1 h. During this time, a colorless precipitate formed. The reaction mixture was then filtered through a plug of Celite with dichloromethane and concentrated under reduced pressure to give a colorless residue. This residue was purified by column chromatography (0-20% ethyl acetate/hexanes) to give **S21** (3.48 g, 6.08 mmol, 73% yield) as a colorless foam.

^{1}H NMR (500 MHz, $CDCl_3$) δ 8.05 (d, *J* = 2.1 Hz, 1H), 7.94 – 7.87 (m, 2H), 7.59 (dd, *J* = 8.4, 2.1 Hz, 1H), 7.08 – 7.01 (m, 2H), 6.88 (d, *J* = 8.4 Hz, 1H), 3.87 (s, 3H), 1.58 (s, 9H), 0.99 (s, 9H), 0.90 (s, 9H), 0.23 (s, 6H), 0.08 (s, 6H).

^{13}C NMR (126 MHz, $CDCl_3$) δ 167.01, 165.88, 149.81, 146.64, 133.13, 131.58, 124.18, 123.99, 123.84, 119.12, 118.80, 115.37, 80.54, 68.13, 52.22, 28.48, 25.88, 25.87, 25.79, 18.46, 18.20, -3.38, -4.18.

HRMS (ESI): *m/z* for $C_{25}H_{35}NNaO_5Si$ $[M\text{-}TBS+H+Na]^+$ calcd.: 480.2182, found: 480.2186.

Synthesis of *tert*-butyl 4-((2-((*tert*-butyldimethylsilyl)oxy)-5-(hydroxymethyl)phenyl)amino) benzoate (**S22**)

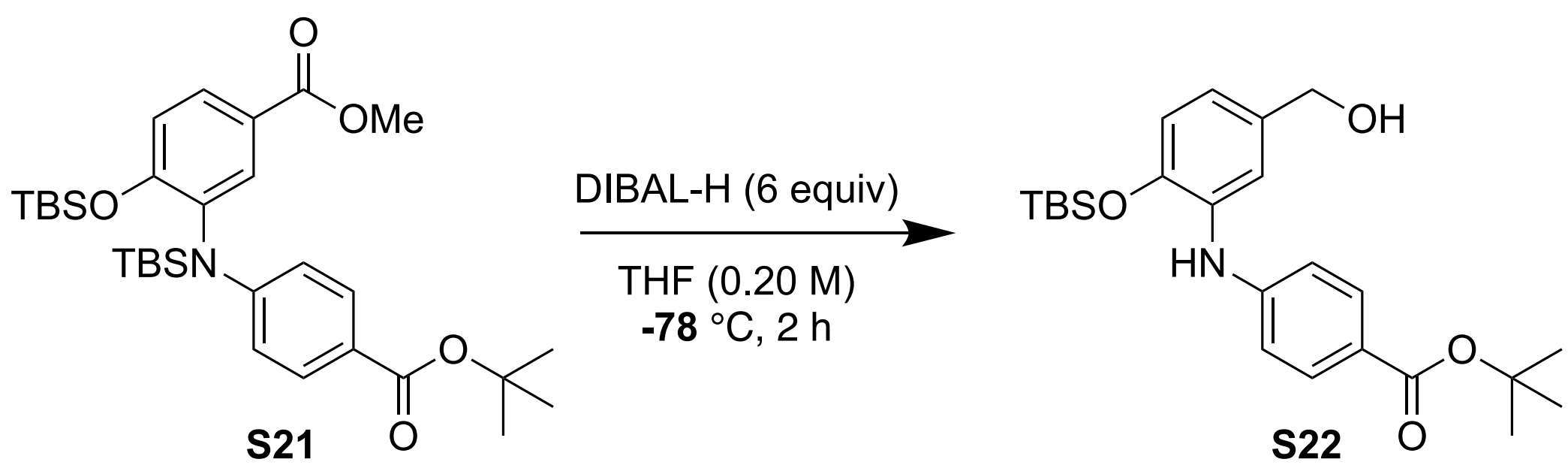

Into a flame-dried, 100-mL round bottom flask with a stir bar was added **S21** (3.48 g, 6.08 mmol) and tetrahydrofuran (30 mL, 0.20 M) under nitrogen. This solution was stirred until fully dissolved then cooled in a dry ice/acetone bath for 20 min. Once cooled, a 1.0 M solution of DIBAL-H in dichloromethane (36.5 mL, 36.5 mmol, 6.0 equiv) was added over the course of 1 h. This solution was allowed to stir at -78 °C for 1 h before being quenched with the slow addition of acetone (12 mL, 160 mmol, 27 equiv). The reaction mixture was allowed to warm to room temperature before being transferred to a separatory funnel. A 2 M solution of Rochelle salt was added and the organic layer was collected. The aqueous layer was then washed three times with ethyl acetate and the organic layers were combined. The organic layer was then washed sequentially with water and brine. The organic layer was dried over $MgSO_4$ and concentrated under reduced pressure to give a light yellow residue which was purified by column chromatography (0-20% ethyl acetate) to give **S22** (1.77 g, 4.12 mmol, 68% yield) as a colorless foam.

^{1}H NMR (500 MHz, $CDCl_3$) δ 7.94 – 7.84 (m, 2H), 7.39 (d, J = 2.0 Hz, 1H), 7.04 – 7.00 (m, 2H), 6.91 – 6.83 (m, 2H), 4.60 (s, 2H), 1.58 (s, 9H), 0.99 (s, 9H), 0.19 (s, 6H).

^{13}C NMR (126 MHz, $CDCl_3$) δ 165.98, 147.12, 145.28, 134.65, 133.16, 131.53, 123.54, 121.00, 119.43, 117.27, 115.13, 80.48, 65.43, 28.52, 25.97, 18.46, -4.19.

HRMS (ESI): *m/z* for $C_{24}H_{36}NO_4Si^+$ $[M+H]^+$ calcd.: 430.2408, found: 430.2422.

Synthesis of *tert*-butyl 4-((2-((*tert*-butyldimethylsilyl)oxy)-5-formylphenyl)amino)benzoate (**S23**)

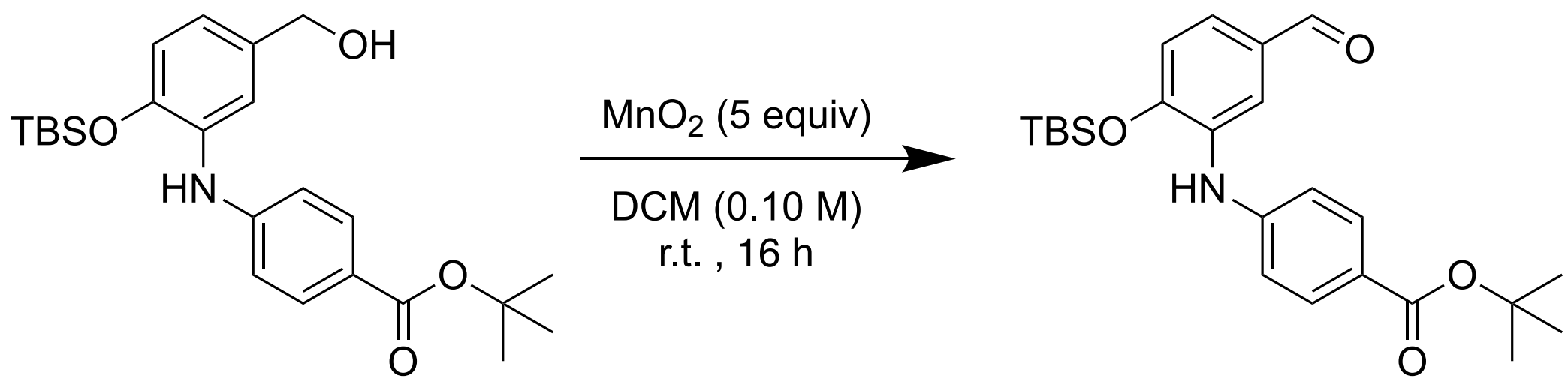

S22 **S23**

Manganese dioxide was prepared freshly as described by reference 60. Into an oven-dried, 100-mL round bottom flask containing a stir bar was added **S22** (1.77 g, 4.12 mmol), MnO_2 (1.79 g, 20.6 mmol, 5 equiv), and dichloromethane (41 mL, 0.10 M). This suspension was allowed to stir under air for 16 h at room temperature. The reaction was quenched by filtration through a silica plug with ethyl acetate. The filtrate was concentrated under reduced pressure to give a dark-red residue which was purified by column chromatography (15-30% ethyl acetate/hexanes) to give **S23** (1.39 g, 3.25 mmol, 79% yield) as an off-white foam.

^{1}H NMR (500 MHz, $CDCl_3$) δ 9.85 (s, 1H), 7.98 – 7.92 (m, 2H), 7.90 (d, *J* = 2.0 Hz, 1H), 7.40 (dd, *J* = 8.2, 2.1 Hz, 1H), 7.13 – 7.06 (m, 2H), 6.98 (d, *J* = 8.1 Hz, 1H), 1.59 (s, 9H), 1.02 (s, 9H), 0.28 (s, 6H).

^{13}C NMR (126 MHz, $CDCl_3$) δ 191.12, 165.69, 150.72, 145.94, 134.28, 131.55, 130.90, 125.05, 124.58, 118.84, 116.56, 115.94, 80.61, 28.43, 25.84, 18.45, -4.16.

HRMS (ESI): *m/z* for $C_{24}H_{34}NO_4Si^+$ $[M+H]^+$ calcd.: 428.2252, found: 428.2265.

Synthesis of 4-((5-formyl-2-hydroxyphenyl)amino)benzoic acid (**S24**)

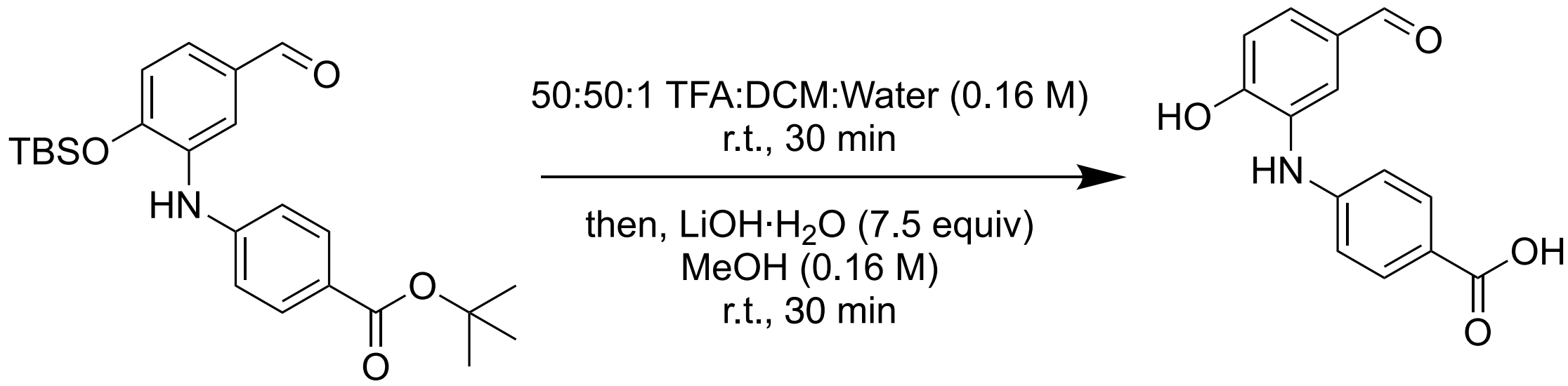

S23 **S24**

Into a 50-mL round bottom flask was added **S23** (1.39 g, 3.25 mmol) and 20 mL of a 50:50:1 solution of trifluoroacetic acid : dichloromethane : water (0.16 M). Upon addition, the reaction mixture became vividly red and emitted a small amount of vapor. This solution was allowed to stir at room temperature for 30 min before the solvent was removed under reduced pressure to give a very dark-red residue. The reaction mixture was then thoroughly dried under vacuum before lithium hydroxide monohydrate (1.02 g, 24.4 mmol, 7.5 equiv) and methanol (20 mL, 0.16 M) were added. This addition caused the reaction to quickly turn pale yellow. The reaction mixture was allowed to stir at room temperature for 30 min under an atmosphere of air before being quenched with glacial acetic acid (1.40 mL, 24.4 mmol, 7.5 equiv). The solvent was then removed under reduced pressure to give a dark brown residue. The residue was suspended in diH_2O to dissolve the lithium acetate, producing an aqueous suspension of a fine brown solid. The precipitate was filtered through a fine glass frit, washed with water, and dried thoroughly to give **S24** (805 mg, 3.13 mmol, 96% yield) as a light brown powder. No further purification was necessary.

^{1}H NMR (500 MHz, DMSO) δ 9.76 (s, 1H), 8.10 (s, 1H), 7.80 – 7.75 (m, 2H), 7.74 (d, J = 2.1 Hz, 1H), 7.50 (dd, J = 8.2, 2.1 Hz, 1H), 7.07 (d, J = 8.2 Hz, 1H), 7.03 – 6.94 (m, 2H).

^{13}C NMR (126 MHz, DMSO) δ 191.27, 167.38, 155.88, 148.61, 131.06, 129.77, 128.59, 126.88, 121.35, 120.39, 115.97, 114.39.

HRMS (ESI): m/z for $C_{14}H_{11}NNaO_4^+$ $[M+Na]^+$ calcd.: 280.058, found: 280.0591.

Synthesis of 4-((2-hydroxy-5-vinylphenyl)amino)benzoic acid (**4**)

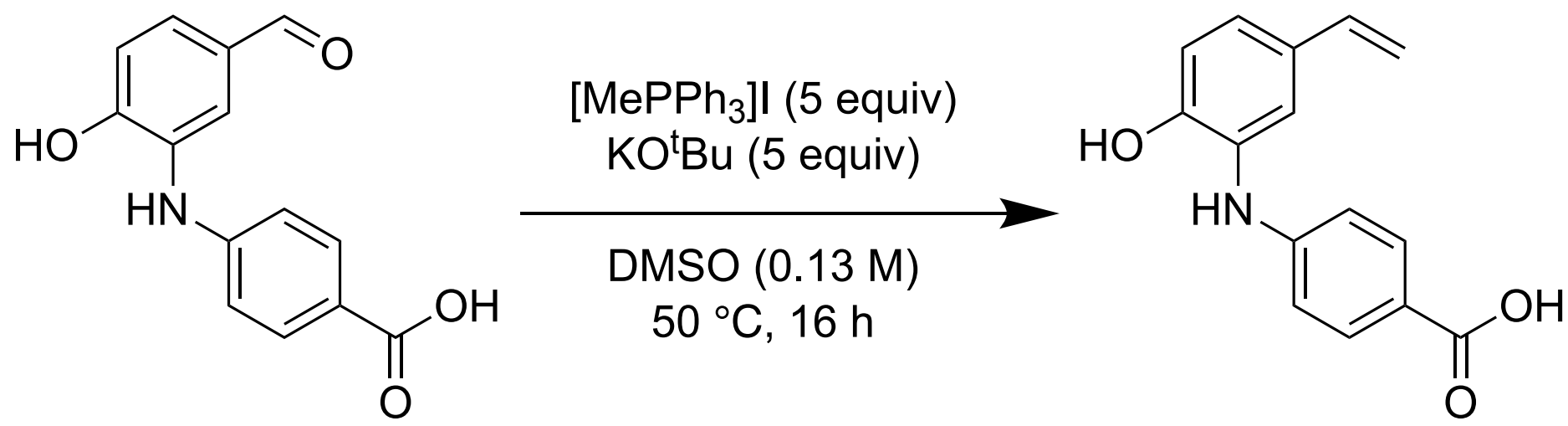

DMSO was dried over CaH_2 for 24 h, vacuum distilled, and stored under an atmosphere of nitrogen prior to use. Into an oven-dried, 20-mL scintillation vial containing a stir bar under nitrogen was added potassium tert-butoxide (1.09 g, 9.72 mmol, 5.0 equiv), methyltriphenylphosphanium iodide (3.93 g, 9.72 mmol, 5.0 equiv), and DMSO (10 mL). The vial was then capped under an atmosphere of nitrogen and allowed to stir at 50 °C for 1 h, forming a bright yellow solution. **S25** was dissolved in 5 mL of DMSO and added to the reaction mixture under an atmosphere of nitrogen (final concentration 0.13 M). The vial was once again capped under an atmosphere of nitrogen and allowed to stir at 50°C for 16 h. After stirring overnight, the reaction was quenched with the addition of methanol. The excess methanol was removed under reduced pressure before the DMSO was removed by vacuum distillation. The final amount of DMSO was then evaporated overnight under vacuum to give a dark brown residue. This residue was purified by reverse-phase chromatography (20-50% acetonitrile/water with 0.1% formic acid) to give **4** (242 mg, 0.95 mmol, 49% yield) as a light-yellow solid.

^{1}H NMR (600 MHz, DMSO) δ 12.16 (s, 1H, COOH), 9.96 – 9.54 (m, 1H, Phenol-OH), 7.93 (s, 1H, NH), 7.79 – 7.67 (m, 2H), 7.29 (d, *J* = 2.2 Hz, 1H), 7.07 (dd, *J* = 8.3, 2.2 Hz, 1H), 6.94 – 6.81 (m, 3H), 6.62 (dd, *J* = 17.6, 11.0 Hz, 1H), 5.58 (dd, *J* = 17.7, 1.1 Hz, 1H), 5.07 (dd, *J* = 10.8, 1.1 Hz, 1H).

^{13}C NMR (151 MHz, DMSO) δ 167.39, 150.37, 149.64, 136.45, 130.97, 128.86, 128.41, 122.36, 120.75, 119.26, 116.13, 113.48, 111.22.

HRMS (ESI): *m/z* for $C_{15}H_{14}NO_3^+$ $[M+H]^+$ calcd.: 256.0968, found: 256.0960.

Synthesis of 4-(*N*-(2-hydroxy-5-vinylphenyl)acetamido)benzoic acid (**5**)

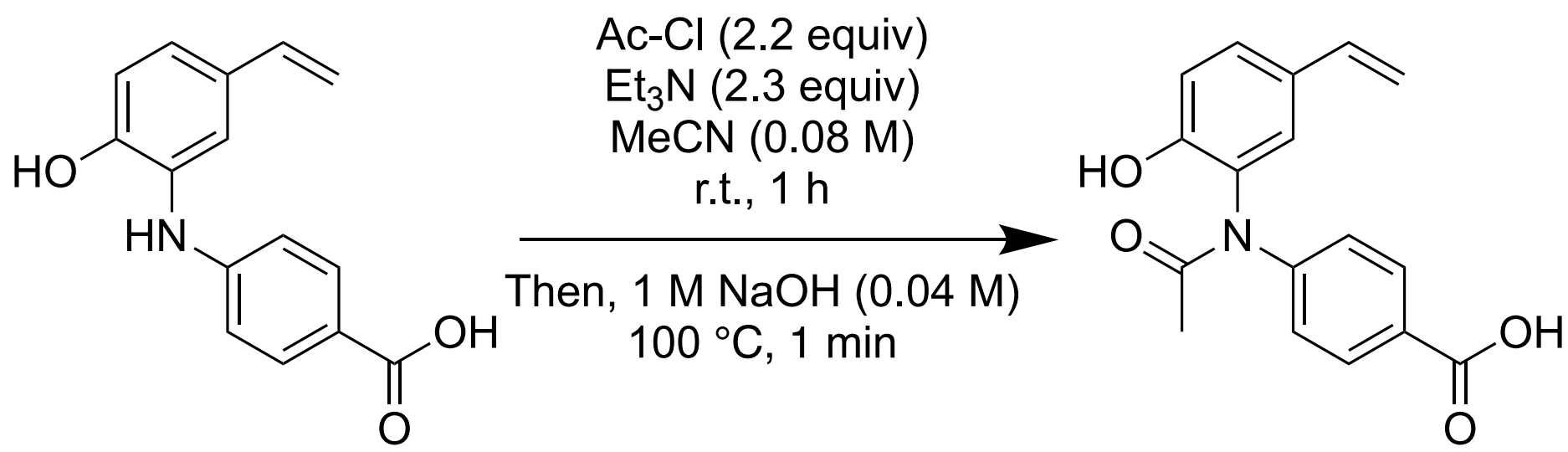

Into an oven-dried, 20-mL scintillation vial containing a stir bar was added **4** (100 mg, 0.392 mmol) and acetonitrile (5 mL, 0.08 M). This solution was stirred until dissolved before acetyl chloride (61 μL, 0.86 mmol, 2.2 equiv) then triethylamine (125 μL, 0.90 mmol, 2.3 equiv) were added sequentially. The solution became yellow and a small amount of colorless precipitate formed. The reaction mixture was allowed to stir at room temperature under an atmosphere of air for 1 h before the solvent was evaporated under reduced pressure, leaving a brown residue. This residue was then dissolved in 10 mL of 1 M NaOH (0.04 M) and heated at 100 ℃ for 1 min before being allowed to cool to room temperature. The reaction mixture was transferred to a separatory funnel and acidified to pH 1 with 6 N HCl, causing the solution to become cloudy. The aqueous layer was extracted three times with ethyl acetate. The organic layers were combined, washed sequentially with diH_2O and brine, then dried over MgSO4, and concentrated under reduced pressure to give a brown residue. This residue was purified by reverse-phase column chromatography (20-40% acetonitrile/water with 0.1% formic acid) to give **5** (63.1 mg, 0.212 mmol, 63% yield) as a light-brown solid.

^{1}H NMR (700 MHz, MeOD) δ 8.01 (d, *J* = 8.3 Hz, 2H), 7.54 – 7.42 (m, 2H), 7.42 – 7.34 (m, 1H), 7.32 (s, 1H), 6.98 (d, *J* = 8.3 Hz, 1H), 6.63 (dd, *J* = 17.7, 10.9 Hz, 1H), 5.64 (d, *J* = 17.6 Hz, 1H), 5.12 (d, *J* = 10.9 Hz, 1H), 2.07 (s, 3H).

^{13}C NMR (151 MHz, MeOD) δ 172.45, 167.84, 153.28, 146.62, 135.33, 130.77, 129.97, 129.85, 128.02, 127.42, 127.30, 125.53, 116.64, 111.20, 29.27.

HRMS (ESI): *m/z* for $C_{17}H_{16}NO_4^+$ $[M+H]^+$ calcd.: 298.1074, found: 298.1076.

Synthesis of 4-((4-hydroxyphenethyl)amino)benzoic acid (**6**)

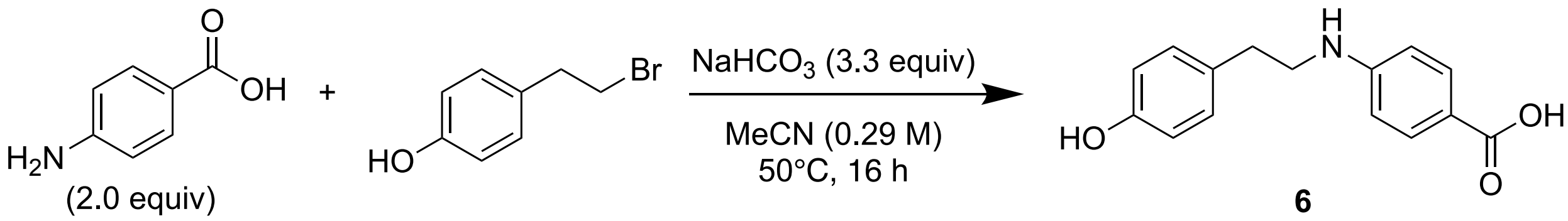

Into a 250-mL round bottom flask charged with a stir bar was added 4-aminobenzoic acid (6.82 g, 49.7 mmol, 2.0 equiv), sodium carbonate (6.89 g, 82.1 mmol, 3.3 equiv), and 4-(2-bromoethyl)phenol (5.00 g, 24.9 mmol). These materials were dissolved in 85 mL of acetonitrile (0.29 M) and heated with stirring at 50 °C in an oil bath for 16 h under an atmosphere of air. After allowing to react overnight, the excess sodium carbonate was removed by filtration using a fine glass frit, and the filtrate was concentrated under reduced pressure, leaving a light-brown residue which was purified by reverse-phase chromatography (0-40% acetonitrile/water with 0.1% formic acid) to give **6** (2.38 g, 9.25 mmol, 37% yield) as a white, crystalline solid.

^{1}H NMR (500 MHz, CD_3CN) δ 7.81 – 7.73 (m, 1H), 7.13 – 7.05 (m, 1H), 6.79 – 6.71 (m, 1H), 6.64 – 6.57 (m, 1H), 3.34 (t, J = 7.3 Hz, 1H), 2.79 (t, J = 7.3 Hz, 1H).

^{13}C NMR (126 MHz, CD_3CN) δ 169.00, 156.32, 153.88, 132.65, 131.61, 130.81, 117.57, 116.13, 112.18, 45.46, 34.90.

HRMS (ESI): *m/z* for $C_{15}H_{16}NO_3^+$ $[M+H]^+$ calcd.: 258.1125, found: 258.1130.

Synthesis of 4-((4-acetoxyphenethyl)amino)benzoic acid (**7**)

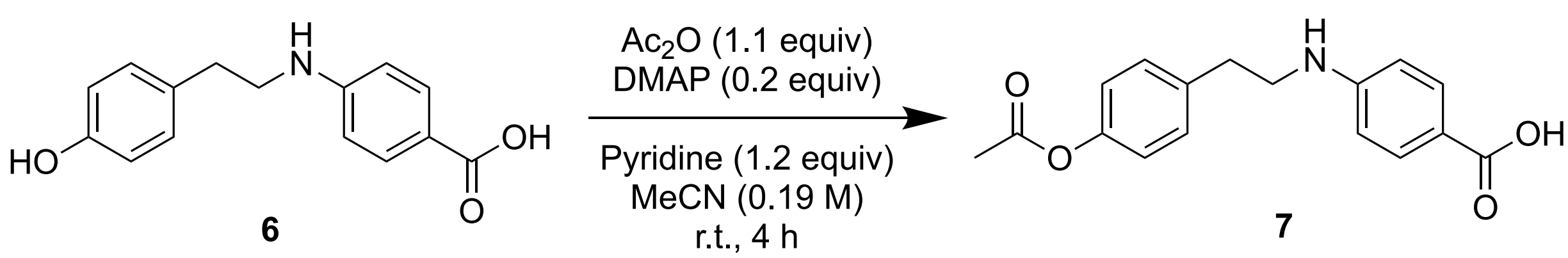

Into a 4-mL vial containing a stir bar was added **6** (100 mg, 0.389 mmol), DMAP (9.50 mg, 0.077 mmol, 0.2 equiv), and acetonitrile (2.0 mL, 0.19 M). This mixture was allowed to stir until fully dissolved, at which time pyridine (38 μL, 0.446 mmol, 1.2 equiv) was added. The reaction mixture was allowed to stir for 10 min over which time it turned pale yellow. Acetic anhydride (40 μL, 0.430 mmol, 1.1 equiv) was added in one portion. This addition caused an immediate loss of the yellow color, resulting in a colorless solution. The reaction mixture was allowed to stir under an atmosphere of air for 4 h, before being concentrated under reduced pressure to give a colorless

residue. This residue was purified by reverse-phase chromatography (20-40% acetonitrile/water with 0.1% formic acid) to give **7** (106 mg, 0.354 mmol, 91% yield) as a colorless, crystalline solid.
^{1}H NMR (500 MHz, CD_3CN) δ 7.76 (dd, *J* = 8.8, 2.1 Hz, 2H), 7.34 – 7.24 (m, 2H), 7.07 – 6.97 (m, 2H), 6.62 (dd, *J* = 8.8, 2.1 Hz, 2H), 5.13 (s, 1H), 3.40 (t, *J* = 7.3 Hz, 2H), 2.90 (t, *J* = 7.3 Hz, 2H), 2.23 (s, 3H).
^{13}C NMR (126 MHz, CD_3CN) δ 170.69, 168.29, 153.70, 150.43, 138.23, 132.58, 130.76, 122.72, 117.90, 112.21, 45.17, 35.13, 21.24.
HRMS (ESI): *m/z* for $C_{17}H_{18}NO_4^+$ $[M+H]^+$ calcd.: 300.1230, found: 300.1237.

NMR Spectra

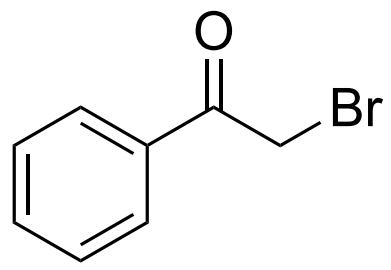

S1

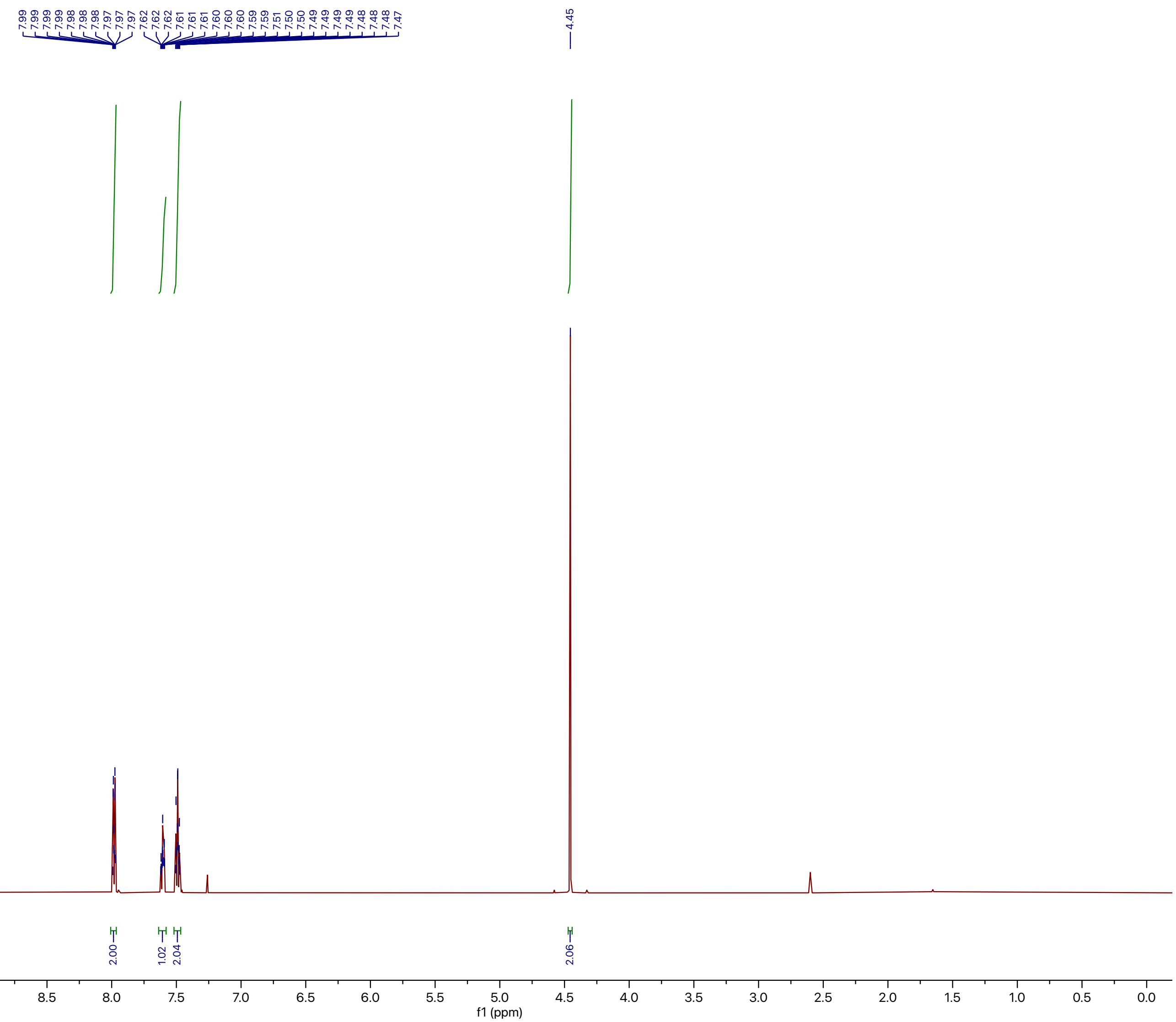

^{1}H-NMR (600 MHz, $CDCl_3$) of **S1**.

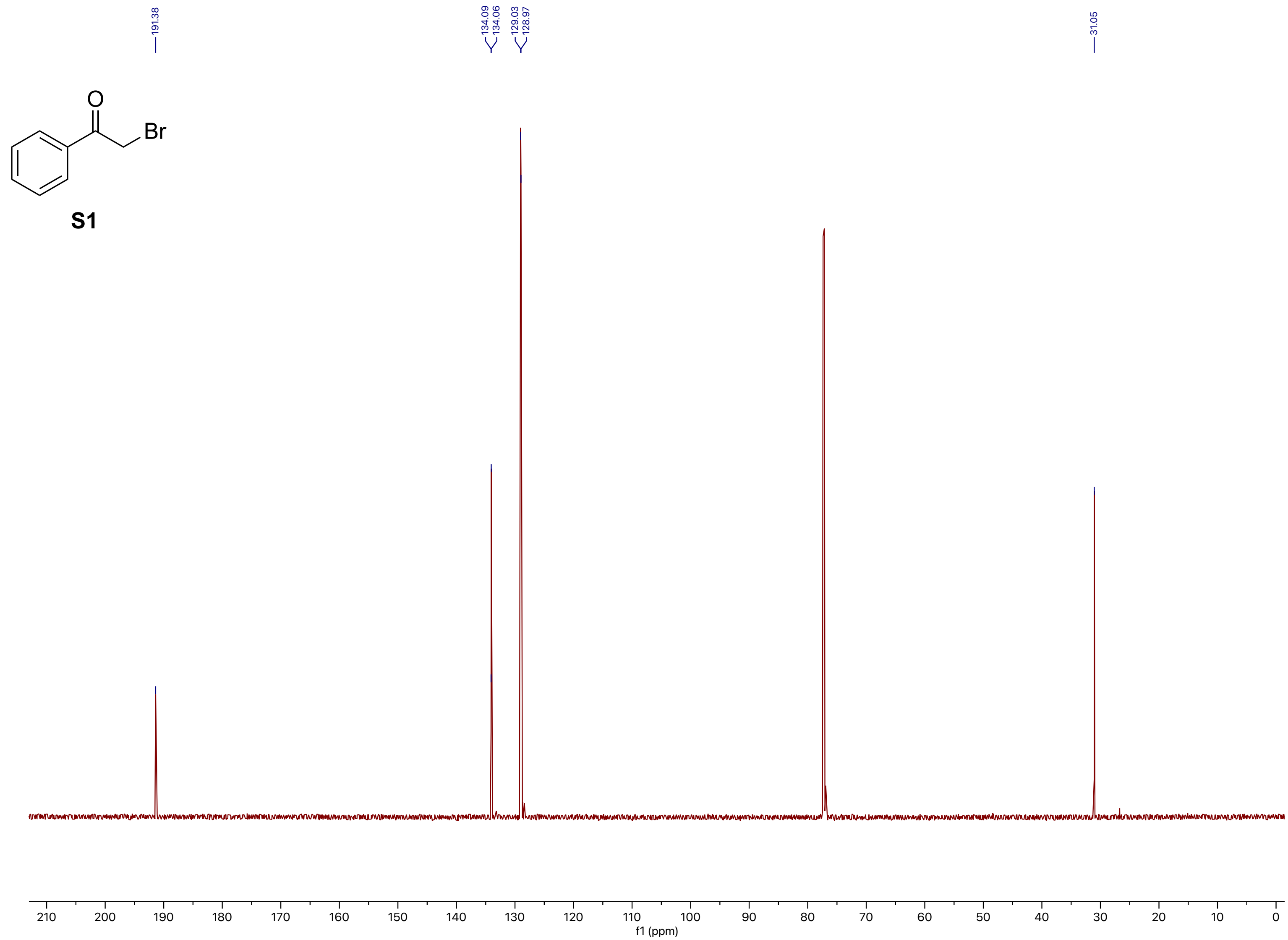

S1

^{13}C-NMR (151 MHz, $CDCl_3$) of **S1**.

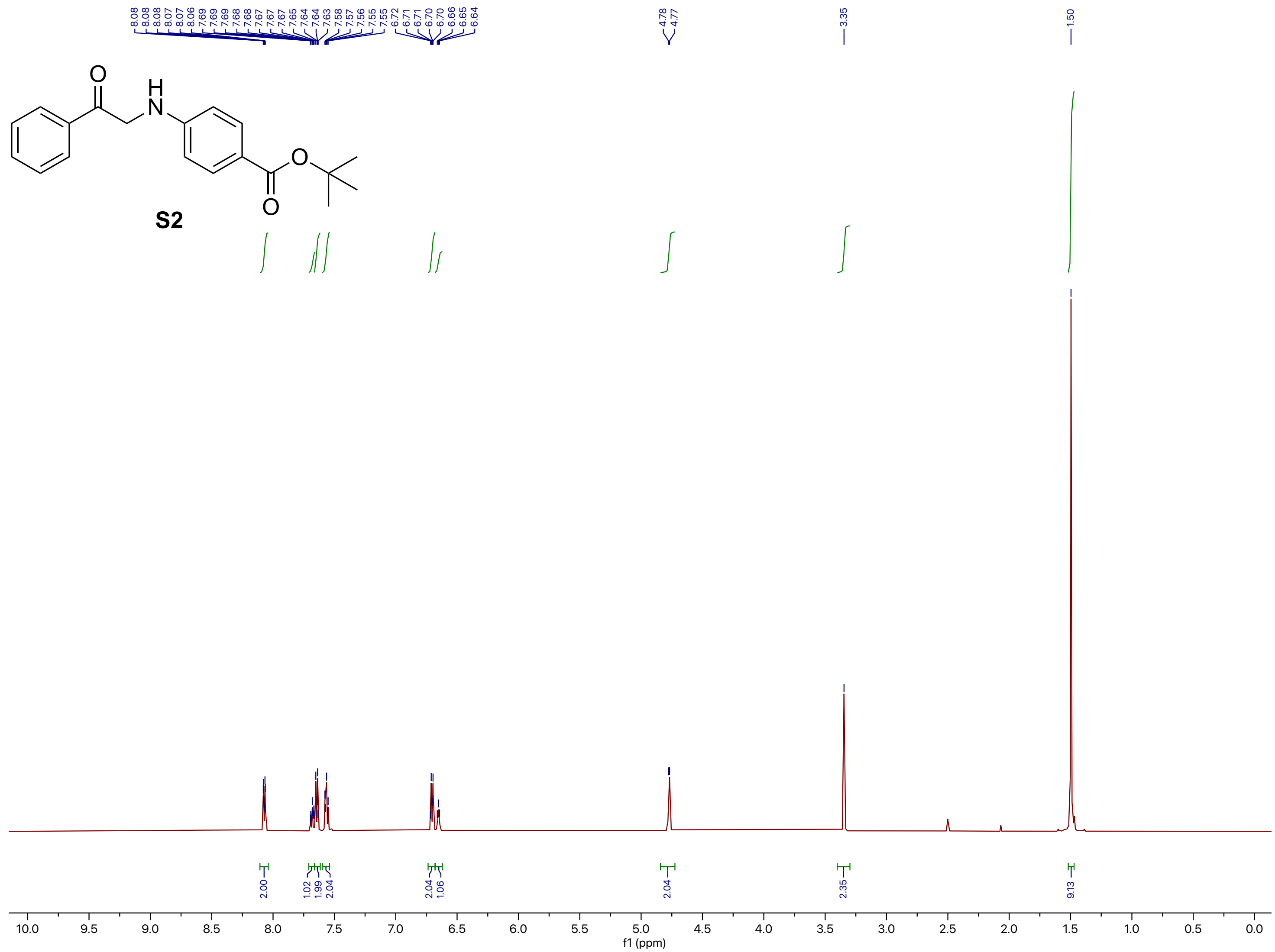

^{1}H-NMR (600 MHz, DMSO) of **S2**.

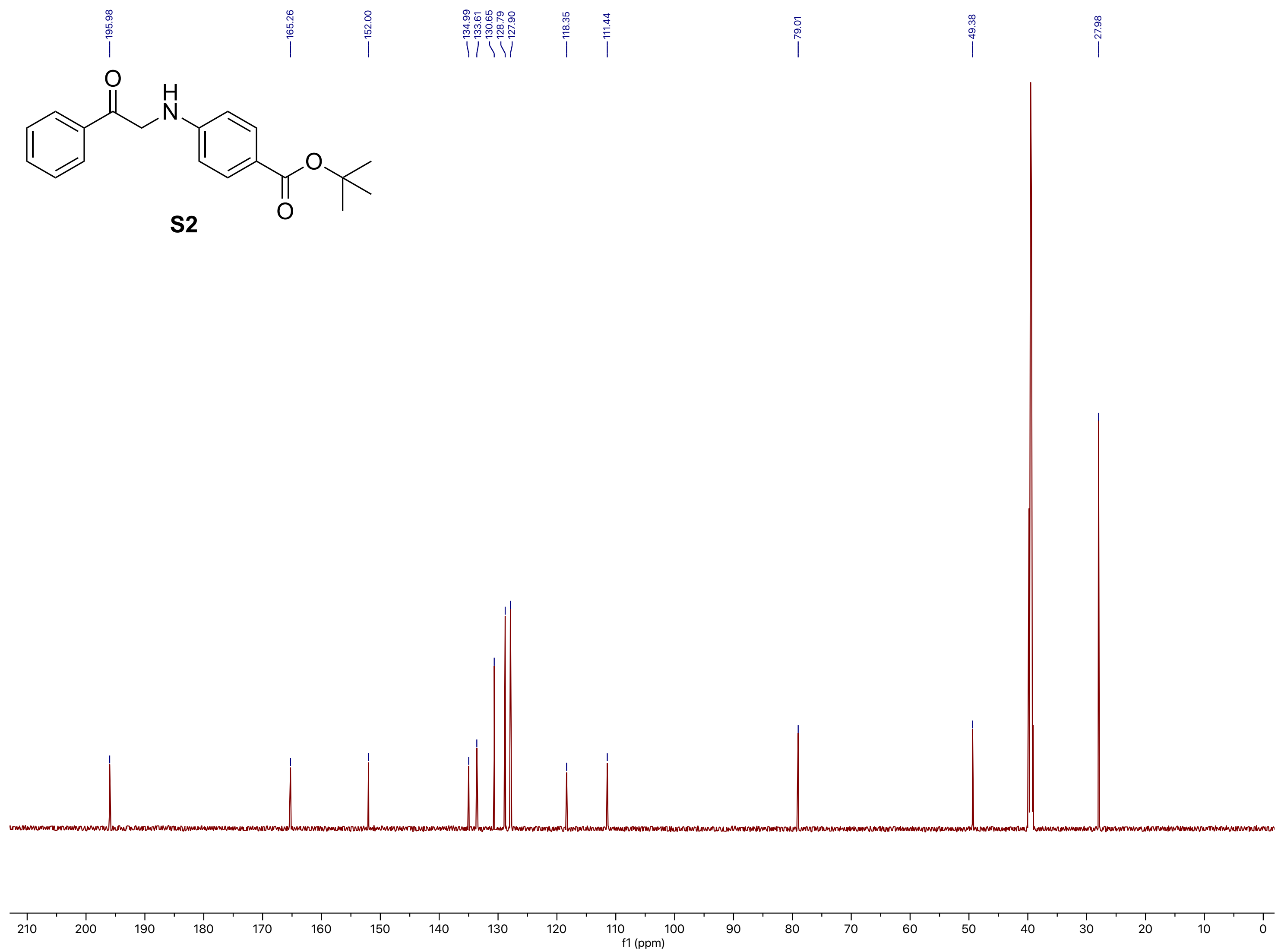

^{13}C-NMR (151 MHz, DMSO) of **S2**.

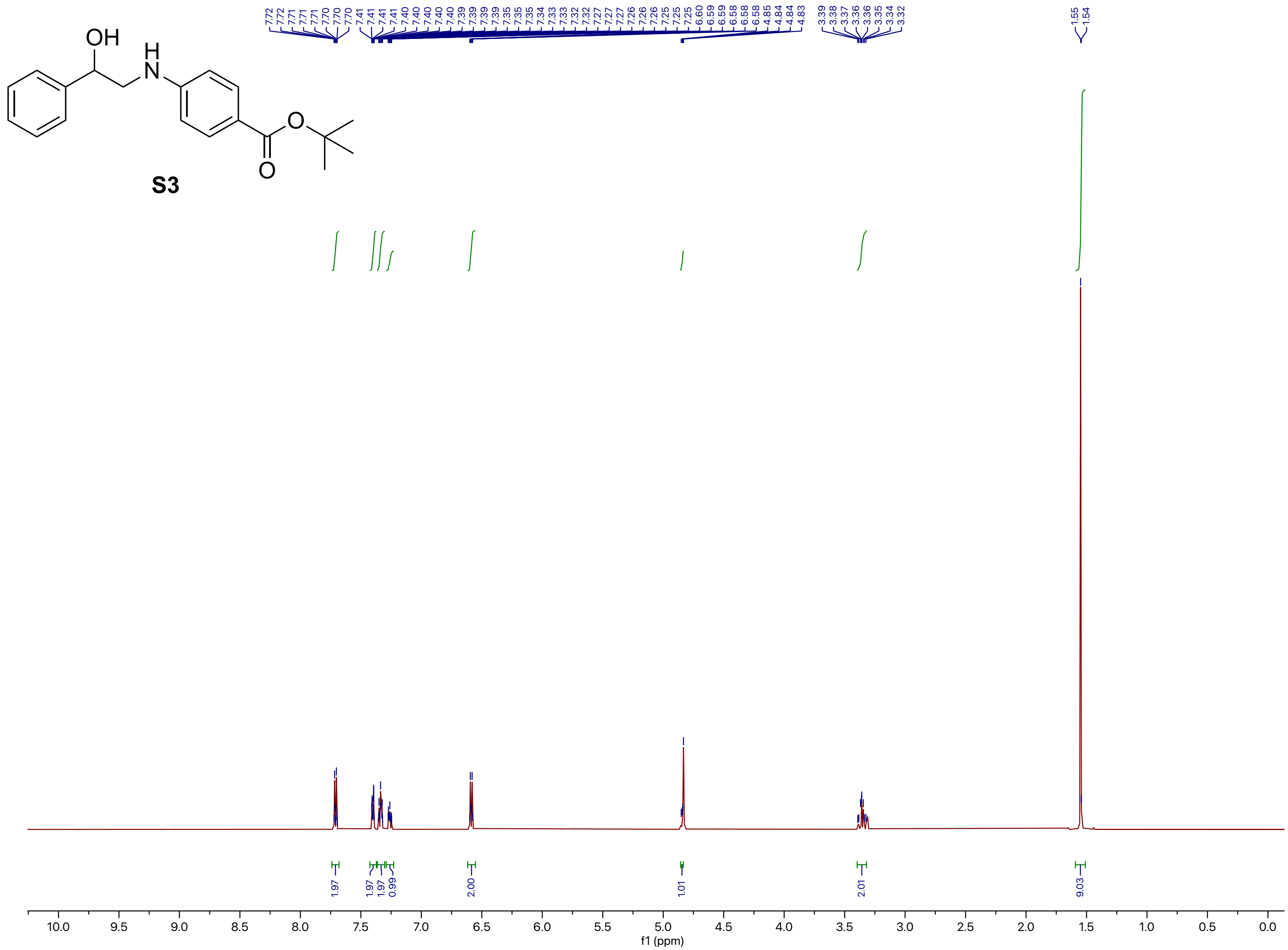

^{1}H-NMR (600 MHz, MeOD) of **S3**.

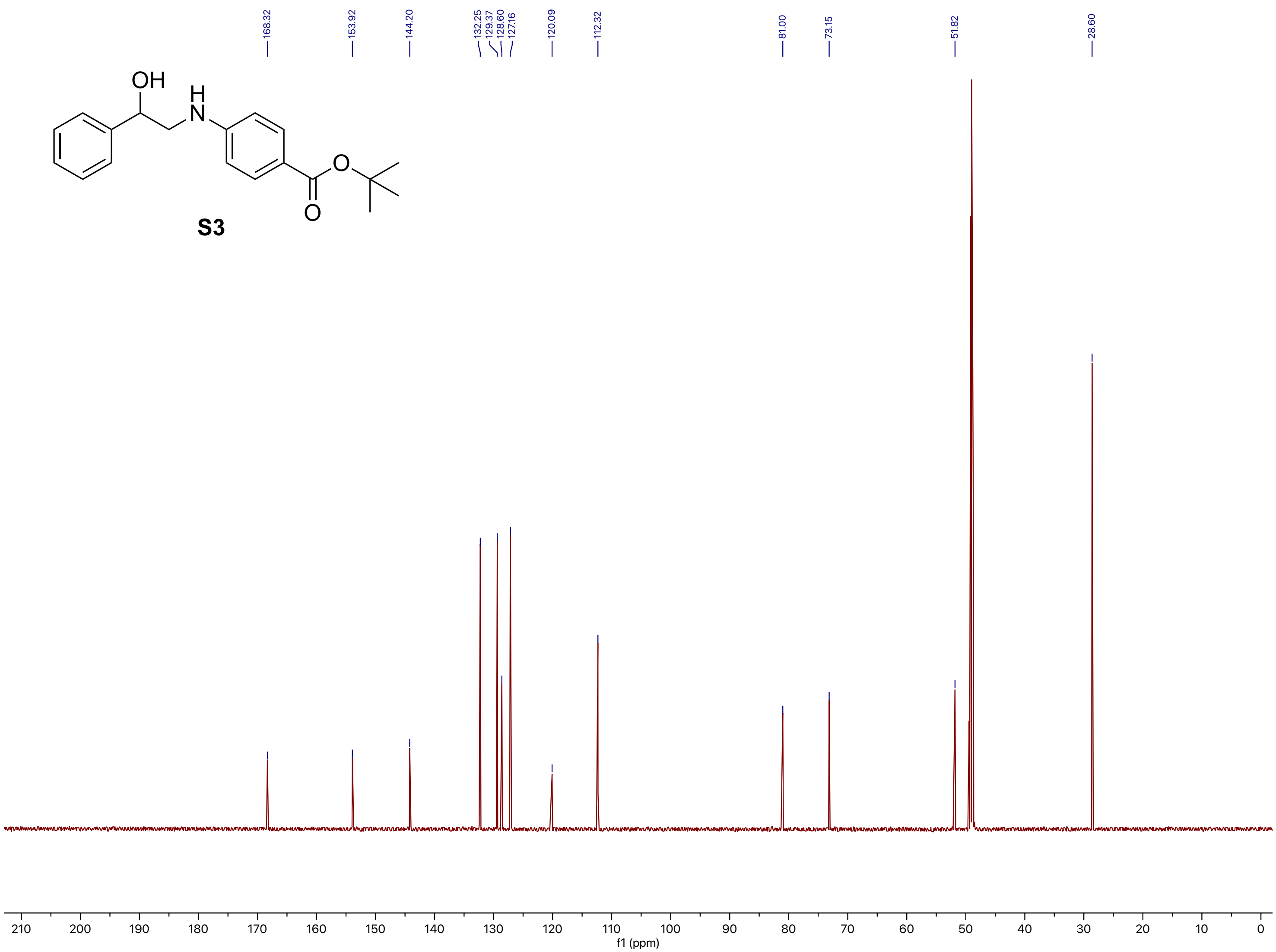

^{13}C-NMR (600 MHz, MeOD) of **S3**.

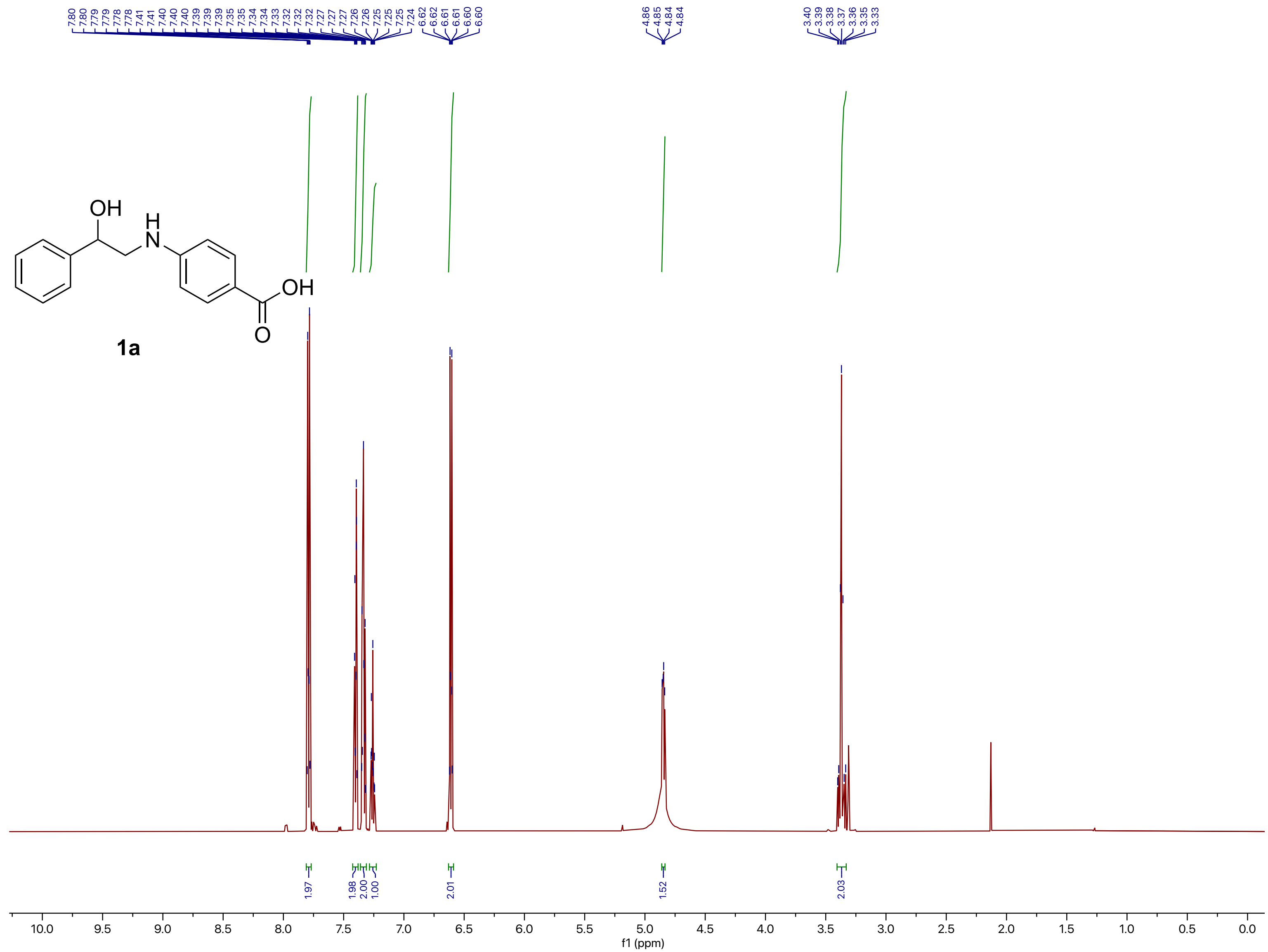

^{1}H-NMR (600 MHz, MeOD) of **1a**.

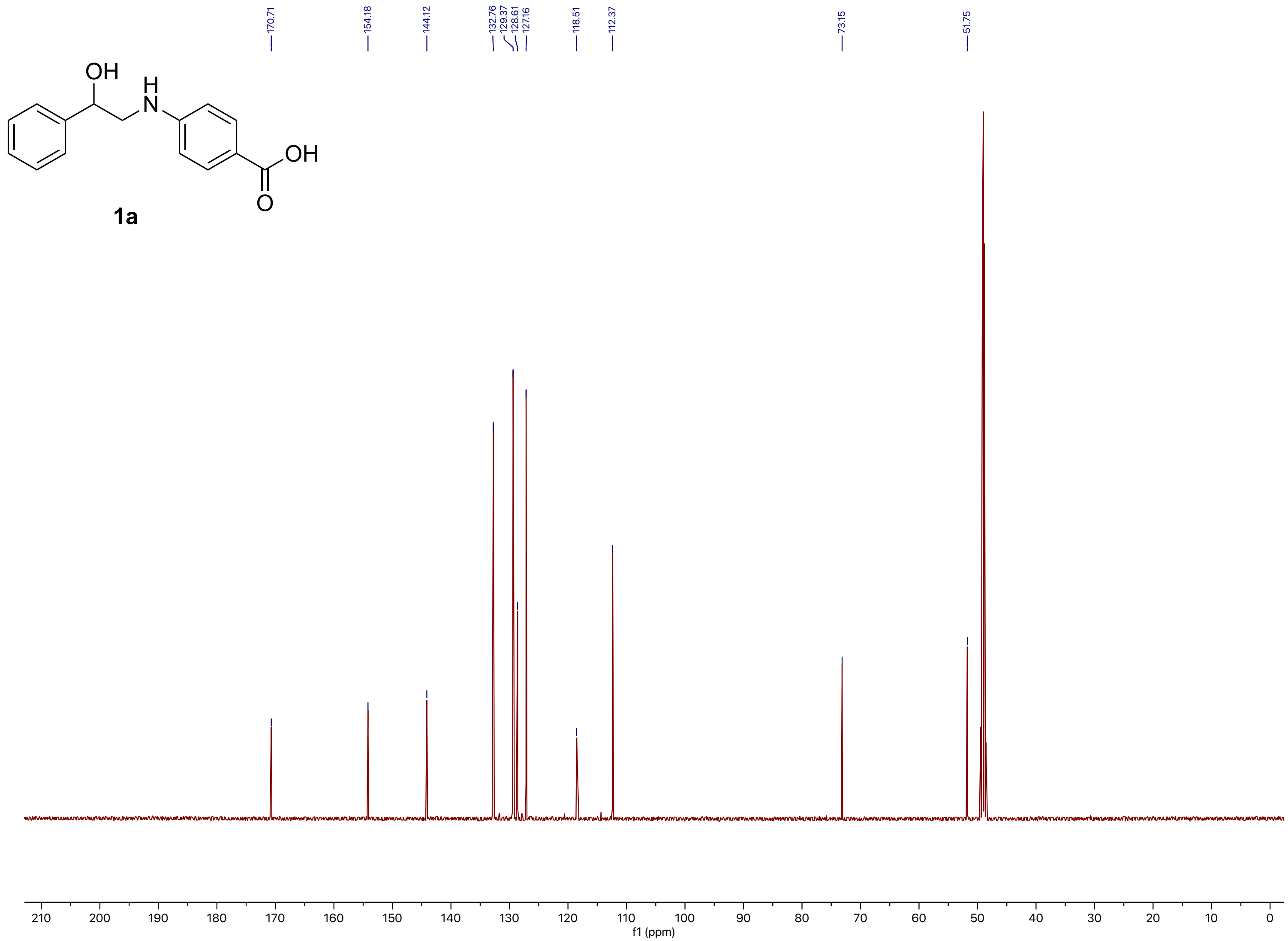

^{13}C-NMR (600 MHz, MeOD) of **1a**.

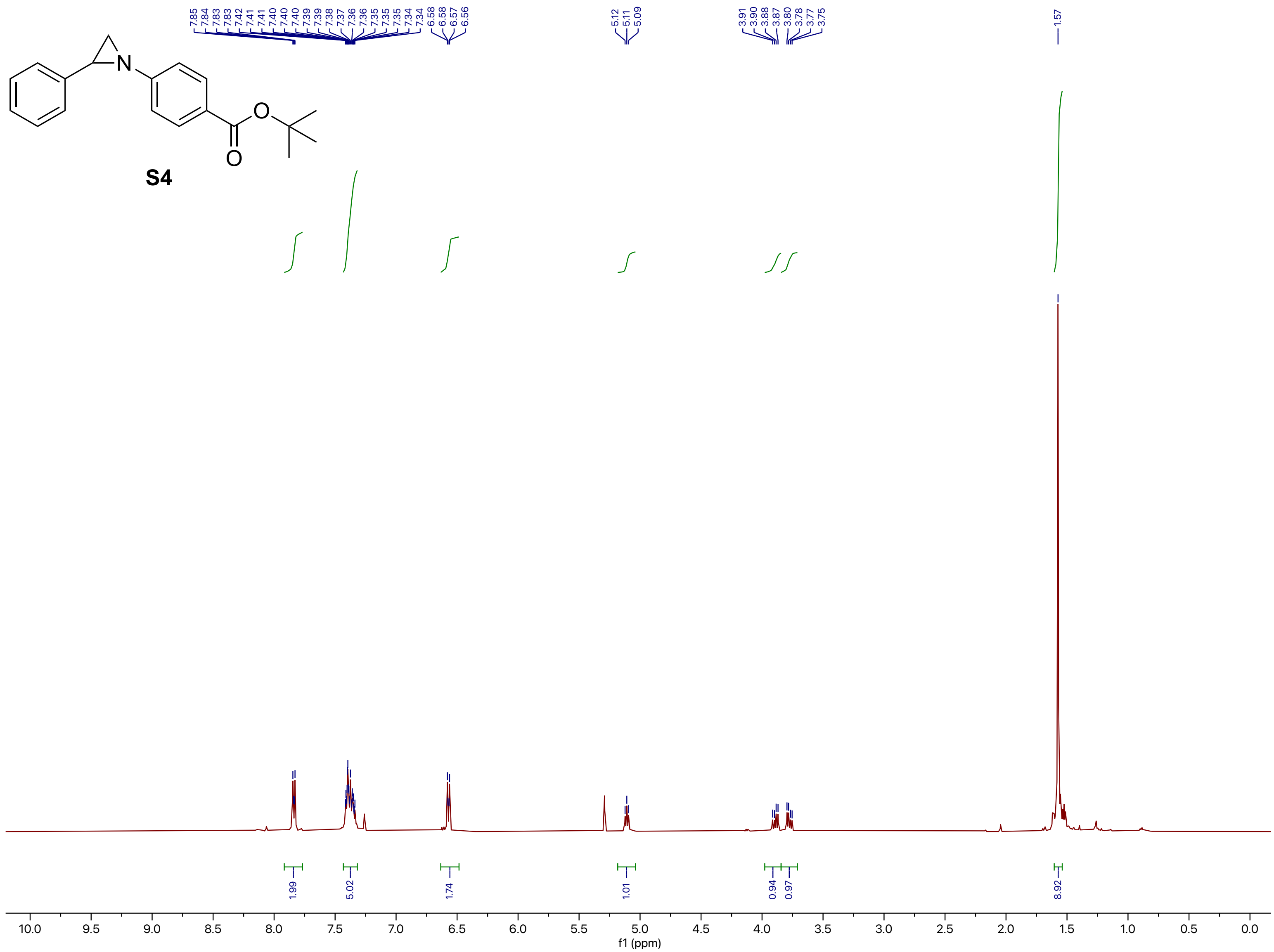

S4

^{1}H-NMR (500 MHz, $CDCl_3$) of **S4**. Compound is estimated to be ~90% pure. Impurities were removed by subsequent steps.

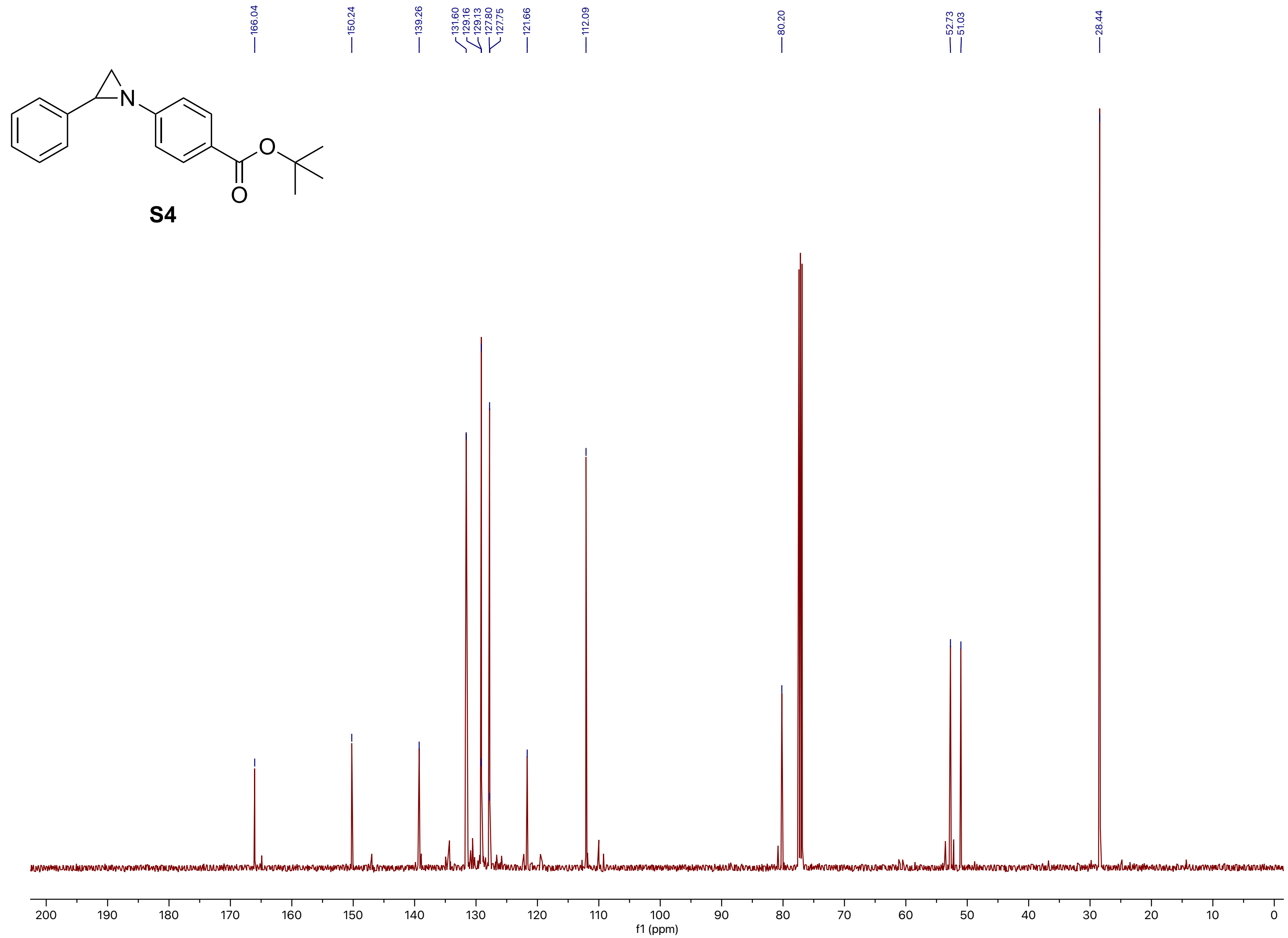

^{13}C-NMR (126 MHz, $CDCl_3$) of **S4**. Compound is estimated to be ~90% pure, Impurities were removed by subsequent steps.

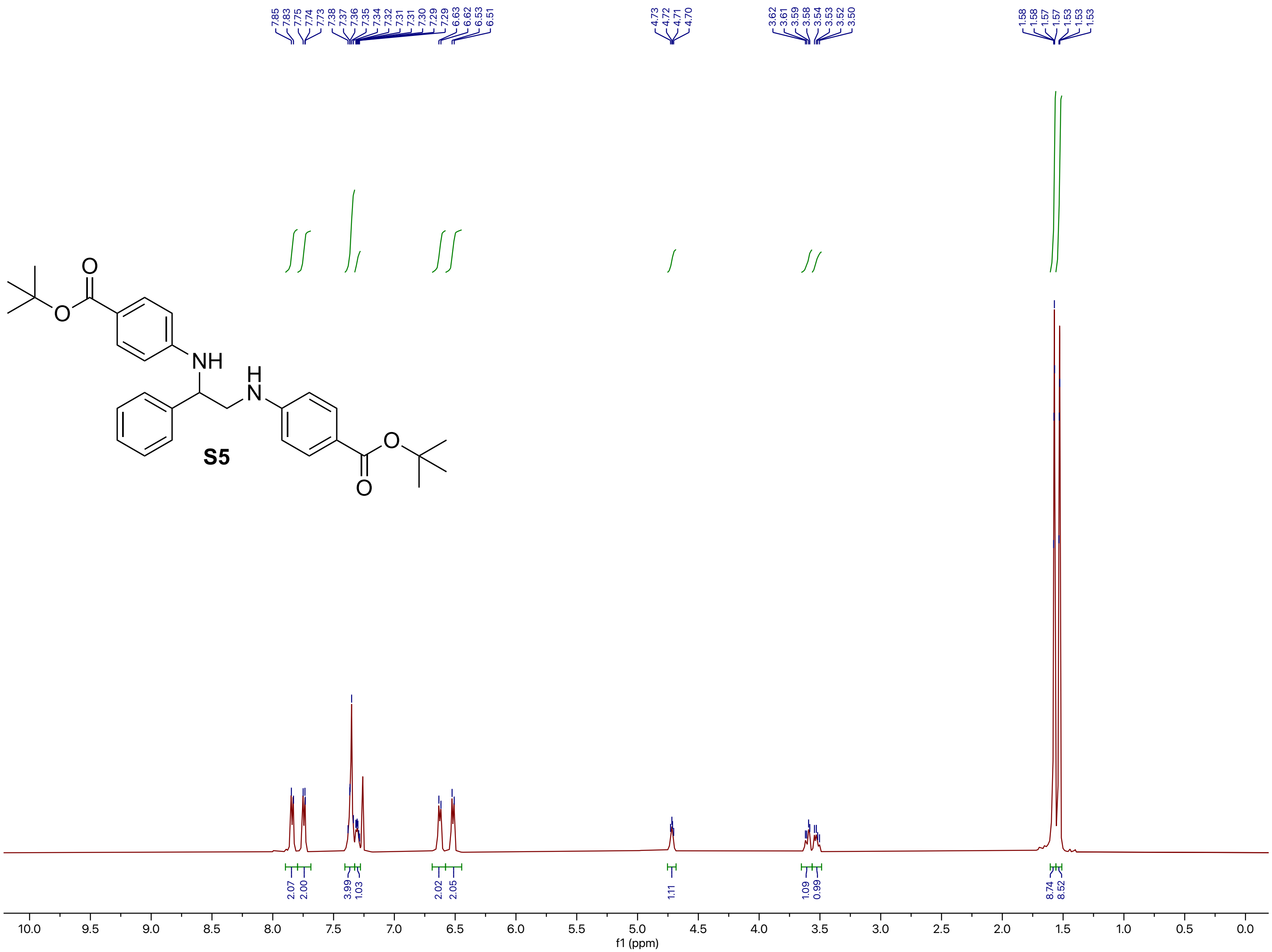

^{1}H-NMR (500 MHz, $CDCl_3$) of **S5**.

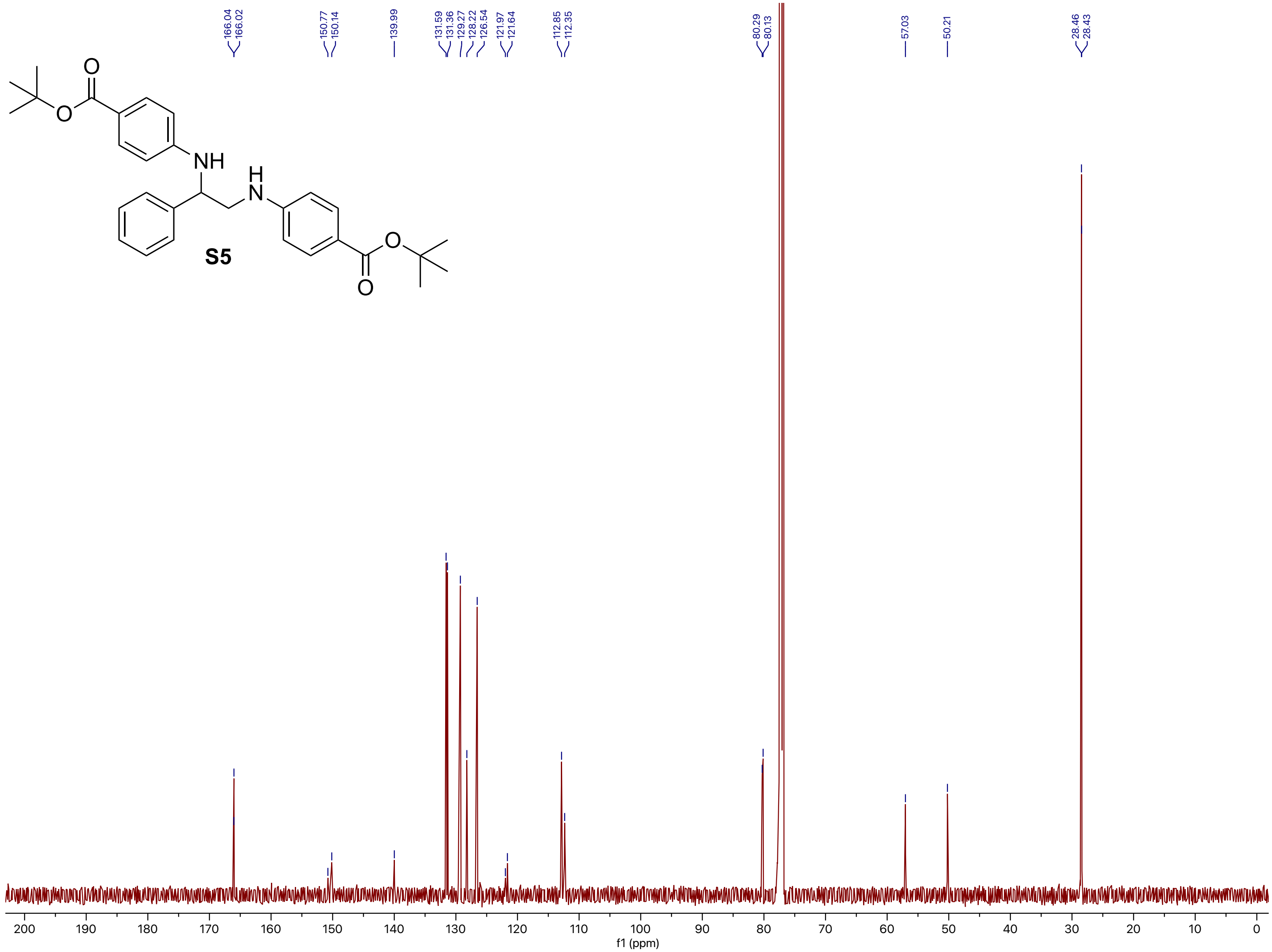

^{13}C-NMR (151 MHz, $CDCl_3$) of **S5**.

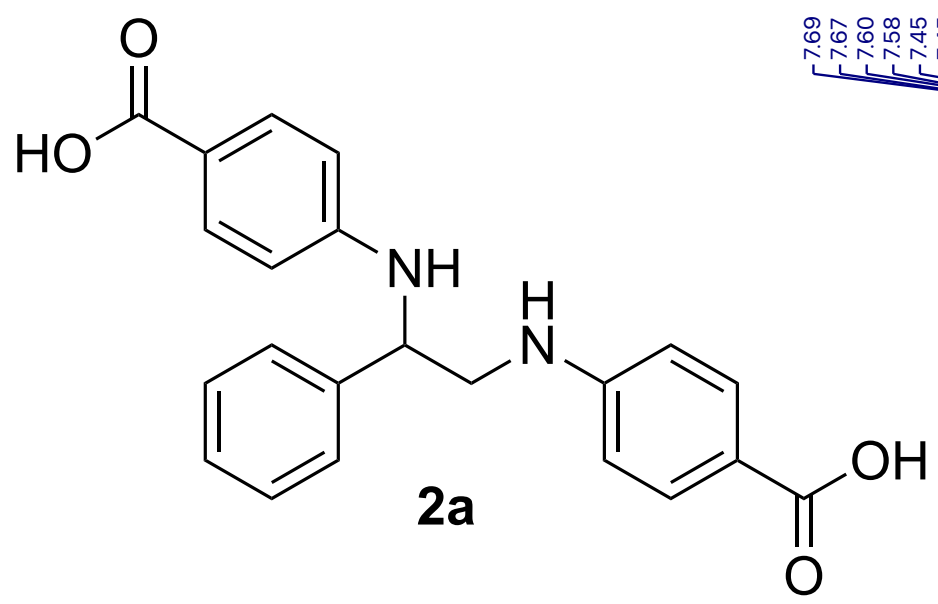

2a

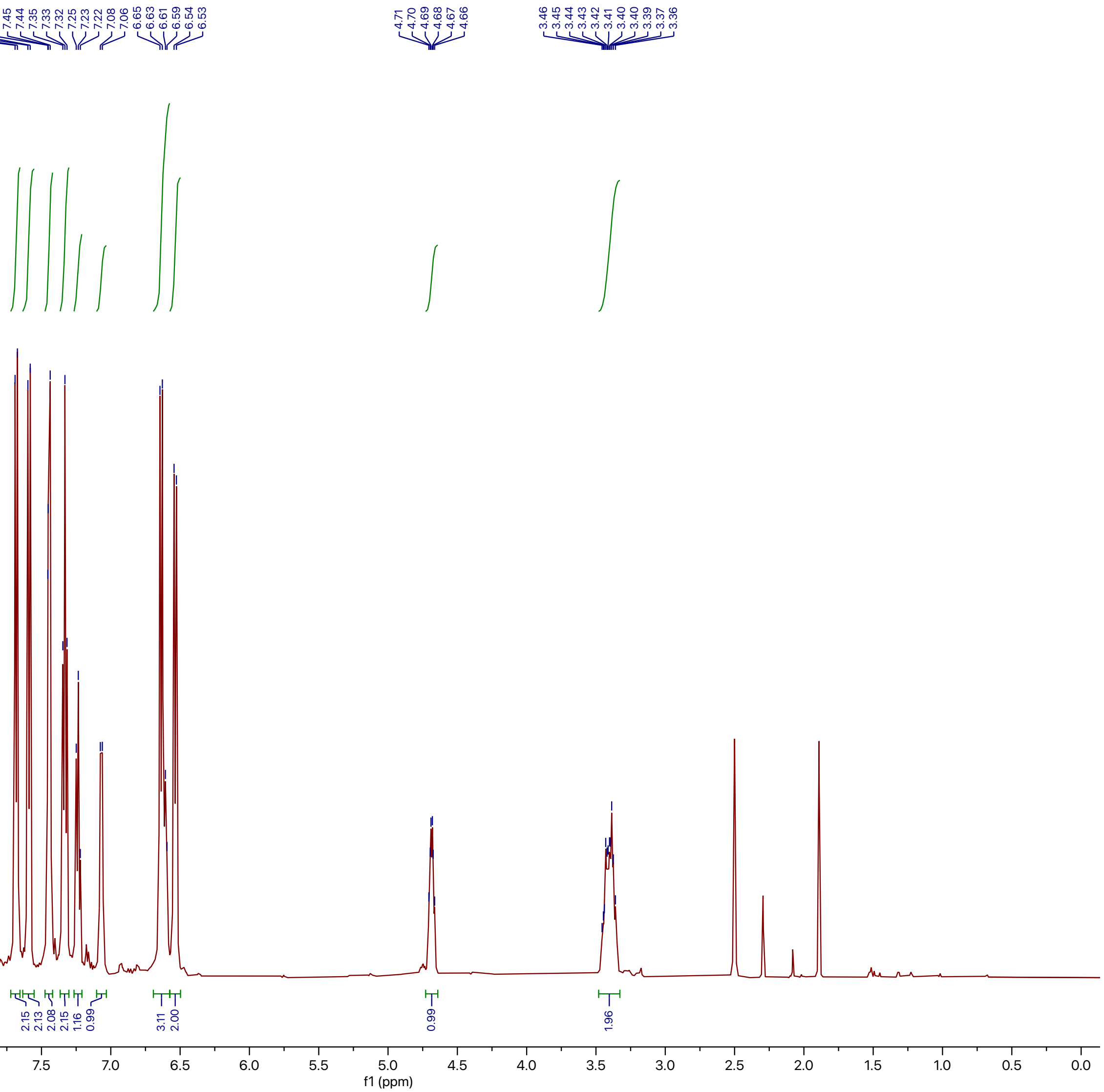

^{1}H-NMR (500 MHz, DMSO) of **2a**.

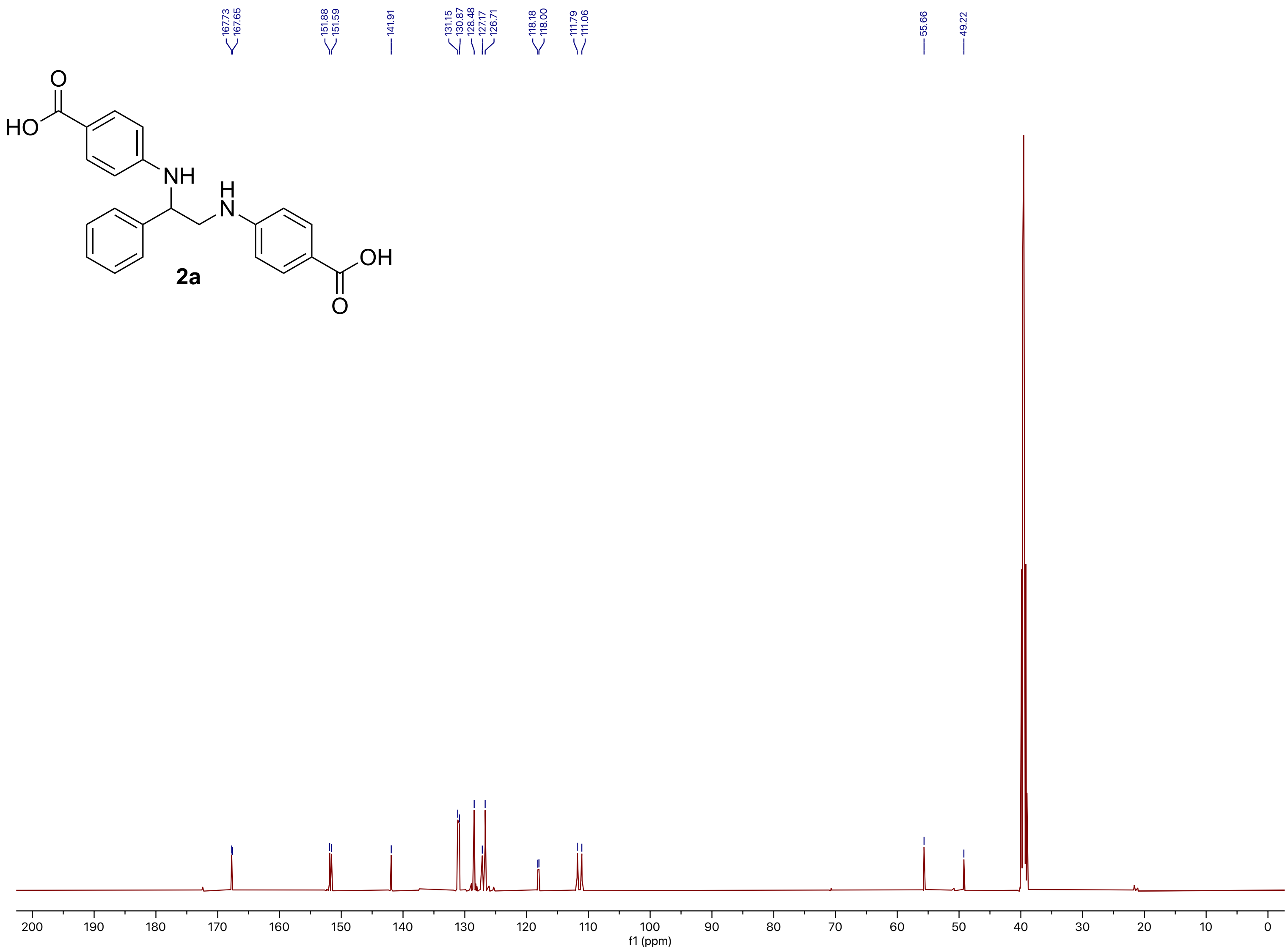

2a

^{13}C-NMR (126 MHz, DMSO) of **2a**.

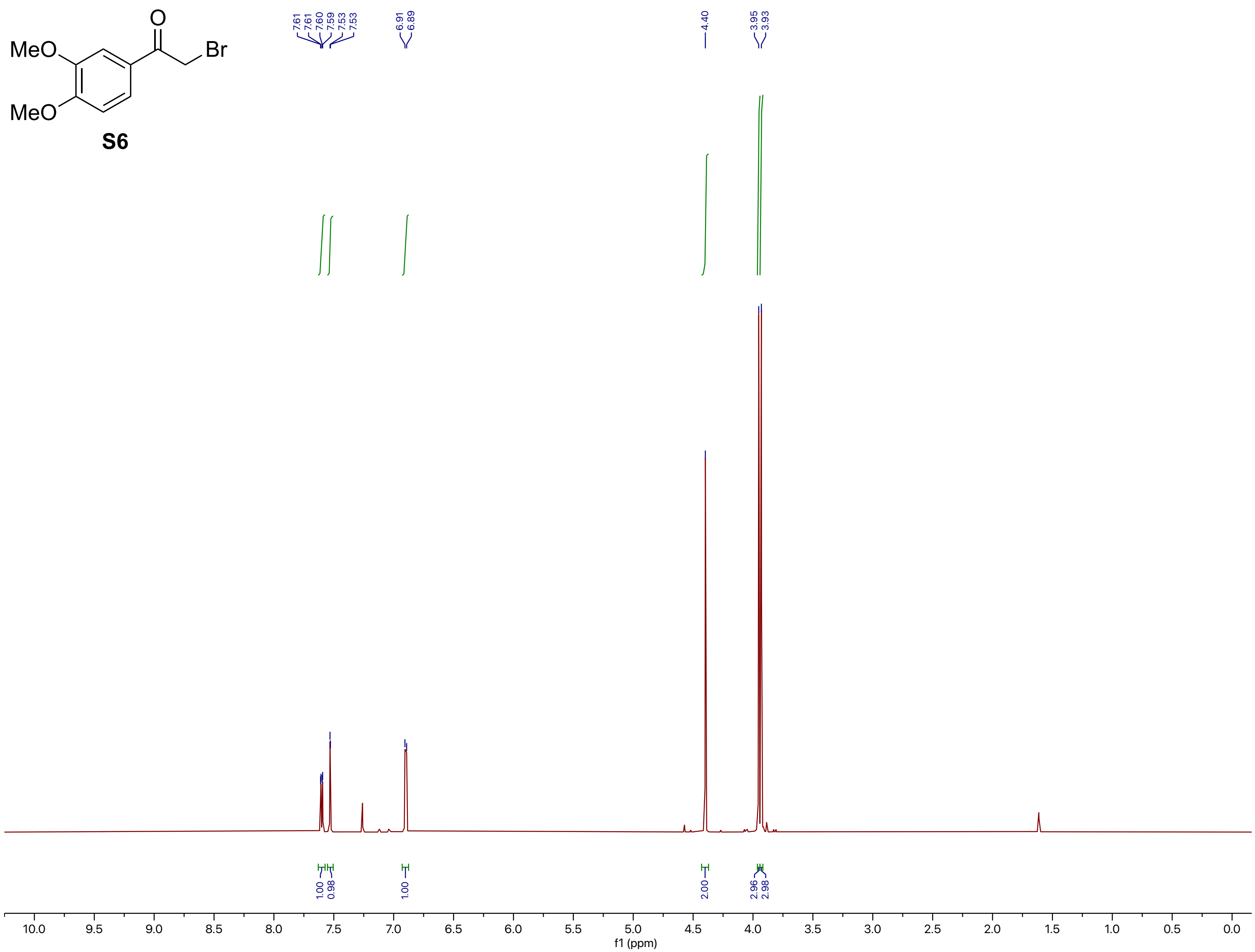

^{1}H-NMR (600 MHz, $CDCl_3$) of **S6**.

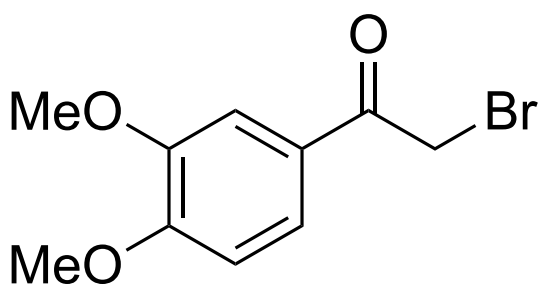

S6

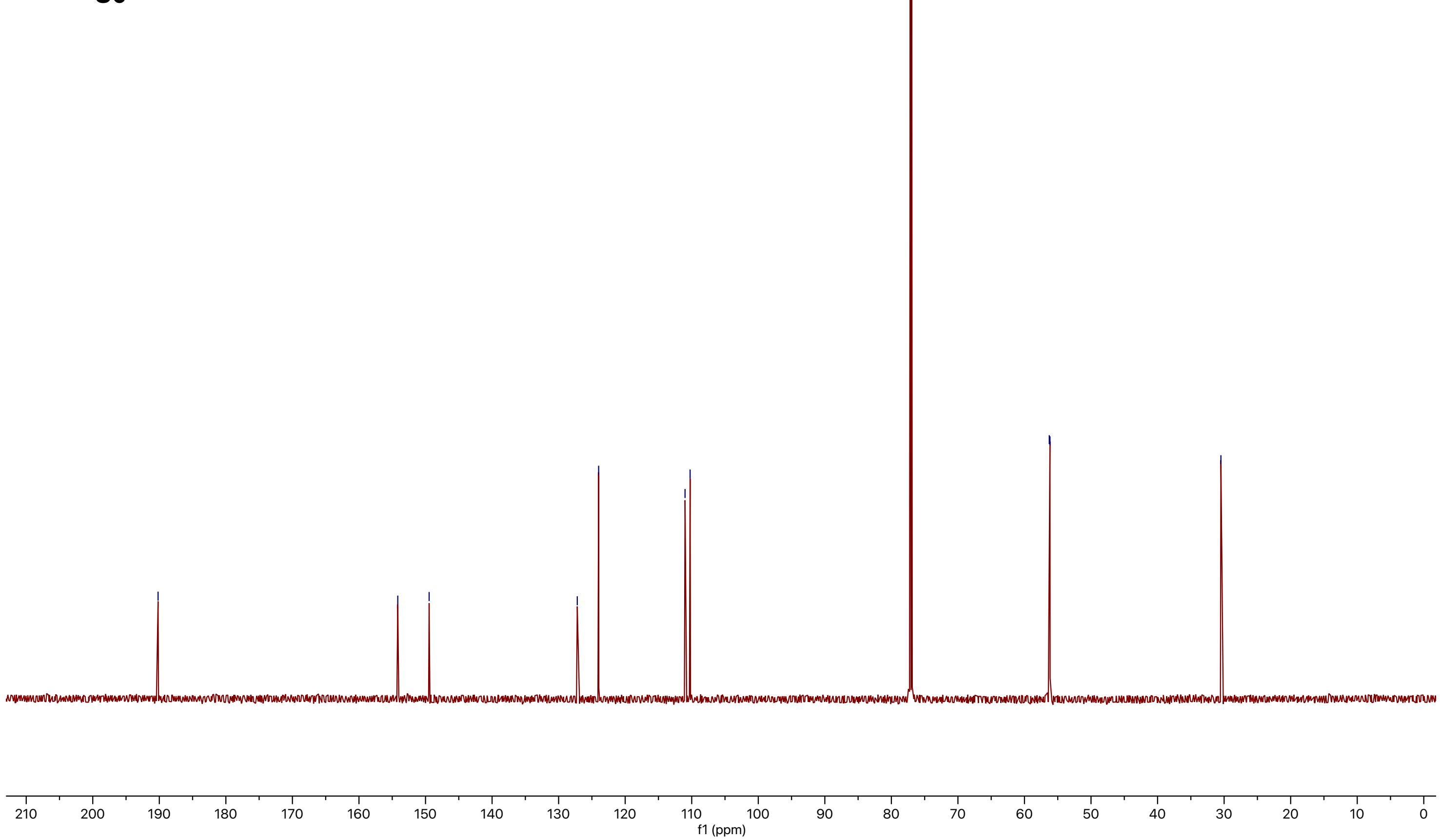

^{13}C-NMR (126 MHz, $CDCl_3$) of **S6**.

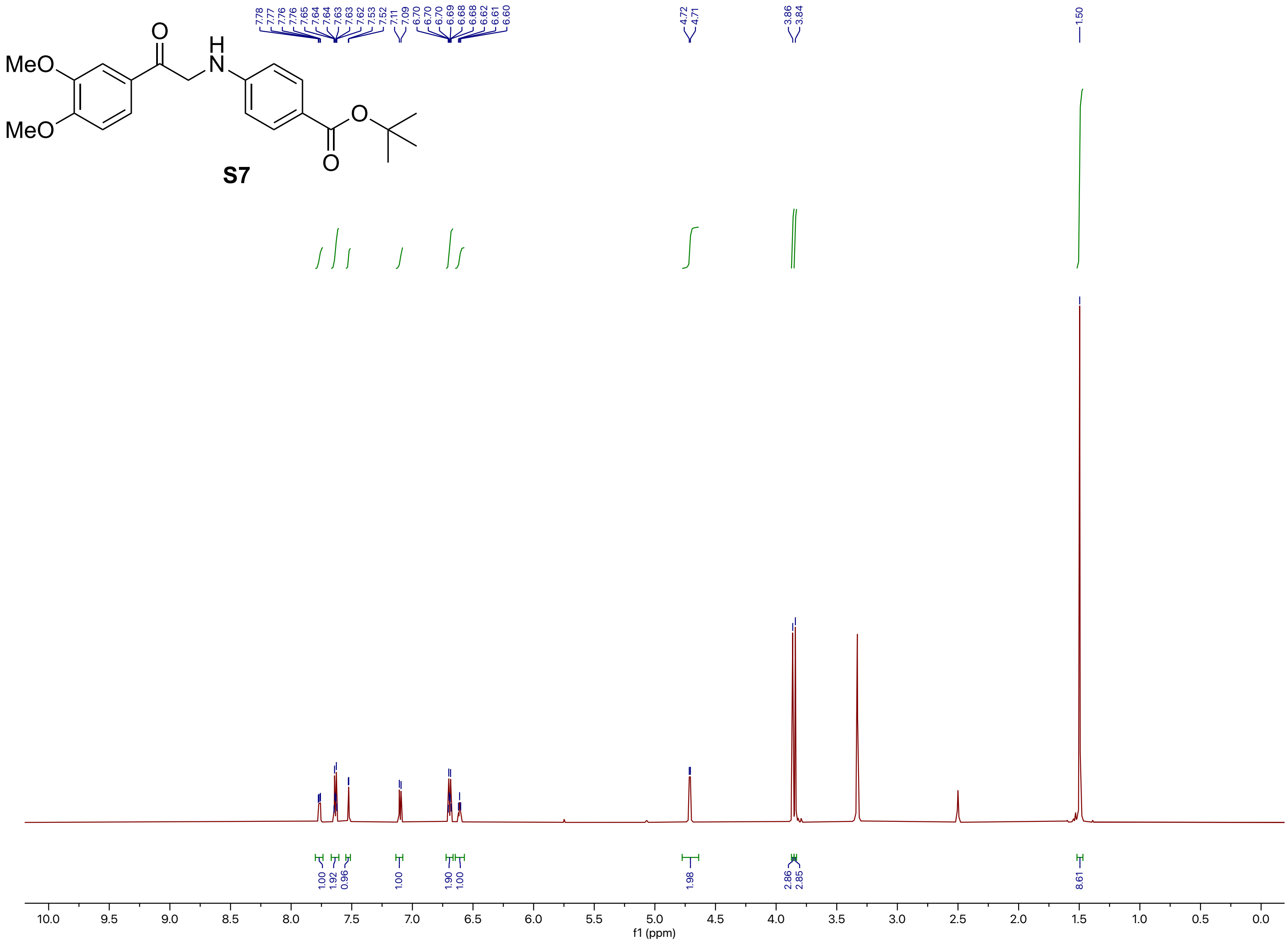

^{1}H-NMR (600 MHz, DMSO) of **S7**.

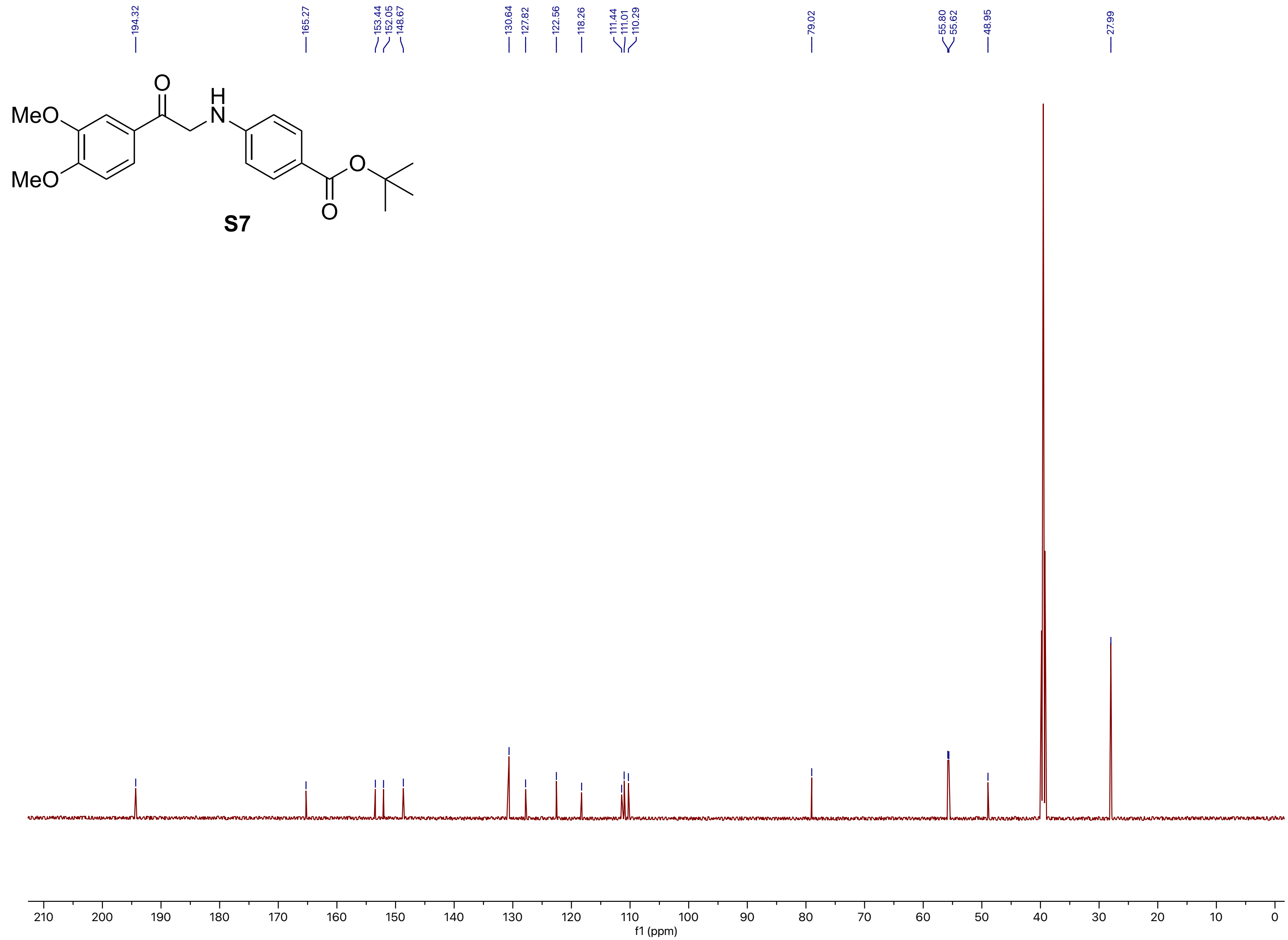

^{13}C-NMR (151 MHz, DMSO) of **S7**.

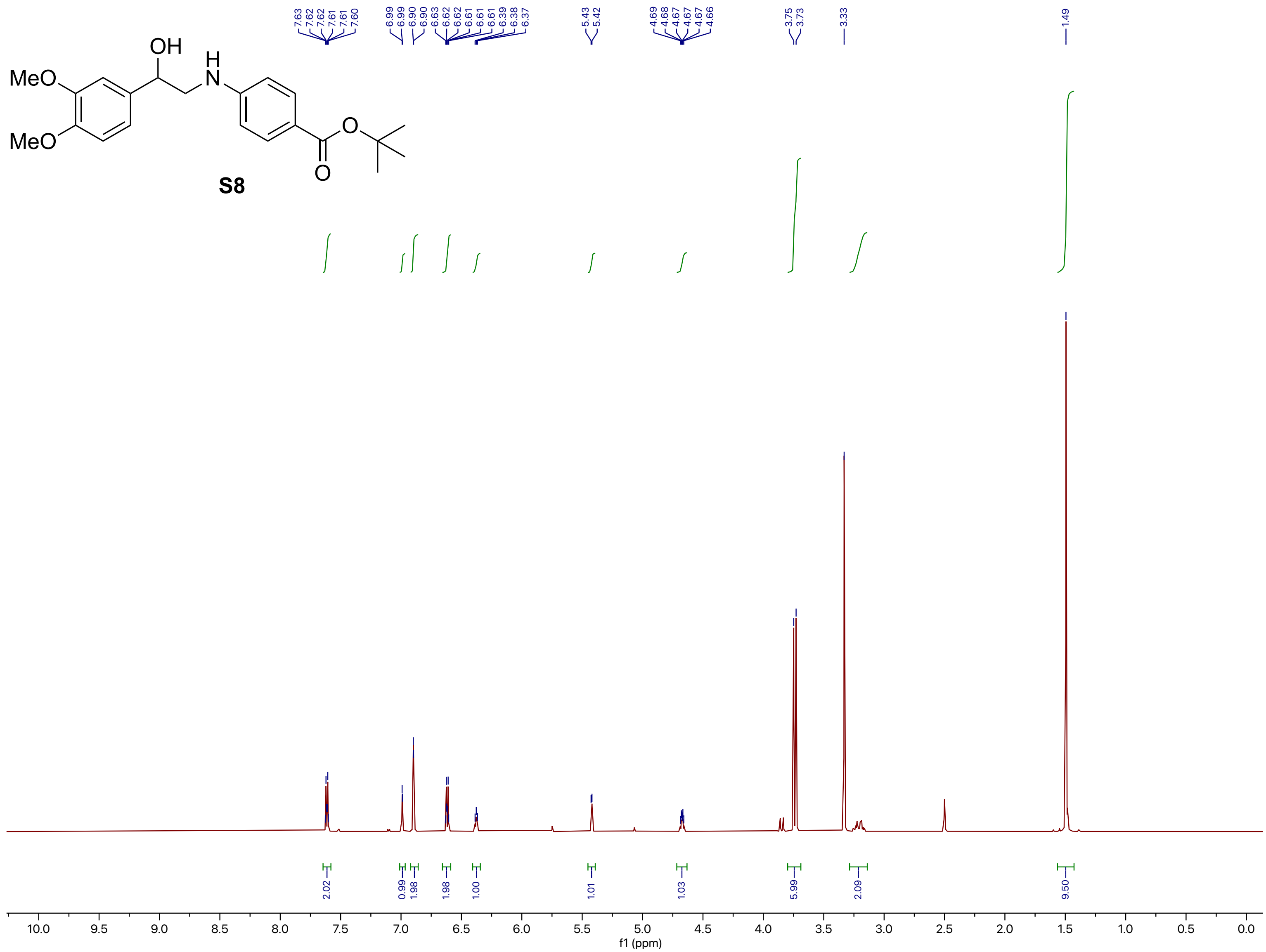

^{1}H-NMR (600 MHz, DMSO) of **S8**.

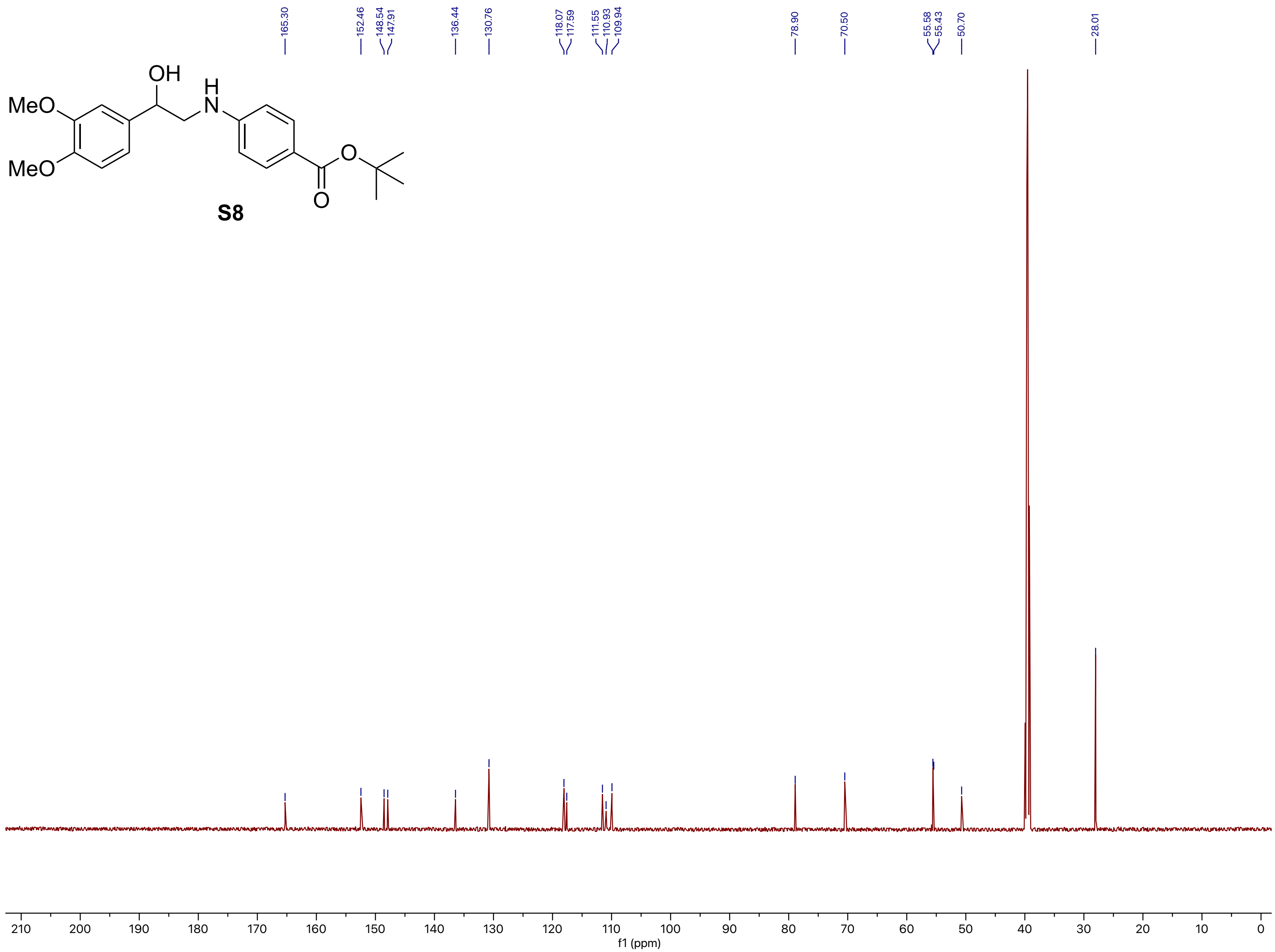

^{13}C-NMR (151 MHz, DMSO) of **S8**.

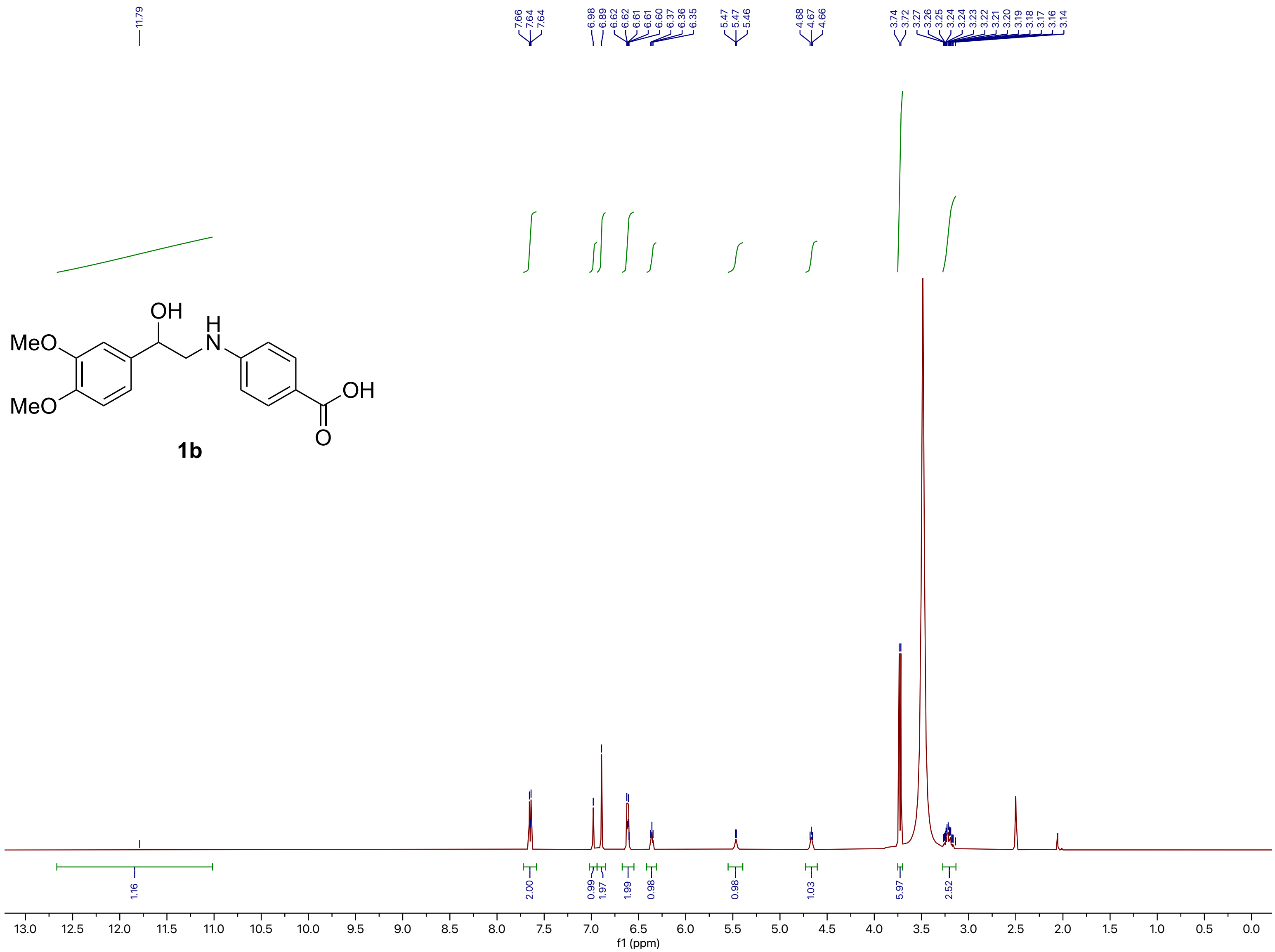

^{1}H-NMR (500 MHz, DMSO) of **1b**.

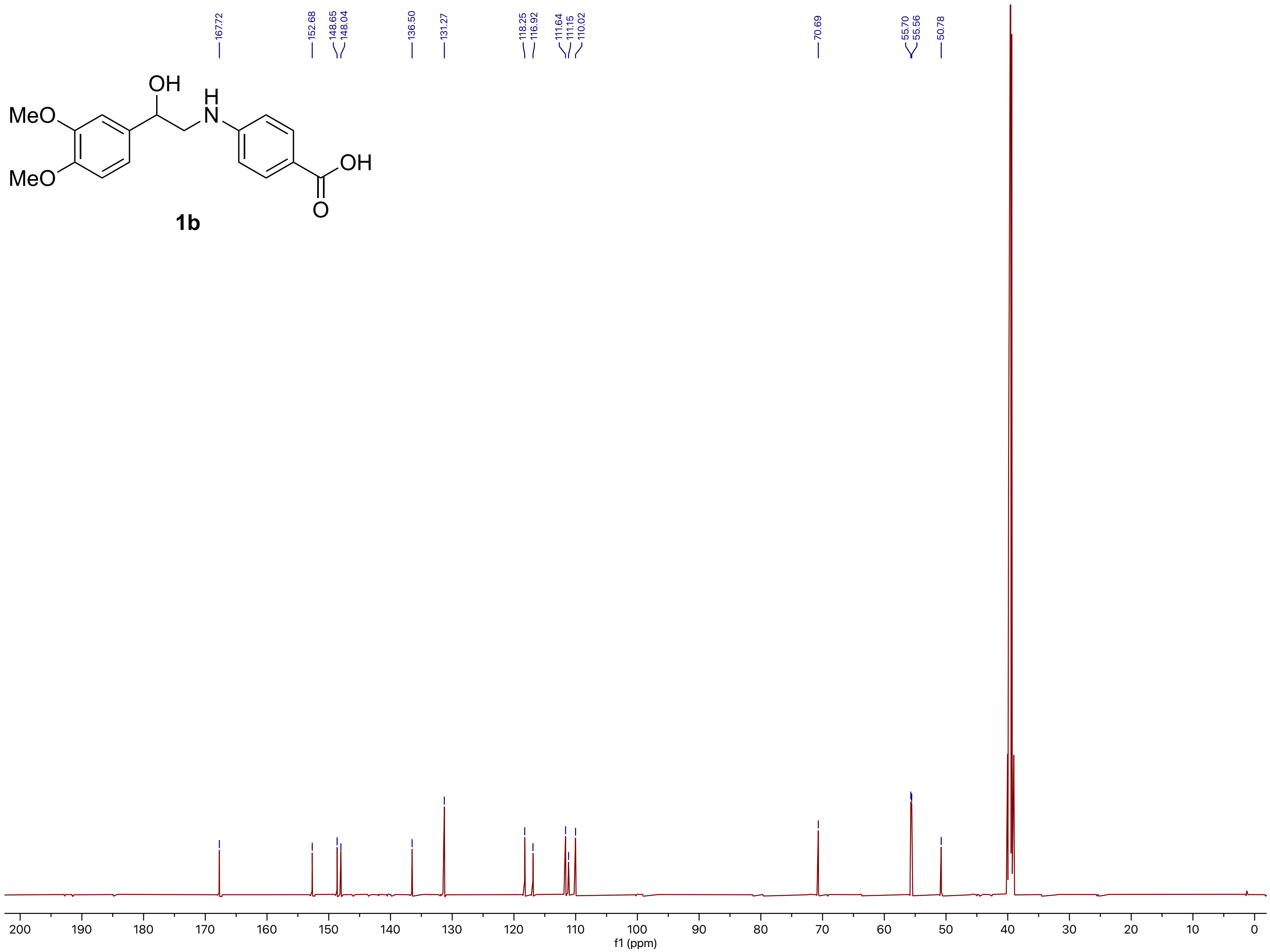

^{1}H-NMR (126 MHz, DMSO) of **1b**.

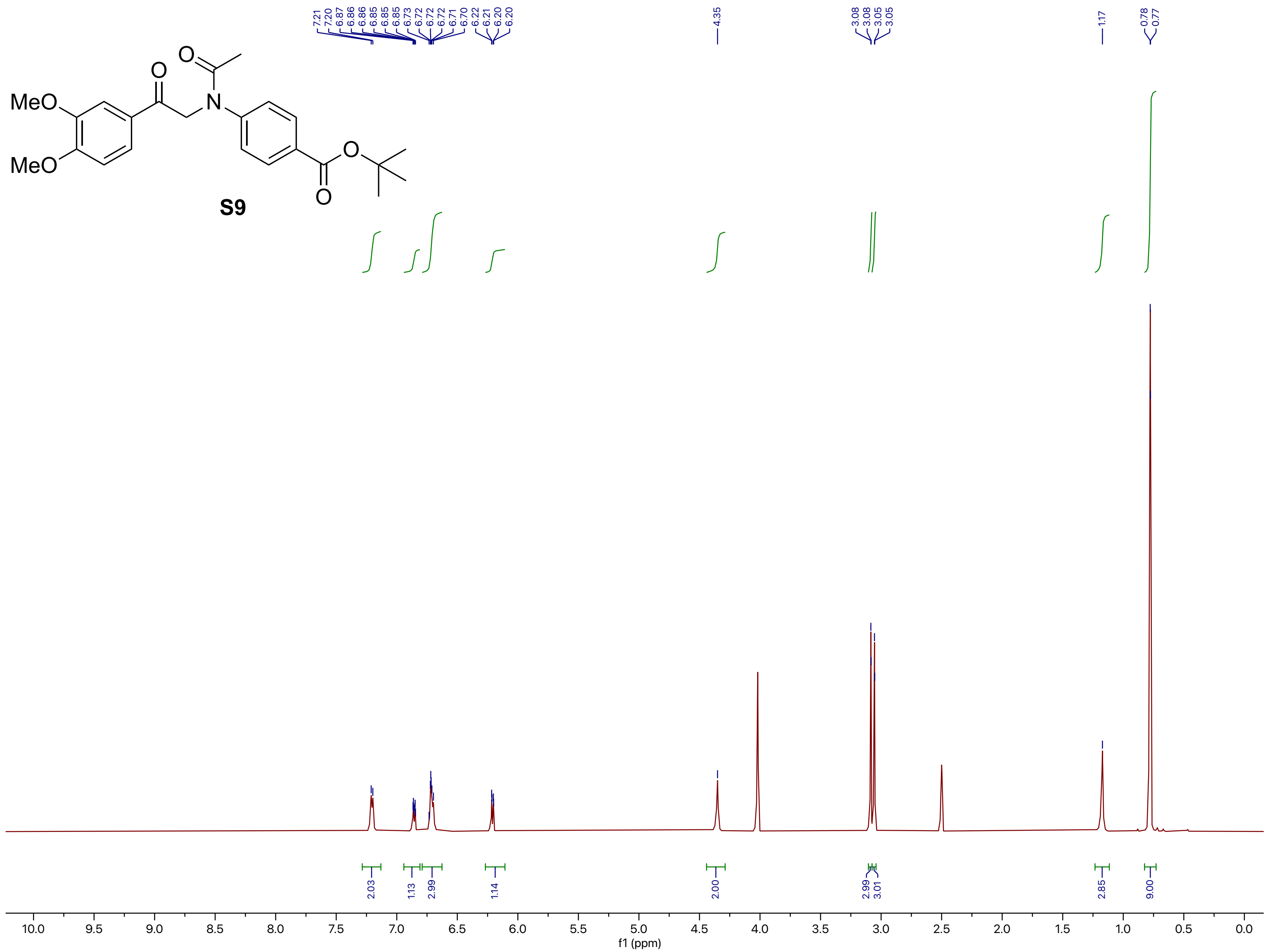

^{1}H-NMR (600 MHz, DMSO) of **S9**.

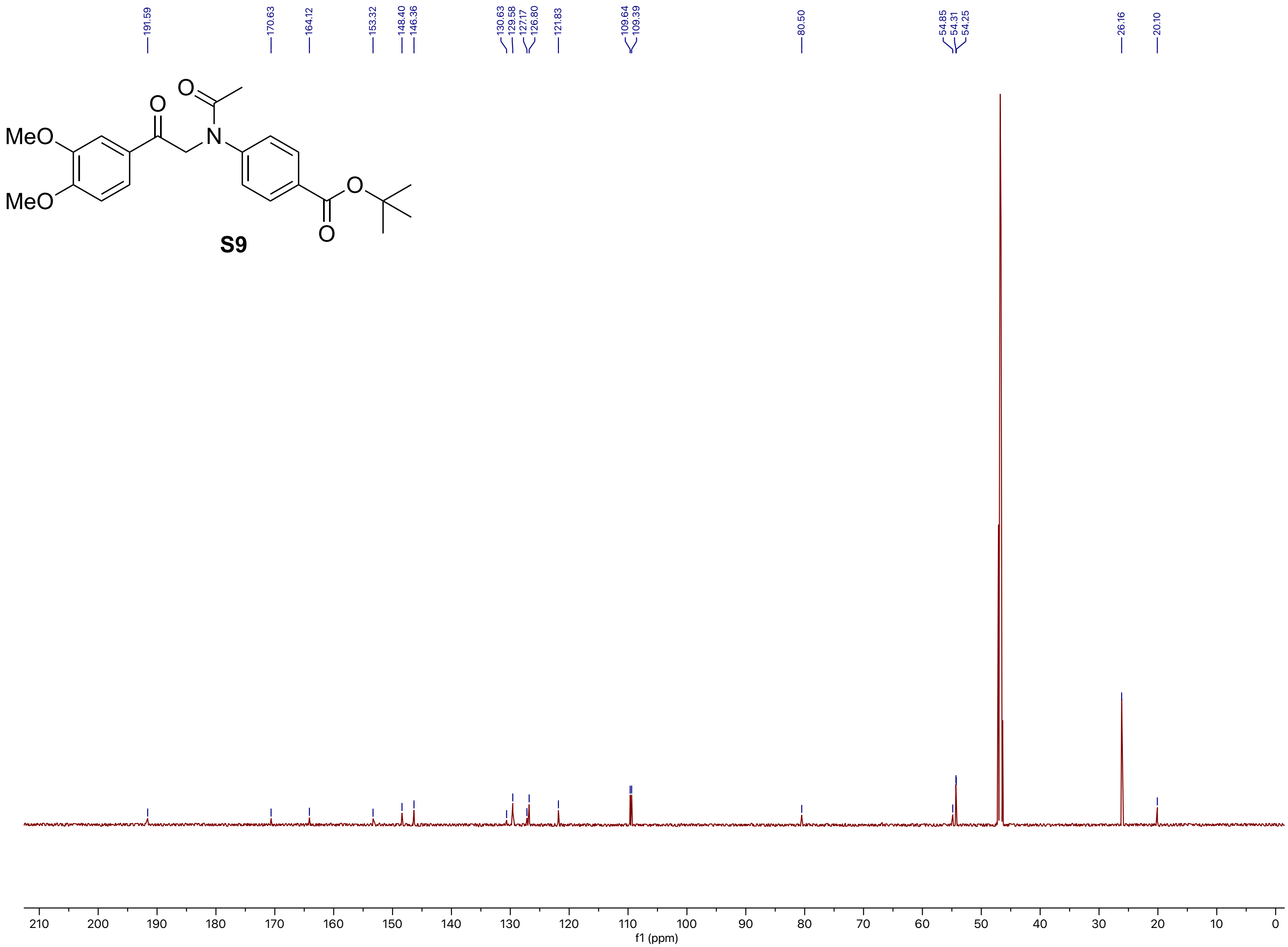

^{13}C-NMR (151 MHz, DMSO) of **S9**.

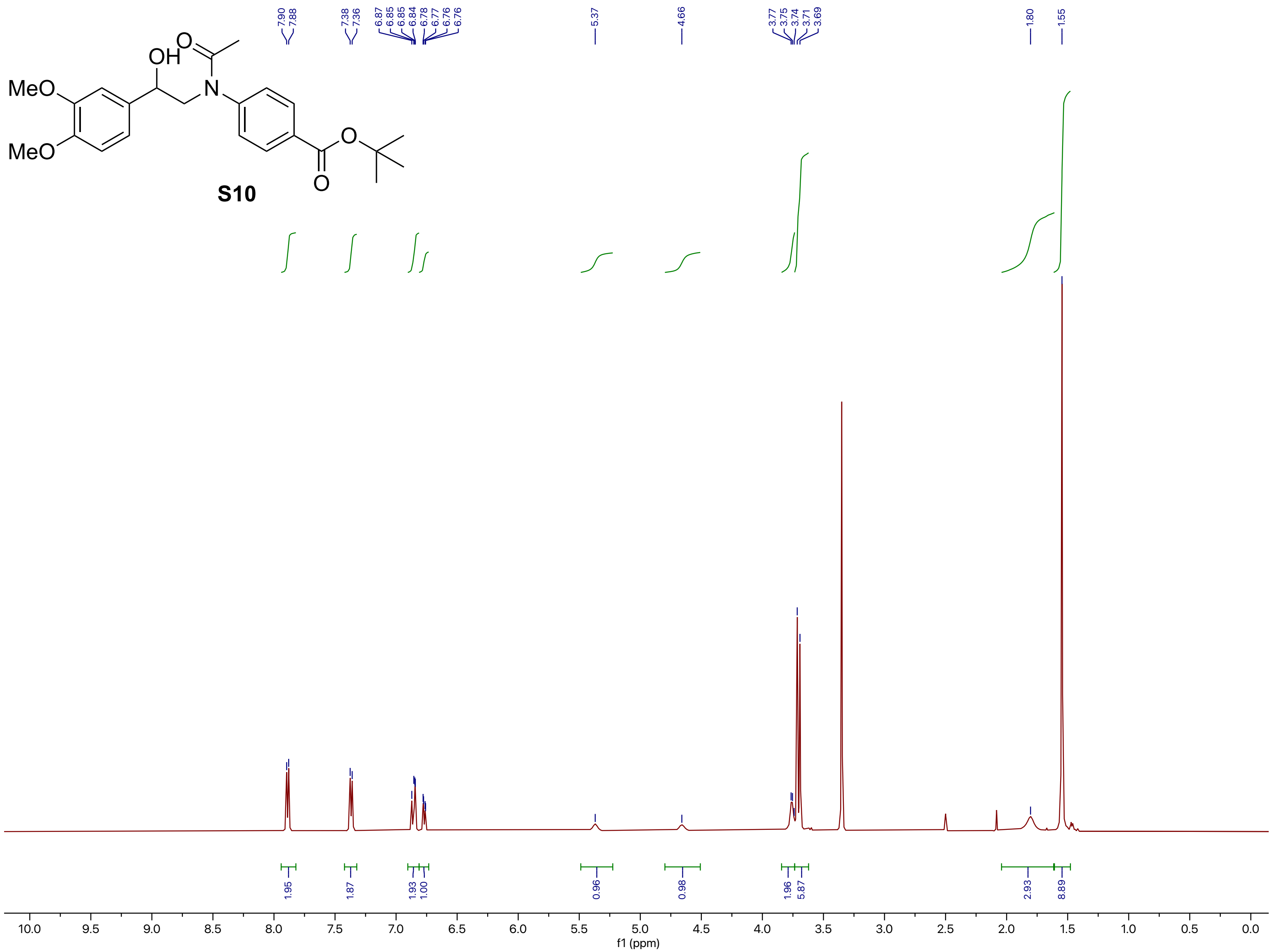

S10

^{1}H-NMR (500 MHz, DMSO) of **S10**.

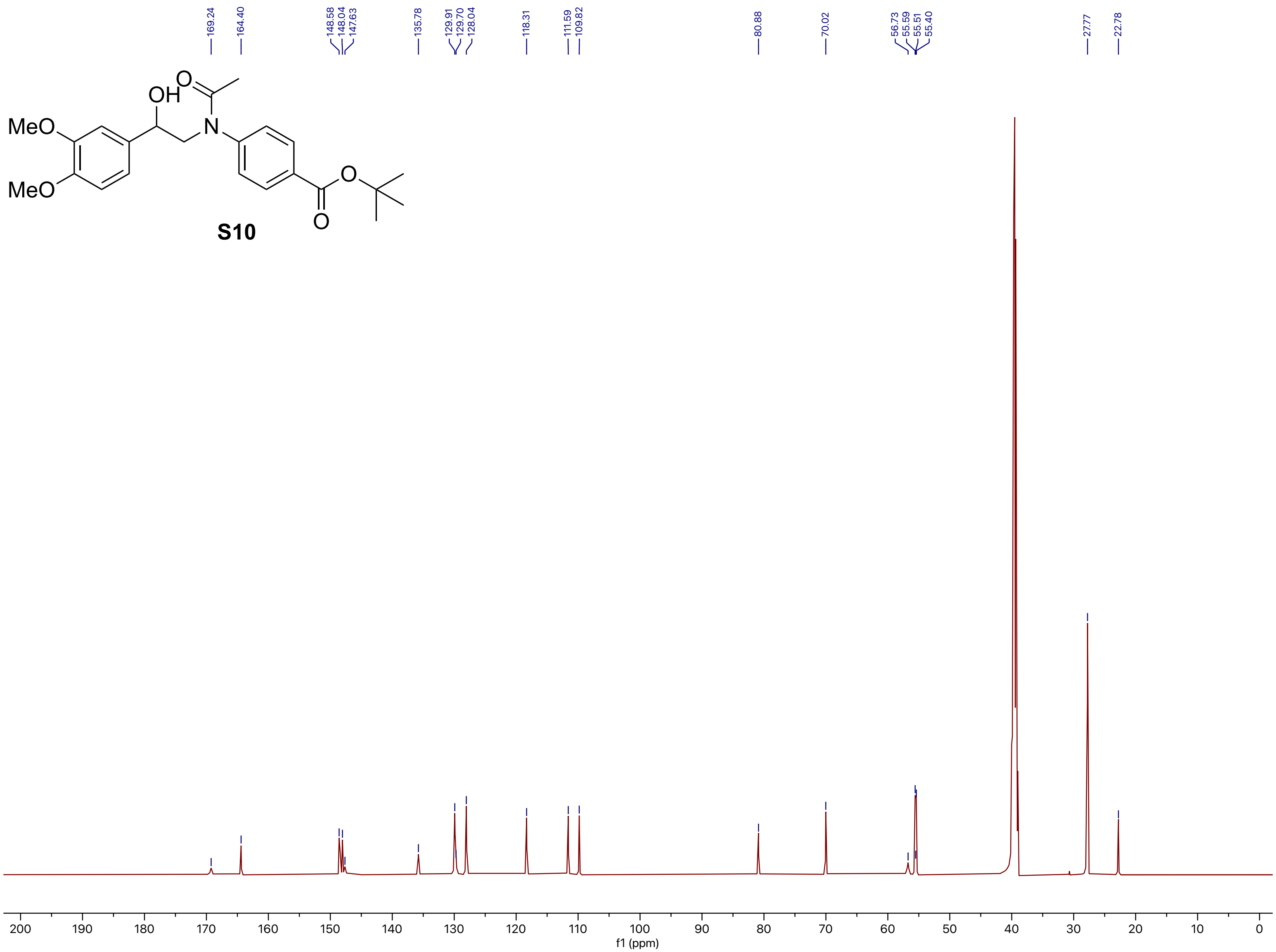

^{13}C-NMR (126 MHz, DMSO) of **S10**.

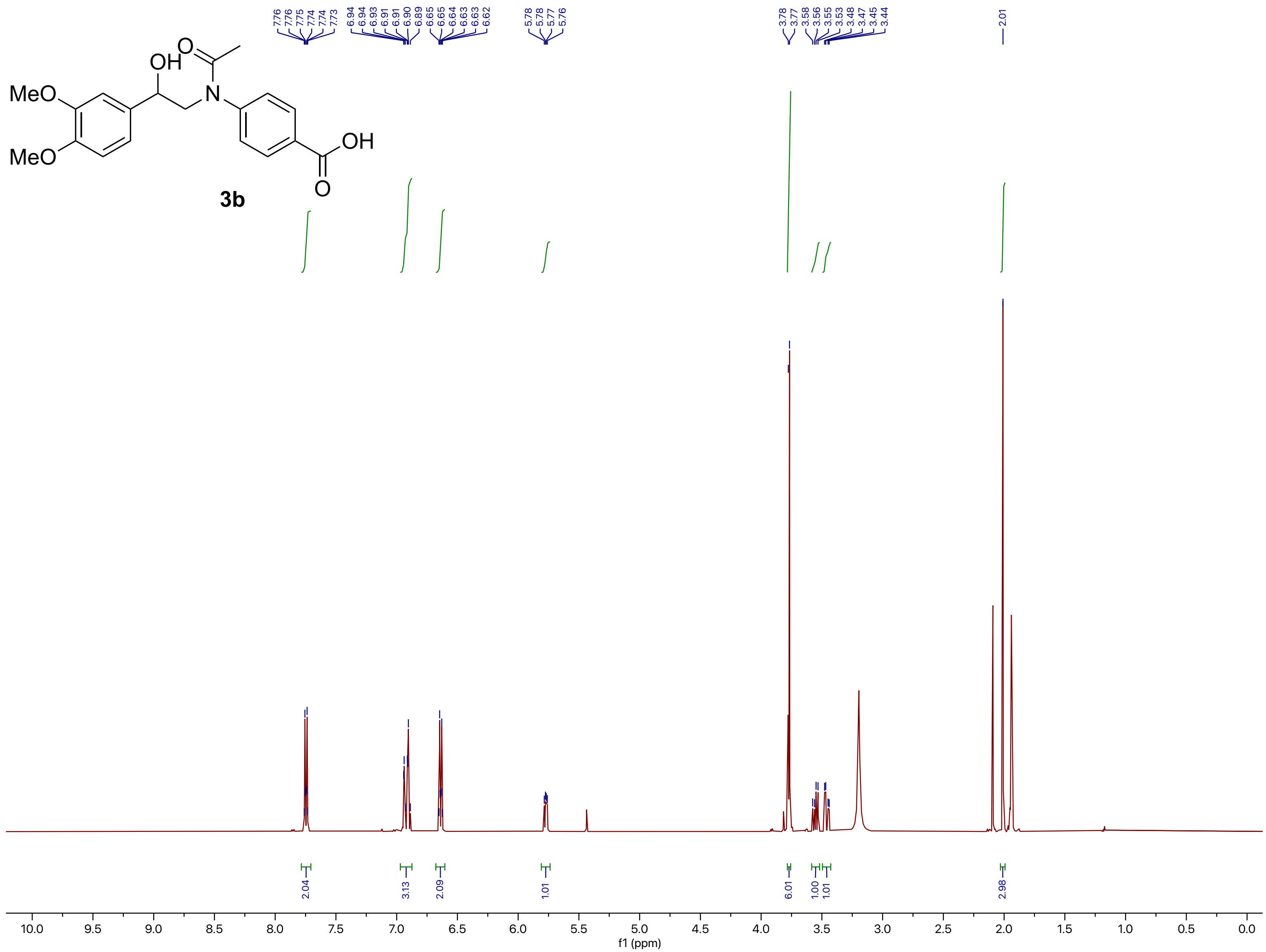

^{1}H-NMR (500 MHz, CD_3CN) of **3b**.

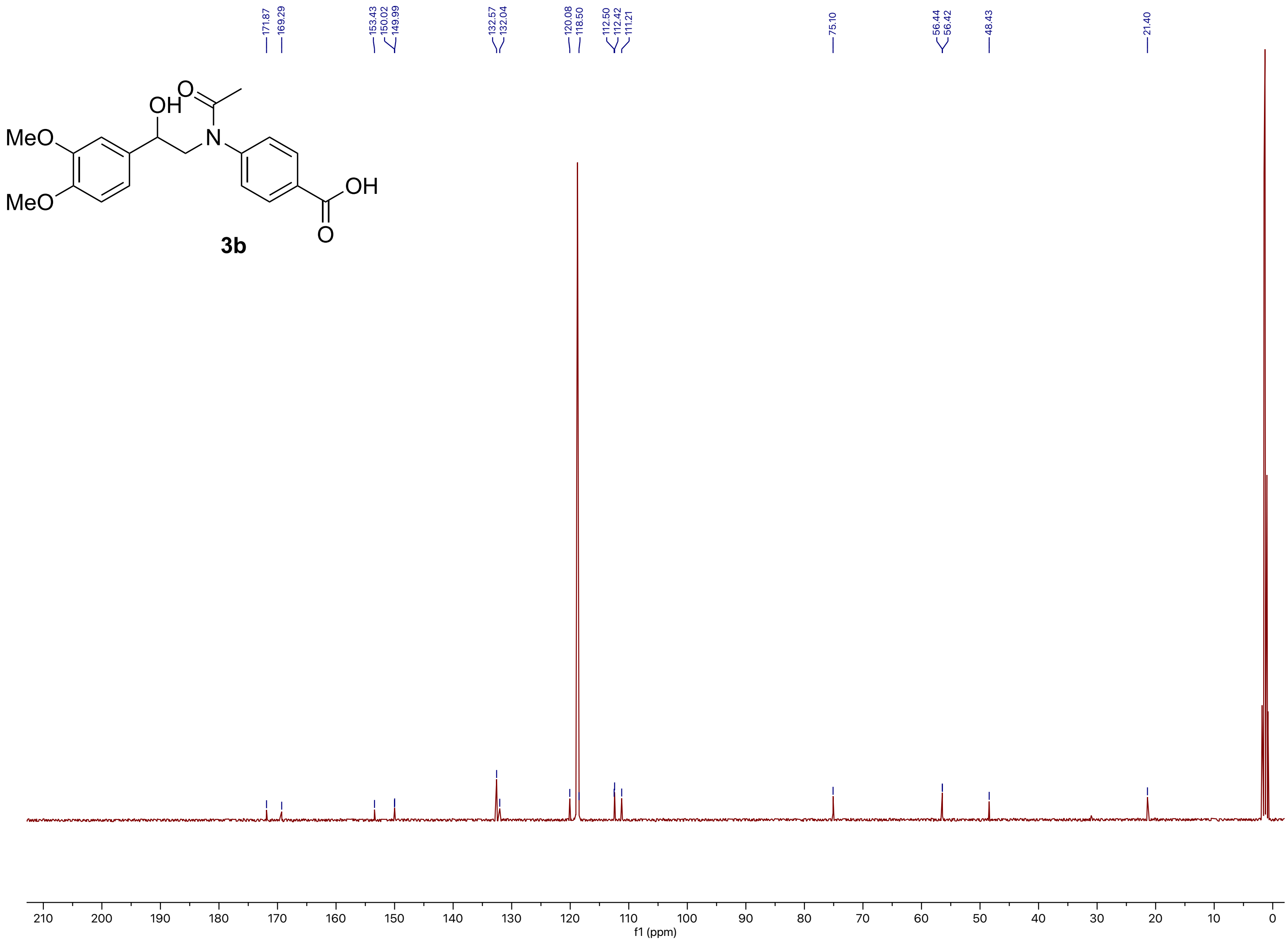

^{13}C-NMR (126 MHz, CD_3CN) of **3b**.

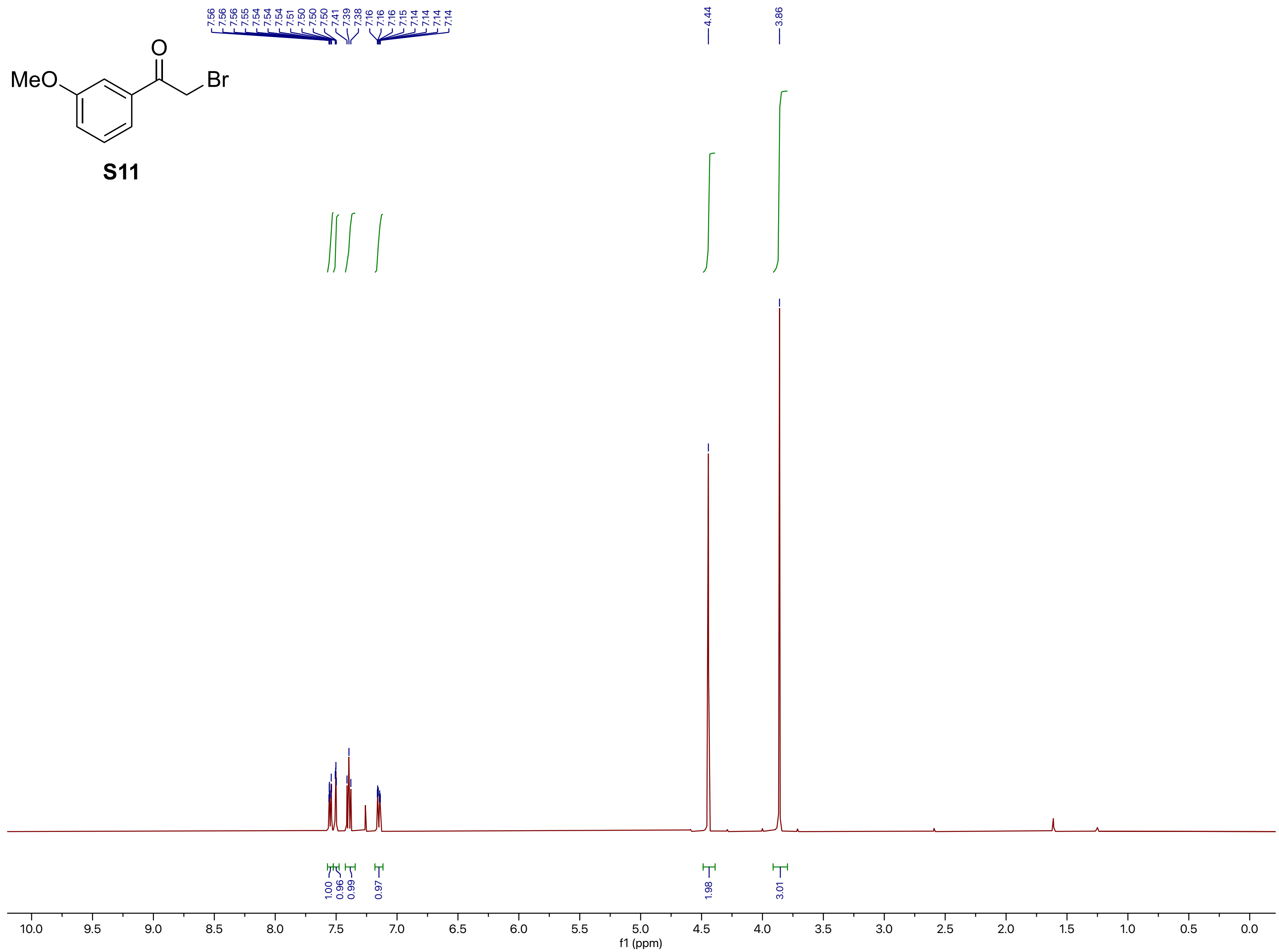

^{1}H-NMR (500 MHz, DMSO) of **S11**.

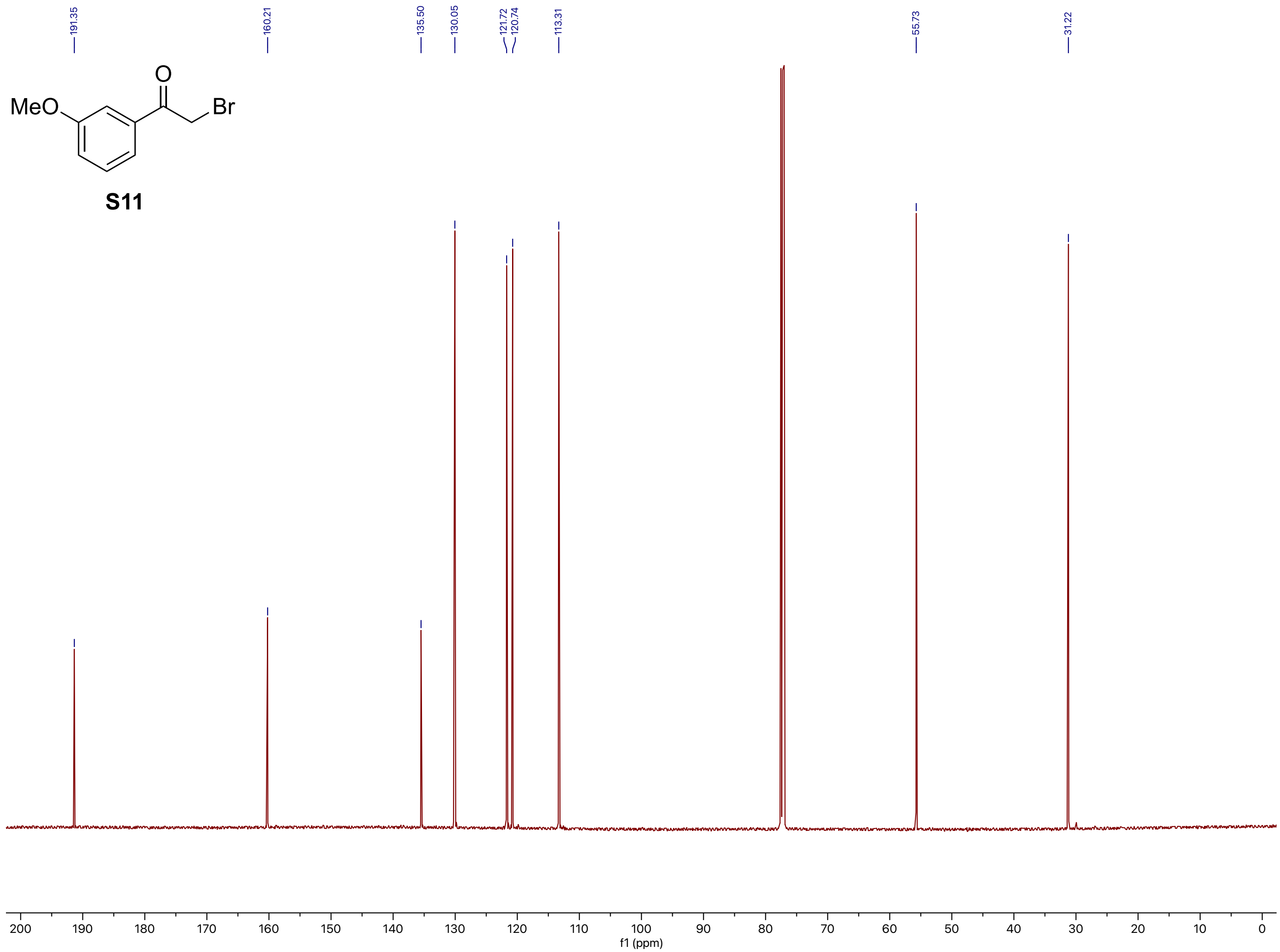

^{13}C-NMR (125 MHz, $CDCl_3$) of **S11**.

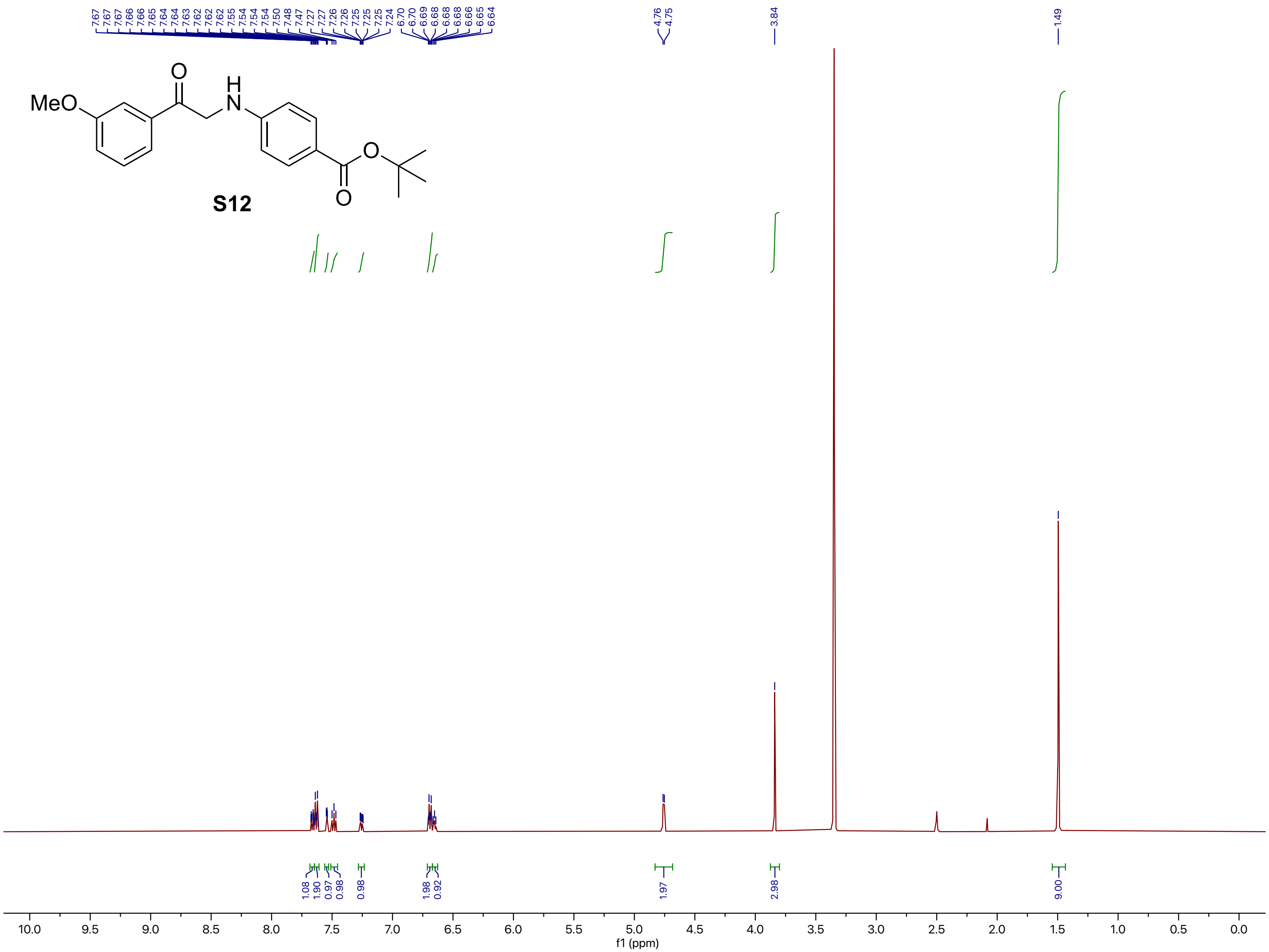

^{1}H-NMR (500 MHz, DMSO) of **S12**.

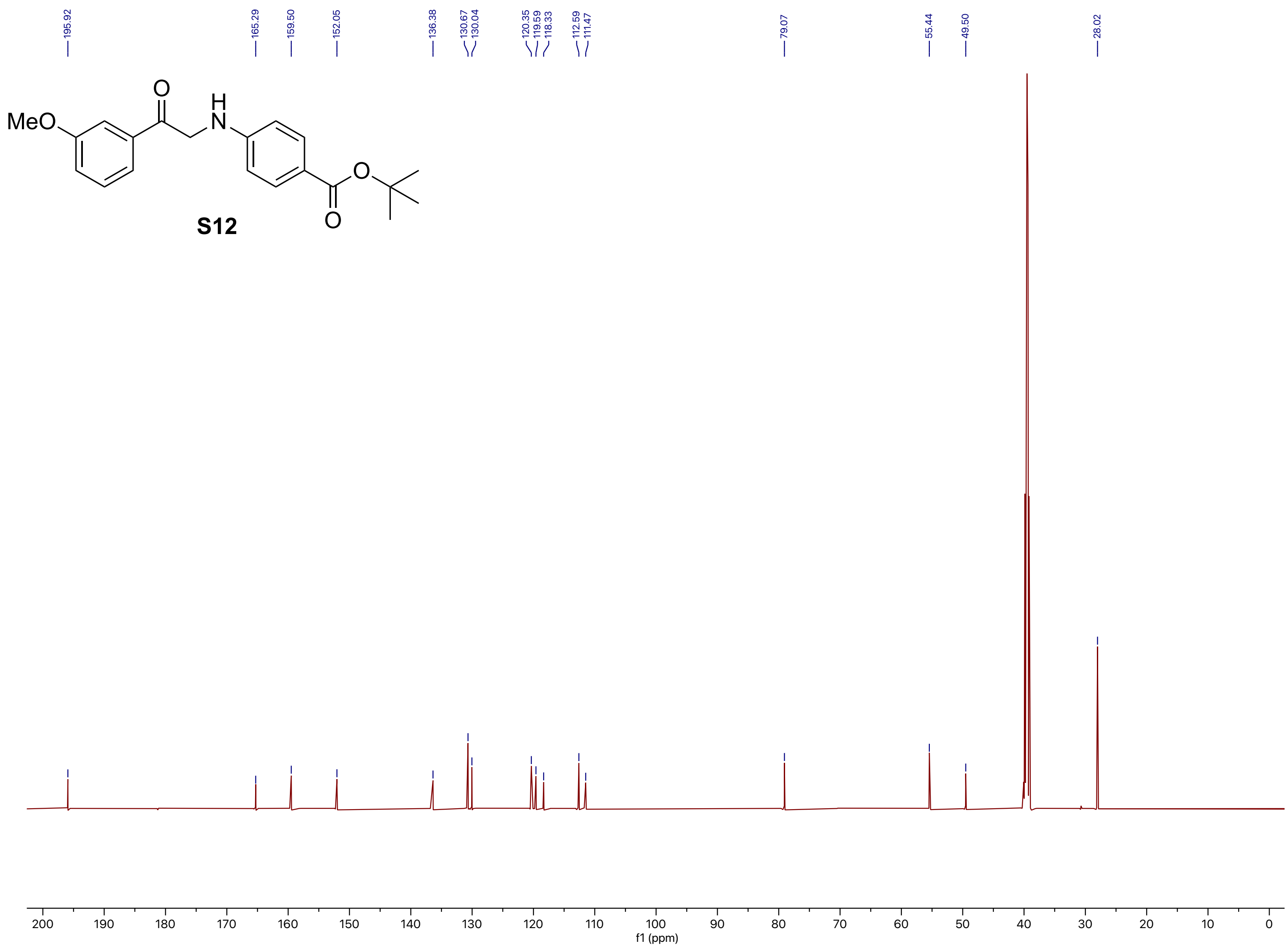

^{13}C-NMR (125 MHz, DMSO) of **S12**.

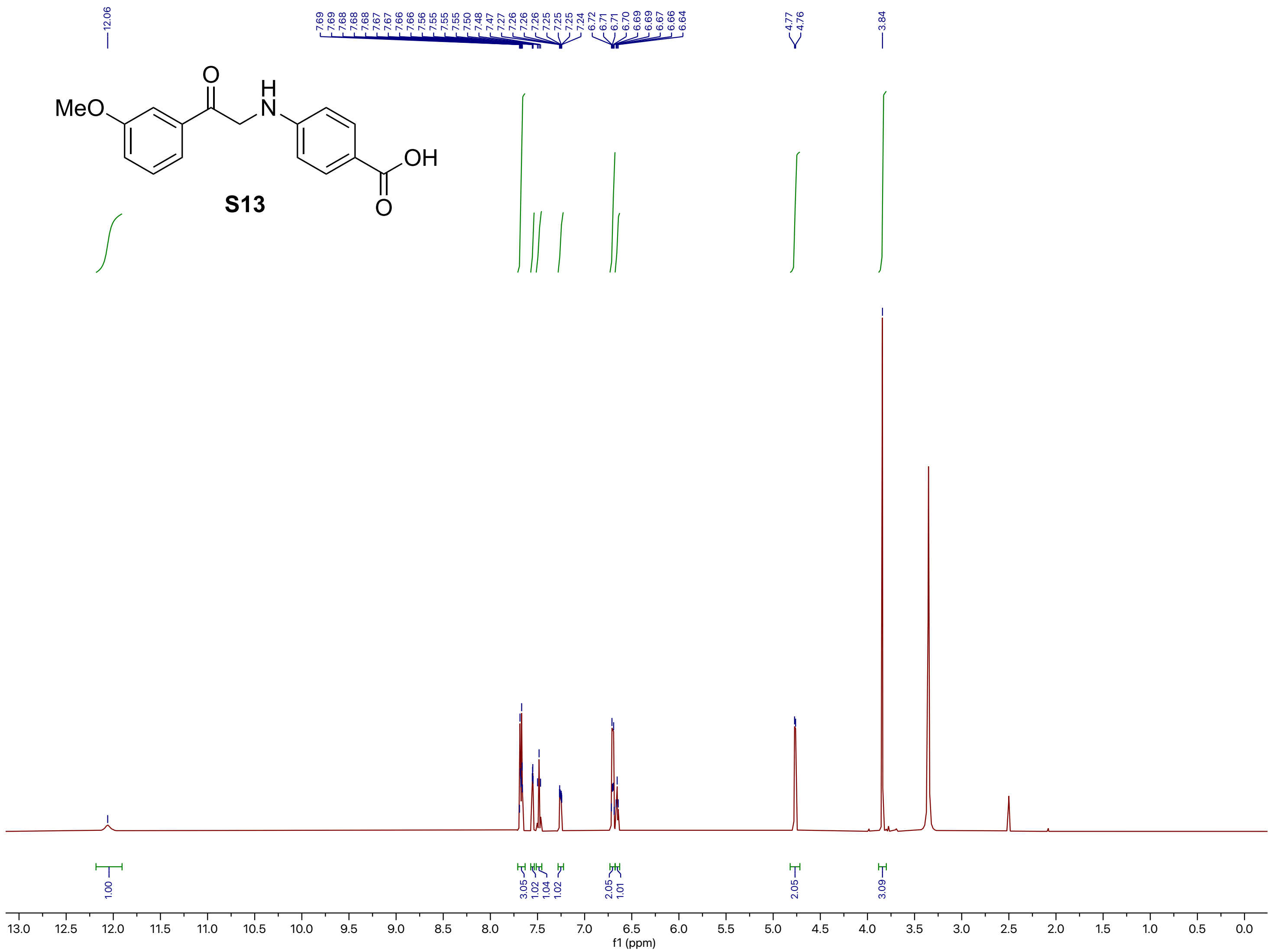

^{1}H-NMR (500 MHz, DMSO) of **S13**.

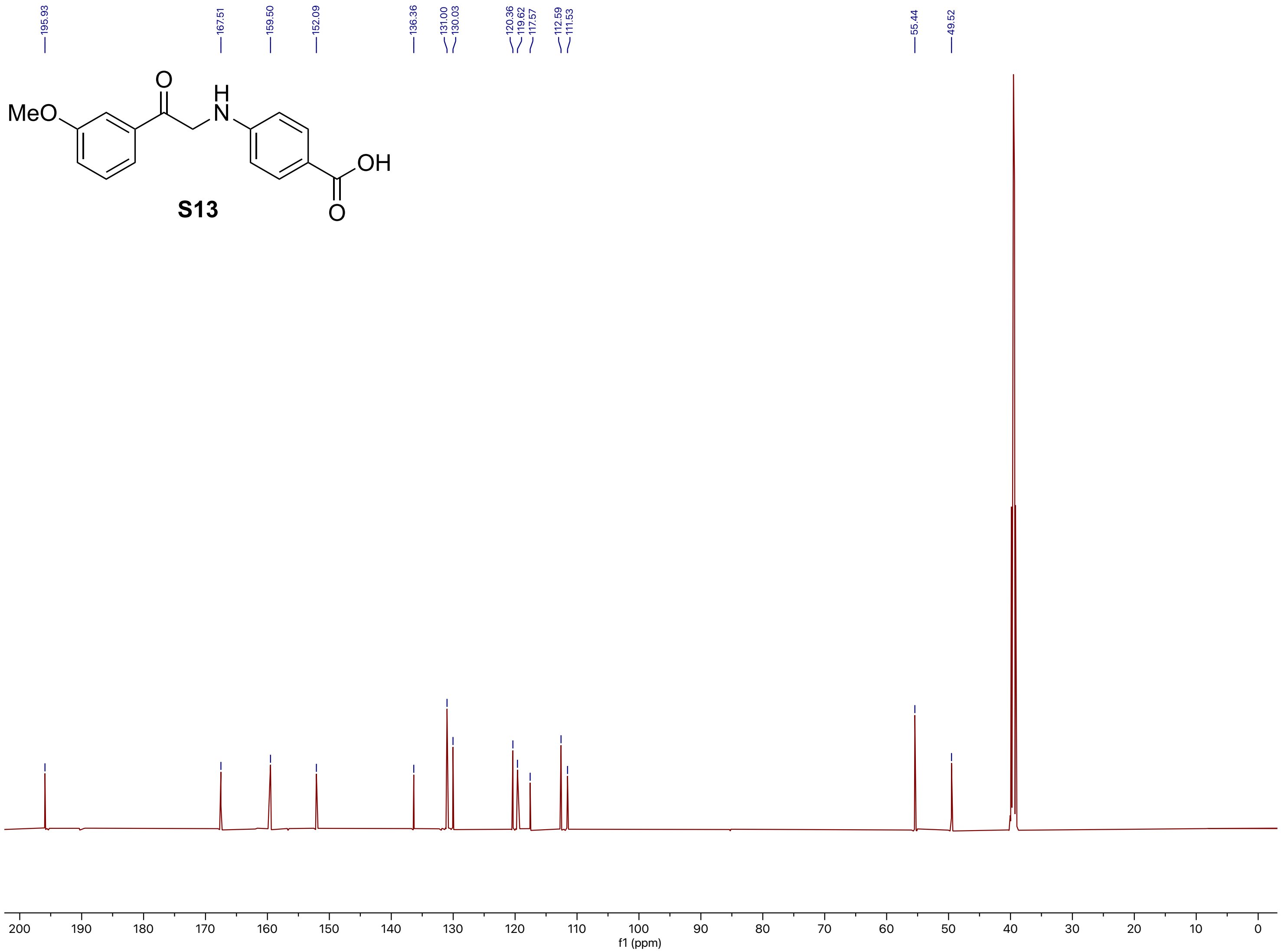

^{13}C-NMR (125 MHz, DMSO) of **S13**.

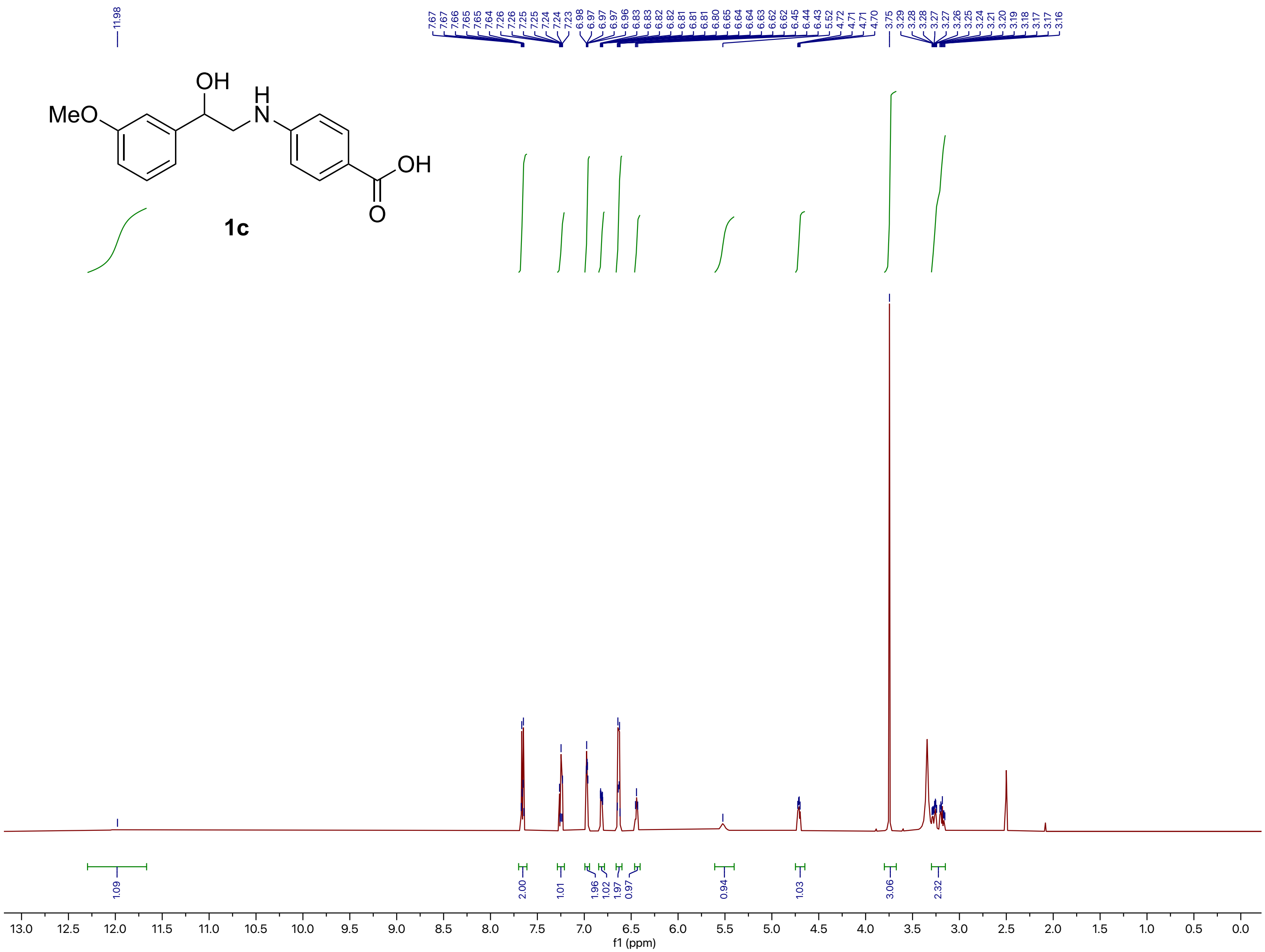

^{1}H-NMR (500 MHz, DMSO) of **1c**.

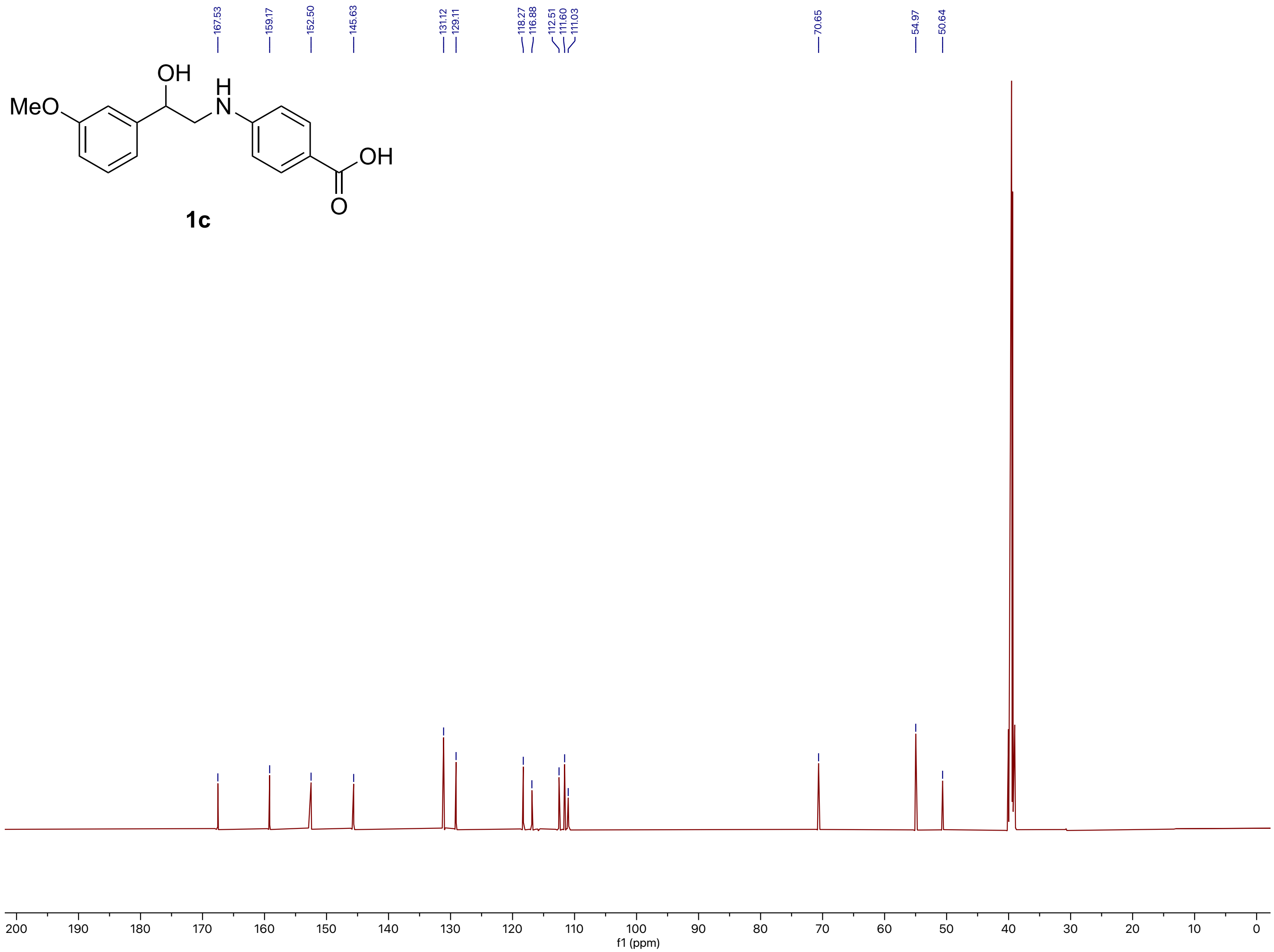

^{13}C-NMR (125 MHz, DMSO) of **1c**.

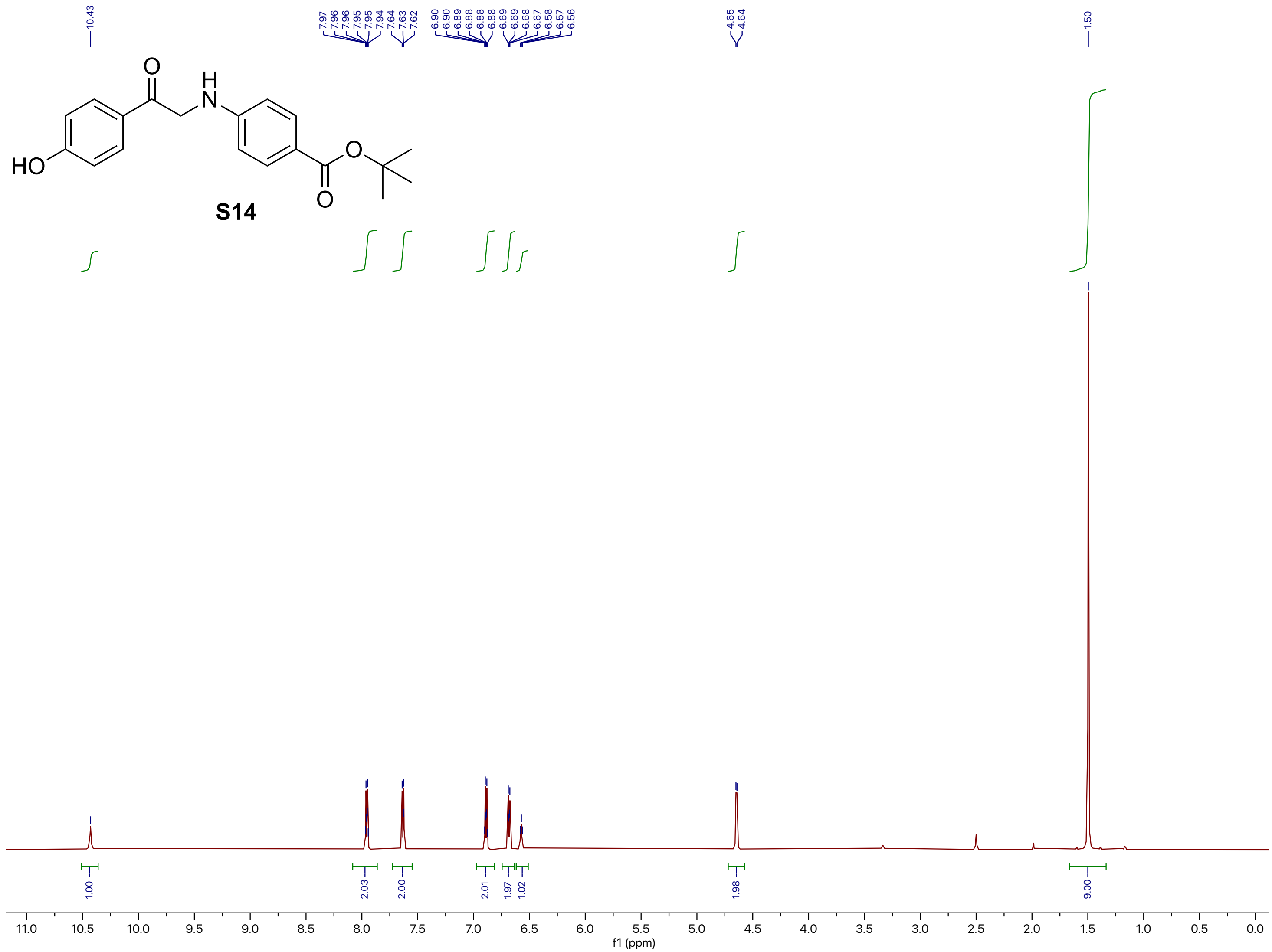

^{1}H-NMR (600 MHz, DMSO) of **S14**.

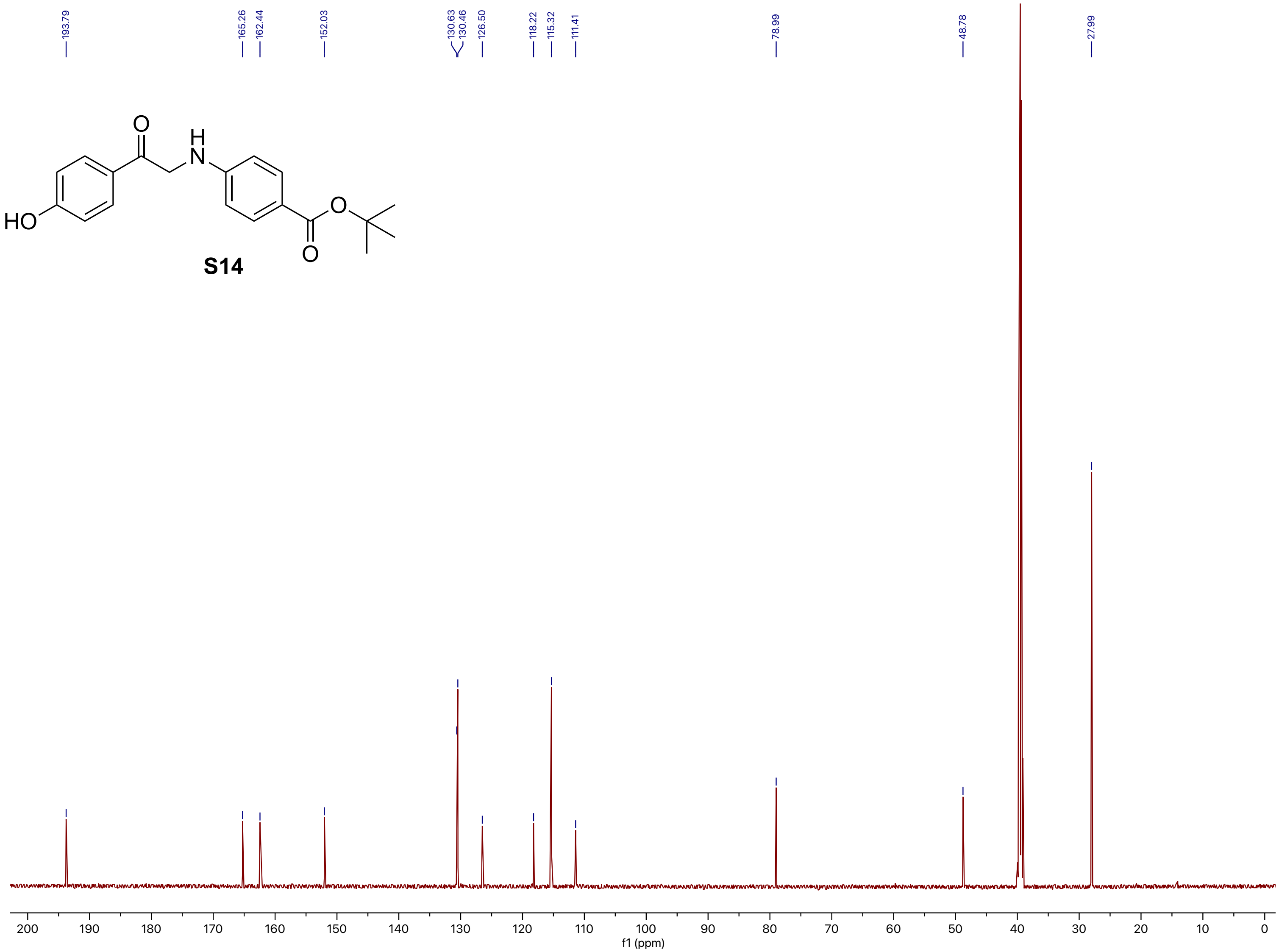

^{13}C-NMR (151 MHz, DMSO) of **S14**.

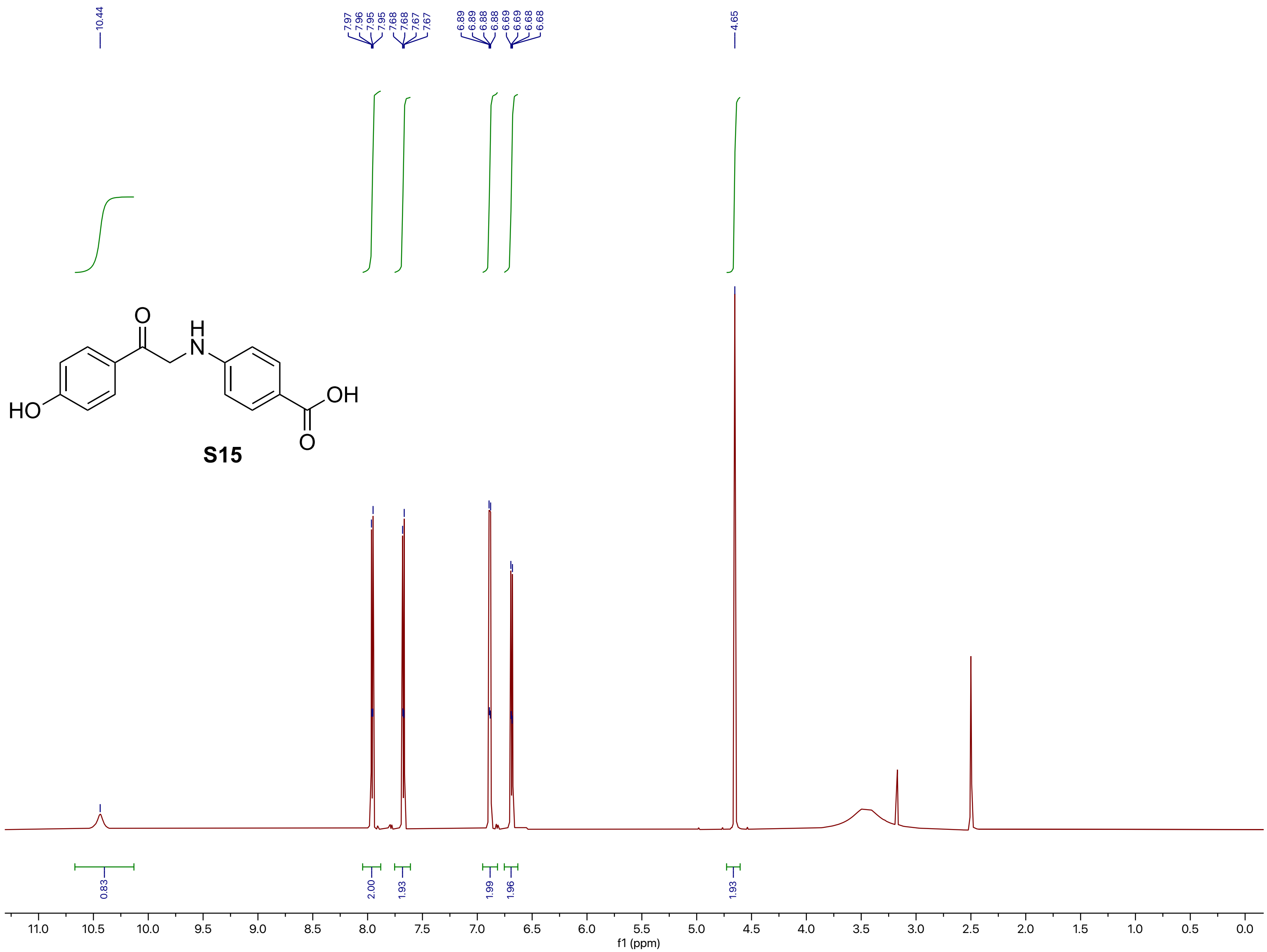

^{1}H-NMR (600 MHz, DMSO) of **S15**.

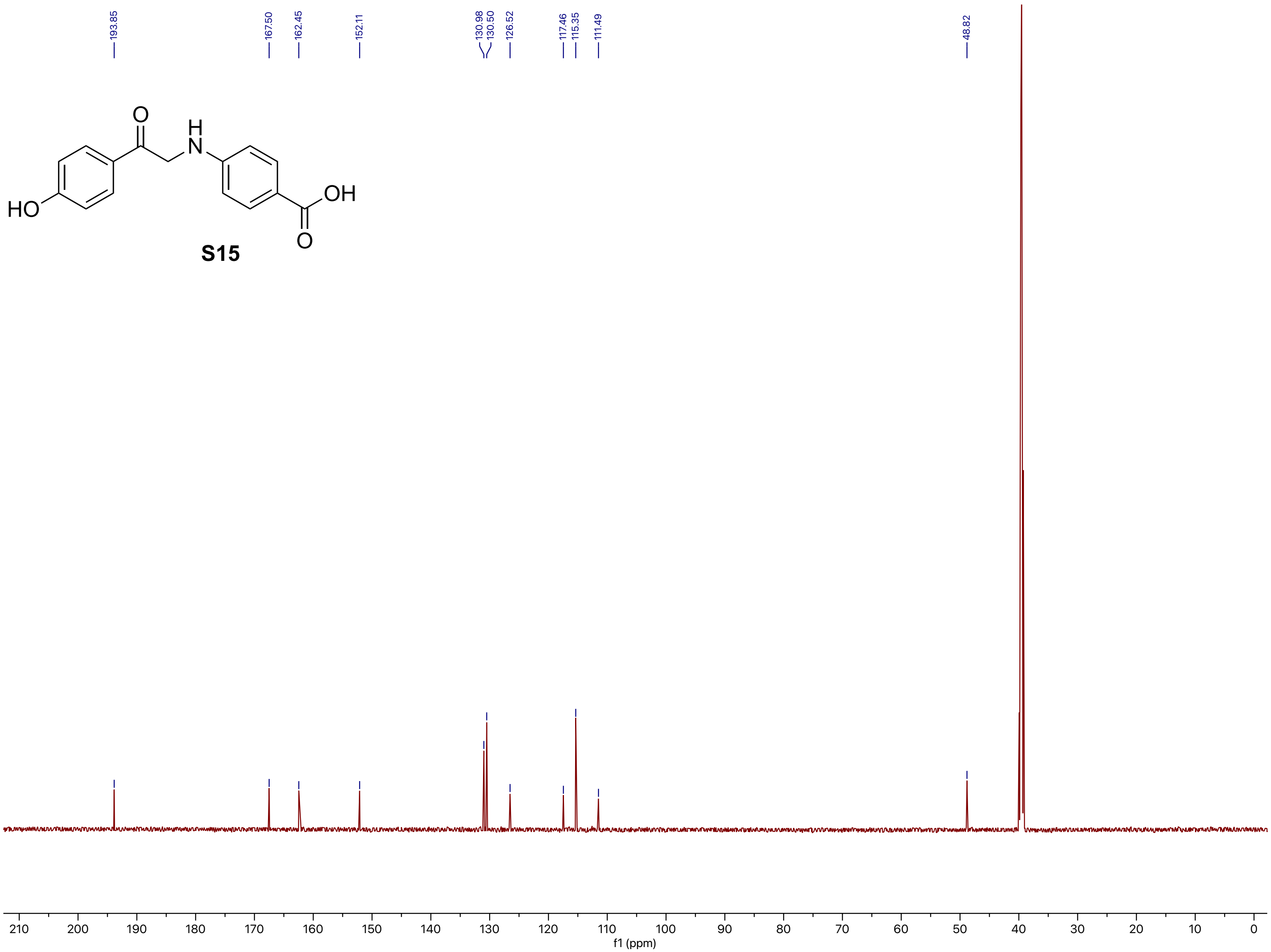

^{13}C-NMR (126 MHz, DMSO) of **S15**.

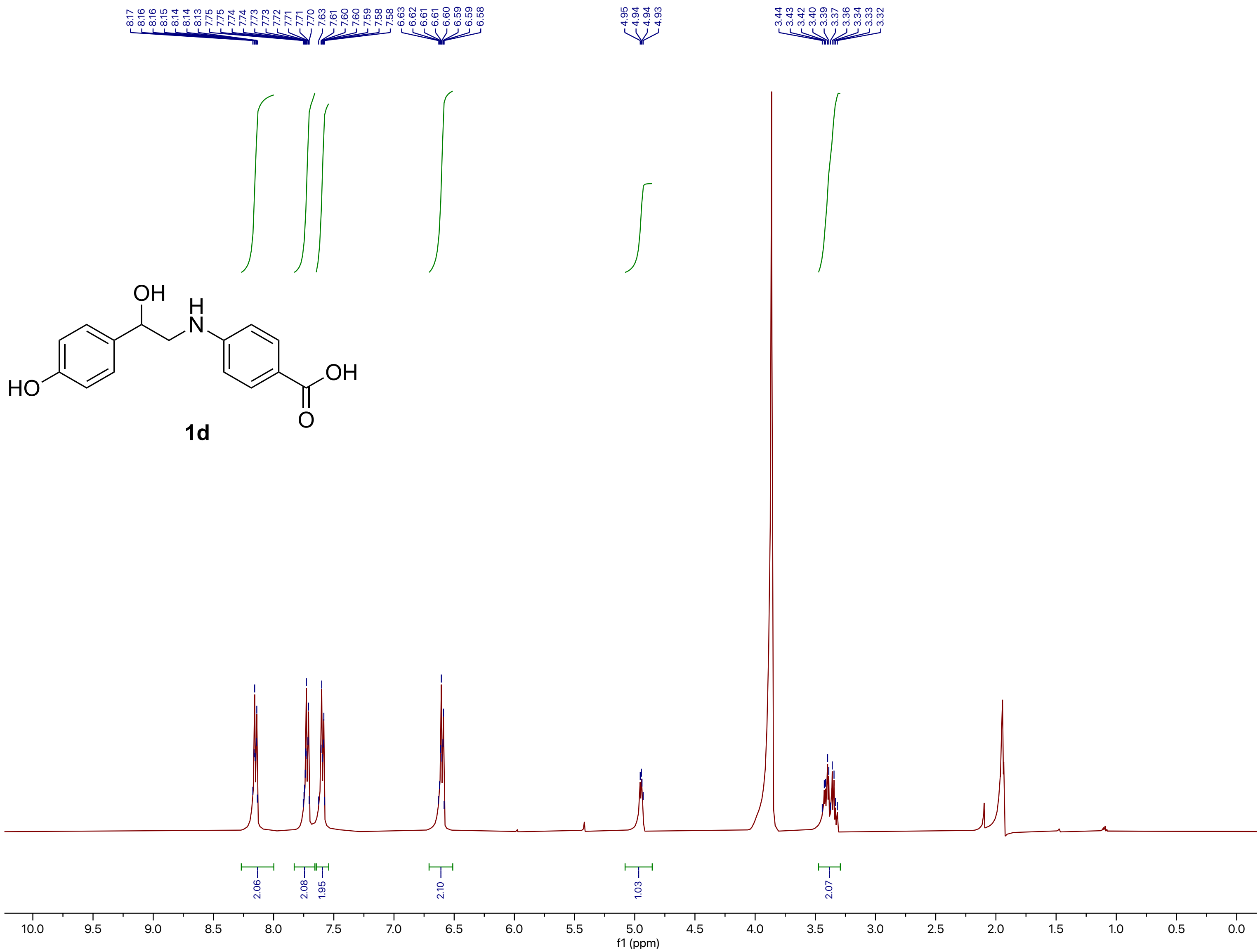

^{1}H-NMR (500 MHz, CD_3CN) of **1d**.

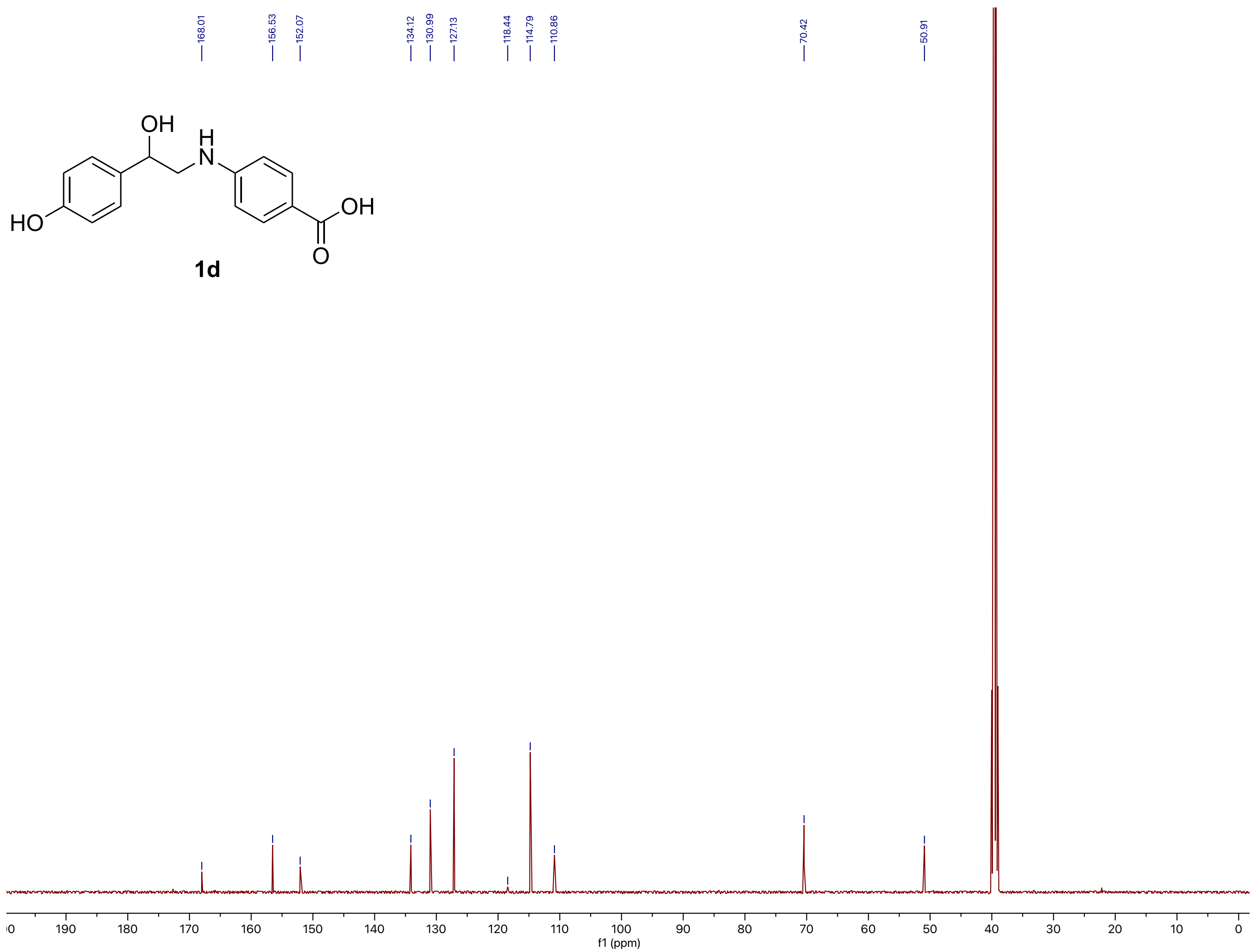

^{13}C-NMR (500 MHz, DMSO) of **1d**.

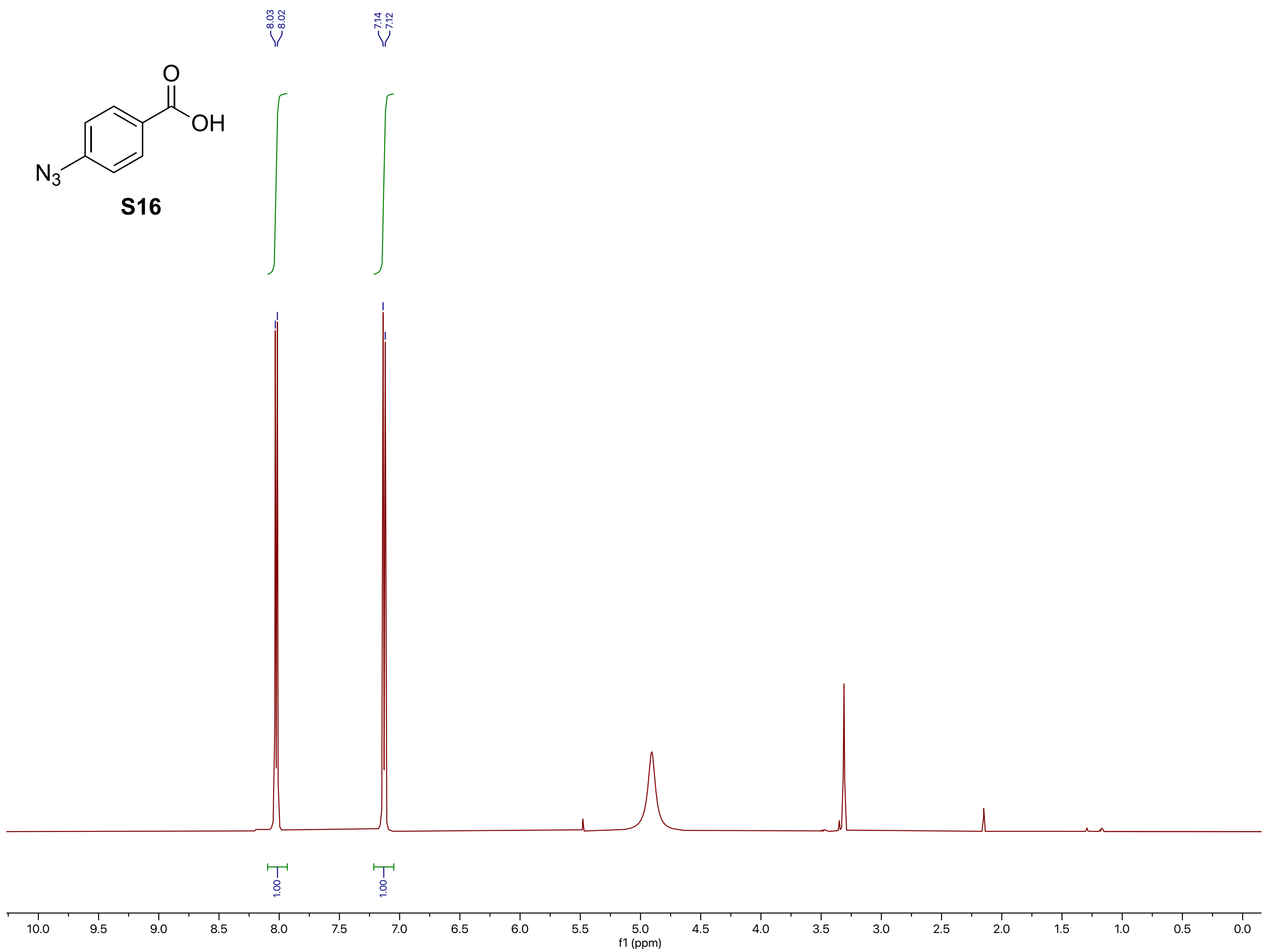

^{1}H-NMR (500 MHz, MeOD) of **S16**.

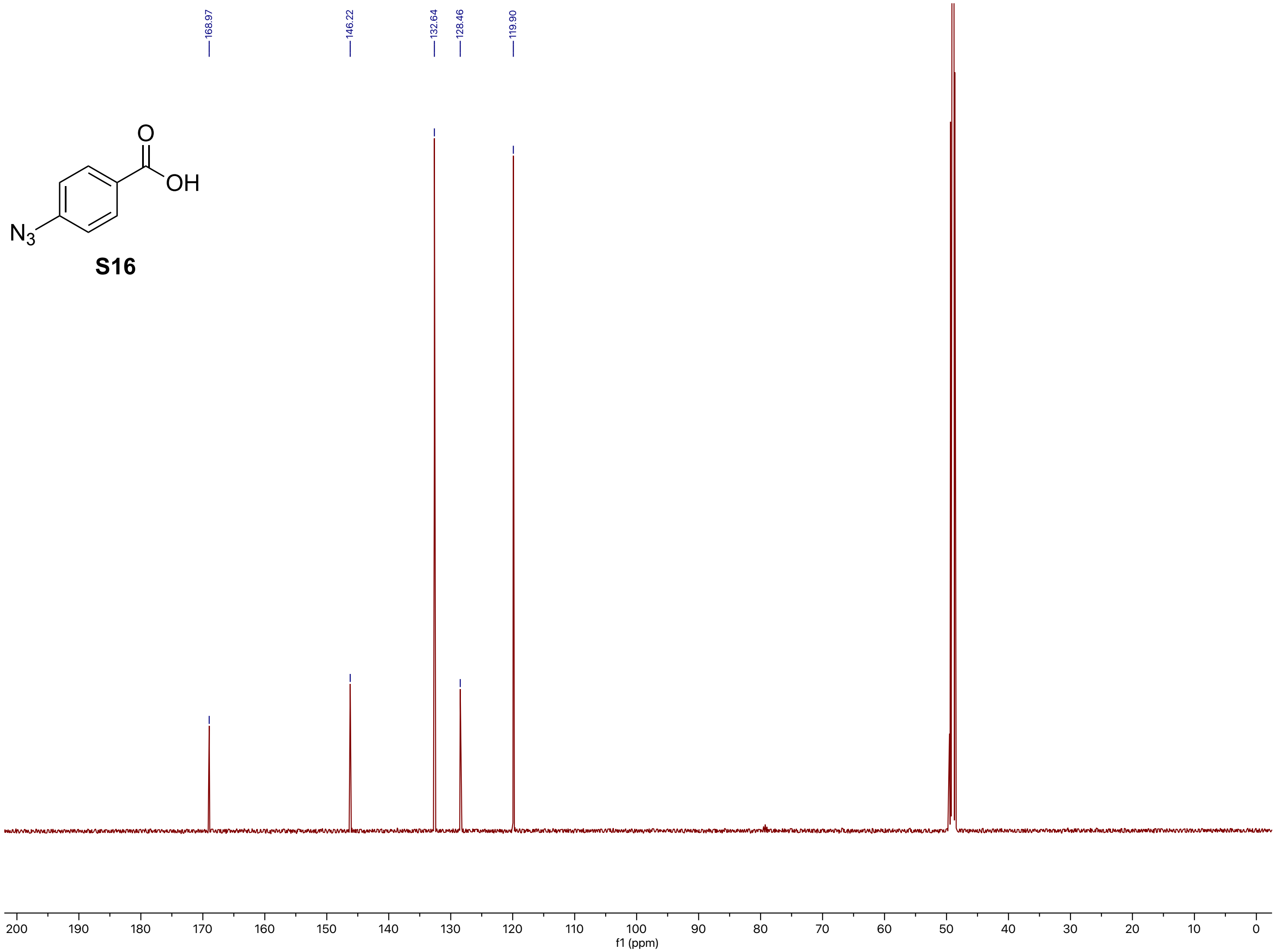

^{1}H-NMR (126 MHz, MeOD) of **S16**.

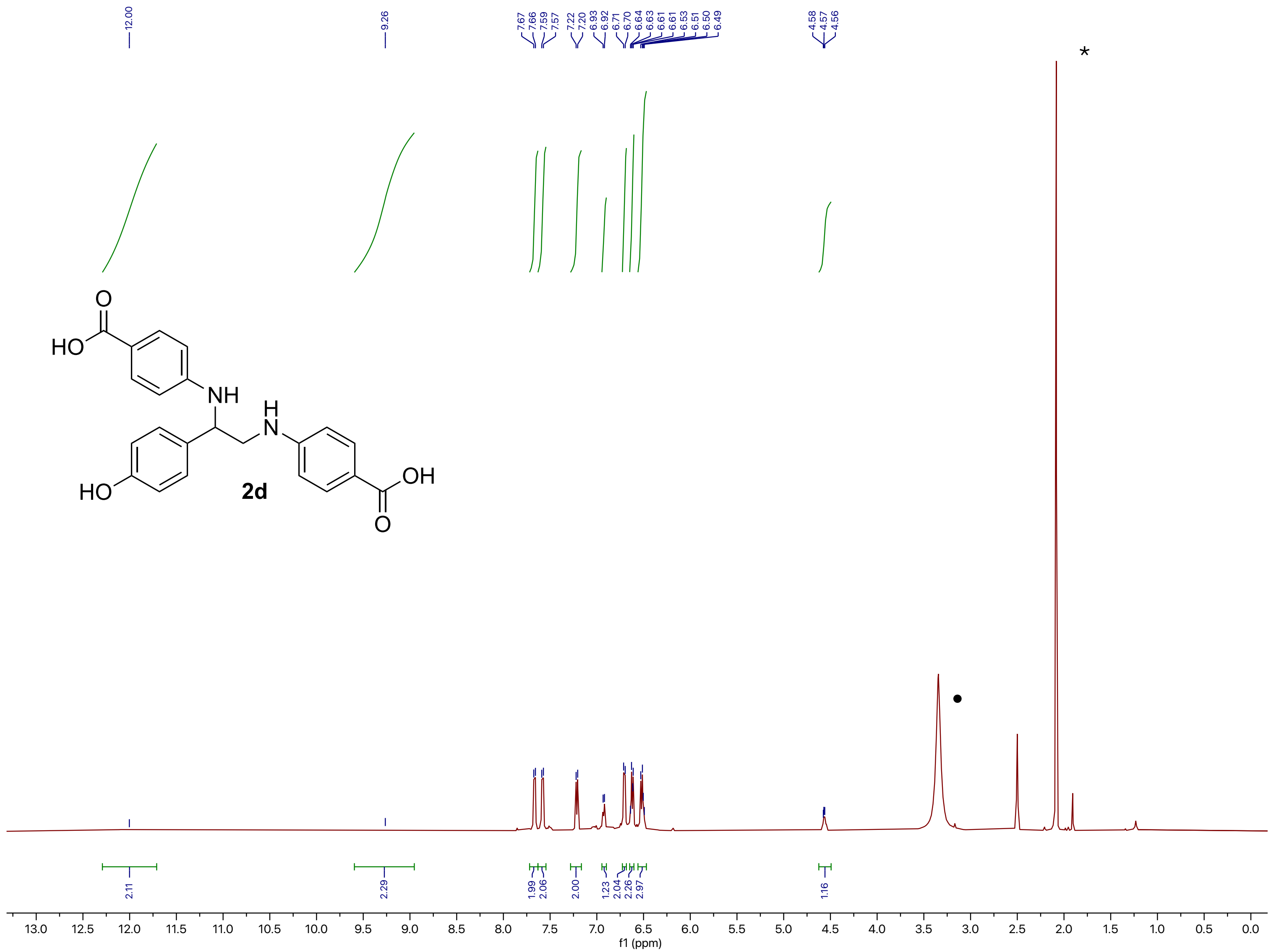

^{1}H-NMR (500 MHz, DMSO) of **2d**. • 2H (CH_2) isochronous with the protons of water (3.2 ppm-3.4 ppm) (see Supplementary Fig. 47-48) *Impurity due to Acetone.

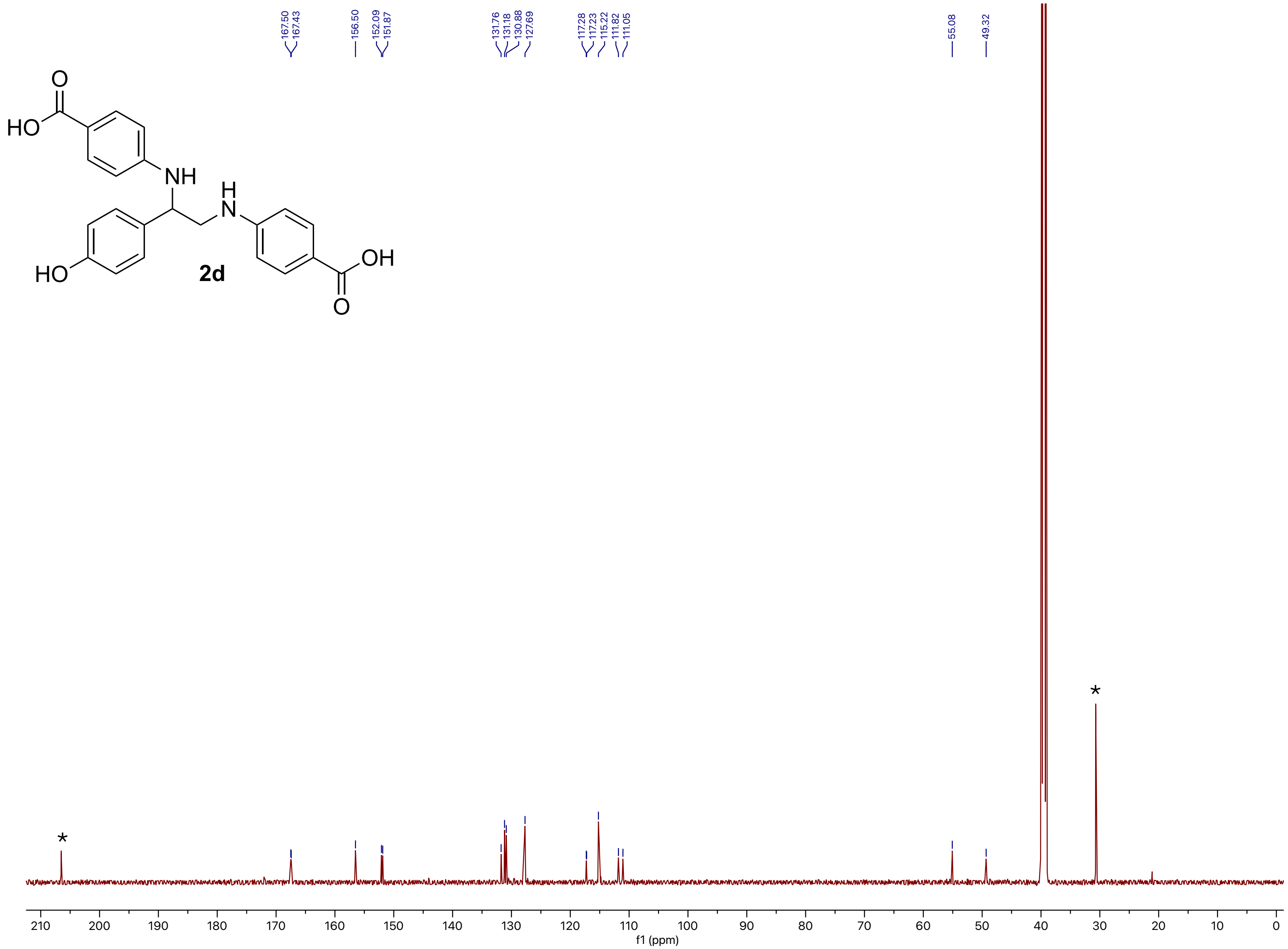

^{13}C-NMR (126 MHz, DMSO) of **2d**. *Impurity due to Acetone.

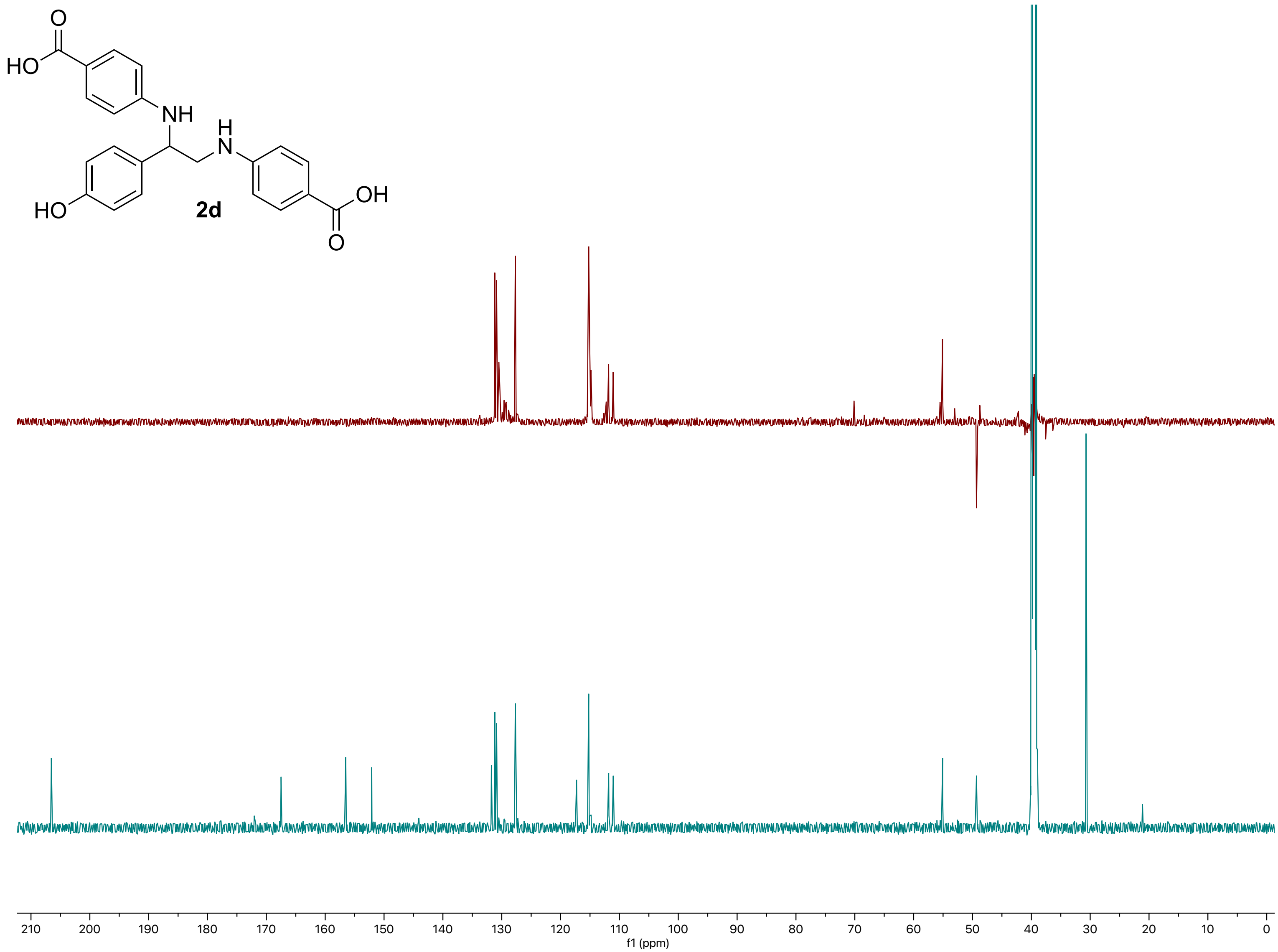

Comparison of DEPT-135 (151 MHz, DMSO) (top) of **2d** with ^{13}C-NMR (126 MHz, DMSO) of **2d** (bottom). Carbon with resonance 49.32 ppm corresponds to a methylene (CH_2). Impurities due to degradation of sample at room temperature.

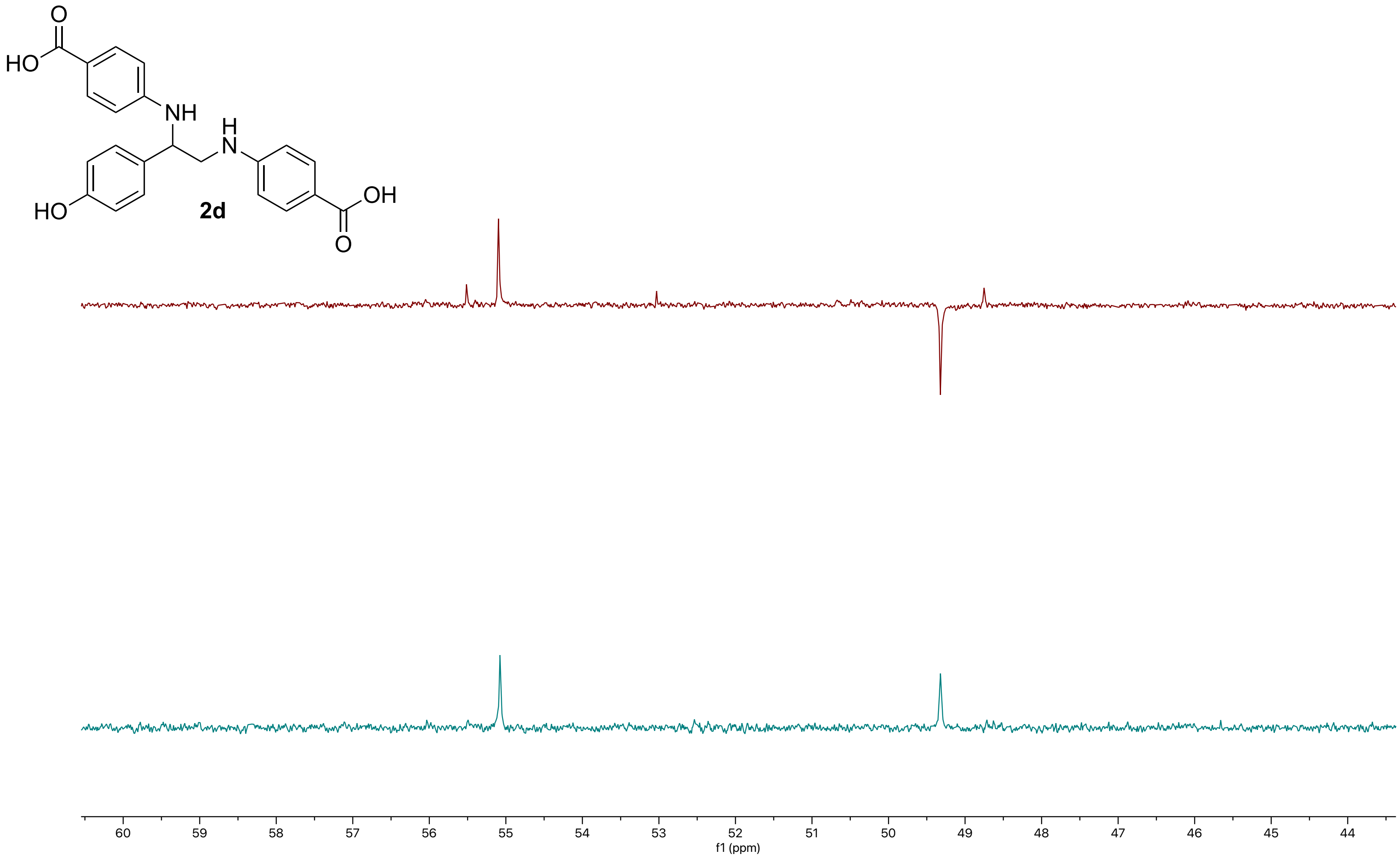

Zoom-in (44-60 ppm) comparison of DEPT-135 (151 MHz, DMSO) (top) of **2d** with ^{13}C-NMR (126 MHz, DMSO) of **2d** (bottom). Carbon with resonance 49.32 ppm corresponds to a methylene (CH_2). Impurities due to degradation of sample at room temperature.

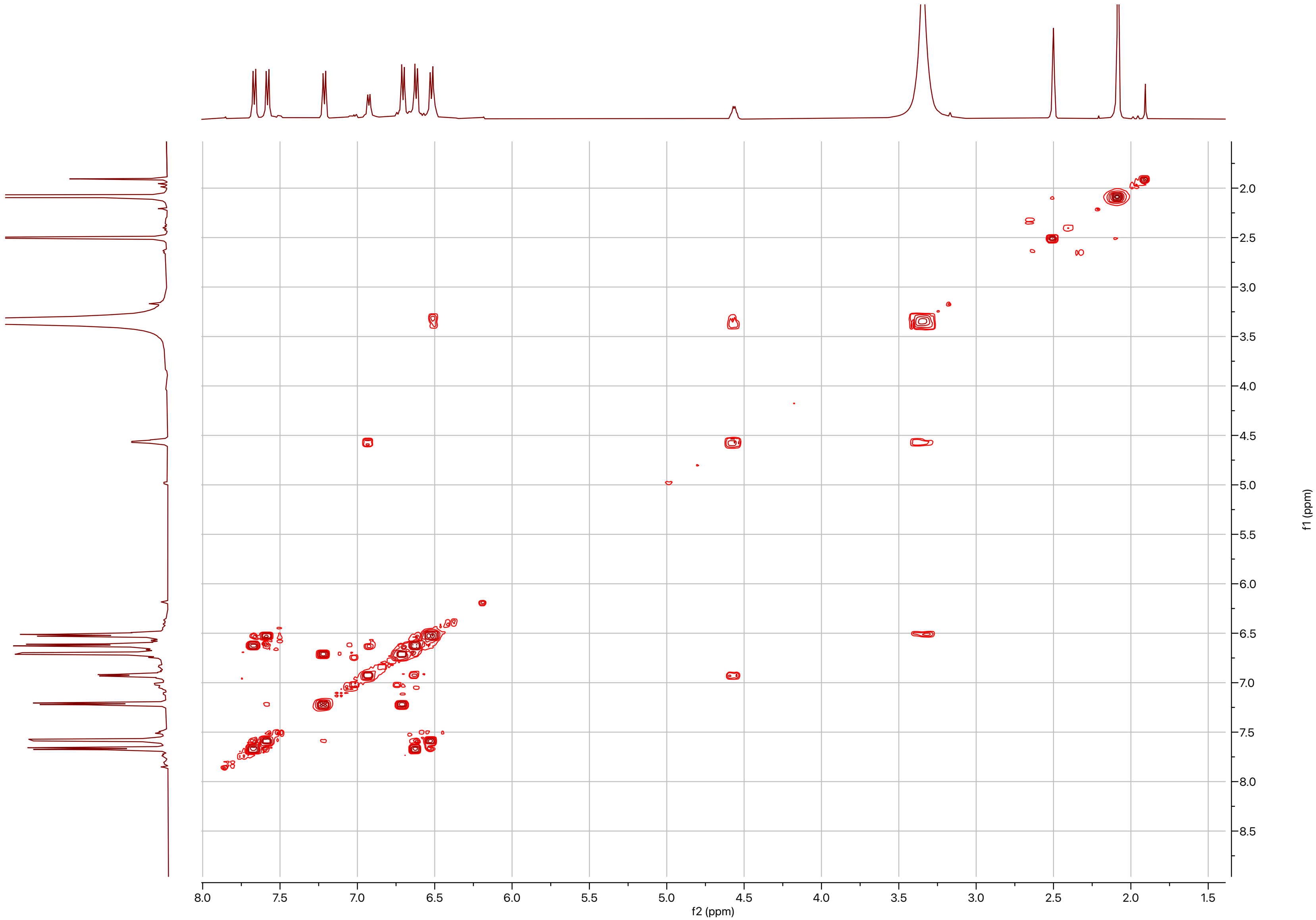

^{1}H-^{1}H COSY (500 MHz, $CDCl_3$) of **2d**.

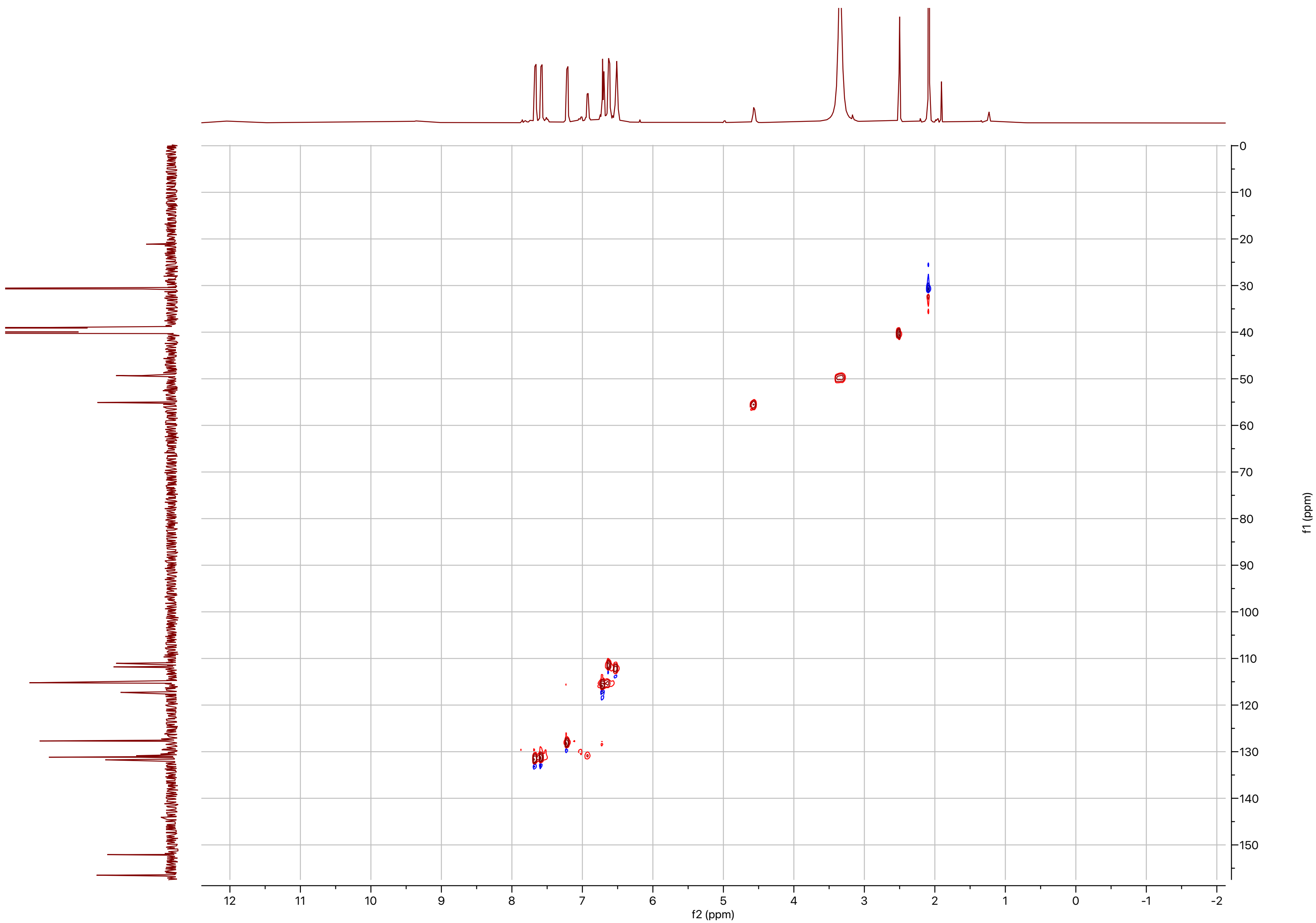

^{1}H-^{13}C HSQC (500 MHz, 126 MHz, $CDCl_3$) of **2d**. Carbon at 49.32 ppm (CH_2) is correlated with protons isochronous with the protons corresponding to water (3.2-3.4 ppm).

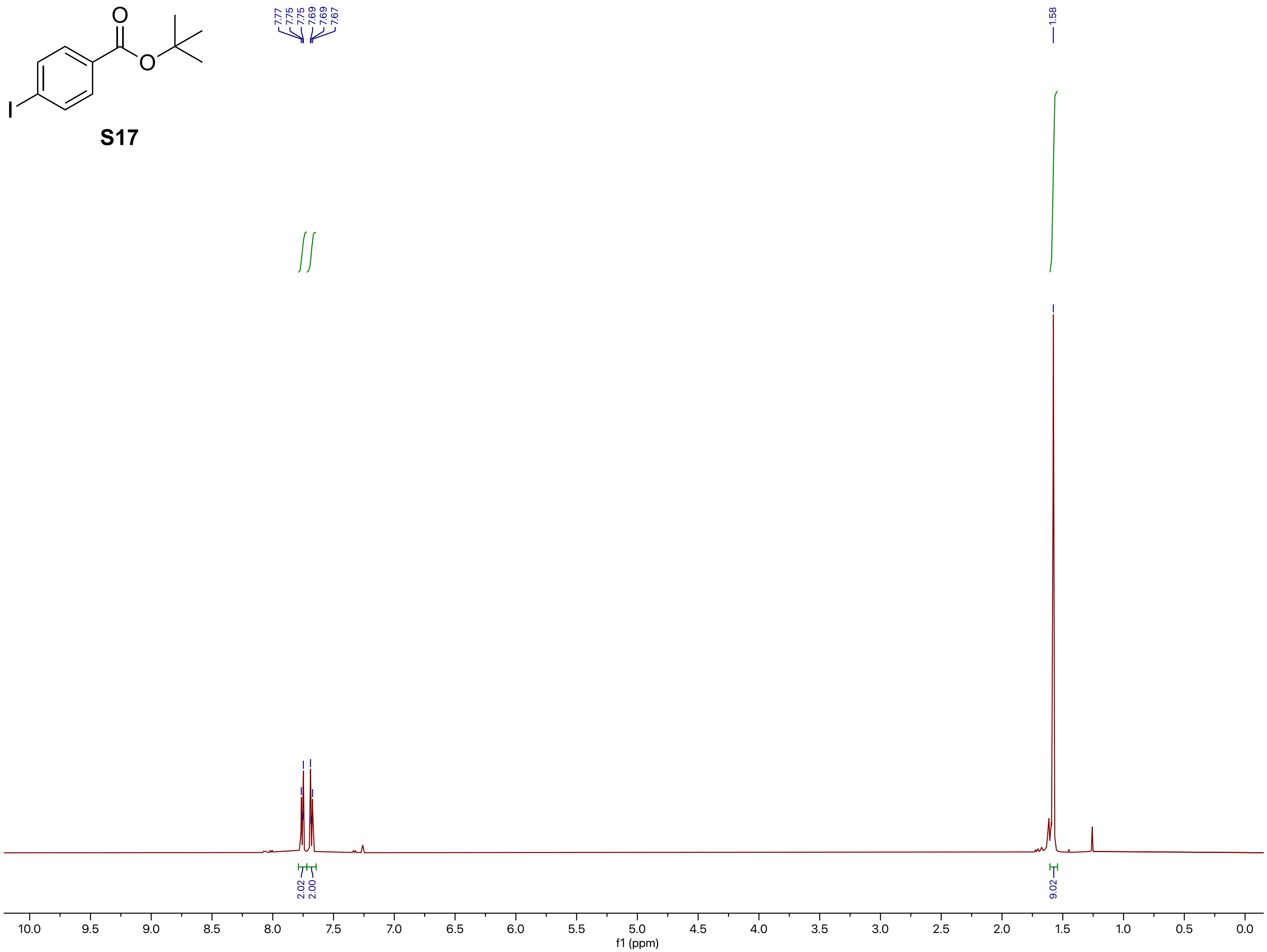

^{1}H-NMR (500 MHz, $CDCl_3$) of **S17**.

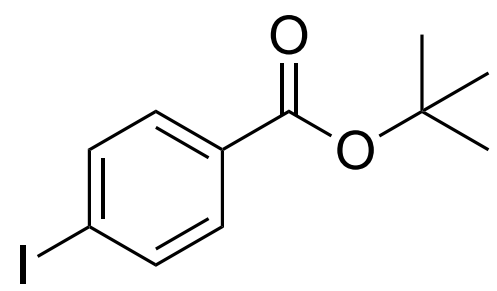

S17

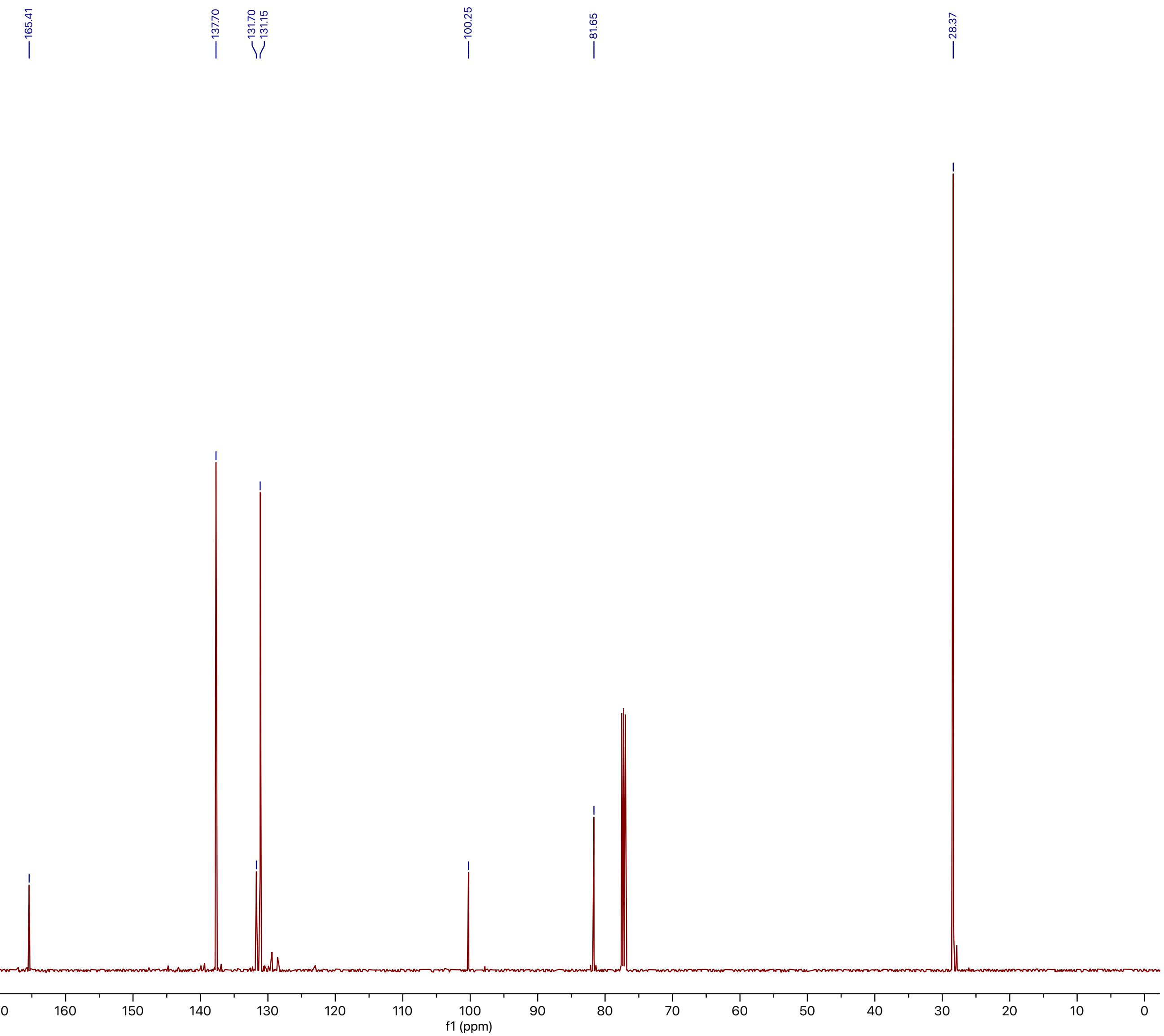

^{13}C-NMR (126 MHz, $CDCl_3$) of **S17**.

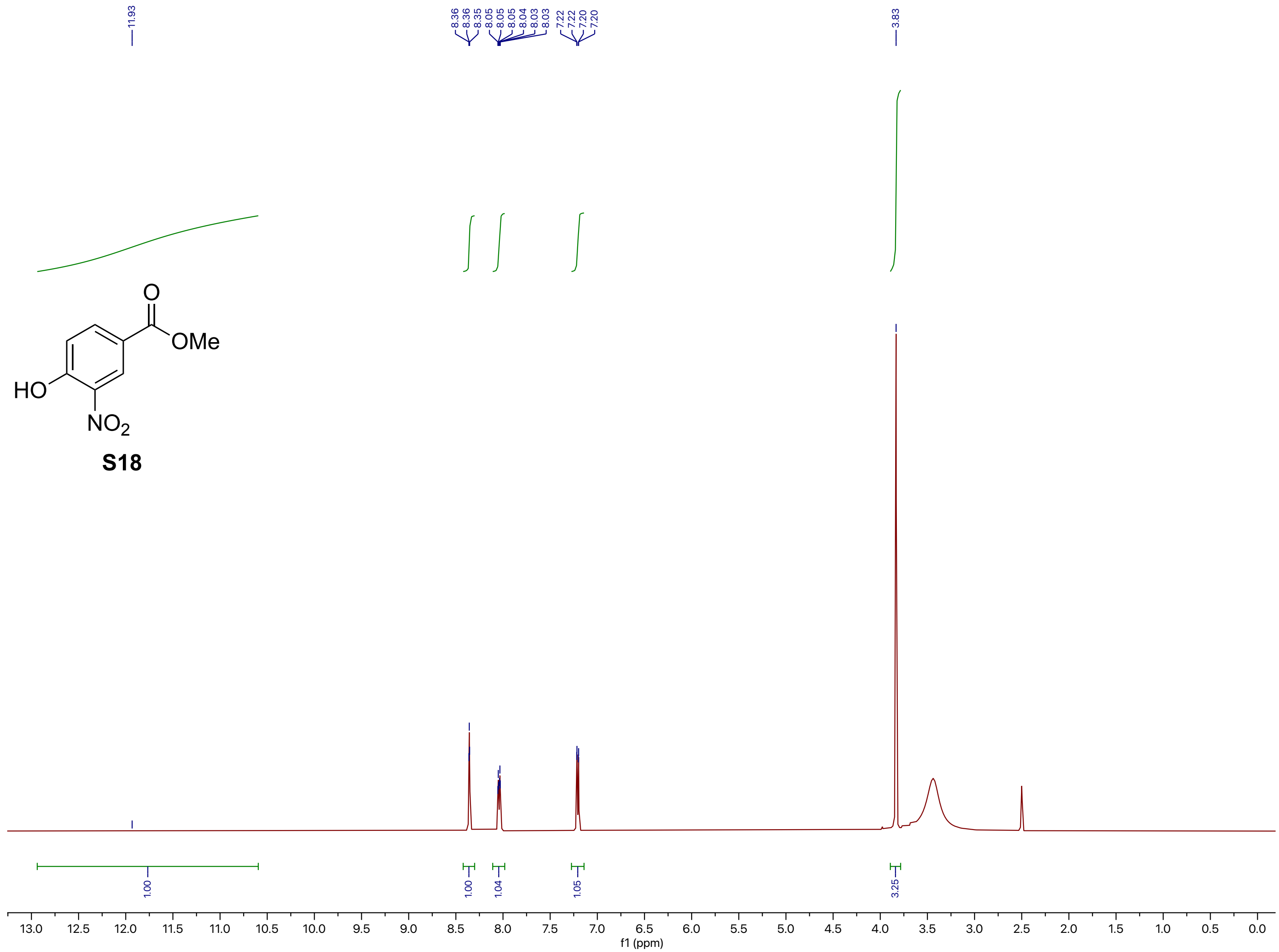

^{1}H-NMR (500 MHz, DMSO) of **S18**.

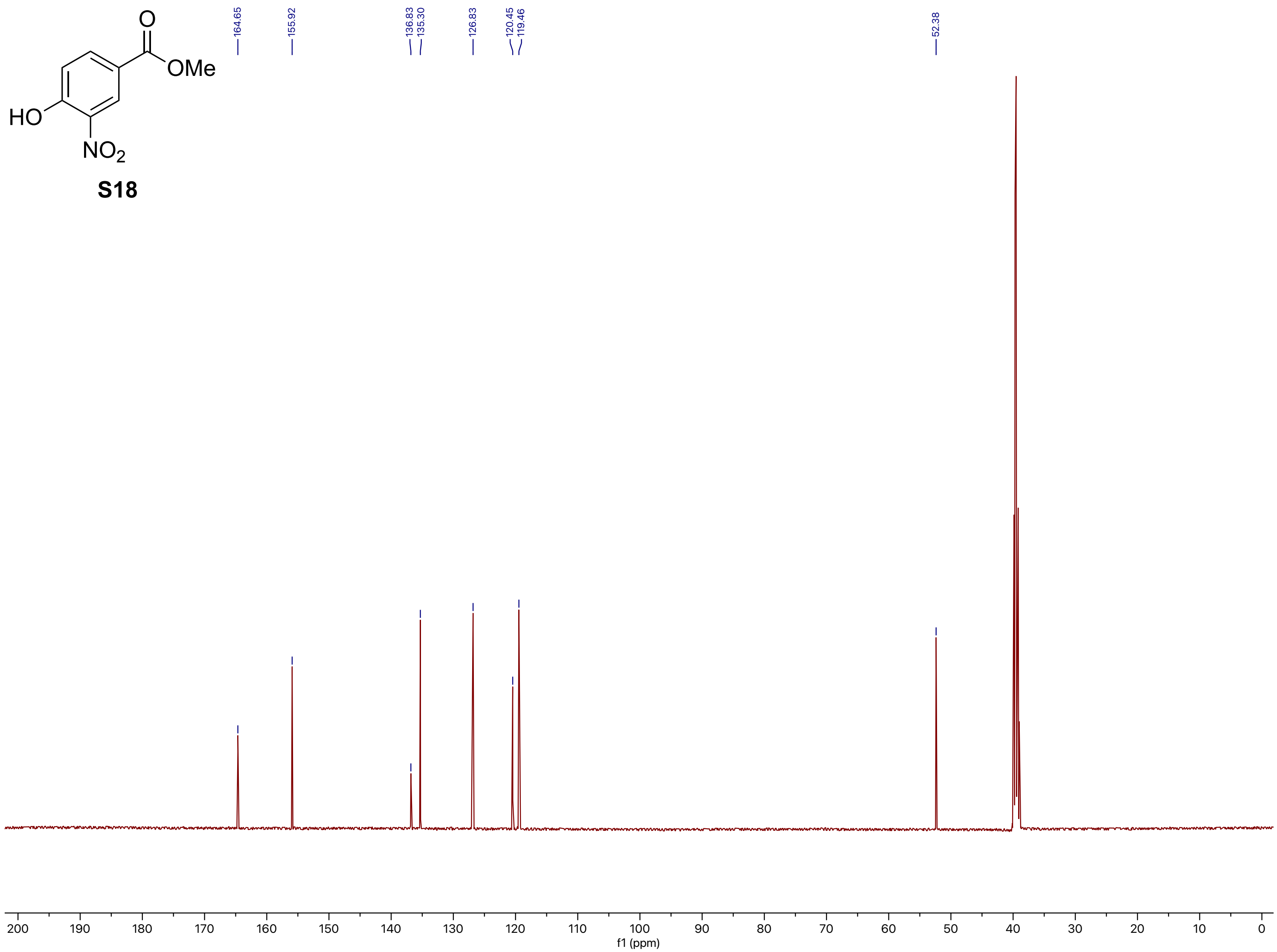

^{13}C-NMR (126 MHz, DMSO) of **S18**.

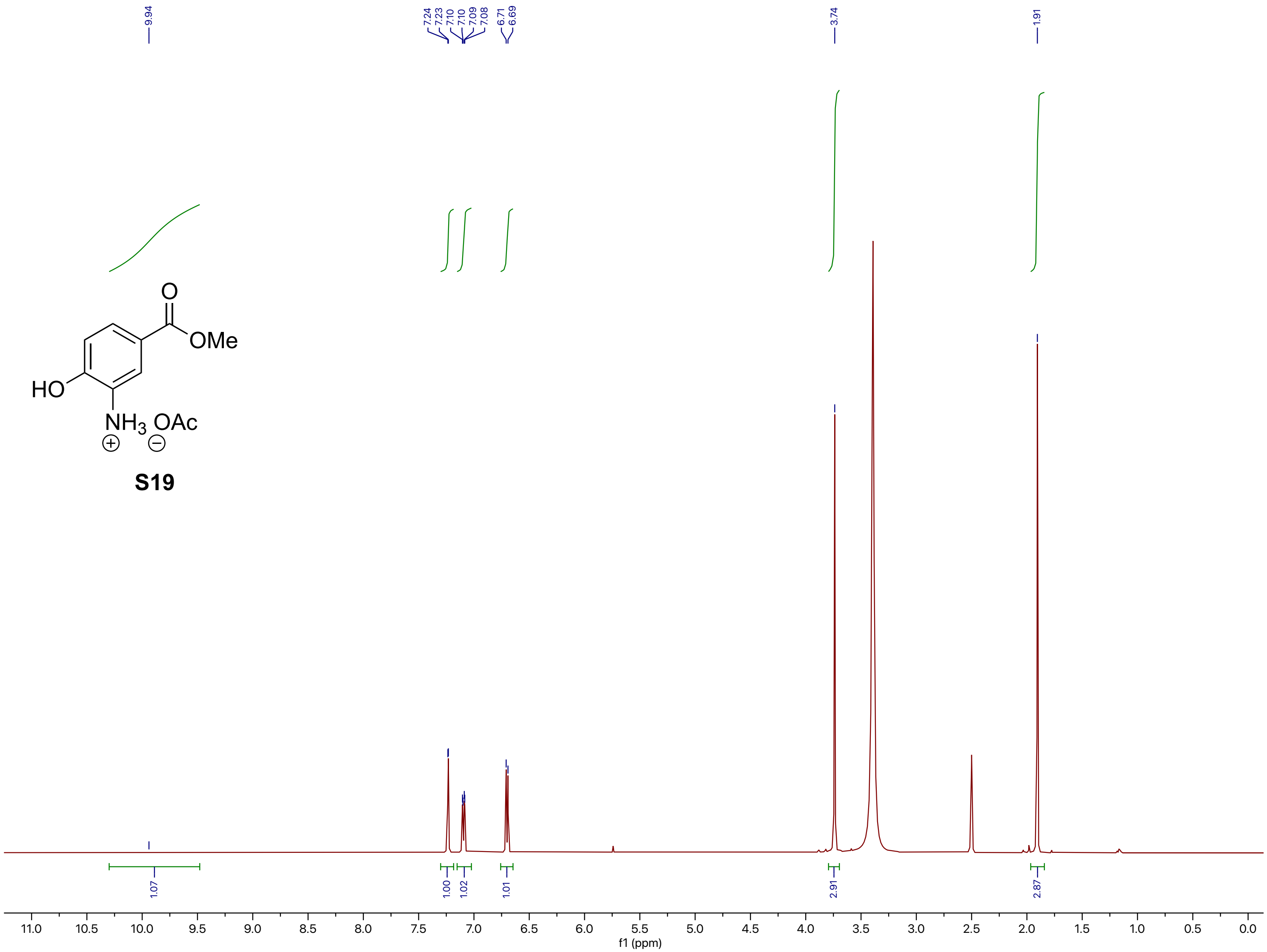

^{1}H-NMR (500 MHz, DMSO) of **S19**.

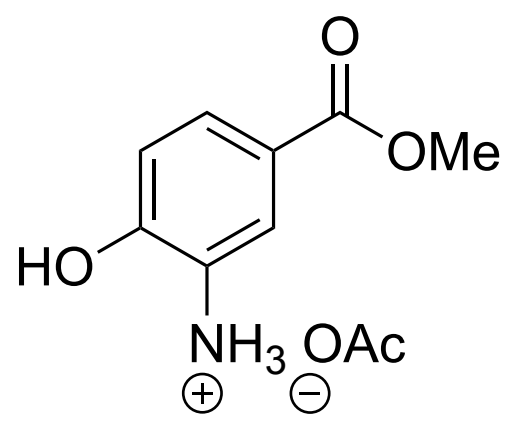

S19

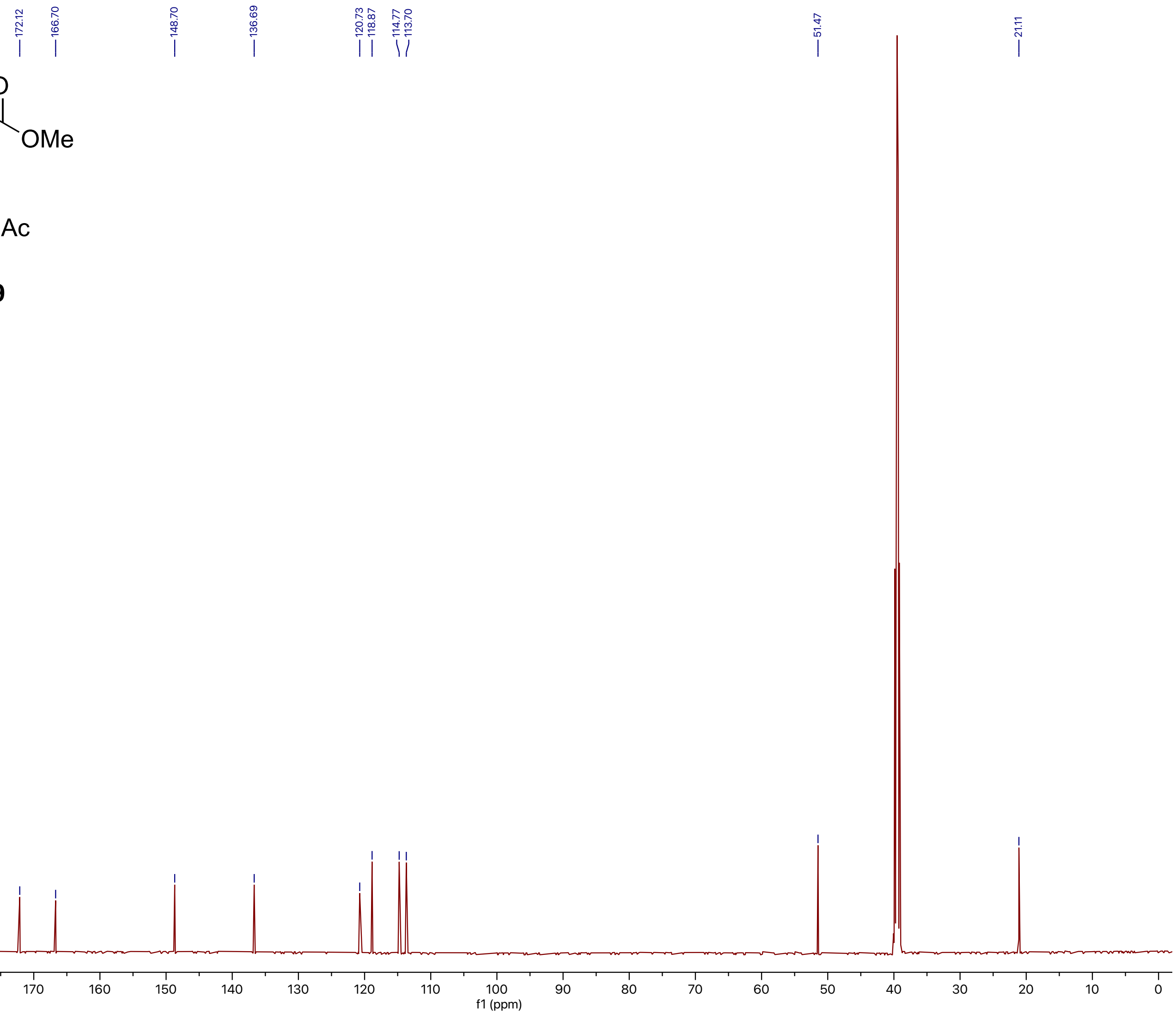

^{13}C-NMR (126 MHz, DMSO) of **S19**.

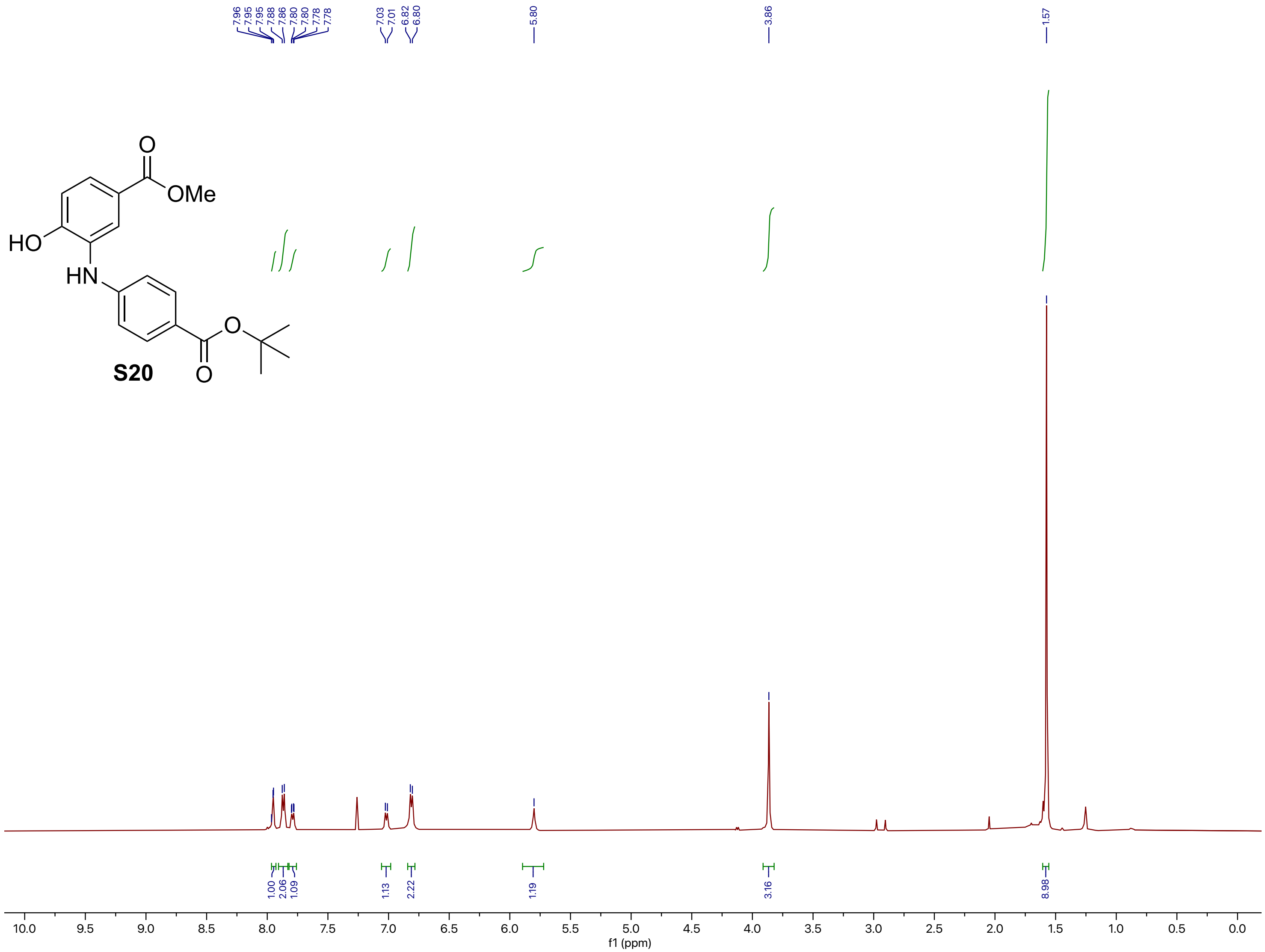

^{1}H-NMR (500 MHz, $CDCl_3$) of **S20**.

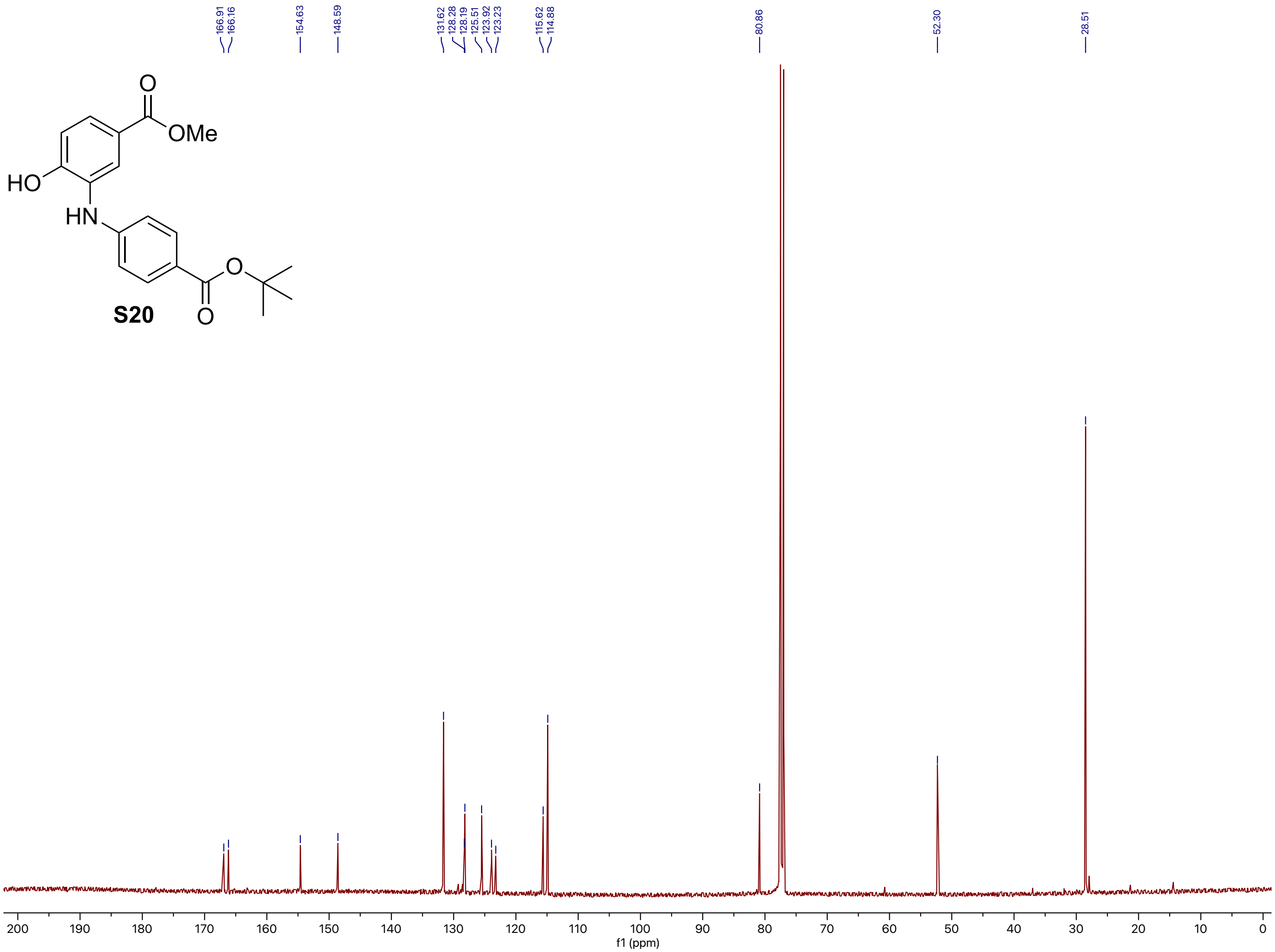

^{13}C-NMR (126 MHz, $CDCl_3$) of **S20**.

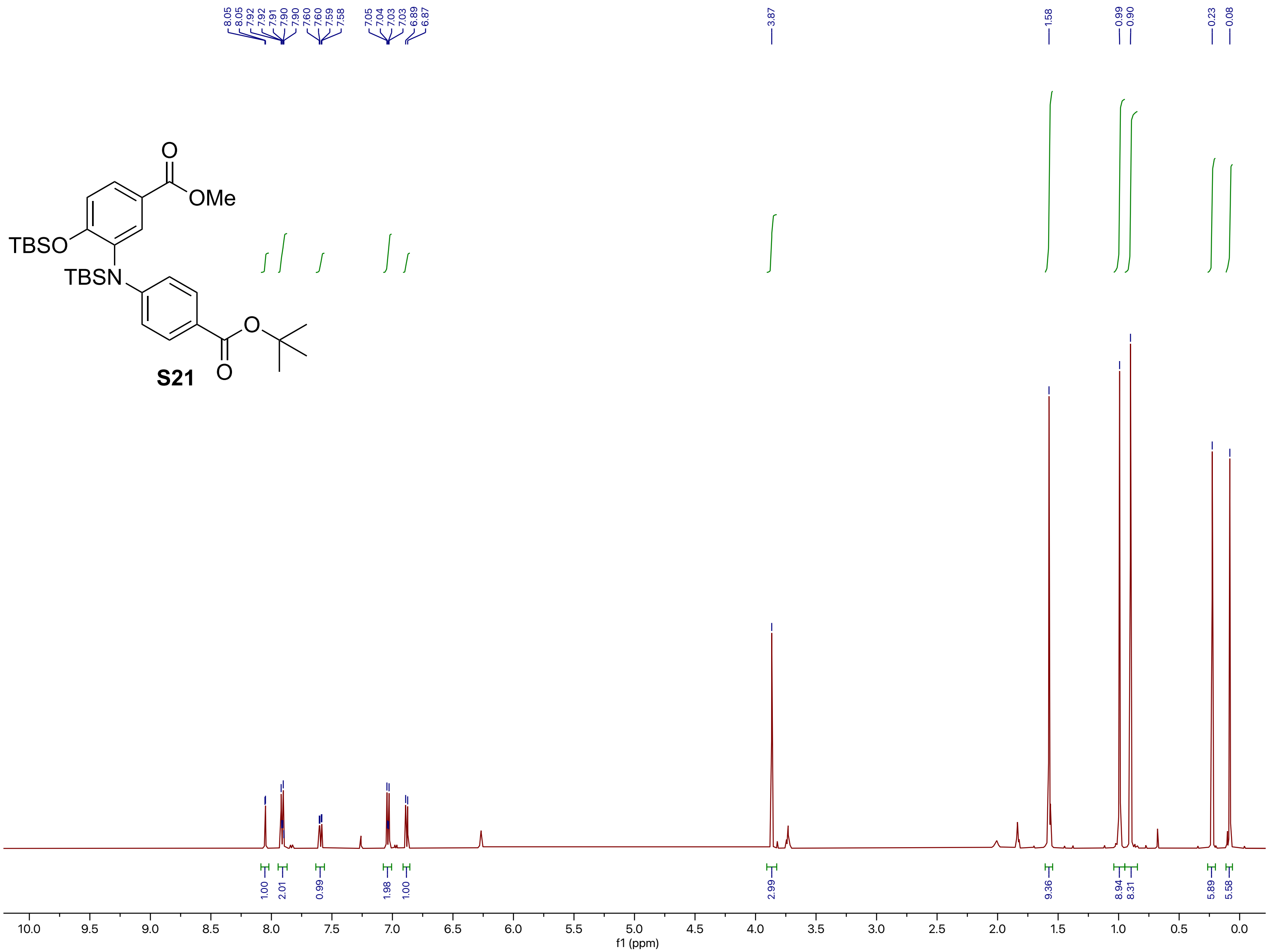

^{1}H-NMR (500 MHz, $CDCl_3$) of **S21**.

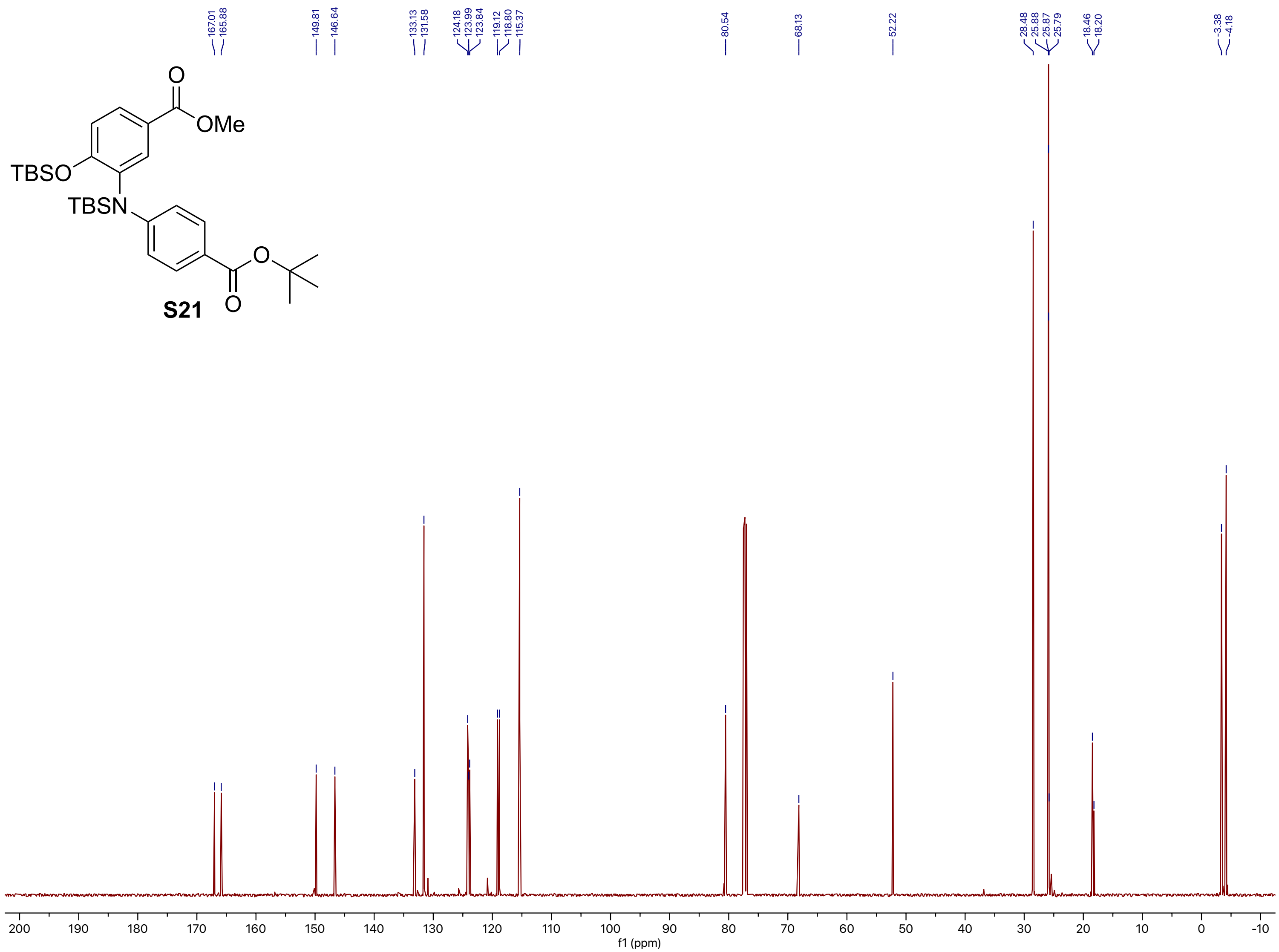

^{13}C-NMR (126 MHz, $CDCl_3$) of **S21**.

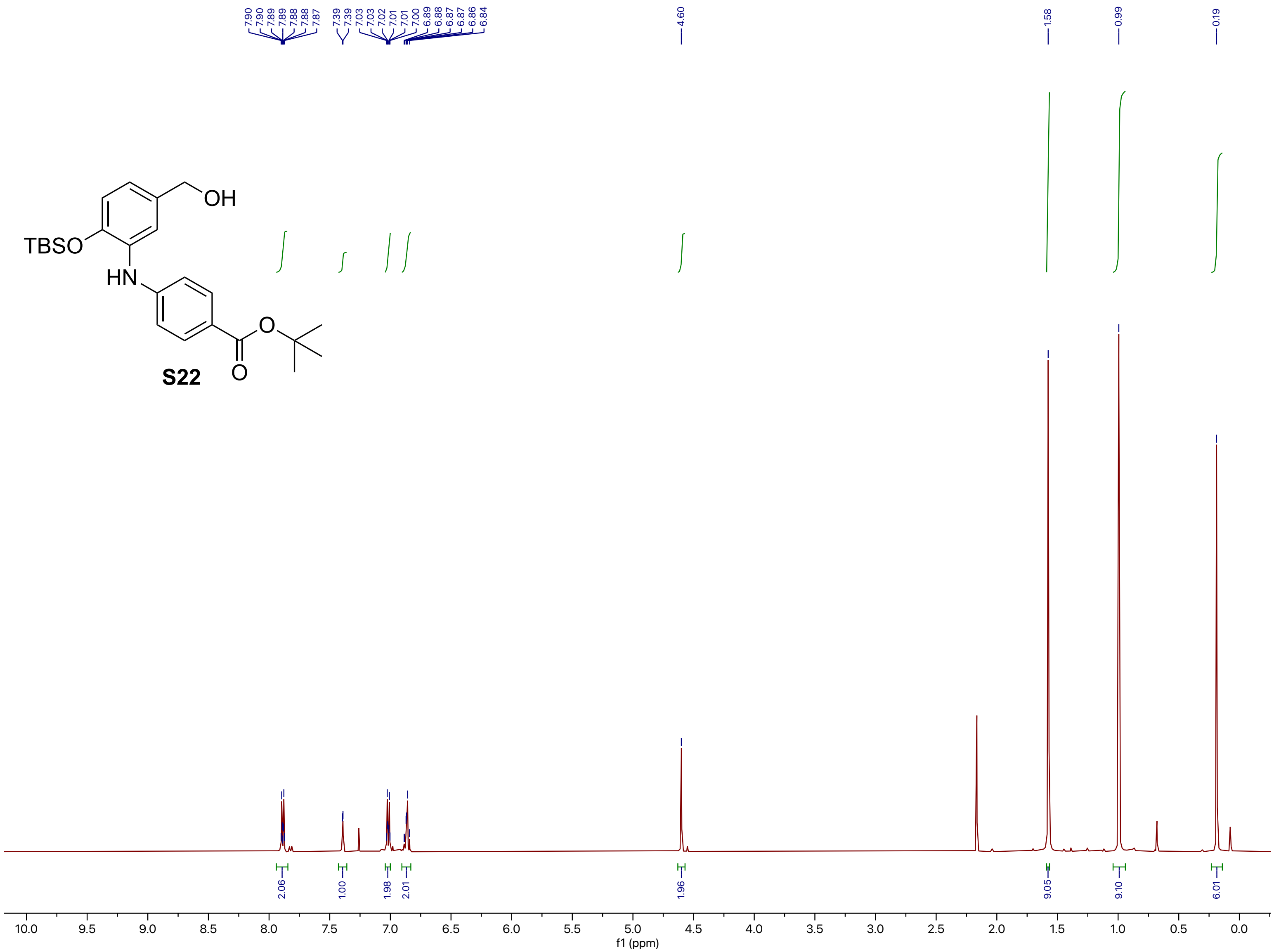

^{1}H-NMR (500 MHz, $CDCl_3$) of **S22**.

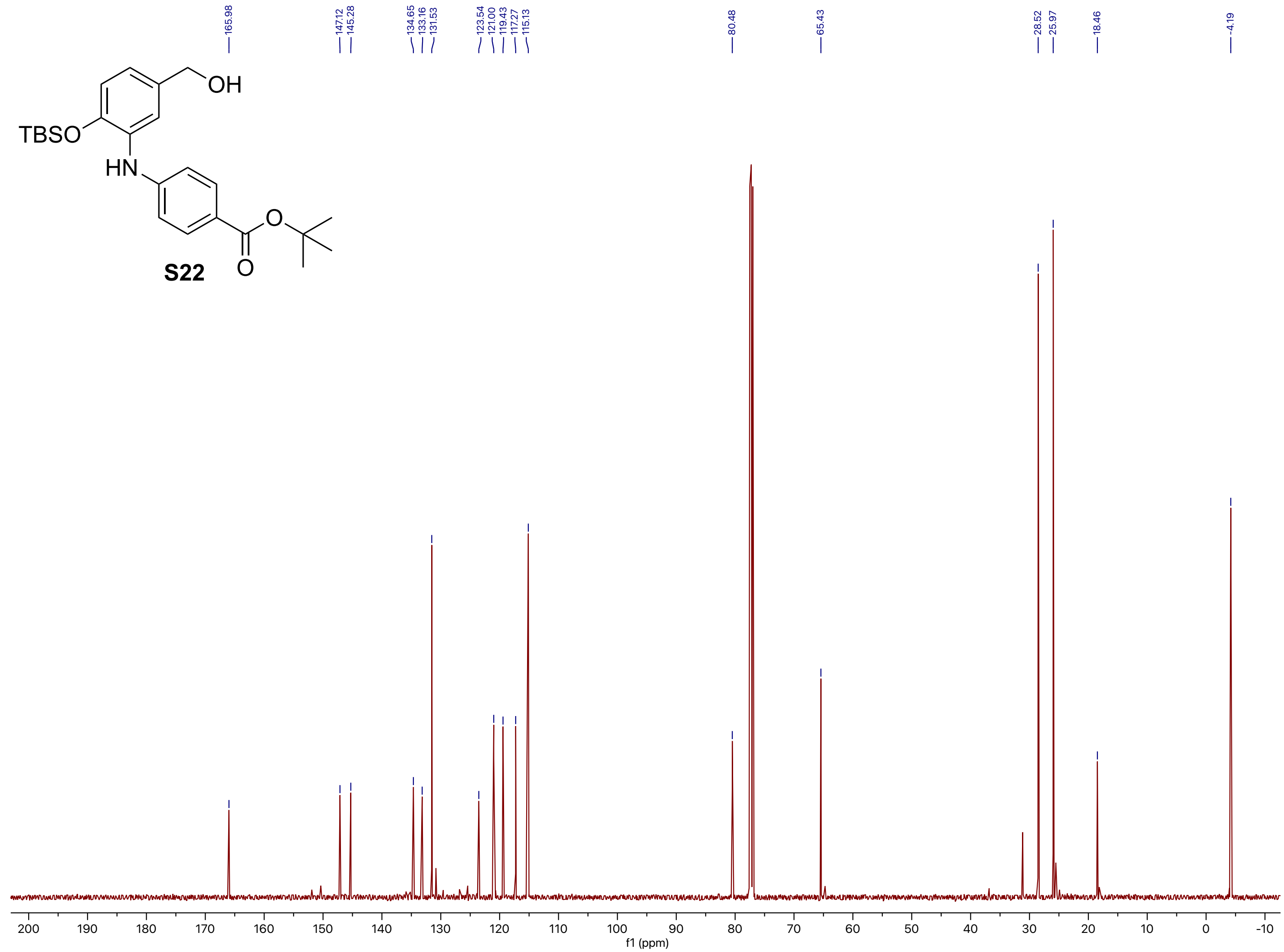

^{13}C-NMR (126 MHz, $CDCl_3$) of **S22**.

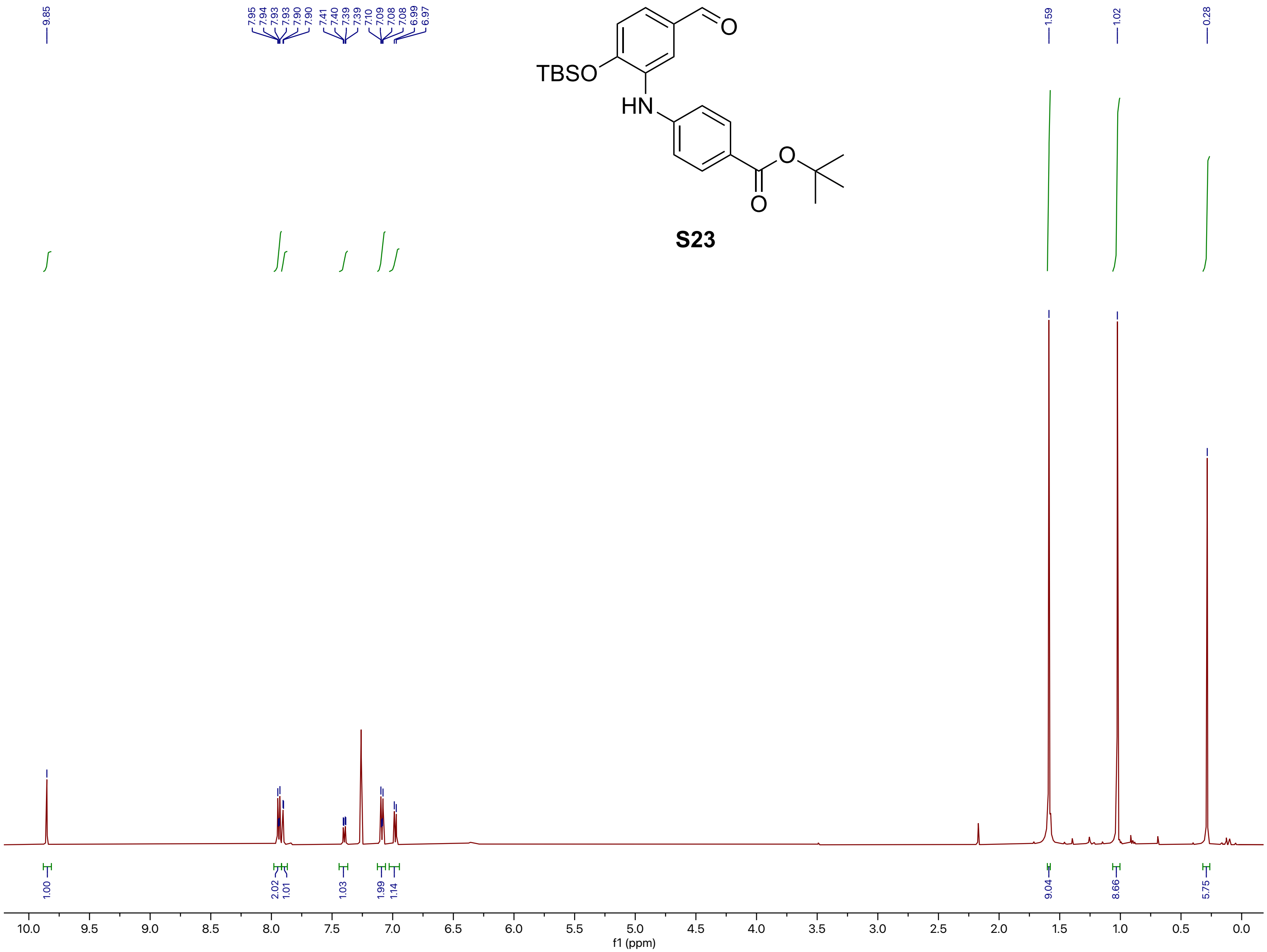

^{1}H-NMR (500 MHz, $CDCl_3$) of **S23**.

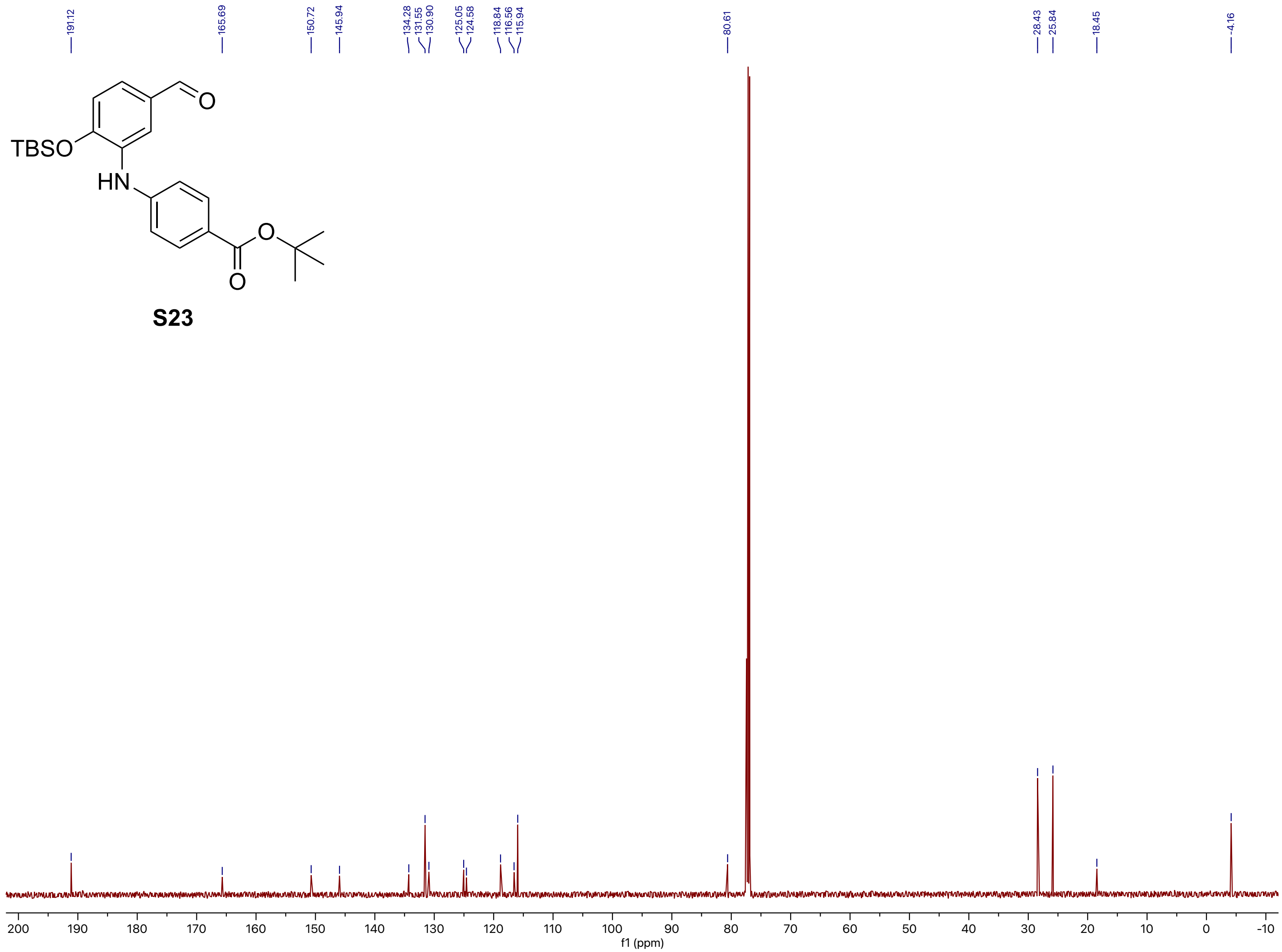

^{13}C-NMR (126 MHz, $CDCl_3$) of **S23**.

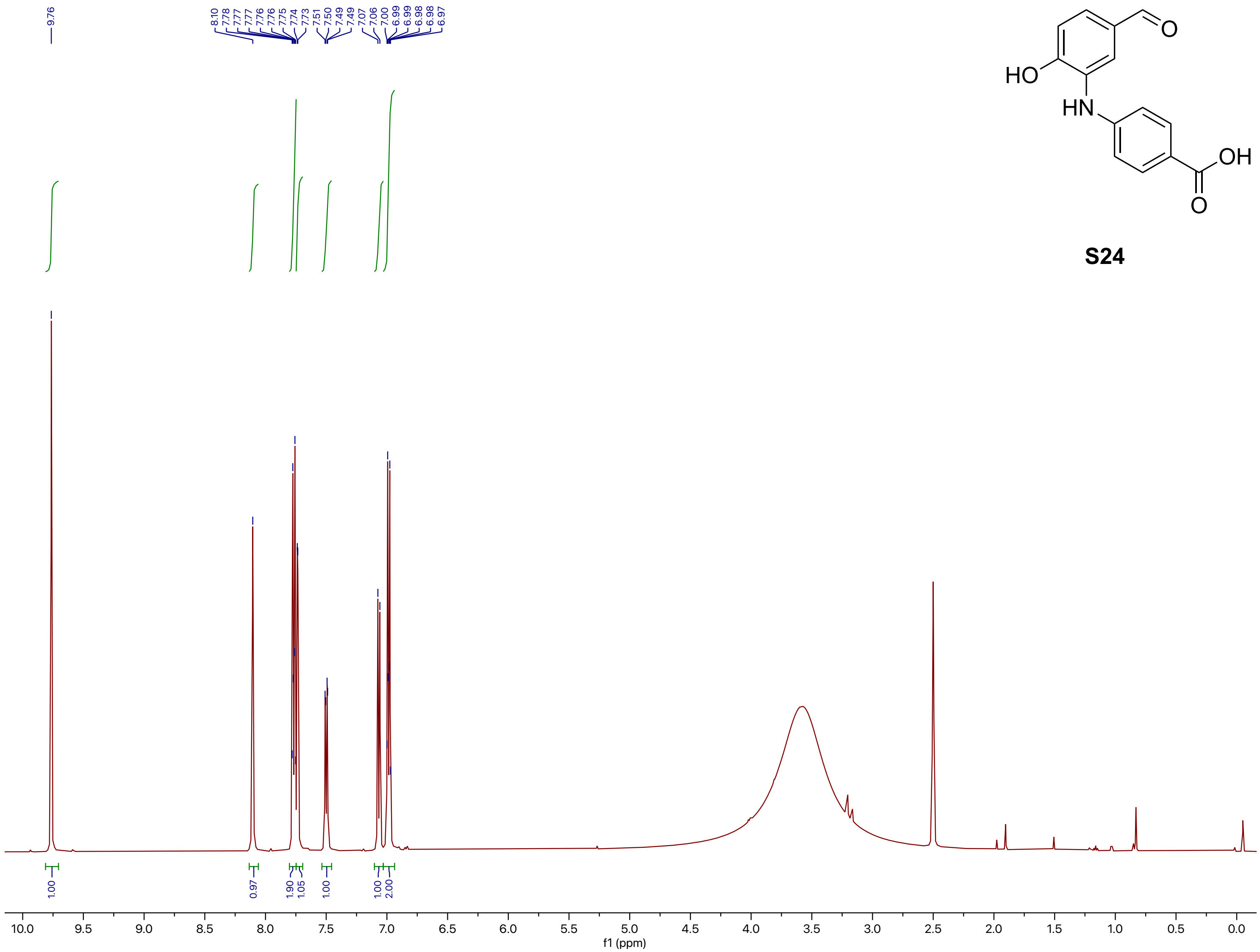

S24

^{1}H-NMR (500 MHz, DMSO) of **S24**.

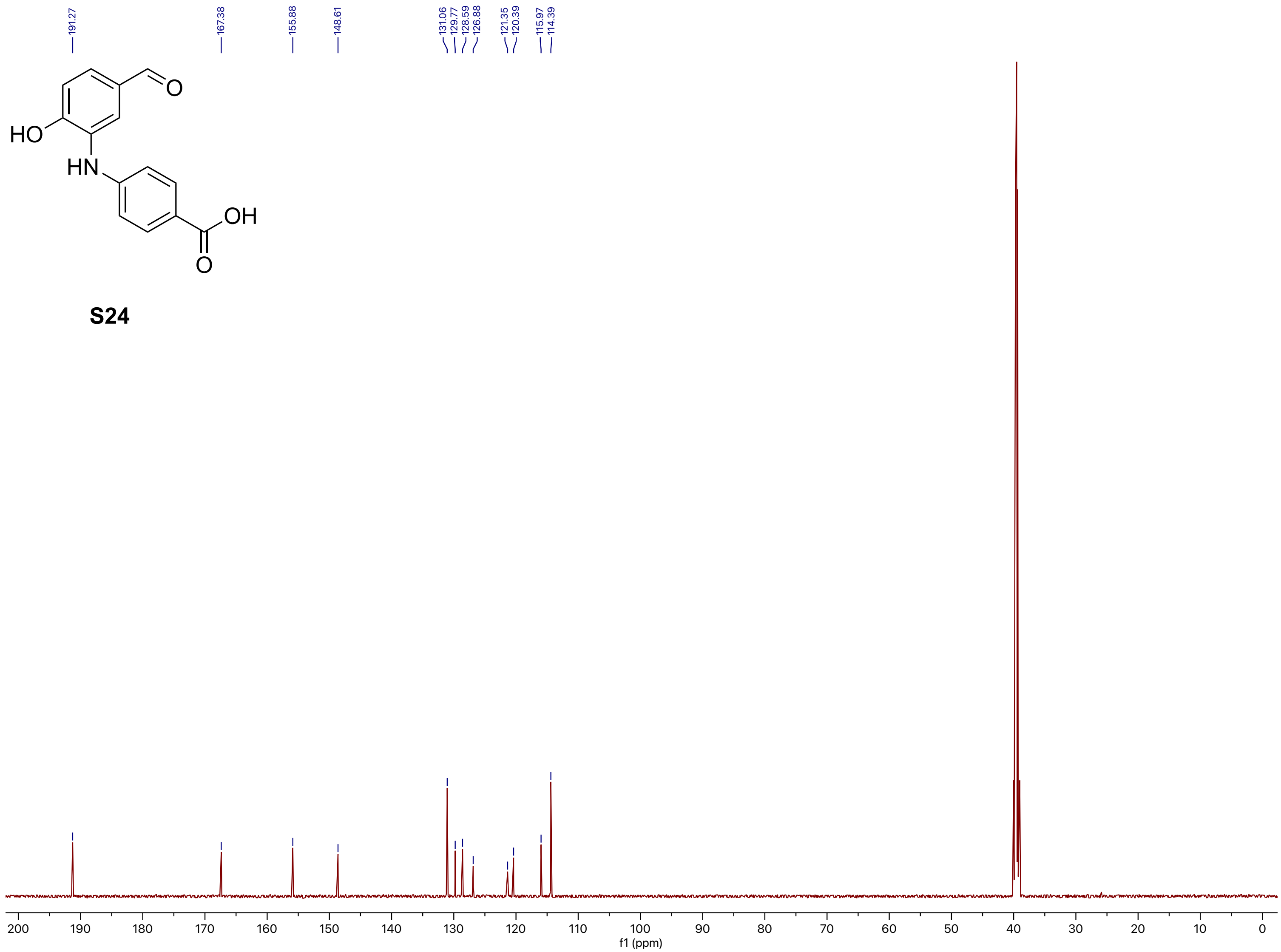

S24

^{13}C-NMR (126 MHz, DMSO) of **S24**.

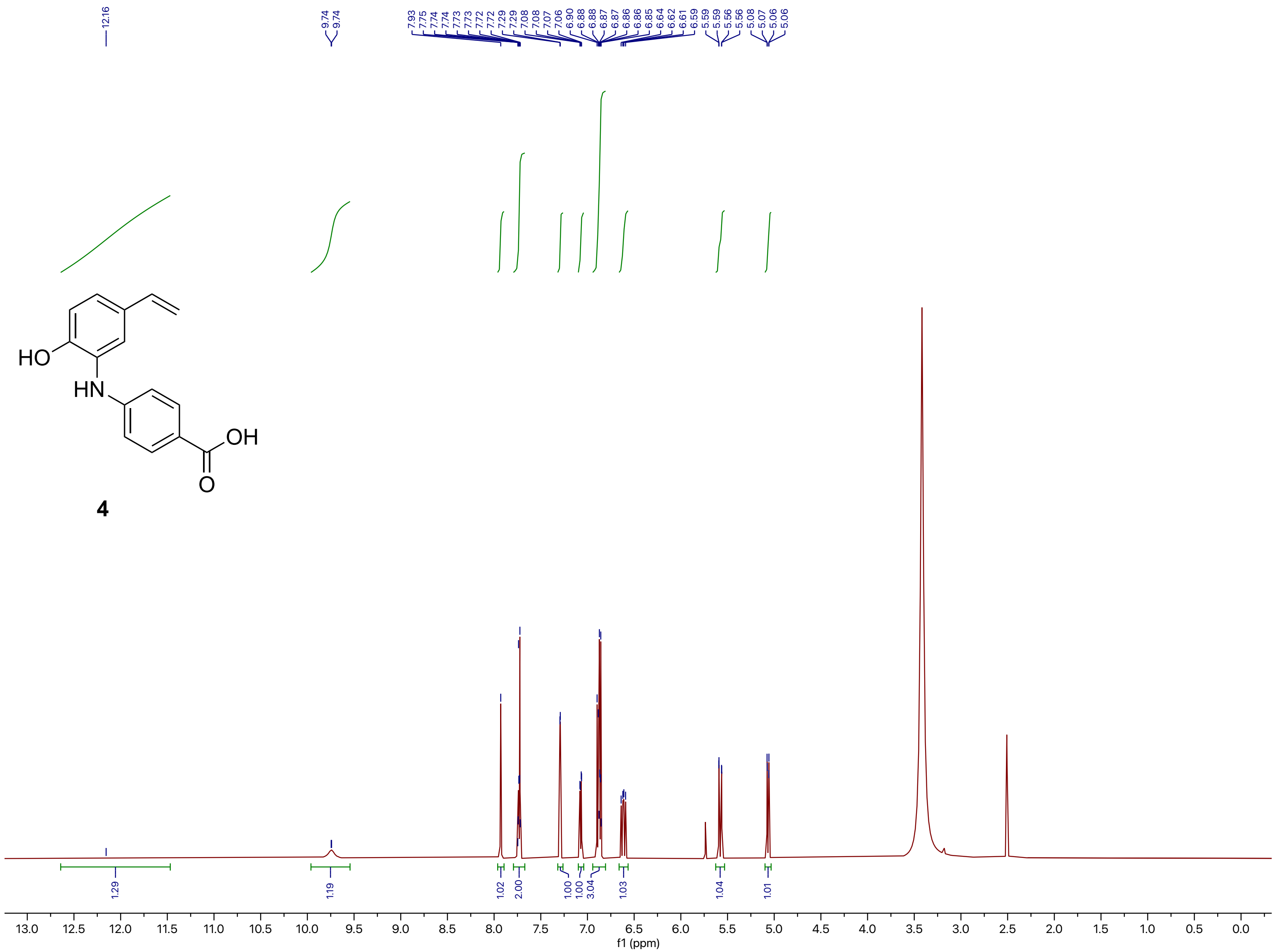

^{1}H-NMR (600 MHz, DMSO) of **4**.

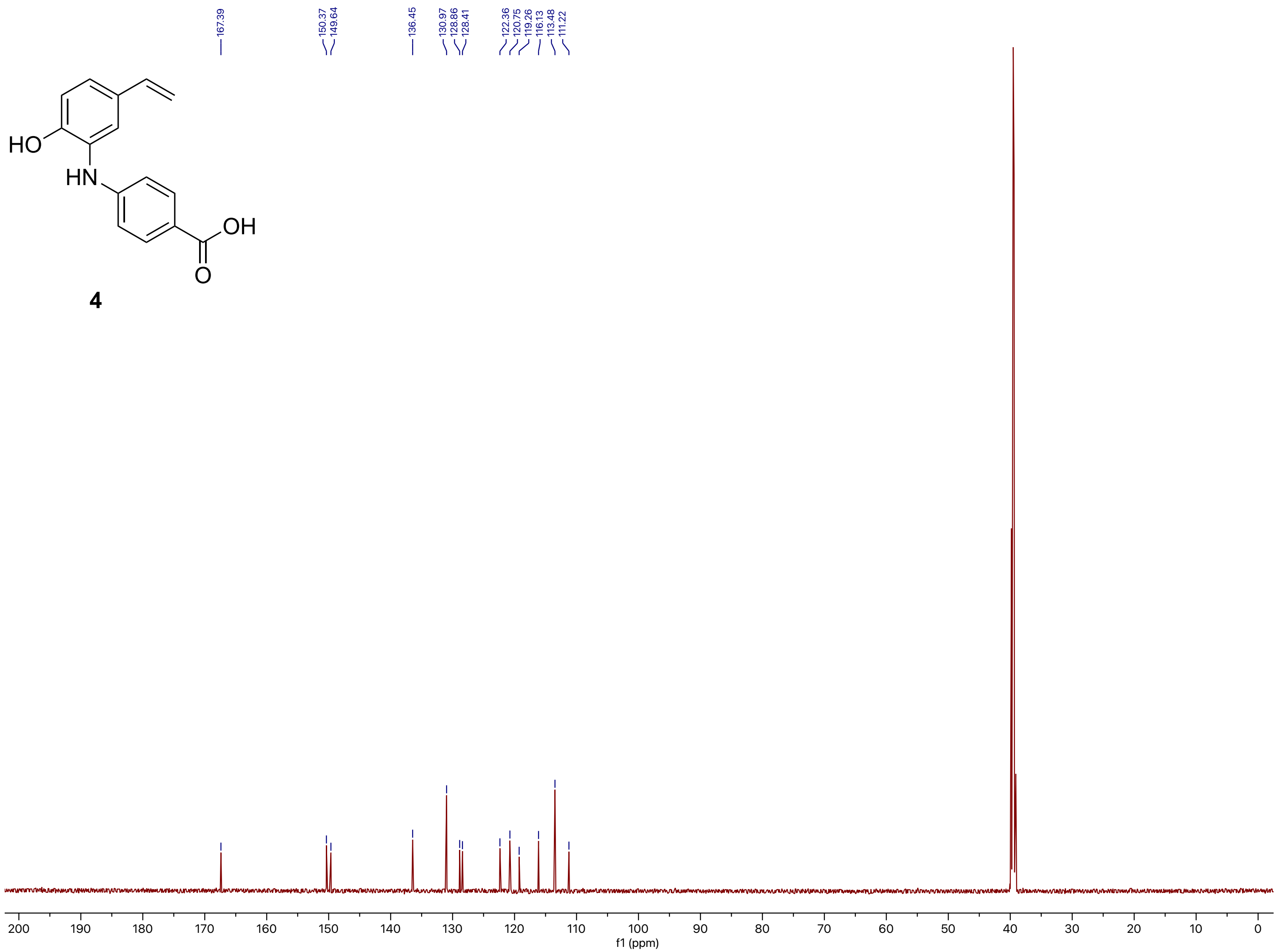

^{13}C-NMR (151 MHz, DMSO) of **4**.

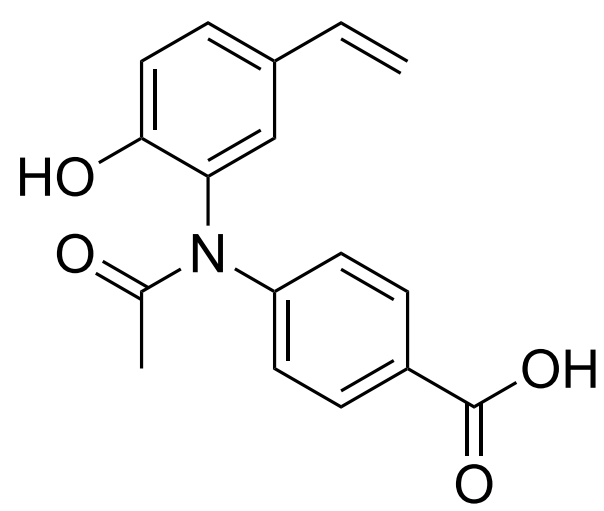

5

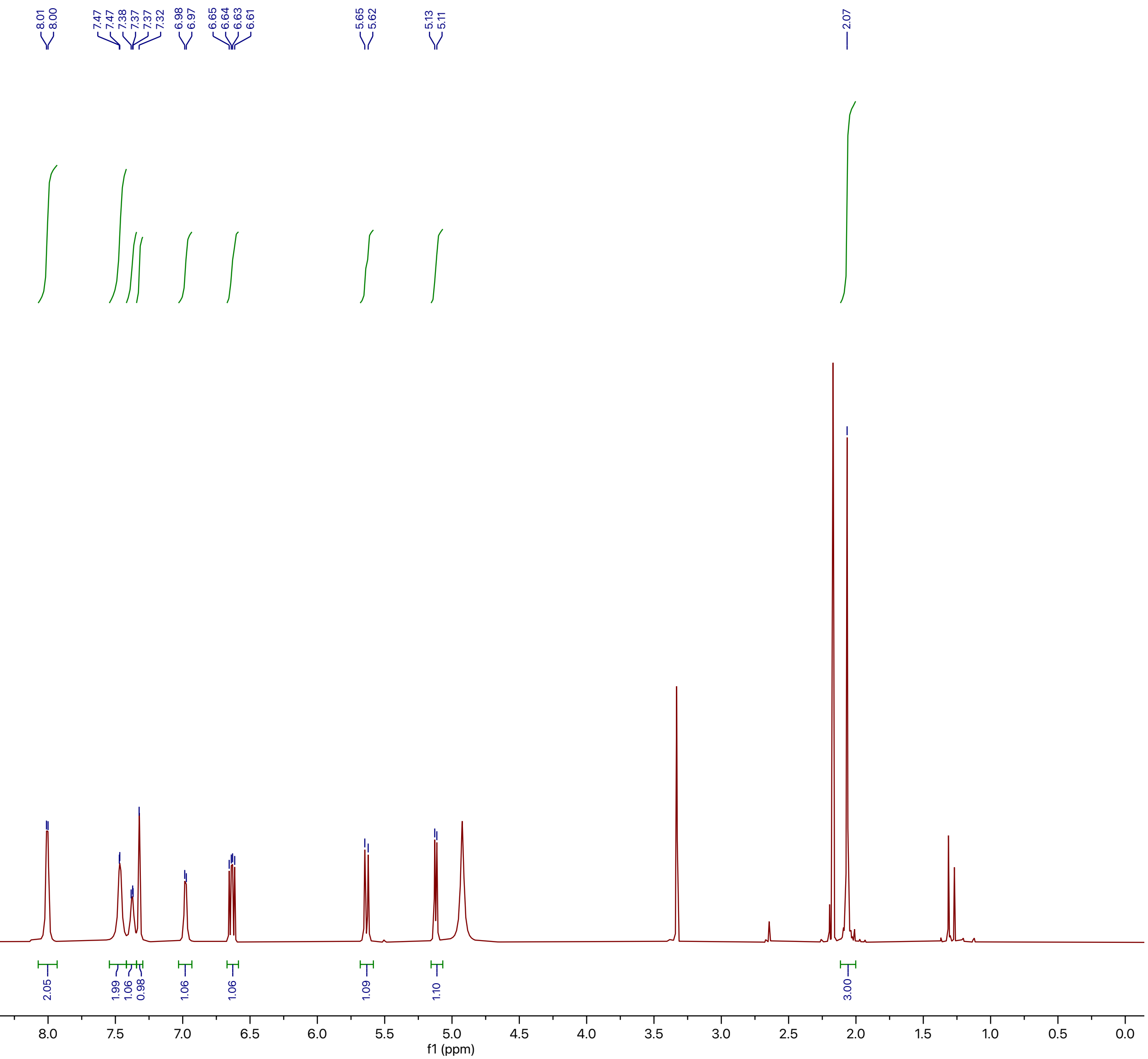

^{1}H-NMR (700 MHz, MeOD) of **5**.

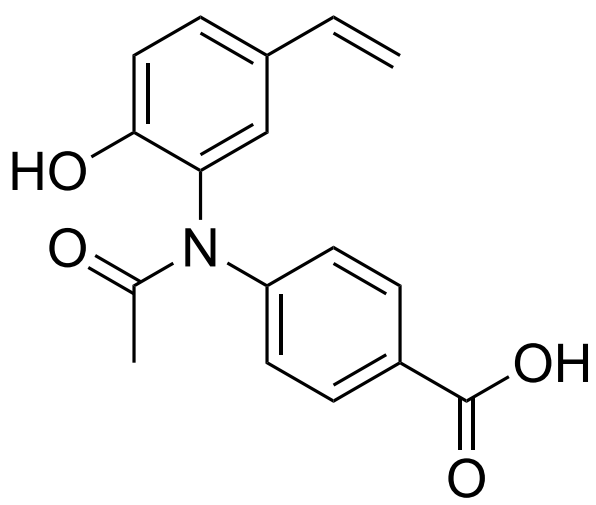

5

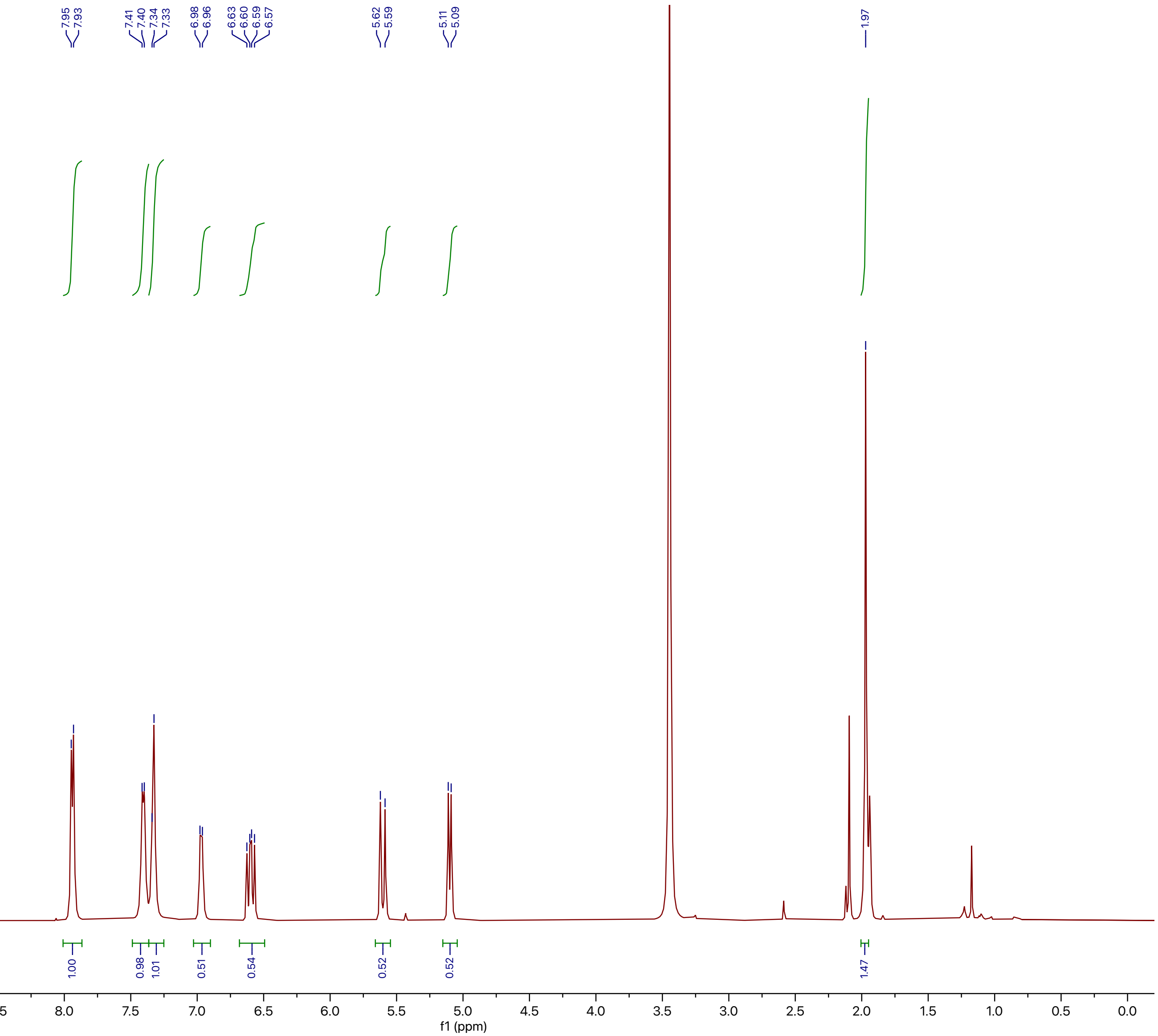

^{1}H-NMR (500 MHz, CD_3CN) of **5**.

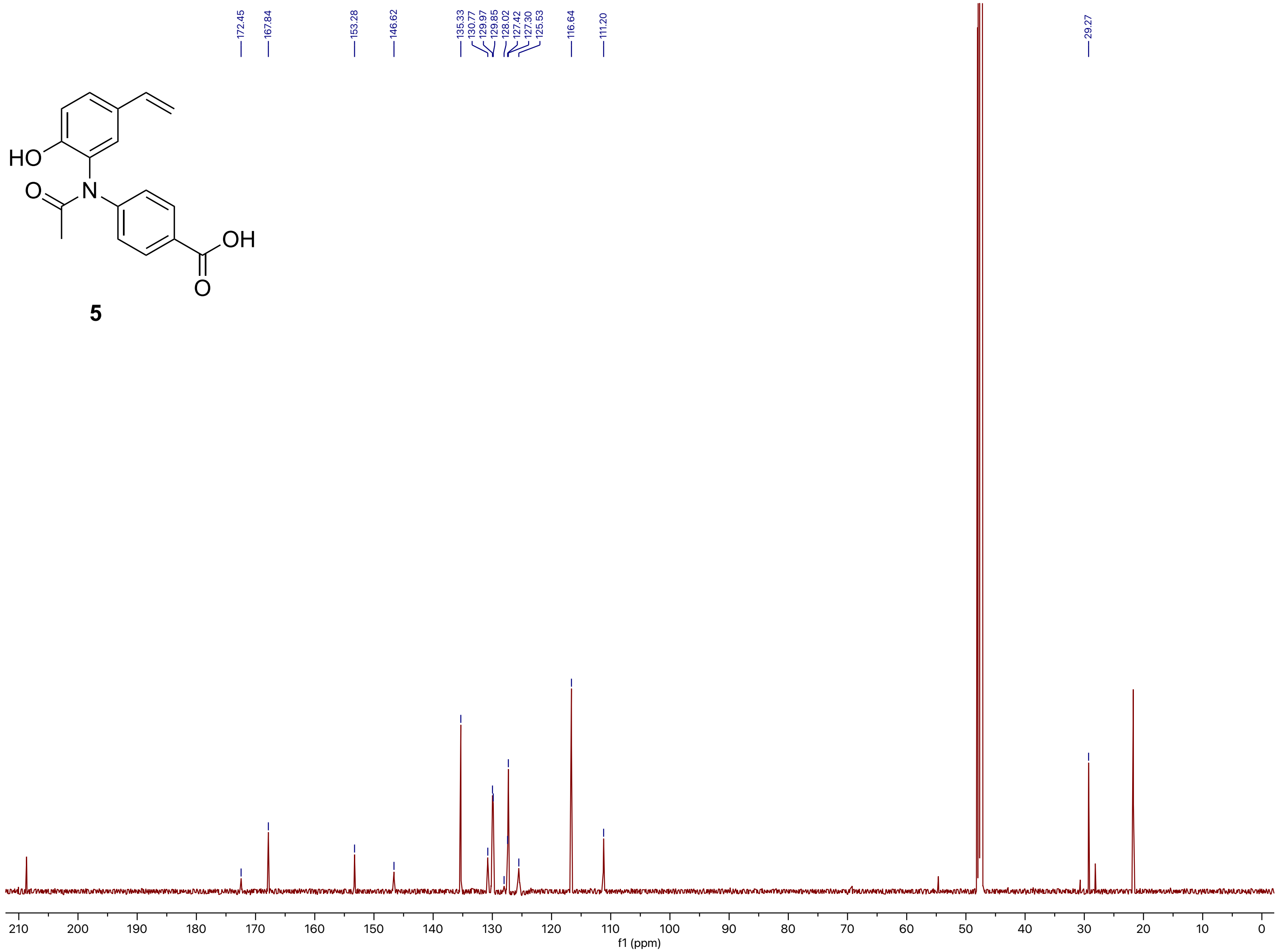

1H-NMR (151 MHz, MeOD) of **5**.

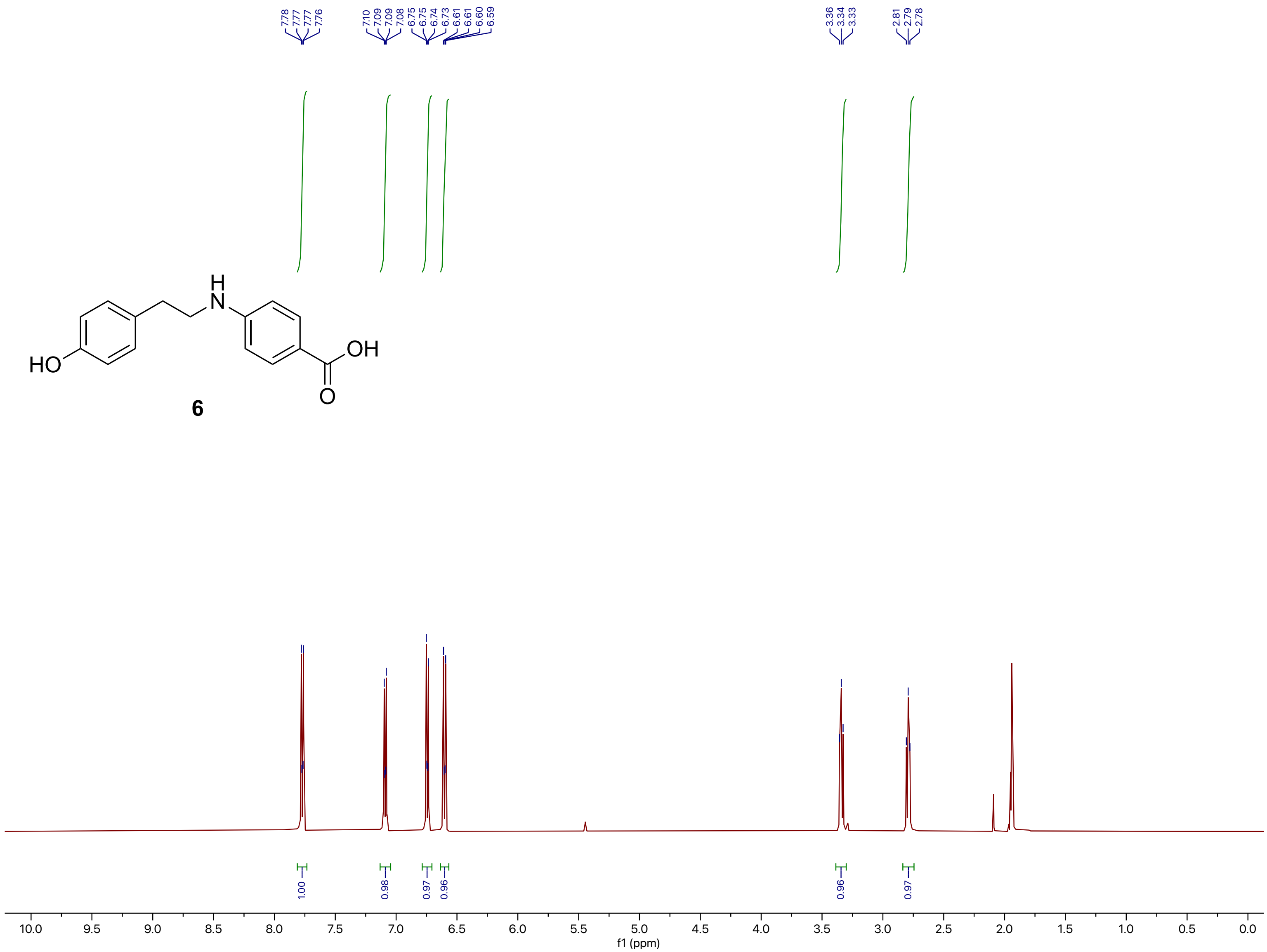

^{1}H-NMR (500 MHz, CD_3CN) of **6**.

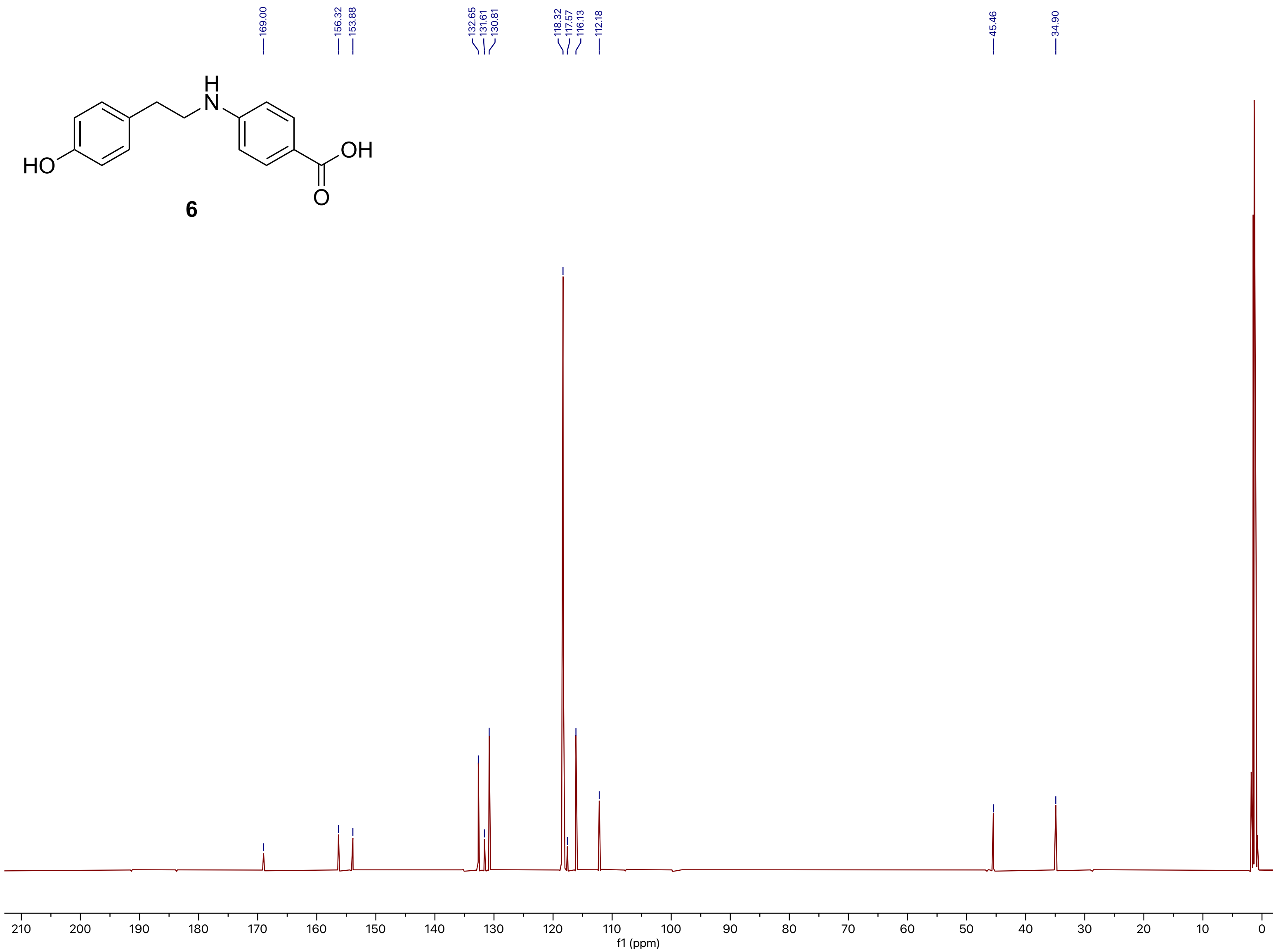

^{13}C-NMR (126 MHz, CD_3CN) of **6**.

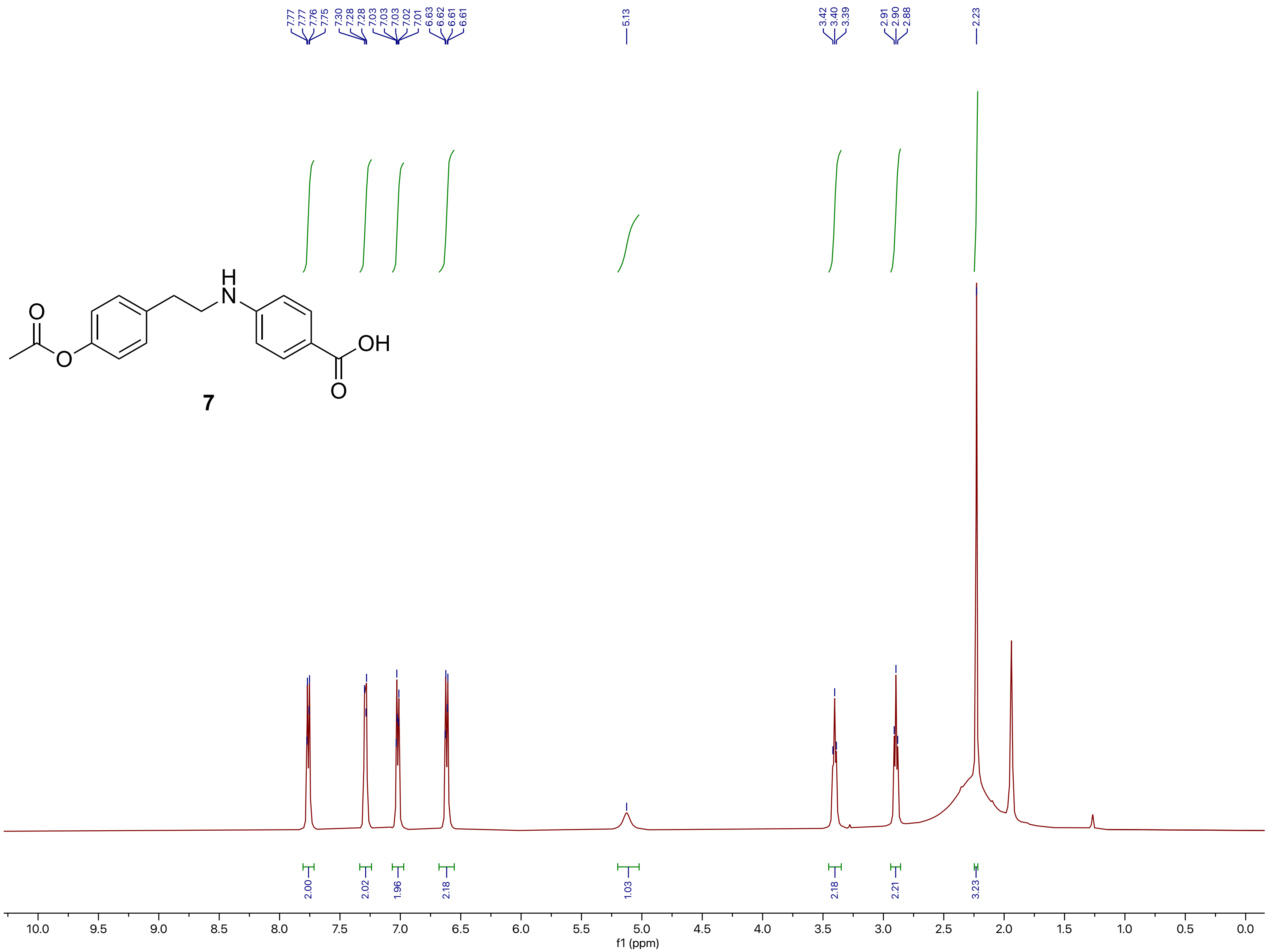

^{1}H-NMR (500 MHz, CD_3CN) of **7**.

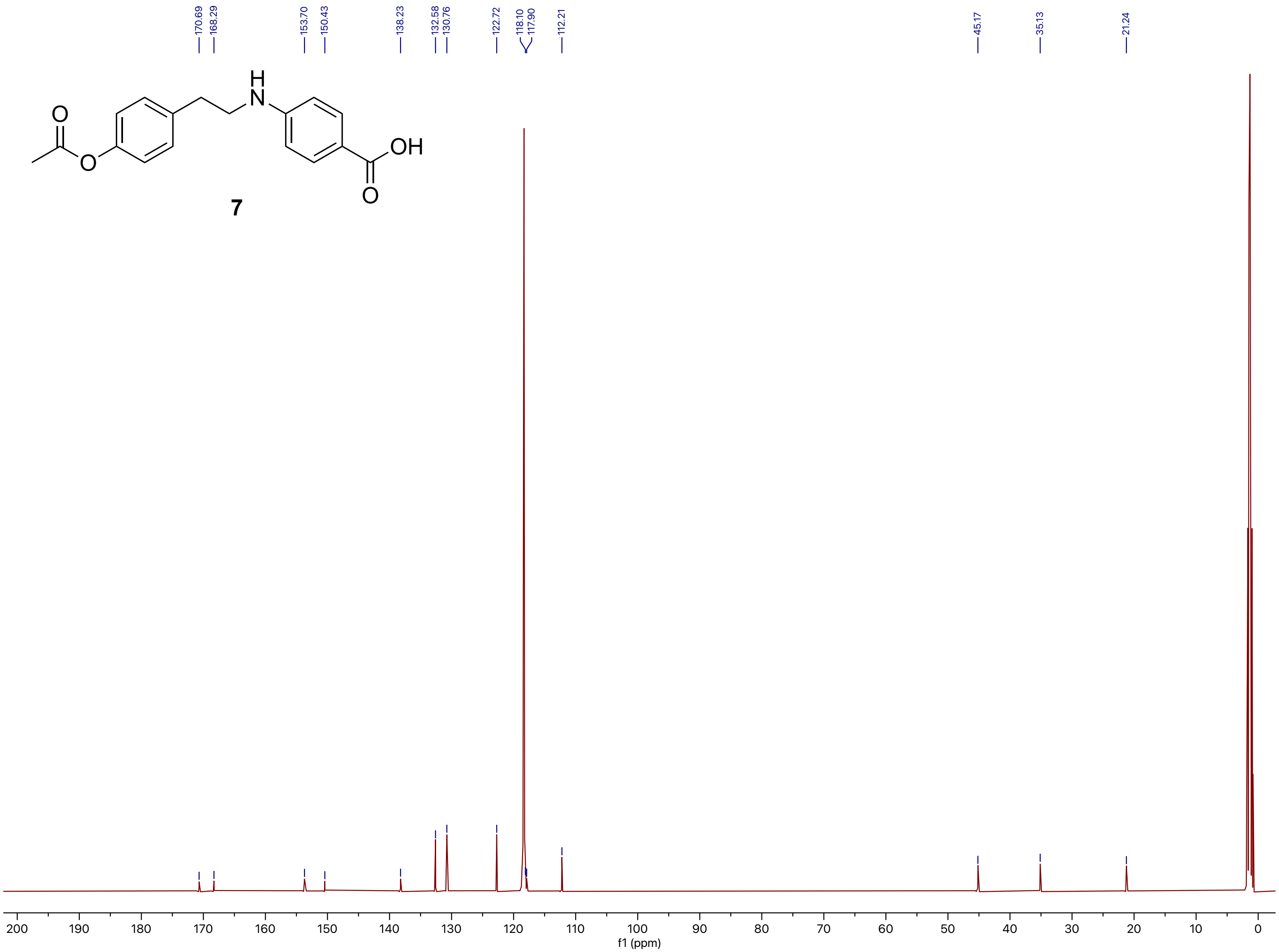

^{13}C-NMR (126 MHz, CD_3CN) of **7**.

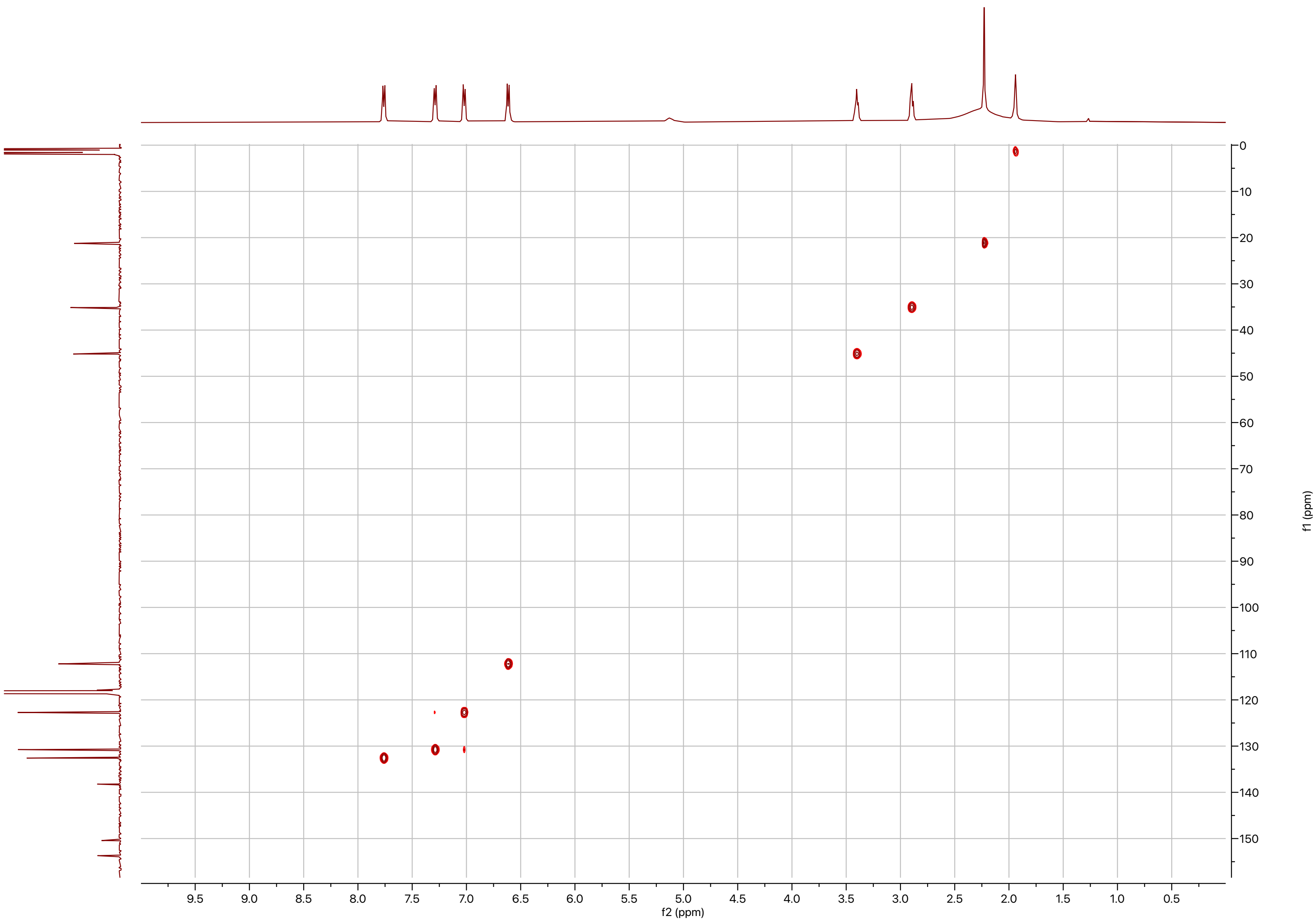

^{1}H-^{13}C HSQC (500 MHz, 126 MHz, CD_3CN) of **7**.

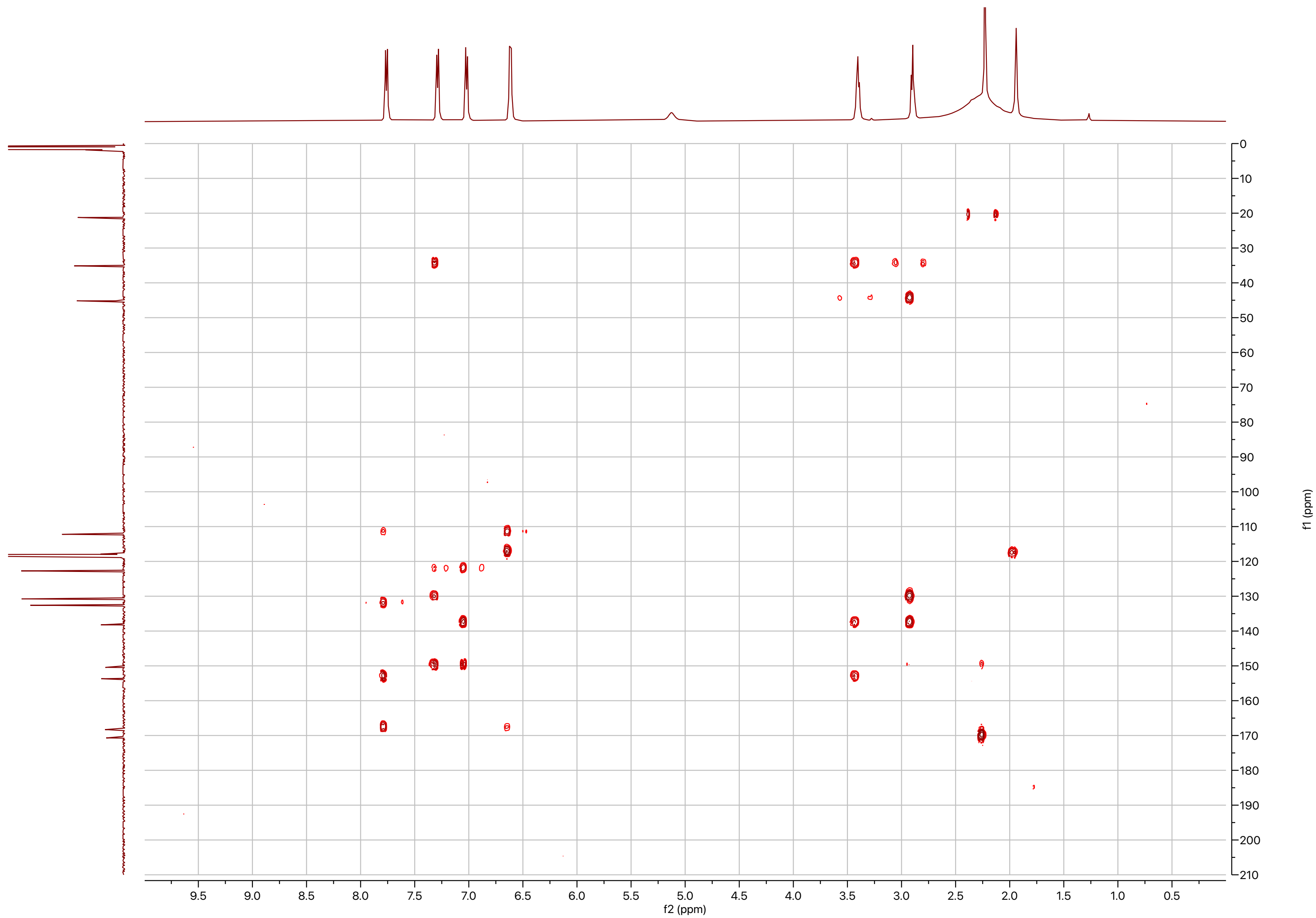

^{1}H-^{13}C HMBC (500 MHz, 126 MHz, CD_3CN) of **7**.

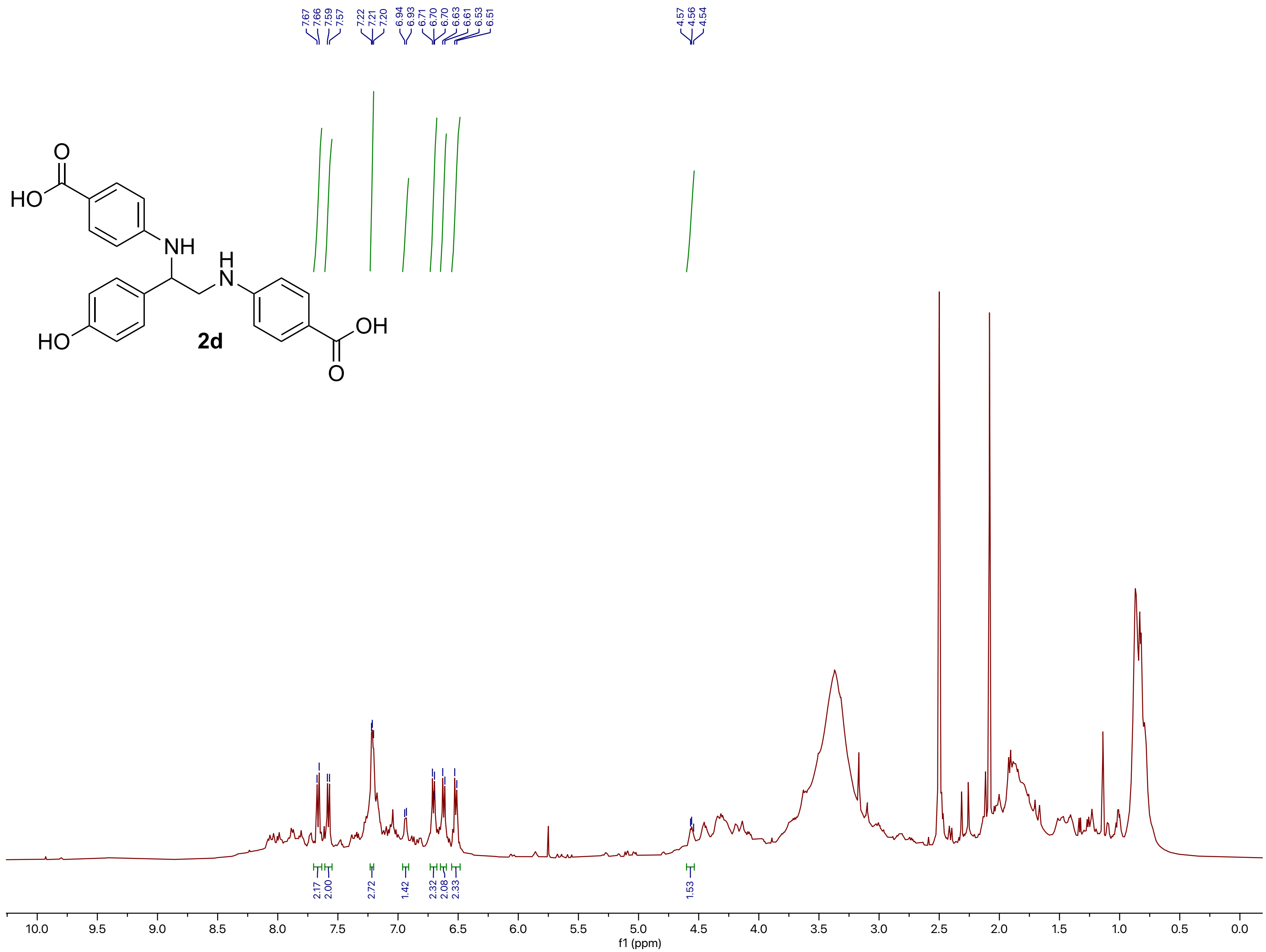

^{1}H-NMR (500 MHz, DMSO) of **2d** derived from biosynthesis.

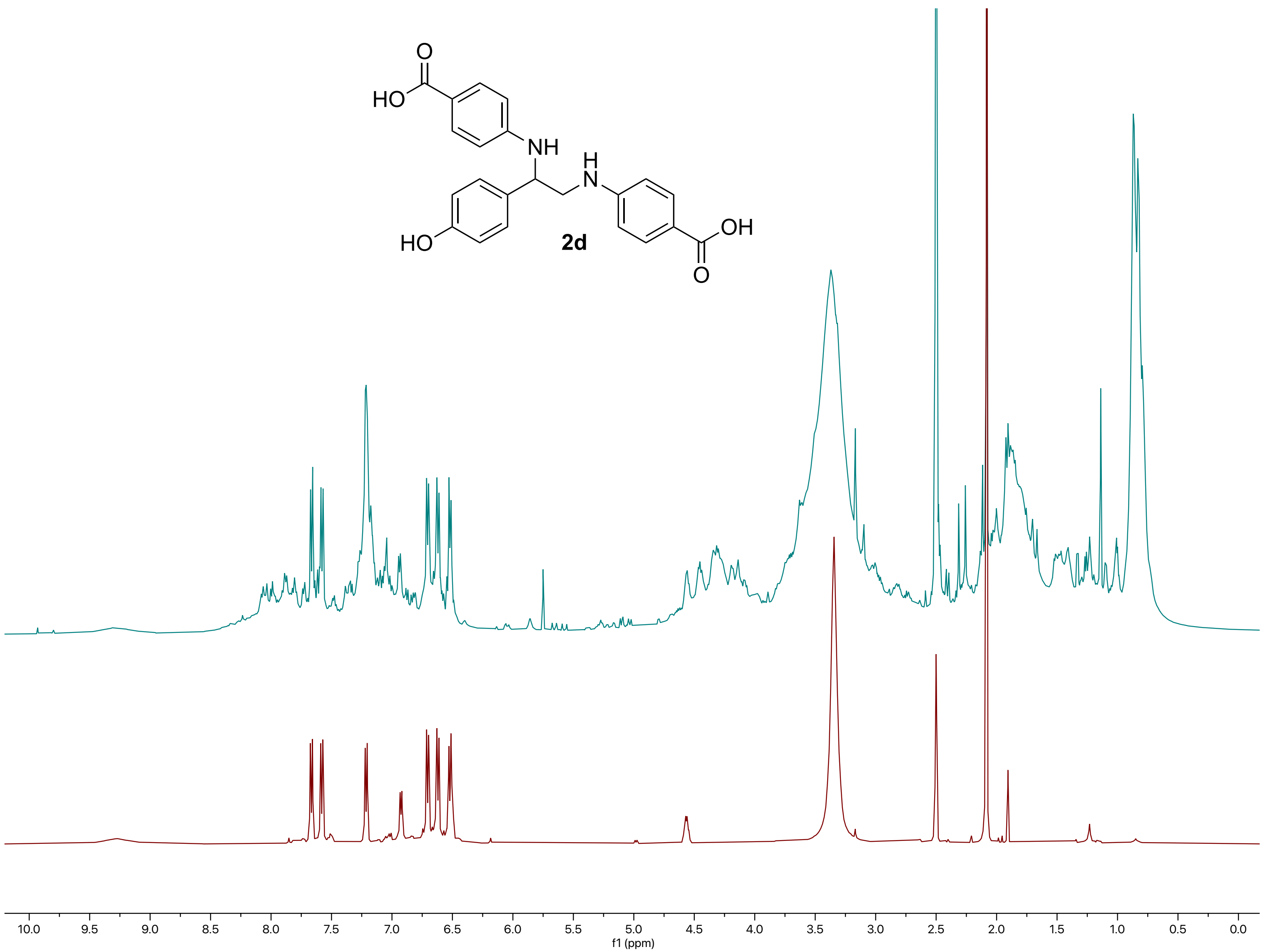

^{1}H-NMR (500 MHz, DMSO) of **2d** derived from biosynthesis (top) compared with ^{1}H-NMR (500 MHz, DMSO) of **2d** derived from independent synthesis (bottom).

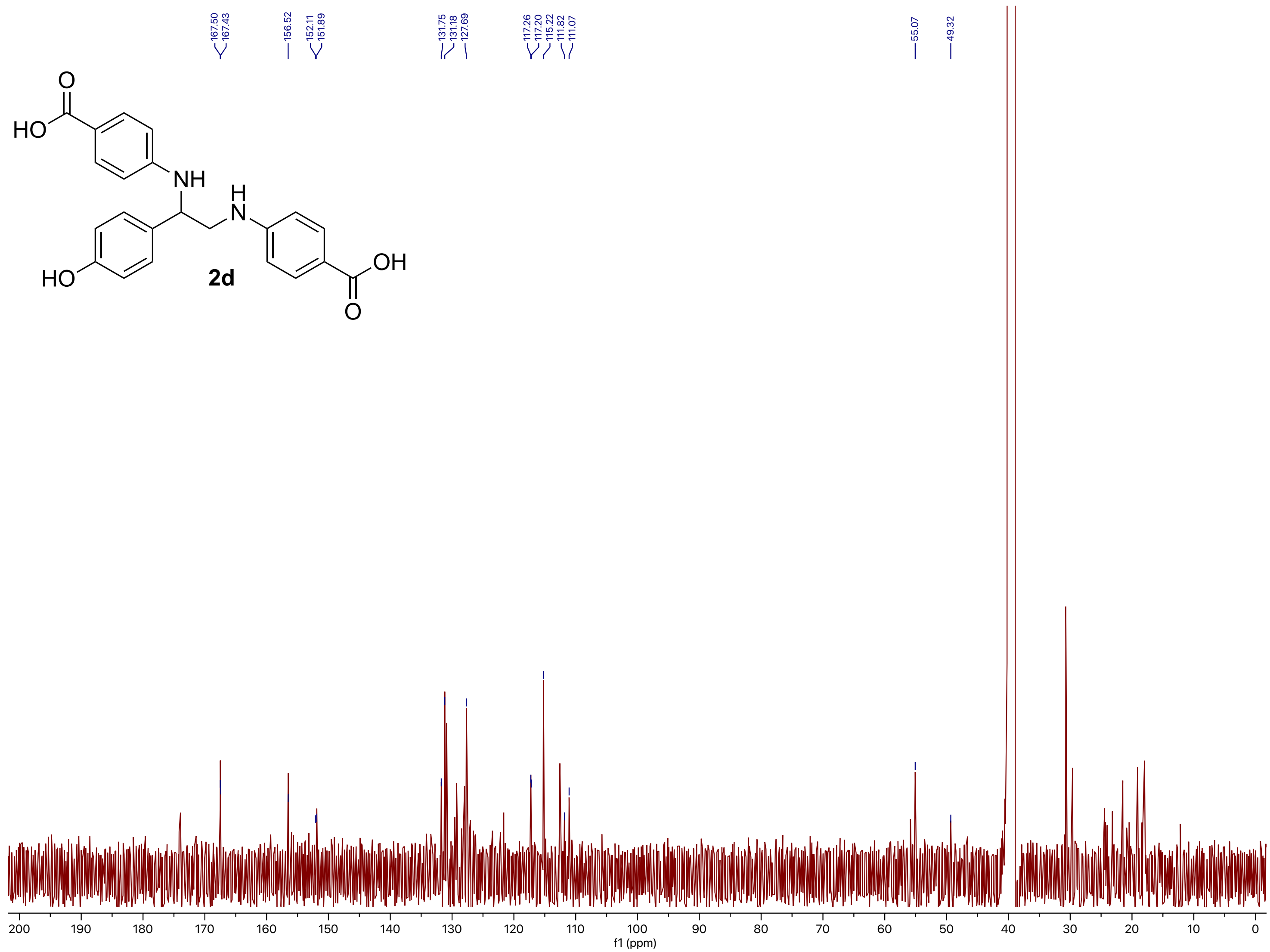

^{13}C-NMR (126 MHz, DMSO) of **2d** derived from biosynthesis.

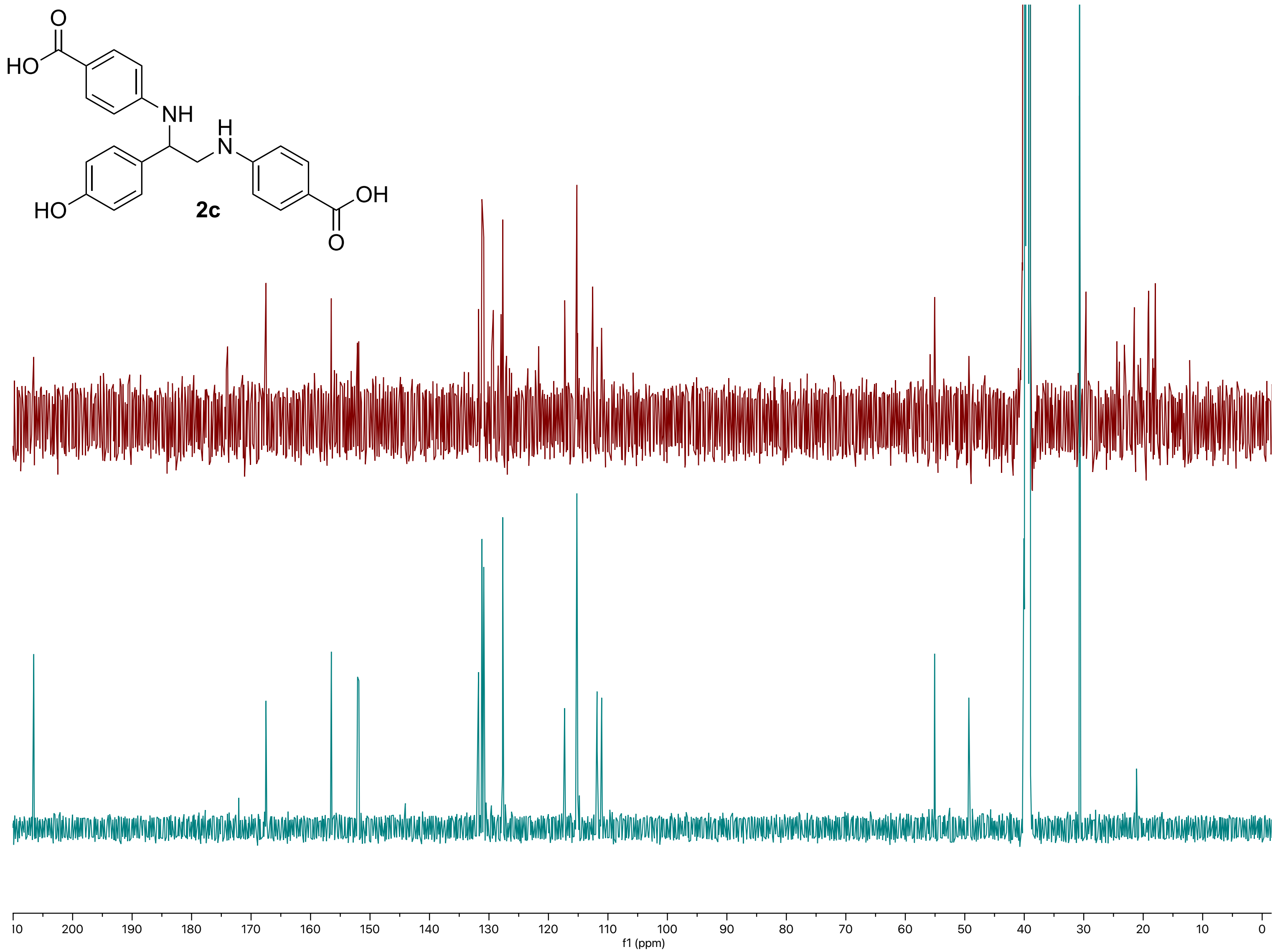

^{13}C-NMR (126 MHz, DMSO) of **2d** derived from biosynthesis (top) compared with ^{13}C-NMR (126 MHz, DMSO) of **2d** derived from independent synthesis (bottom).

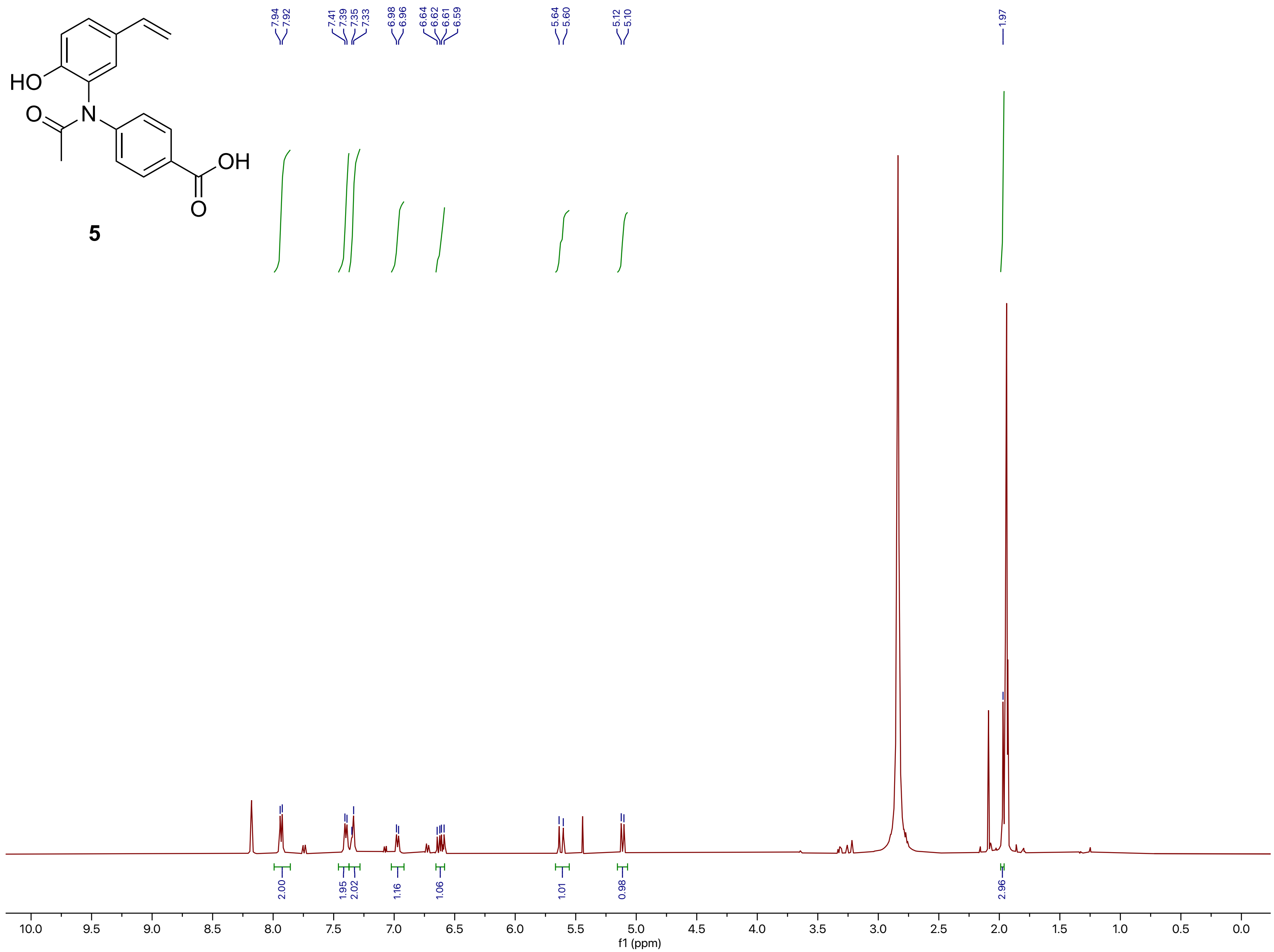

^{1}H-NMR (600 MHz, CD_3CN) of **5** derived from biosynthesis.

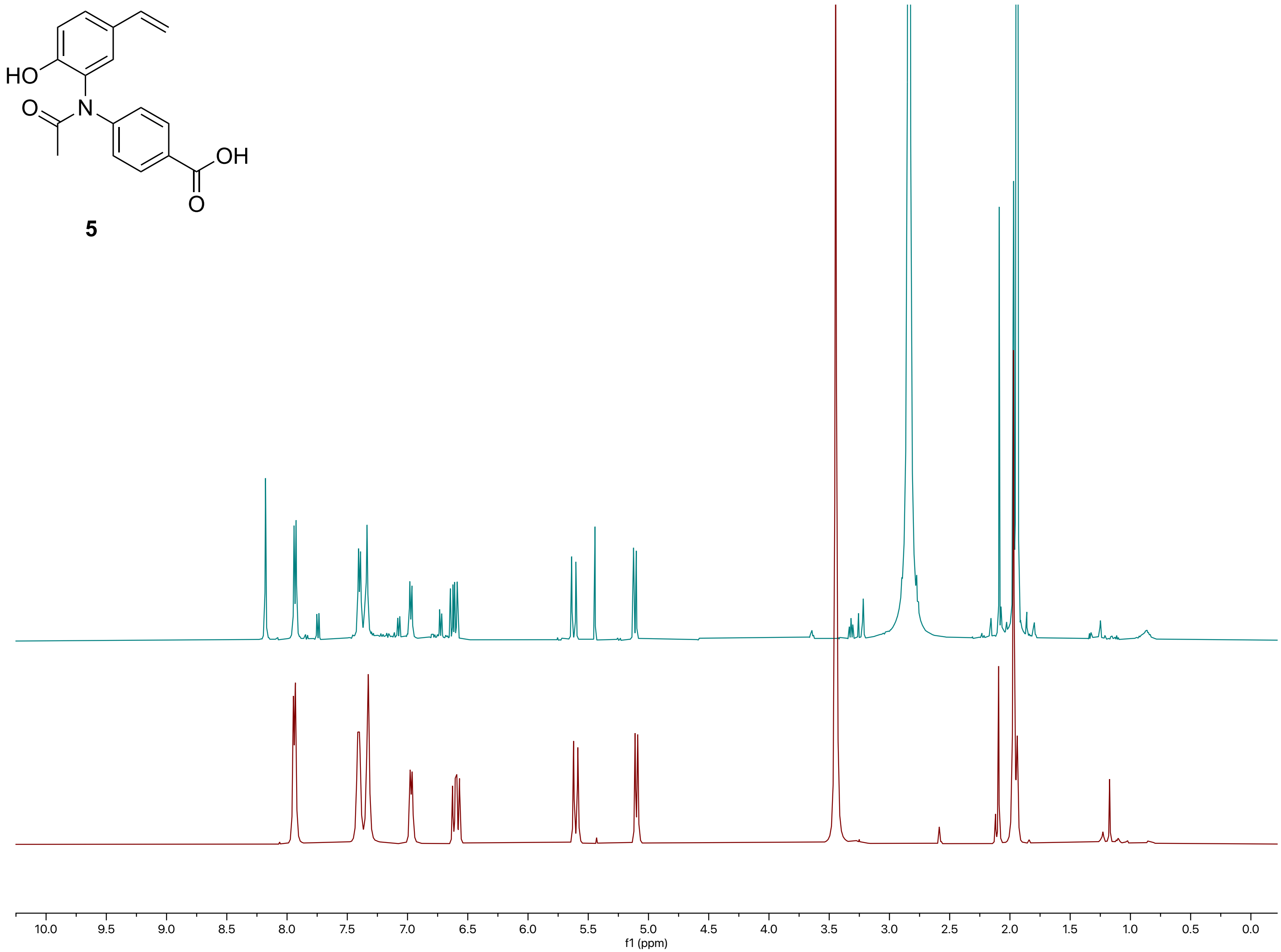

5

^{1}H-NMR (600 MHz, CD_3CN) of **5** derived from biosynthesis (top) compared with ^{1}H-NMR (500 MHz, CD_3CN) of **5** derived from independent synthesis (bottom).

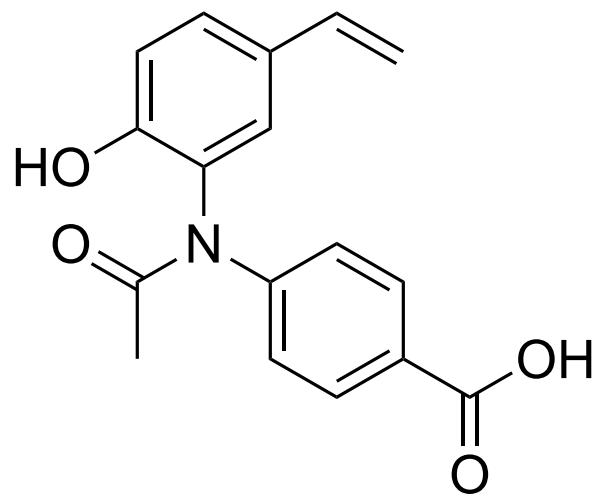

5

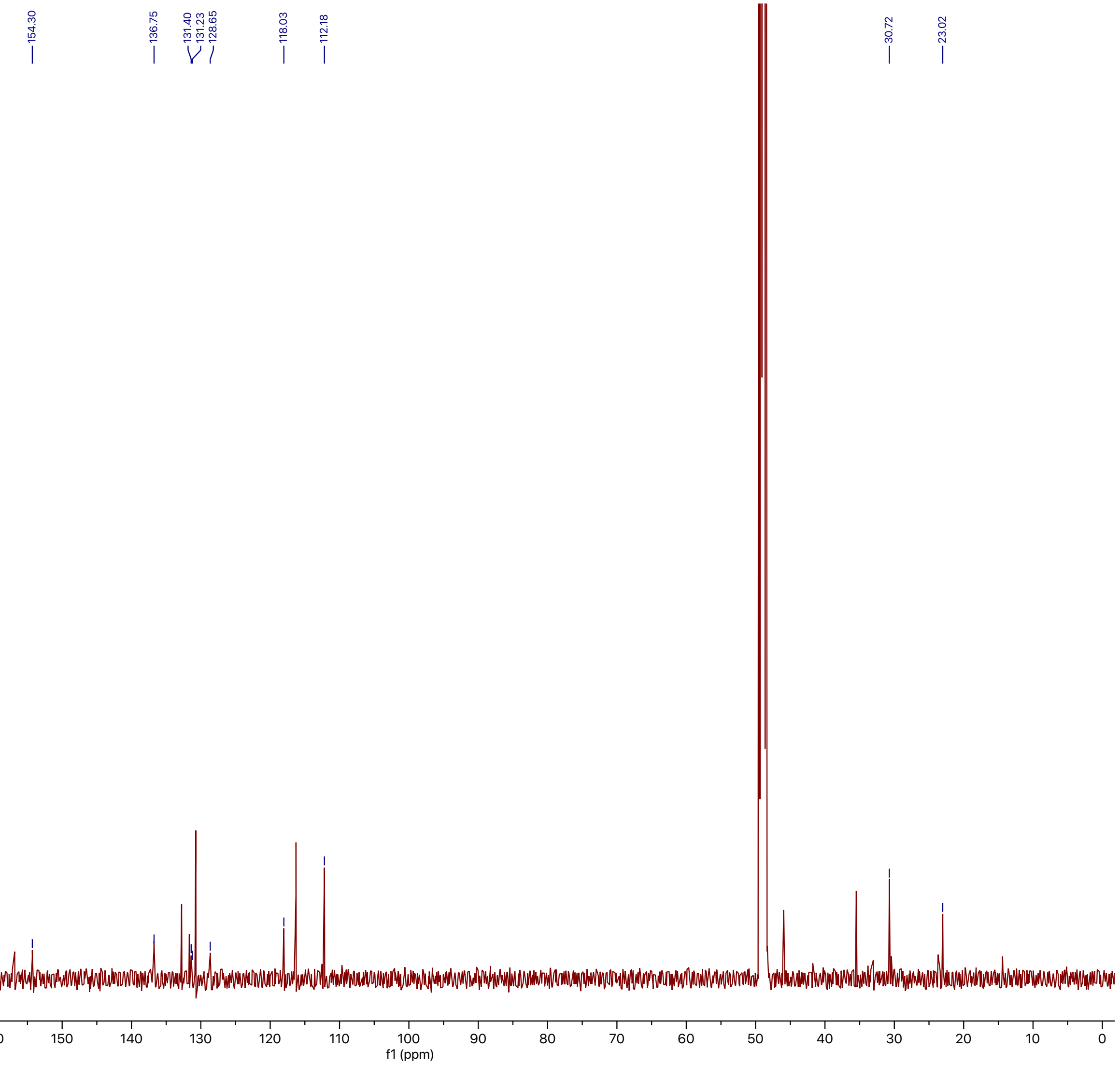

^{13}C-NMR (151 MHz, MeOD) of **5** derived from biosynthesis.

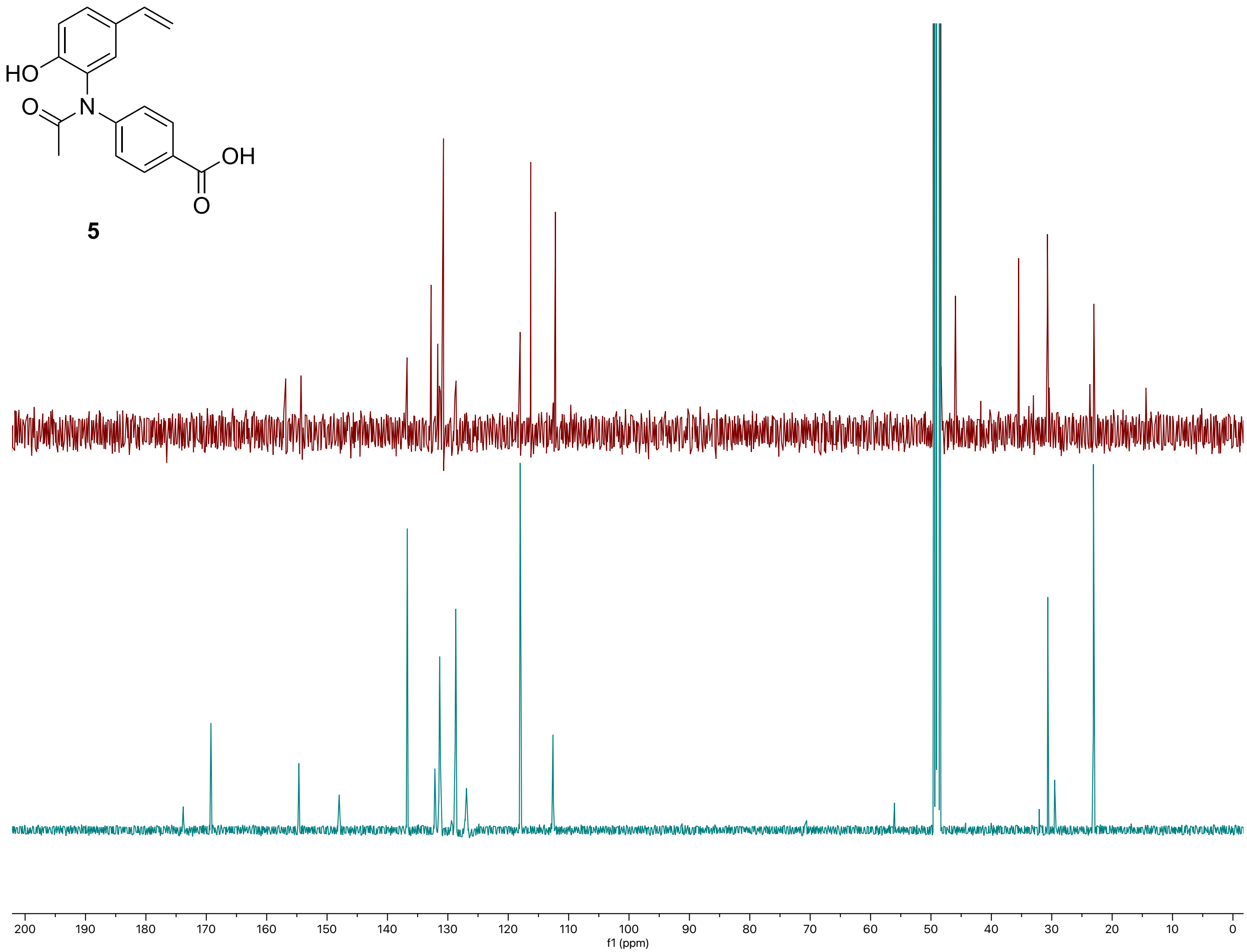

^{13}C-NMR (151 MHz, MeOD) of **5** derived from biosynthesis (top) compared with ^{13}H-NMR (600 MHz, MeOD) of **5** derived from independent synthesis (bottom).

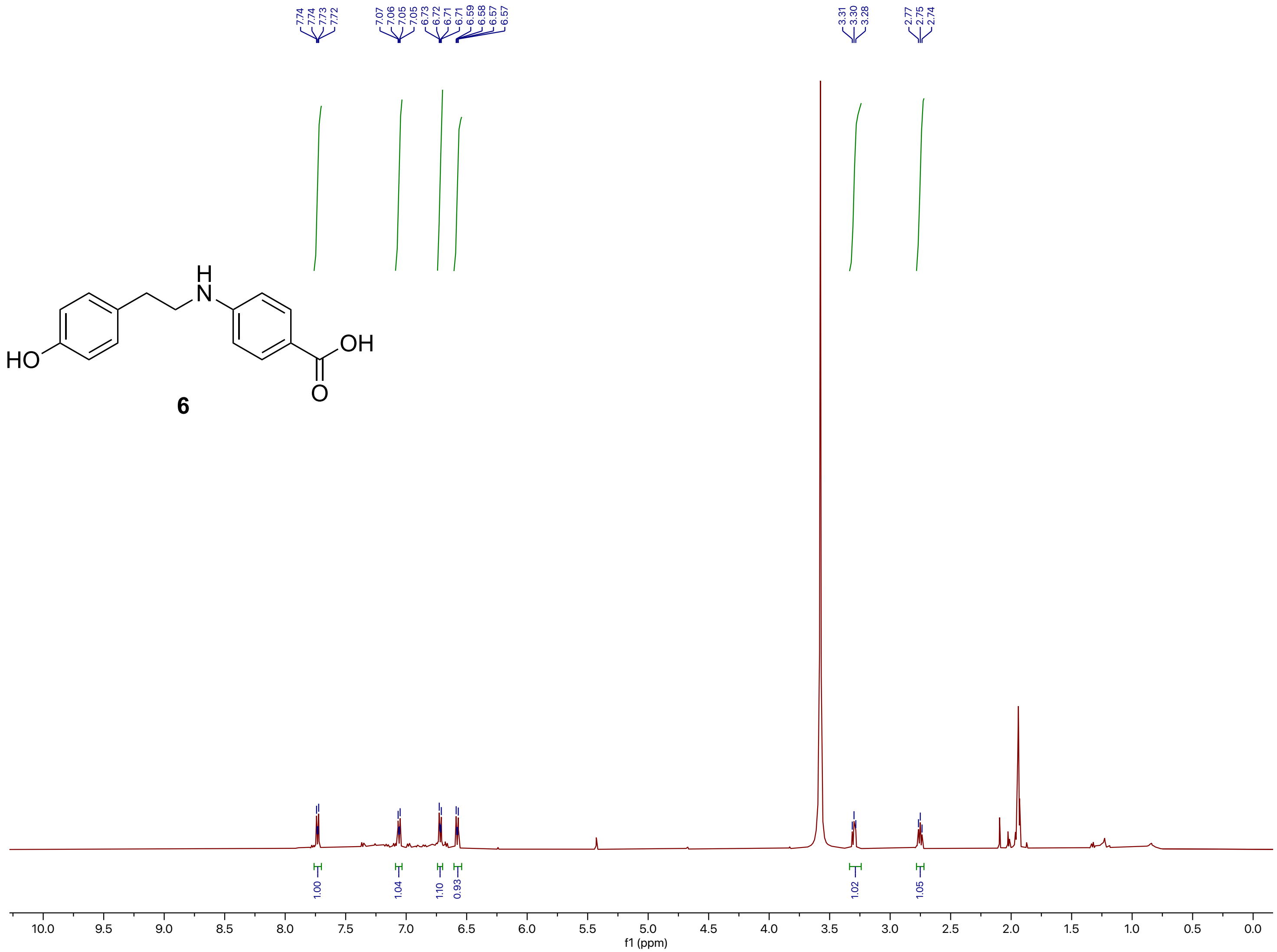

6

^{1}H-NMR (500 MHz, CD_3CN) of **6** derived from biosynthesis.

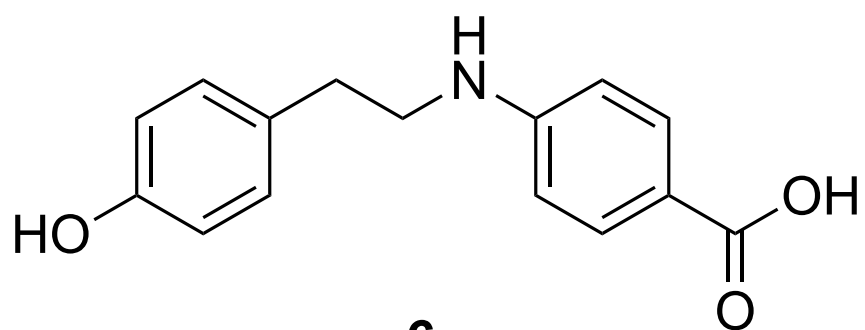

6

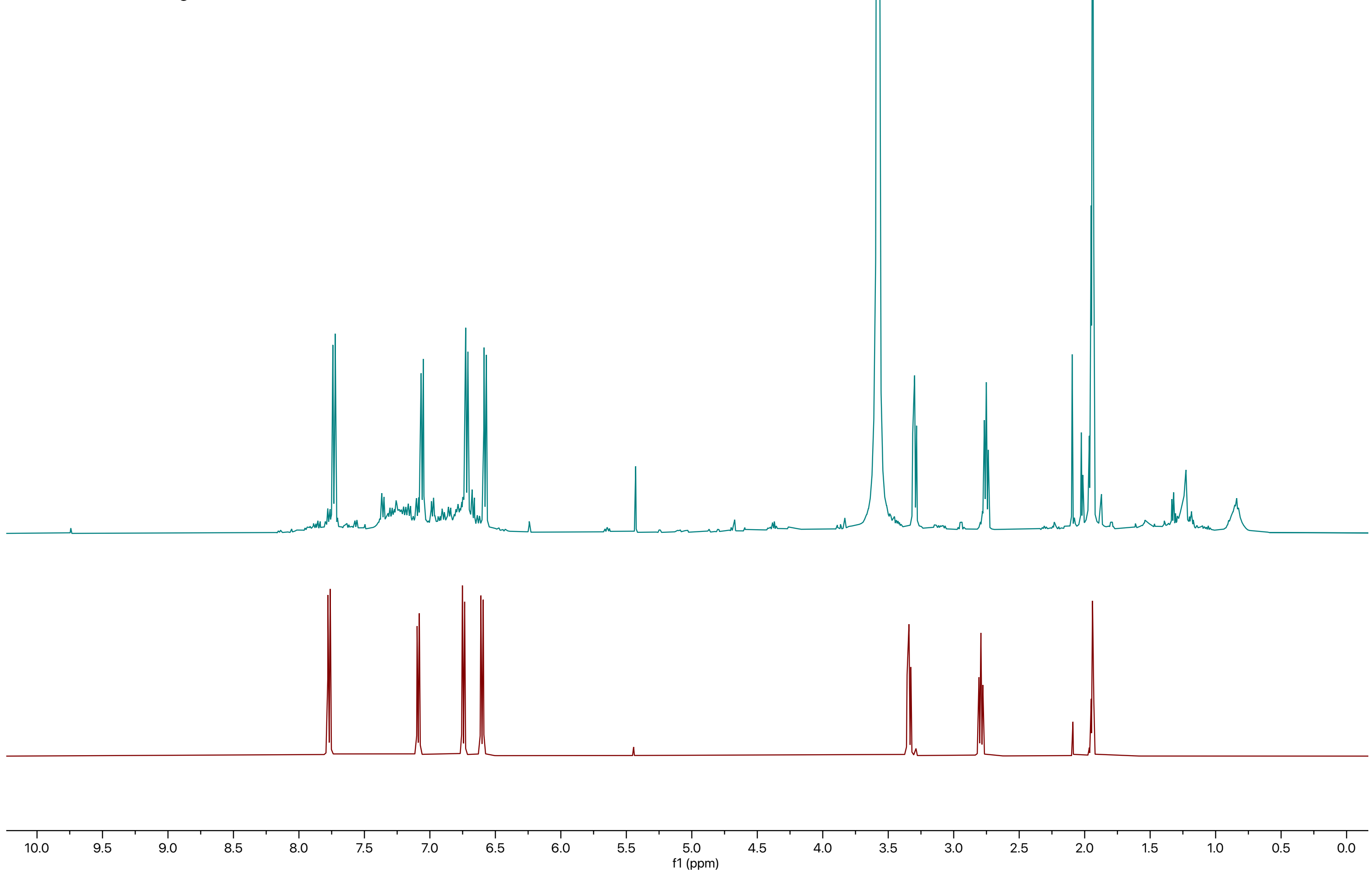

^{1}H-NMR (500 MHz, CD_3CN) of **6** derived from biosynthesis (top) compared with ^{1}H-NMR (500 MHz, CD_3CN) of **6** derived from independent synthesis (bottom).

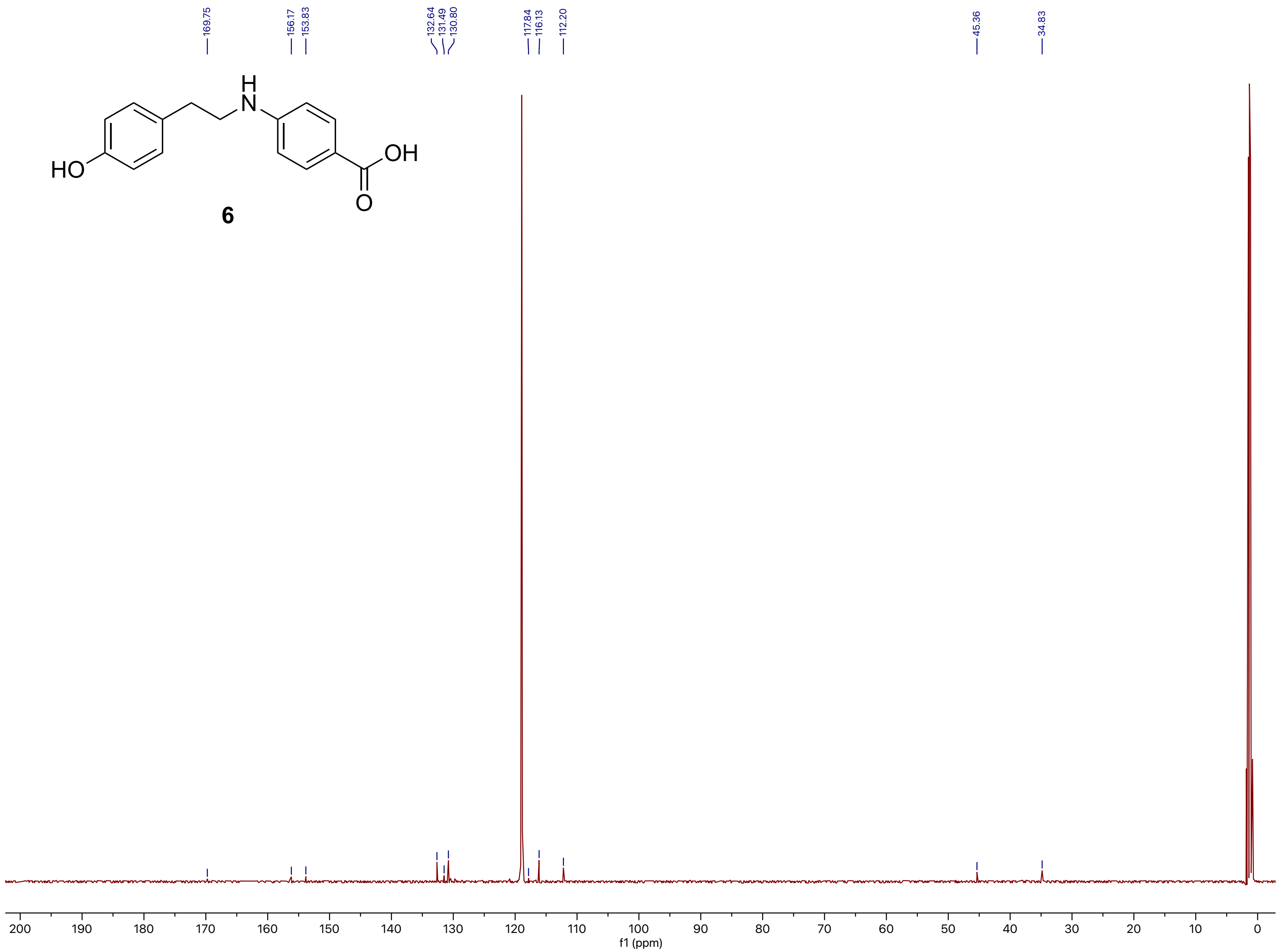

^{13}C-NMR (126 MHz, CD_3CN) of **6** derived from biosynthesis.

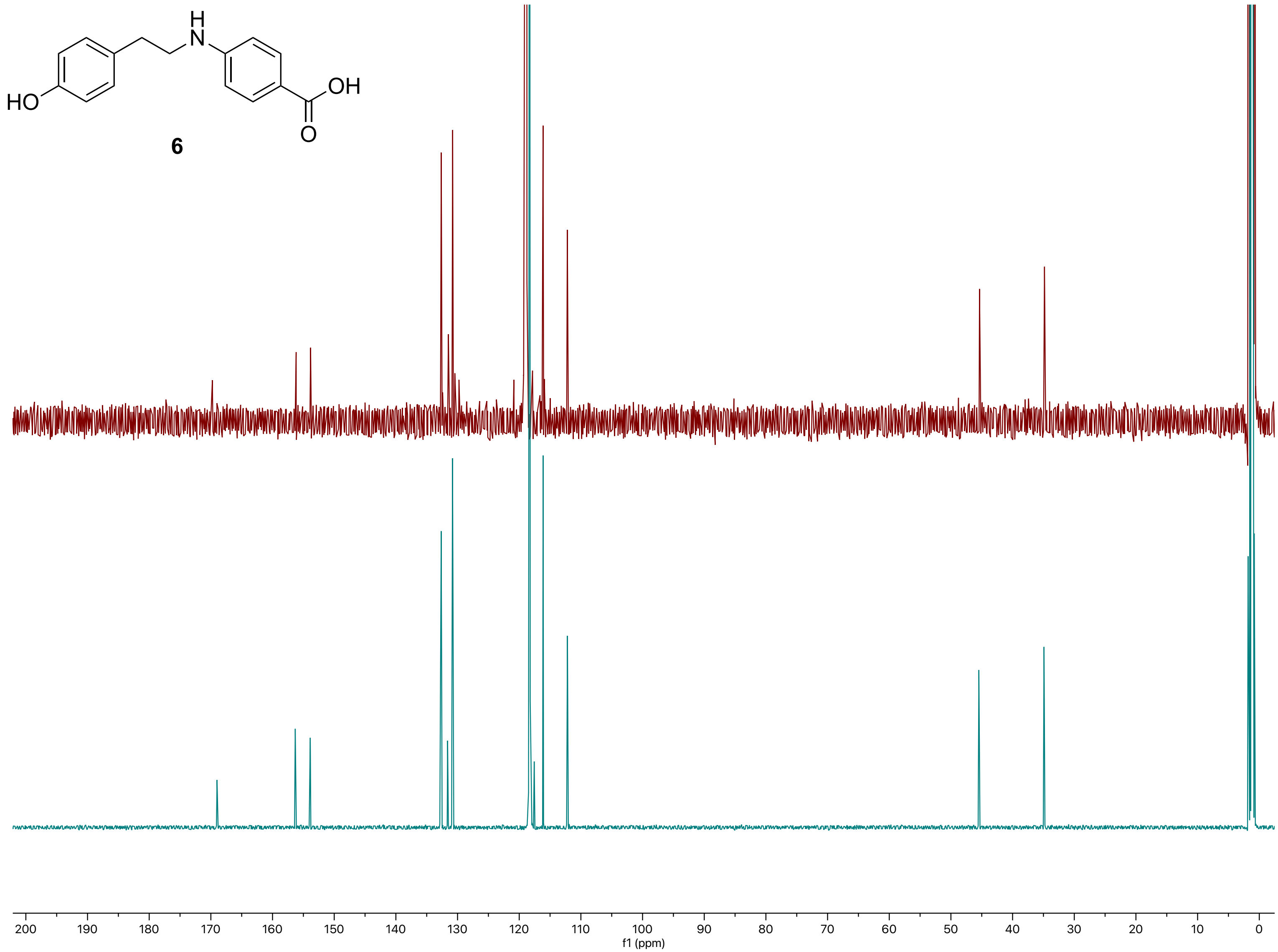

^{13}C-NMR (126 MHz, CD_3CN) of **6** derived from biosynthesis (top) compared with ^{13}C-NMR (126 MHz, CD_3CN) of **6** derived from independent synthesis (bottom).

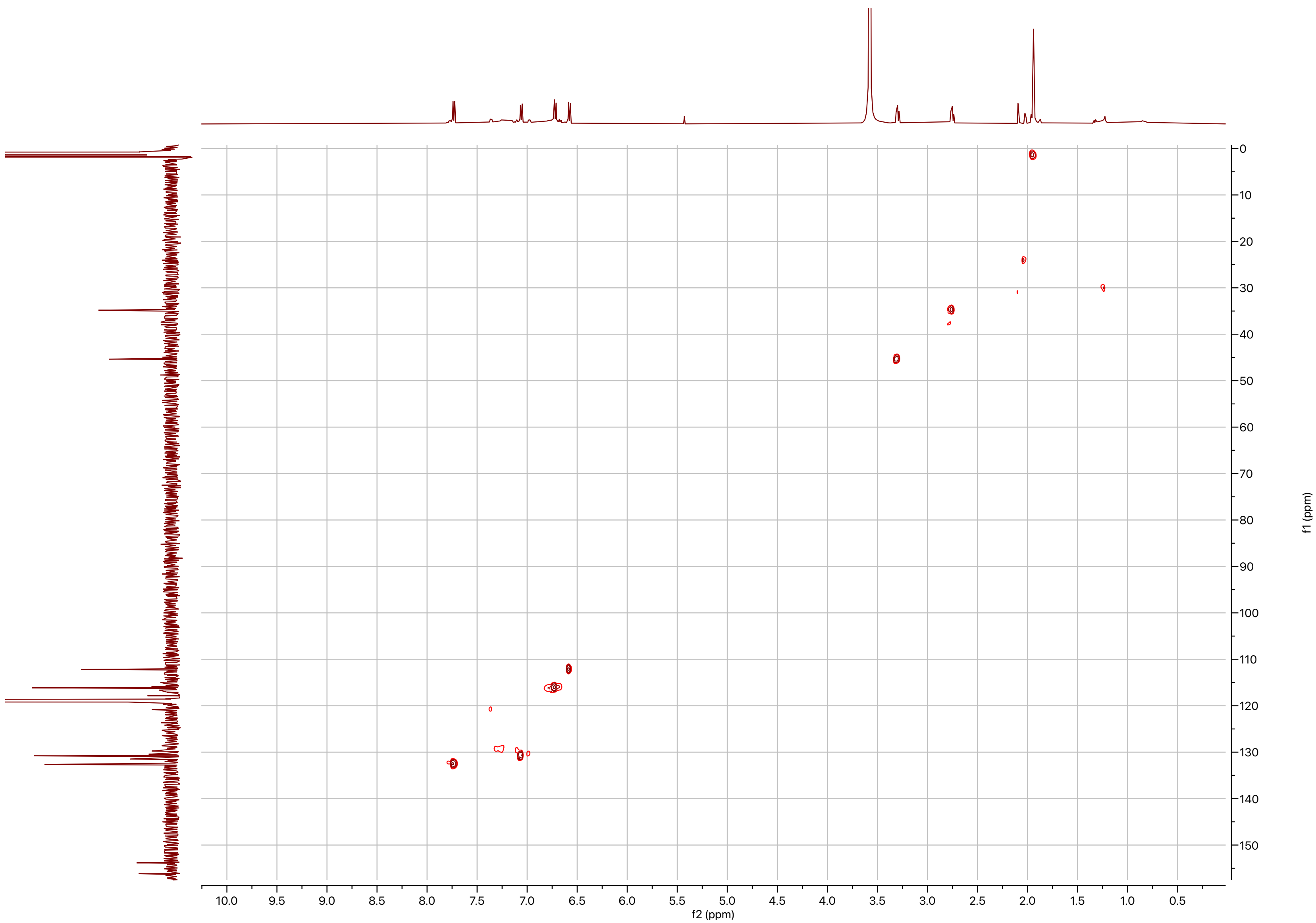

^{1}H-^{13}C HSQC (500 MHz, 126 MHz, CD_3CN) of **6** derived from biosynthesis.

Supplementary Figures

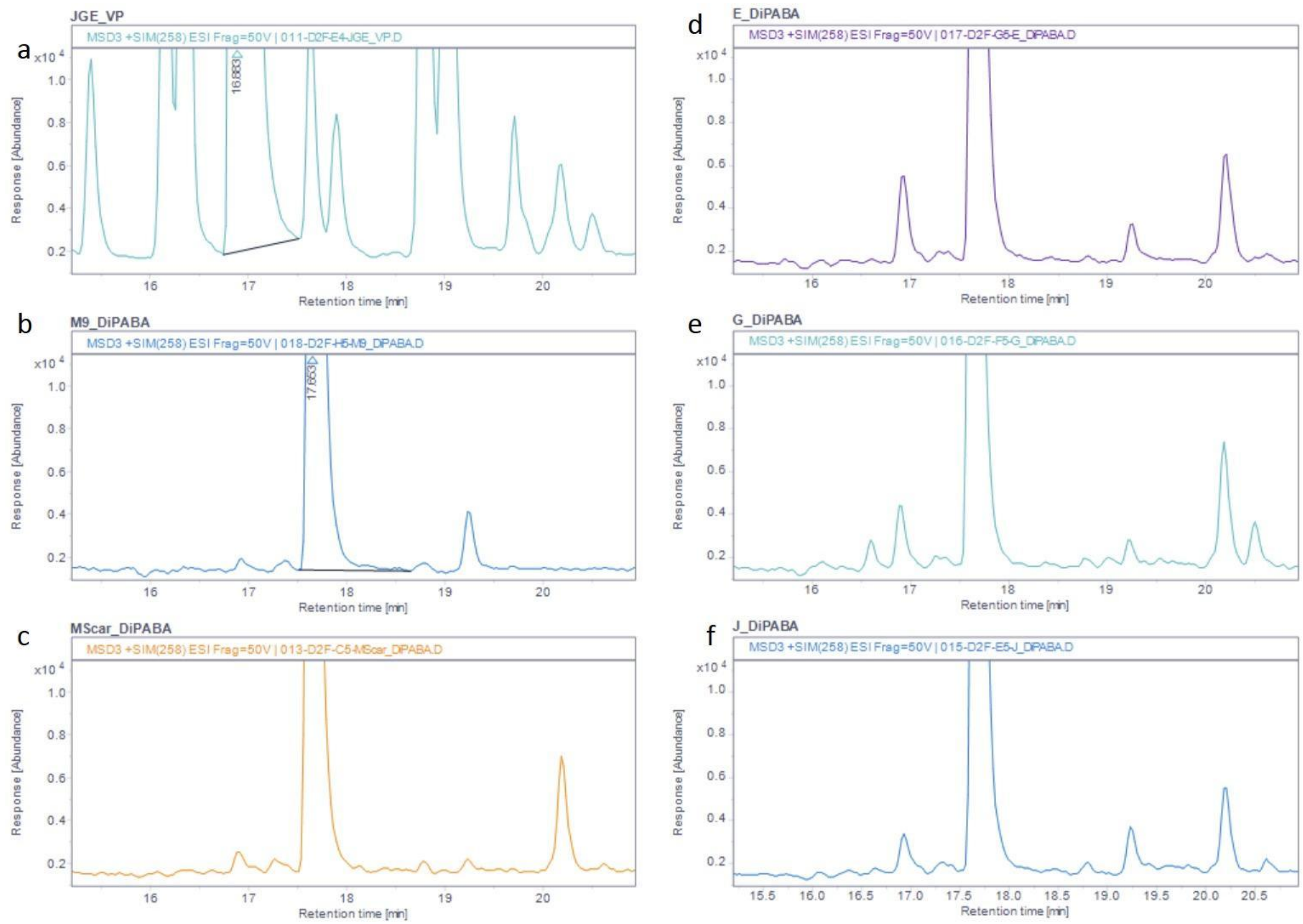

Fig. 1: LC-MSD (SIM, positive mode, 258 *m/z*) of tyramine **6** produced by enzymatic nitrene transfer to 4-VP in strain ID04 (a) or by addition of diamine **2c** to blank M9 (b), BL21 DE3 Star with pMscar, which expresses MScarlet-2I (c), BL21 DE3 Star with pBezEu, which expresses BezEu (d), BL21 DE3 Star with pBezG, which expresses BezG (e), and BL21 DE3 Star with pBezJ, which expresses BezJ (f). These results suggest some biological mechanism for the conversion of **2c** to **6**, however the low yield of the reactions suggest alternative routes for the generation of the tyramine, which are likely mediated during nitrene transfer by the P450.

References

56. Okada, I. Ichimura, K., and Sudo, R. *Bull. Chem. Soc. Jpn.* **1970**, *43*(4), 1185-1189.
57. Brandt, F., Ullrich, M., Laube, M., Kopka, K., Bachmann, M., Löser, R, et al. *J. Med. Chem.* **2021**, *65*(1), 710-733.
58. Key, H.M., Dydio, P., Clark, D.S., and Hartwig, J.F. *Nature*. **2016**, *534*, 534-537.
59. Zhang, H., Cai, Q., and Ma, D. *J. Org. Chem.* **2005**, *70*(13), 5164-5163.
60. Attenburrow, J., Cameron, A.F.B., Chapman, J.H., Evans, R.M., Hems, B.A., Jansen, A.B.A., and Walker, T. *J. Chem. Soc.* **1952**, 1094-1111.
